# Patterns of Freshwater Species Richness, Endemism, and Vulnerability in California

**DOI:** 10.1371/journal.pone.0130710

**Published:** 2015-07-06

**Authors:** Jeanette K. Howard, Kirk R. Klausmeyer, Kurt A. Fesenmyer, Joseph Furnish, Thomas Gardali, Ted Grantham, Jacob V. E. Katz, Sarah Kupferberg, Patrick McIntyre, Peter B. Moyle, Peter R. Ode, Ryan Peek, Rebecca M. Quiñones, Andrew C. Rehn, Nick Santos, Steve Schoenig, Larry Serpa, Jackson D. Shedd, Joe Slusark, Joshua H. Viers, Amber Wright, Scott A. Morrison

**Affiliations:** 1 The Nature Conservancy, San Francisco, California, United States of America; 2 Trout Unlimited, Boise, Idaho, United States of America; 3 USDA Forest Service, Vallejo, California, United States of America; 4 Point Blue Conservation Science, Petaluma, California, United States of America; 5 Center for Watershed Sciences and Department of Wildlife Fish and Conservation Biology, University of California Davis, Davis, California, United States of America; 6 Integrative Biology, University of California, Berkeley, Berkeley, California, United States of America; 7 Biogeographic Data Branch, California Department of Fish and Wildlife, Sacramento, California, United States of America; 8 Aquatic Bioassessment Laboratory, California Department of Fish and Wildlife, Rancho Cordova, California, United States of America; 9 School of Engineering, University of California Merced, Merced, California, United States of America; 10 Department of Biology, University of Hawaii at Manoa, Honolulu, Hawaii, United States of America; Smithsonian's National Zoological Park, UNITED STATES

## Abstract

The ranges and abundances of species that depend on freshwater habitats are declining worldwide. Efforts to counteract those trends are often hampered by a lack of information about species distribution and conservation status and are often strongly biased toward a few well-studied groups. We identified the 3,906 vascular plants, macroinvertebrates, and vertebrates native to California, USA, that depend on fresh water for at least one stage of their life history. We evaluated the conservation status for these taxa using existing government and non-governmental organization assessments (e.g., endangered species act, NatureServe), created a spatial database of locality observations or distribution information from ~400 data sources, and mapped patterns of richness, endemism, and vulnerability. Although nearly half of all taxa with conservation status (n = 1,939) are vulnerable to extinction, only 114 (6%) of those vulnerable taxa have a legal mandate for protection in the form of formal inclusion on a state or federal endangered species list. Endemic taxa are at greater risk than non-endemics, with 90% of the 927 endemic taxa vulnerable to extinction. Records with spatial data were available for a total of 2,276 species (61%). The patterns of species richness differ depending on the taxonomic group analyzed, but are similar across taxonomic level. No particular taxonomic group represents an umbrella for all species, but hotspots of high richness for listed species cover 40% of the hotspots for all other species and 58% of the hotspots for vulnerable freshwater species. By mapping freshwater species hotspots we show locations that represent the top priority for conservation action in the state. This study identifies opportunities to fill gaps in the evaluation of conservation status for freshwater taxa in California, to address the lack of occurrence information for nearly 40% of freshwater taxa and nearly 40% of watersheds in the state, and to implement adequate protections for freshwater taxa where they are currently lacking.

## Introduction

Freshwater habitats cover less than 1% of the earth’s surface (about the size of the European Union) but support roughly 125,000 described species, or 10% of the described species on the planet [1]. Species dependent on freshwater habitats are in decline globally [2, 3]; between 10,000 and 20,000 freshwater species are thought to be extinct or imperiled by human activities [1, 3], with freshwater species at higher risk of extinction than their terrestrial counterparts [4]. In North America, extinction rates for freshwater species are four to five times greater than those for terrestrial species [5–7], and increasing human population and climate change are predicted to exacerbate extinctions in the future [7–10]. Estimates of known extinctions however, are likely gross underestimates because most groups of freshwater organisms are understudied [11]. The insular and fragmented nature of freshwater habitats, which often results in high levels of endemism, makes freshwater populations highly vulnerable to extirpation [1].

Although great strides are being made in the methods to adapt conservation planning principals and conservation strategies to the particularities of freshwater systems [12–13], conservation action is hampered by a lack of basic information about the definition and location of these species. The first stage of systematic conservation planning is compiling information about the location of threatened and rare species in a region [14], but for freshwater species, this information tends to be lacking, dispersed, or focused on limited taxonomic groups even in data rich areas.

Because data is lacking, conservation groups often focus on focal species or taxonomic groups that have better distribution data. Recent studies have attempted to systematically address broad-scale patterns of freshwater species distribution, and spatial congruence among taxonomic groups [4, 15]. These studies show that congruence between taxonomic groups at global and continental scales are low, suggesting that focusing on a single species or taxonomic group may not benefit all freshwater species[4, 15].

California (USA) encompasses an exceptionally diverse array of freshwater ecosystem types, from rivers flowing through temperate rainforests to desert springs where ancient aquifers come to the surface [16]. In addition, demands on California’s freshwater resources to meet human needs are intensifying as its population grows, and climate change further strains an already over-allocated water supply system [17–18]. Water allocations are currently five times the state’s mean annual runoff and, in many of the state’s major river basins, rights to divert water lay claim to up to 1,000% of natural surface water supplies [19].

Recent studies have highlighted dramatic declines of California native fishes with 80% either extinct or threatened with extinction within 100 years [10, 20]. Yet, the composition, distribution, and status of the broader suite of freshwater taxa in the state are not well understood. To address this need, we assembled the first comprehensive database of spatial observations for freshwater vascular plants, macroinvertebrates, and vertebrates in California. Here, we use this new and now publicly available database [21] to evaluate the patterns of freshwater species richness, endemism, and vulnerability, identify hotspots of freshwater richness, and to evaluate the spatial congruence of species richness across taxonomic groups.

## Materials and Methods

### Study Area

**Fig 1:**
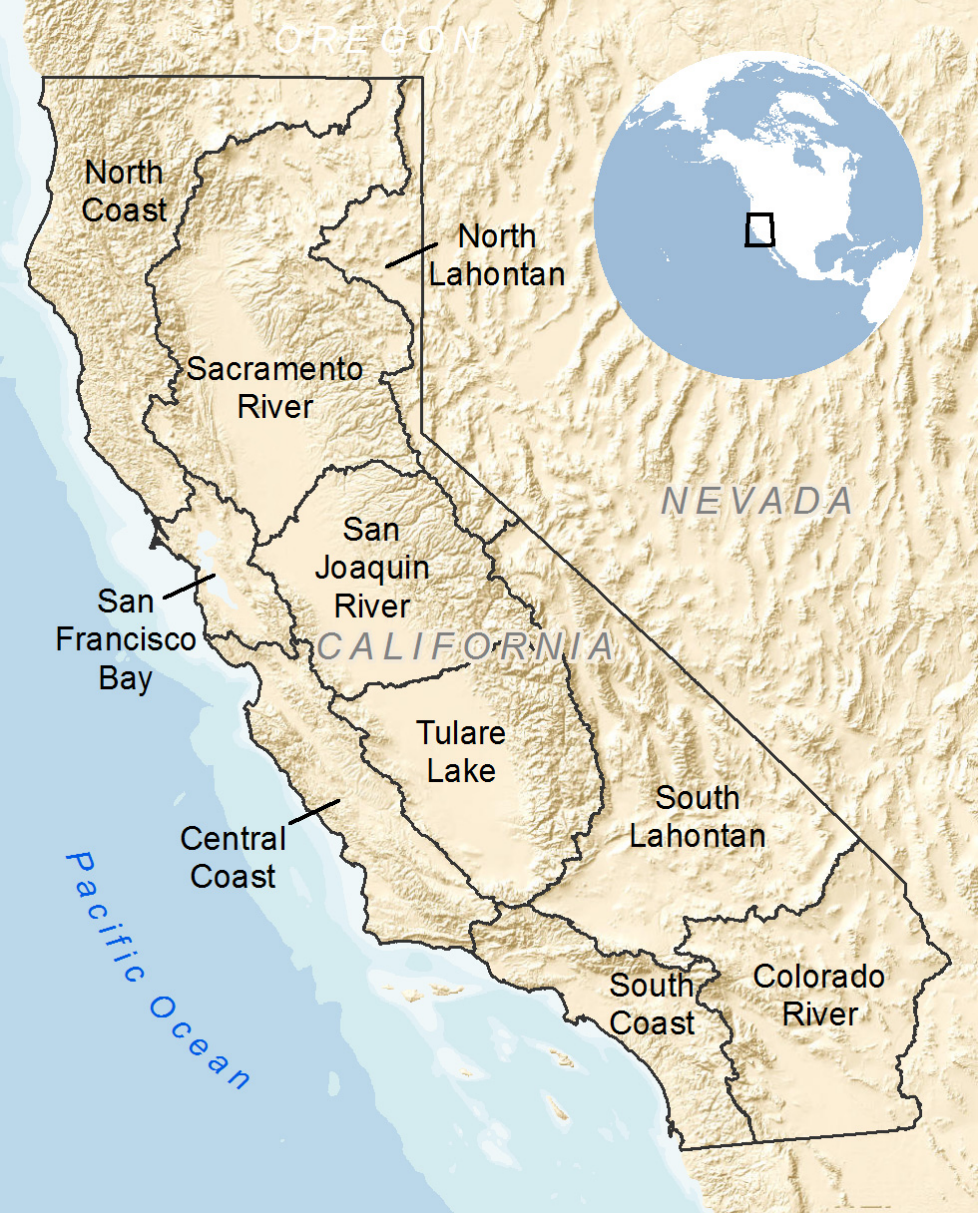
Study area. The extent of the study area in California and the major hydrologic regions it contains. Inset shows the location of California in North America. Shaded relief is from “The National Map” by the U.S. Geological Survey.

The spatial unit of analysis for this assessment was the smallest-size watershed (12-digit hydrologic unit code, or HUC12, watershed) available in the nested national dataset compiled by the US Department of Agriculture Natural Resource Conservation Service [22]. Our study area included those watersheds (n = 4,450) within the administrative boundary of the state of California, totaling 410,515 km^2^ (Fig 1). For reporting results, we nested the HUC12 watersheds within 10 major hydrologic management regions defined by California’s Department of Water Resources corresponding to the state’s major drainage basins [23] (Fig 1)(S1 Table).

### Taxa List

The taxonomic units of analysis for this assessment were drawn from an initial list of species and sub-species known to utilize freshwater habitats within California from NatureServe (http://natureserve.org) (n = 1,903)[24]. Because NatureServe collects and manages information for only a subset of species throughout the U.S., Canada, Latin America, and the Caribbean we assessed regional and specific taxonomic reviews and checklists to identify missing taxa (S2 Table). For example, we relied on the PISCES for all fish data because the software and database is comprehensive and quality-controlled [25–26].

Comprehensive taxonomic reviews are not available in California for non-vascular plants, such as benthic algae and mosses, planktonic microcrustacea, segmented worms, and water mites; consequently, these groups are excluded from our effort. The authors, selected for their taxonomic expertise in the state, compiled and reviewed lists of freshwater dependent species and subspecies that occur within California (S1 Table). The experts removed redundancies due to changes in taxonomy or nomenclature, and assembled a definitive list of freshwater taxa (S3 Table). Our final database augmented the freshwater taxa included in the NatureServe list by 105% (n = 2,003), for a total of 3,906 taxa (S3 Table). Species, subspecies, Evolutionary Significant Units, and Distinct Population Segments are hereafter referred to as “taxa” for convenience.

Criteria for categorizing taxa as “freshwater dependent” varied by taxonomic group (S1 File). For example, freshwater fishes were defined as those that spawn in freshwater habitats. Herpetofauna, were included if: 1) they rely on fresh water to complete one or more life stage (e.g., all anurans and many caudates); or, 2) forage within fresh water as obligates (e.g., western pond turtle, *Actinemys marmorata marmorata*) or non-obligates (e.g., western terrestrial garter snake, *Thamnophis elegans elegans*) at some stage of development; or, 3) they would not persist without freshwater microhabitats (e.g., Inyo mountain salamander, *Batrachoseps campi*); or, 4) they are found within splash zones of freshwater springs and creeks (e.g., Dunn’s salamander, *Plethodon dunni*). Plant species were included if: 1) they occur exclusively in fresh water and have special adaptations for living submerged in water, or at the water's surface; or, 2) occur primarily in freshwater wetland habitats but are not strictly aquatic; or, 3) require freshwater inundation to complete their life-cycle, such as plants occurring in long-inundated portions of vernal pools (e.g., *Orcuttia californica*); or, 4) were identified in the Jepson Manual of Vascular Plants of California [27] as associated with wetland habitats such as marshes, lakes, vernal pools, fens, springs, and bogs, and dependent on wetland habitat; or, 5) were included as wetland obligates or as facultative wetland plants in the U.S. Army Corps of Engineers list of wetland plant species [28]. See S1 File for criteria used for birds, mammals, vascular plants and invertebrates. We limited our list to taxa native to California.

Taxa were classified as endemic if they are known to be restricted to California based on available data sources (S2 Table).

### Conservation Status

We evaluated the conservation status for all taxa on our final list (S3 Table) by reviewing the scientific literature, NatureServe, state and federal Endangered Species Act lists, management agency designations, and taxonomic group reviews (S1 Table). We attempted to be as complete as possible, and use available conservation status sources for the taxonomic groups considered in this study. Table 1 provides sources and criteria for classifying taxon as listed, vulnerable or apparently secure. Note that taxa were not classified as “apparently secure” if they fell under any criteria listed under “vulnerable” in Table 1.

**Table 1:** Sources and criteria used to rank taxa.

Source	Criteria for “listed ranking”	Criteria for “Vulnerable” ranking	Criteria for “Apparently Secure” ranking
ESA federal or state lists [29–30]	• Endangered OR	• Under Review in the Candidate or Petition Process OR	
	• Threatened	• Proposed Threatened OR	
		• Species of Special Concern OR	
		• Candidate OR	
		• Bird of Conservation Concern OR	
		• Special Concern OR	
		• Special	
NatureServe [24]		Ranked at either the global (G) or state (S) scales as:	Ranked at either the global (G) or state (S) scales as:
		• Vulnerable (NatureServe ranking of 3) OR	• Apparently Secure (NatureServe ranking of 4) OR
		• Imperiled (NatureServe ranking of 2) OR Critically imperiled (NatureServe ranking of 1)	• Secure” (NatureServe ranking of 5
Status assessment of California’s native inland fishes [20]		• EN (endangered) OR	• NT (near-threatened) OR
		• VU (vulnerable)(following IUCN definitions)	• LC (least concern)
Conservation Status of Freshwater Gastropods of Canada and the United States [31]		• Endangered OR	Currently Stable (CS)
		• Threatened OR	
		• Vulnerable	
California Native Plant Society – Rare Plant Inventory [32]		• 1A (Plants Presumed Extirpated in California and Either Rare or Extinct Elsewhere) OR	
		• 1B (Plants Rare, Threatened, or Endangered in California and Elsewhere) OR	
		• 2A (Plants Presumed Extirpated in California, But Common Elsewhere) OR	
		• 2B (Plants Rare, Threatened, or Endangered in California, But More Common Elsewhere	
Amphibian and Reptile Species of Special Concern (ARSSC) [33]		Appears on list	
California Department of Fish and Wildlife (CDFW) Species of Special Concern [34]		Appears on list	
USFWS Species of Concern [35]		Appears on list	
USFWS Birds of Conservation Concern [36]		Appears on list	
US Forest Service National Threatened, Endangered and Sensitive Species (TES) Program [37]		Appears on list	
US Bureau of Land Management Special Status Species [38]		Appears on list	

A taxon was classified as listed, vulnerable or apparently secure if one of the criteria conditions were met. For example, if a taxon is classified as endangered on the federal ESA list, we designated the taxon as “listed” in our database. Alternatively, if a taxon was classified as EN (endangered) in Moyle et al. 2011, we classified the taxon as “vulnerable” in our database.

### Spatial Data and Summaries

We collected spatial data related to the occurrence or distribution of the freshwater taxa included on our final list (S3 Table), and assembled a geographic database using Esri ArcGIS version 10.1 software. Due to taxonomic changes and differences among data sources, we were not always able to attribute spatial records at the subspecies level. As a result, all spatial data summaries and analyses are conducted at the species level. Data were collected from a variety of sources (S2 Table). The subsequent database includes available spatial data for each taxon categorized by observation type (Table 2), data format (i.e. point, line, and polygon), origin (i.e. native range vs. translocation), conservation status, and habitat usage (e.g. seasonal or migratory use).

**Table 2:** Classifications used to group spatial data records in the California Freshwater Species Database.

Spatial Data Classification Groups
Current observations (post-1980)
Observation with undefined date
Historical observation (pre-1980)
Extirpated
Modeled habitat/ generalized observation
Expert Opinion
Management area designations*
Range
Historical range
Unknown

* e.g., Critical Habitat designation by the U.S. Fish and Wildlife Service

While this effort represents the most comprehensive compilation of freshwater species occurrence in the state, we acknowledge that data quality may vary among sources. With the exception of PISCES, which has been expert reviewed for data quality, other data sources have not undergone such review, and therefore may not accurately represent species ranges. For example, most invertebrate data come from bioassessment monitoring efforts which are known to under sample certain habitats such as non-perennial streams, large rivers, springs, high altitude streams, and wet meadows.

To examine and compare patterns of freshwater species richness, endemism, and vulnerability, we summed and mapped unique species by HUC12 watershed, and calculated the percentage of species that are endemic, vulnerable, and listed in each watershed. We also mapped richness by eight taxonomic groups (fish, herpteofauna, mollusks, birds, crustaceans, plants, mammals, insects and other invertebrates) by summing the number of species in each taxonomic group within the HUC12s. We identified hotspots as the top 5% richest watersheds [39].

We recognize that spatial data for freshwater species is often lacking, so we tested how each taxonomic group serves as a proxy for the full suite of freshwater species. First we calculated the pairwise Pearson’s correlation coefficient of species richness counts in HUC12 watersheds by taxonomic group to evaluate the relationship between taxonomic groups. Next, we calculated the Pearson’s correlation coefficient for each taxonomic group compared to all other freshwater species not in that taxonomic group. For example, we calculated the correlation coefficient for fish richness compared to all other freshwater species (excluding fish) by HUC12 watershed. In addition, we calculated the correlation of all listed species in each HUC12 compared to all other non-listed species.

We also tested whether geographical patterns of richness in one group act as a surrogate for those in other groups by comparing the overlap of hotspots for one group with corresponding hotspots for other groups [39]. Finally, we compared the hotspots for each group with vulnerable freshwater species to test how well each group acts as a surrogate for vulnerable freshwater biodiversity in most need of conservation action.

## Results

### Richness, Endemism, and Vulnerability

**Fig 2:**
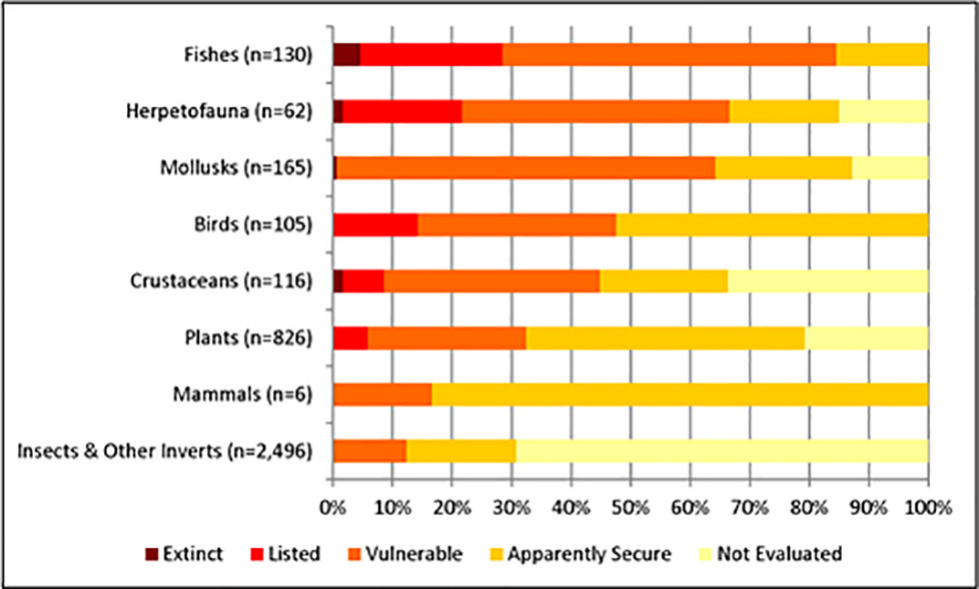
Taxonomic grouping and conservation status of freshwater taxa native to California. Percentage of freshwater species by taxonomic groups that are considered vulnerable (at risk of extinction) in California watersheds, “Insects and other invertebrates” includes the classes Arachnida, Branchiopoda, Insecta and Polychaeta.

We identified 3,906 freshwater taxa in California (S3 Table) which included 336 subspecies, evolutionary significant units, or distinct population segments. Insects, arachnids, branchiopods, and polychaetes (referred to henceforth as “insects and other invertebrates”) comprise over two-thirds (63%) of the freshwater taxa in the study, with 2,496 taxa (Fig 2). The next largest group is vascular plants (n = 826), followed by mollusks (n = 165), fish (n = 130), crustaceans (n = 116) birds (n = 105), herpetofauna (n = 62), and mammals (n = 6) (Table 3). Eleven freshwater taxa that were once found in the study area are now considered extinct, including one plant (*Potentilla multijuga*), two crustaceans (*Pacifastacus nigrescens* and *Syncaris pasadenae*), one mollusk (*Planorbella traski*), one frog (*Rana lithobates] yavapaiensis*), and six fishes (*Cyprinodon nevadensis calidae*, *Siphatales bicolor ssp*. *11*, *Gila crassicauda*, *Pogonichthys ciscoides*, *Ptychocheilus lucius*, and *Salvelinus confluentus*). An additional 14 species considered possibly extinct include eight insects (*Farula davisi*, *Hygrotus artus*, *Mesocapnia bakeri*, *Paraleptophlebia californica*, *Paraleptophlebia clara*, *Paraleptophlebia rufivenosa*, *Parapsyche extensa*, *Rhyacophila amabilis*), two amphibians (*Rana pretiosa*, and *Incilius alvarius*), one mollusk (*Valvata virens*), two plants (*Plagiobothrys glaber* and *Potentilla uliginosa*), and one turtle (*Kinosternon sonoriense*).

**Table 3:** Number of taxa included in each taxonomic group along with the number and percentage of species by conservation status.

Group	All	Extinct	Listed	Vulnerable (but not listed)	Apparently Secure	Not Evaluated
Insects and Other Inverts*	2,496	0	0	309 (12%)	460 (18%)	1,727 (70%)
Plants^1^	826	1 (0%)	47 (5%)	220 (27%)	387 (47%)	171 (21%)
Mollusks	165	1 (0.5%)	0	105 (63.5%)	38 (23%)	21 (13%)
Fishes	130	6 (5%)	31 (24%)	73 (56%)	20 (15%)	0
Crustaceans	116	2 (2%)	8 (7%)	42 (36%)	25 (21%)	39 (34%)
Birds	105	0	15 (14%)	35 (34%)	55 (52%)	0
Herpetofauna	62	1 (2%)	12 (19%)	29 (46%)	11 (18%)	9 (15%)
Mammals	6	0	0	1 (17%)	5 (83%)	0
Total	3,906	11 (0.3%)	113 (3%)	814 (21%)	1,001 (26%)	1,967 (50%)

* Includes Arachnida, Branchiopoda, Insecta and Polychaeta.

^1^All California plants are evaluated for rarity. Due to the lack of a ‘secure’ category in the CNPS ranking system, common taxa may not appear to have been evaluated.

To date, conservation status has been assessed for only 50% (N = 1,939) of the state’s freshwater taxa (Table 3). Moreover, the conservation status of some taxonomic groups is disproportionally understudied. For example, the conservation status of all fish and bird taxa have been evaluated, but only 31% (n = 769) of insects and other invertebrates (Table 3). Of the freshwater taxa evaluated, 51.5% are considered secure (n = 1,001), 48% are ranked as vulnerable (n = 927), and 0.5% (n = 11) are now considered extinct. Although nearly half of the freshwater taxa were classified as vulnerable, only 113 (6%) are listed as endangered or threatened under the federal or state ESA.

Some taxonomic groups contain considerably more vulnerable taxa than others (Fig 2). For example, 104 of the 130 (80%) fishes, 66% of herpetofauna (n = 41) and 64% (n = 105) of mollusks are considered vulnerable. On the other hand, 83% of the mammals, 52% of the birds, and 47% of the plants are considered secure. However, as noted above, the comprehensiveness of data varies by taxonomic group such that the true level of imperilment could be much greater for taxonomic groups such as insects, other invertebrates and crustaceans where the majority of known taxa have not been evaluated for conservation state (Fig 2 and Table 3).

**Fig 3:**
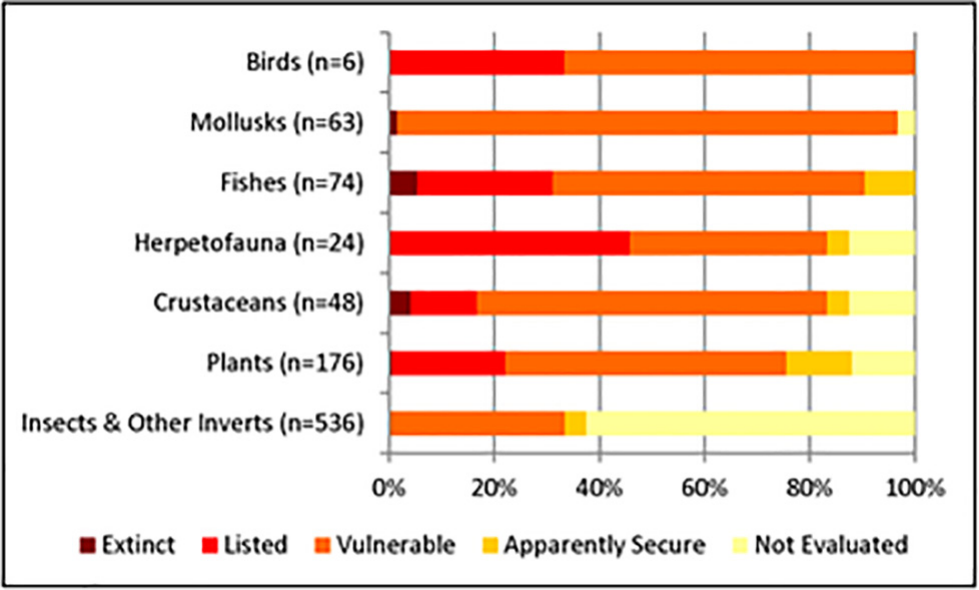
Taxonomic grouping and conservation rank of freshwater taxa endemic to study area. Percentage of freshwater endemic species by taxonomic groups that are considered vulnerable (at risk of extinction) in California watersheds. “Insects and other invertebrates” includes the classes Arachnida, Branchiopoda, Insecta and Polychaeta.

Nearly a quarter of the 3,906 native freshwater taxa found in California are endemic (n = 927), including 536 insects and other non-molluscan invertebrates, 176 plants, 74 fishes, 63 mollusks, 48 crustaceans, 24 herpetofauna, and 6 birds (Fig 3). Of the 560 endemic taxa that were evaluated for conservation status, nearly 90% (n = 498) are considered vulnerable (Fig 3). All 6 endemic birds are considered vulnerable, as are 98% of the endemic mollusk taxa. In addition, 85% of endemic fishes are considered vulnerable (Fig 3). Eight endemic taxa are considered extinct including four fishes (*Cyprinodon nevadensis calidae*, *Siphatales bicolor ssp*. *11*, *Gila crassicauda*, and *Pogonichthys ciscoides*), two crustaceans (*Pacifastacus nigrescens* and *Syncaris pasadenae)*, one plant (*Potentilla multijuga*) and one mollusk (*Planorbella traski*). Only 76 (14%) of vulnerable endemic taxa are formally listed on state or federal endangered species lists.

### Spatial Data and Summaries

To map spatial patterns of freshwater diversity in the state, we compiled spatial data from 408 different sources (S2 Table) and assembled a database with over 9,000 polygon, 23,000 line, and 3,484,000 point records. As noted in the above Methods, we compiled spatial data only at the species level. Therefore, although our final species list contains information on 3,906 taxa, we compiled spatial data for the 3,727 species in the database. It should be noted that although there are 336 subspecies, ESUs, or DPSs in the database, 179 species are comprised of at least two subspecies.

We obtained spatial data (see Table 2 for data types) for 2,276 (61%) of the 3,727 total freshwater species, including 588 (68%) of the 862 endemic species, 752 (90%) of the 838 vulnerable species, and all 94 species listed under state or federal Endangered Species Acts [29–30]. We were unable to find any spatially explicit data for 1,451 (39%) of the species.

**Fig 4:**
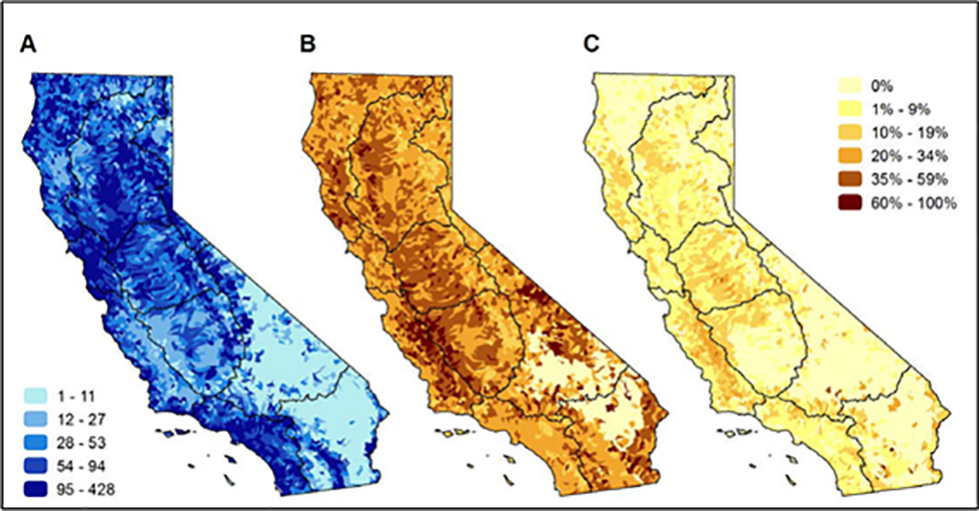
Patterns of richness and vulnerability of freshwater species native to California watersheds. Maps of (A) the number of native freshwater species in each HUC12 watershed (includes current, historic, range and modeled data). The range of species richness is shown in quintiles, therefore the darkest blue is the top 20% of species richness, the lightest blue the lowest 20%.; (B) percentage of species in each HUC12 watershed that are ranked as vulnerable; and (C) percentage of species in each HUC12 watershed that are listed as endangered or threatened under state or federal ESA lists. Maps in panels B and C share the legend on the right of the figure. The black lines on the maps represent the major hydrologic regions in the study area.

Hydrologic regions with the greatest species richness include portions of the San Francisco Bay (average species richness by HUC12 = 111 species), South Coast (average species richness by HUC12 = 91) and Sacramento River (average species richness by HUC12 = 74) (Fig 4A). The average richness of vulnerable taxa per HUC12 by hydrologic regions was greatest in the San Francisco Bay (n = 31), South Coast and San Joaquin (n = 22), Sacramento (n = 21), and North Coast (n = 19). However, the regions with the highest percentage of vulnerable species per HUC12 are the South Lahontan, Tulare Lake, South Coast, Colorado, and Central Coast regions (Fig 4B). Listed species are found across the study area with at least one being as either currently or historically found in watersheds that cover 76% of the state (Fig 4C). However, in contrast to vulnerable species (Fig 4B), the proportion of listed species per HUC12 watershed is relatively low (Fig 4C).

**Fig 5:**
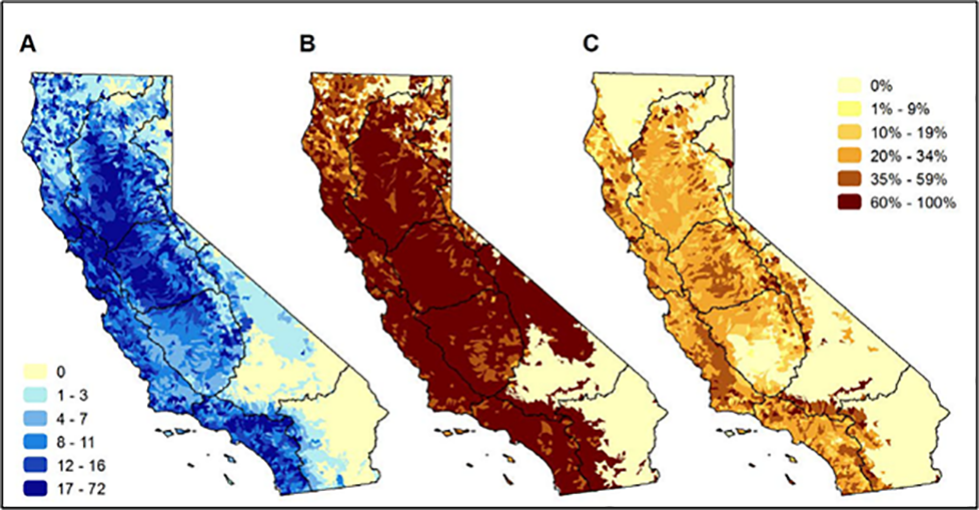
Patterns of richness and vulnerability of freshwater species endemic to California, watersheds. Maps of (A) the number of endemic freshwater species in each HUC12 watershed (includes current, historic, range and modeled data). The range of endemic species richness is shown in quintiles, therefore the darkest blue is the top 20% of species richness, the lightest blue the lowest 20%.; (; (B) percentage of endemic species considered vulnerable in each HUC12 watershed; and (C) percentage of endemic species in each HUC12 watershed that are listed as endangered or threatened under state or federal ESA lists. Maps in panels B and C share the legend on the right of the figure. The black lines on the maps represent the major hydrologic regions in the study area.

The average richness of endemic taxa per HUC12 by hydrologic regions was greatest in the San Francisco Bay (n = 19), San Joaquin (n = 15), South Coast (n = 14), Sacramento (n = 12), and the Central Coast (n = 11) (Fig 5A). Regions with hydrological connections outside of California–North Coast, North and South Lahontan, and Colorado River–have a lower percentage of California endemic species (n = 7, 5, 3, 4 on average, respectively). More than half of the study area (61%) is comprised of HUC12 watersheds in which over 60% of the endemic species found in those watersheds are considered vulnerable (Fig 5B). As with all native freshwater species, the proportion of endemic species that are listed under state or federal ESA lists is considerably less than the proportion of those considered vulnerable in most HUC12 watersheds (Fig 5C).

**Fig 6:**
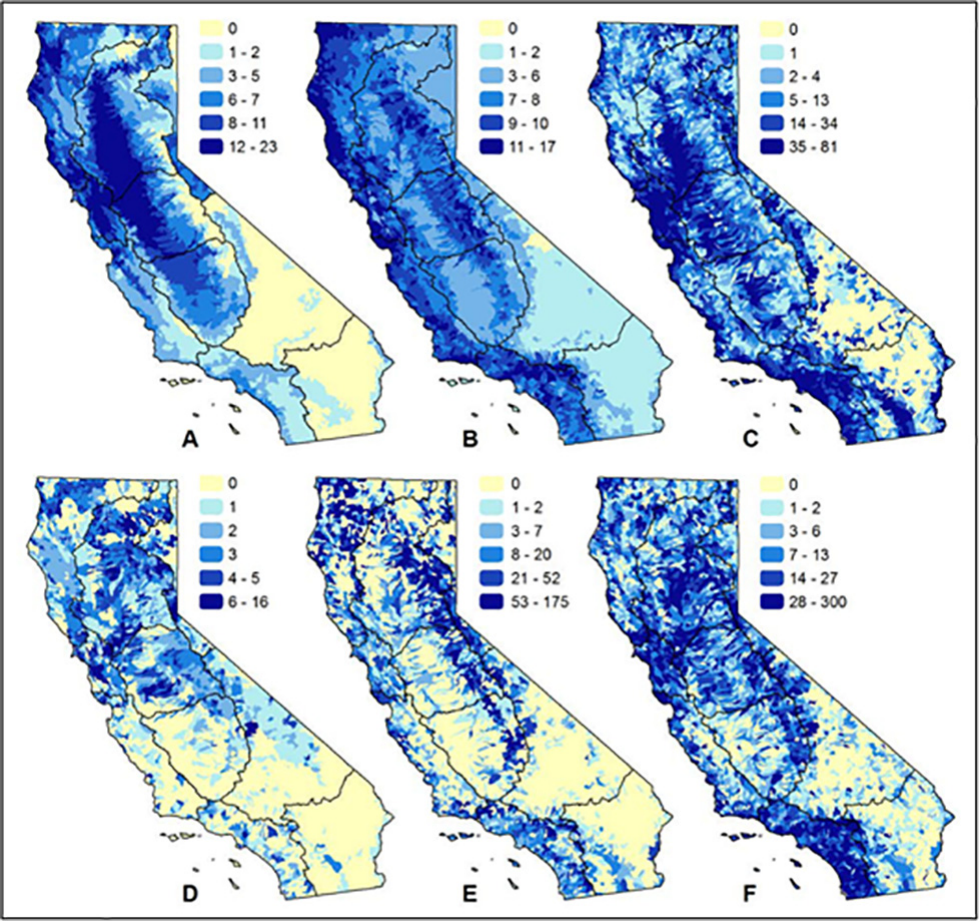
Patterns of freshwater species richness by taxonomic group. Maps show richness of: (A) fishes; (B) herpetofauna; (C) birds; (D) mollusks/crustaceans; (E) insects and other invertebrates; (F) plants.

Spatial patterns of richness vary by taxonomic group and appear to correspond with distribution of freshwater habitat (Fig 6). For example, fish richness is highest in major rivers in the state including the Sacramento and Klamath river watersheds located in the Sacramento and North Coast hydrologic regions (Fig 6A)(S1 Table). Herpetofauna richness is highest in mountain foothill and coastal areas (Fig 6B), with bird richness being highest in wetland, coastal, and compatible agriculture (e.g., flooded rice) regions of the state (Fig 6C). Richness of mollusks/crustaceans, insects and other invertebrates is concentrated in headwater, spring systems and more isolated pockets of habitat (Fig 6D and 6E). Plant richness appears distributed throughout the state with pockets of high richness even in desert regions which are underrepresented by other taxonomic groups (Fig 6F).

**Fig 7:**
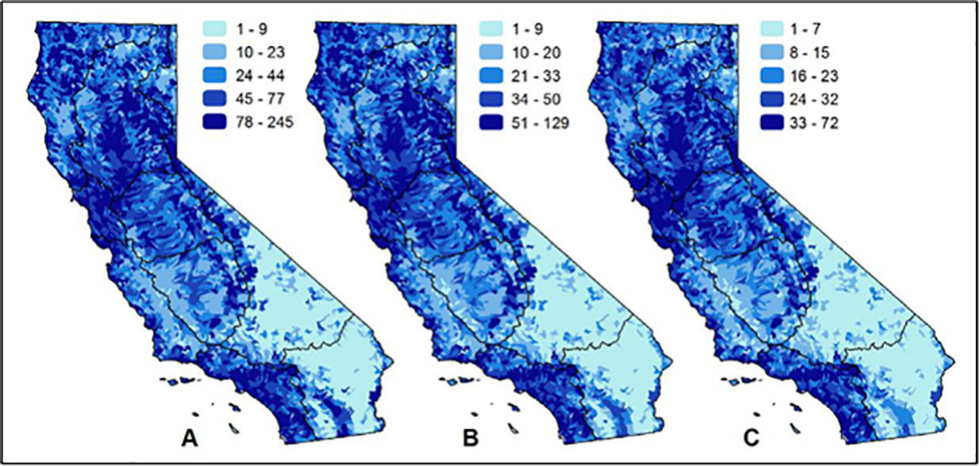
Patterns of richness by data type of California freshwater species. Maps show the number of native freshwater species when summarized by: (A) observational data recorded after 1980; (B) observational data recorded before 1980 or observations of extirpated populations; and (C) data that includes range maps, historical range maps, modeled habitat, professional judgment, critical habitat designations, and management area designations. Spatial data with an unknown observation date or unknown type are not included in any panel. The black lines on the maps represent the major hydrologic regions in the study area.

Geographies noted for high species richness are consistent regardless of observation type (Table 2). The San Francisco Bay, Sacramento River, and portions of the South Coast hydrologic regions are consistently identified as biodiversity hotpots whether observational, range, or modeled data are considered (Fig 7). Patterns of diversity for historical observations and extirpated populations appear similar to current observations (Fig 7A and 7B). Modeled and generalized data such as range maps completely cover the study area and provide perhaps the clearest pattern of diversity of freshwater taxa (Fig 7C); however, these patterns are only predictions of taxa presence. Nearly 40% of the study area does not contain a recent (post-1980) observation for any of the freshwater taxa considered in this study (Fig 7A).

The correlation coefficients of species richness at the HUC12 watershed scale between the various taxonomic groups are relatively low (Table 4), with the highest being between mollusks and mammals (0.52); fishes and mammals (0.52); and fishes and herps (0.51). The lowest correlations coefficients are between insects and other inverts and birds (0.03); crustaceans (0.06) and fishes (0.07).

**Table 4:** Correlation matrix of the richness within each HUC12 watershed summarized by taxonomic groups.

	Fishes	Crustaceans	Herps	Insects & Other Inverts	Mollusks	Plants	Birds	Mammals
**Fishes**	1.00	0.33	0.51	0.07	0.35	0.22	0.42	0.52
**Crustaceans**		1.00	0.09	0.06	0.14	0.20	0.26	0.11
**Herps**			1.00	0.32	0.32	0.34	0.32	0.32
**Insects & Other Inverts**				1.00	0.44	0.26	0.03	0.28
**Mollusks**					1.00	0.23	0.17	0.52
**Plants**						1.00	0.38	0.15
**Birds**							1.00	0.09
**Mammals**								1.00

**Fig 8:**
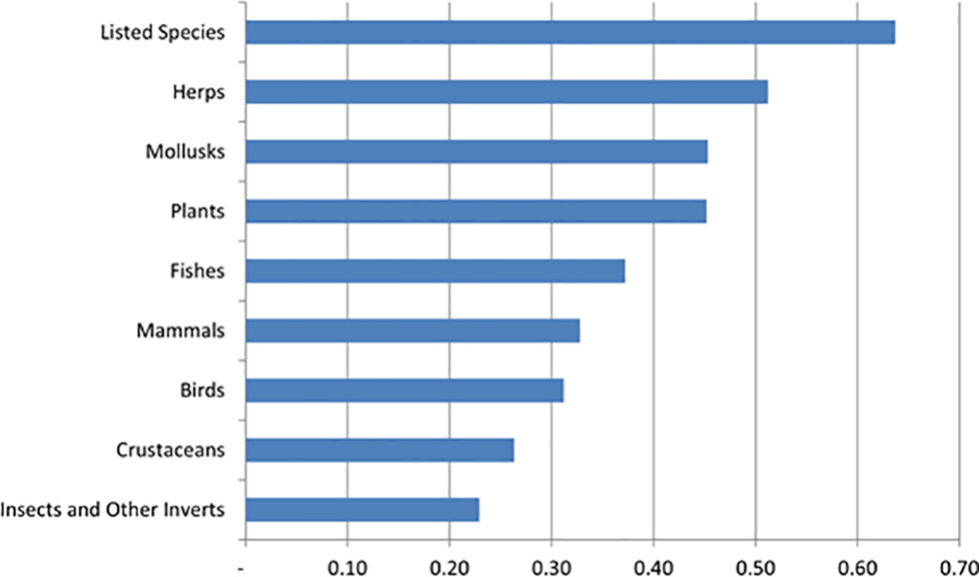
Relationship among taxonomic groups. Correlation of the richness within each HUC12 watershed for taxonomic groups of species when compared to all other freshwater species (excluding that group).

**Fig 9:**
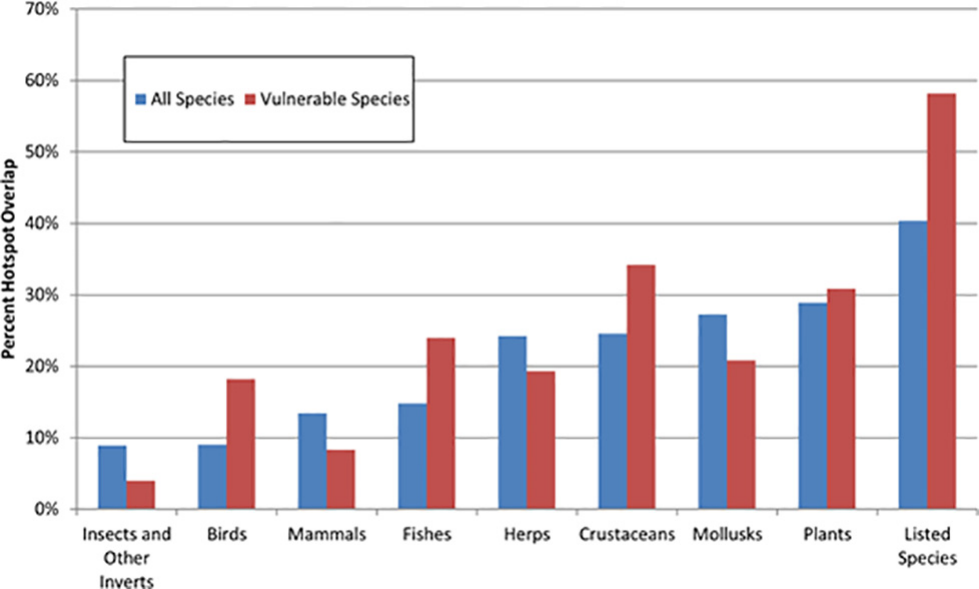
Overlap of hotspots. The relative performance of hotspots (top 5% of watersheds by richness) for taxonomic groups of species in matching hotspots for all (blue bars) and vulnerable (red bars) freshwater species. To avoid double counting, hotspots for all and vulnerable species were identified excluding the species in each subgroup for each comparison.

**Fig 10:**
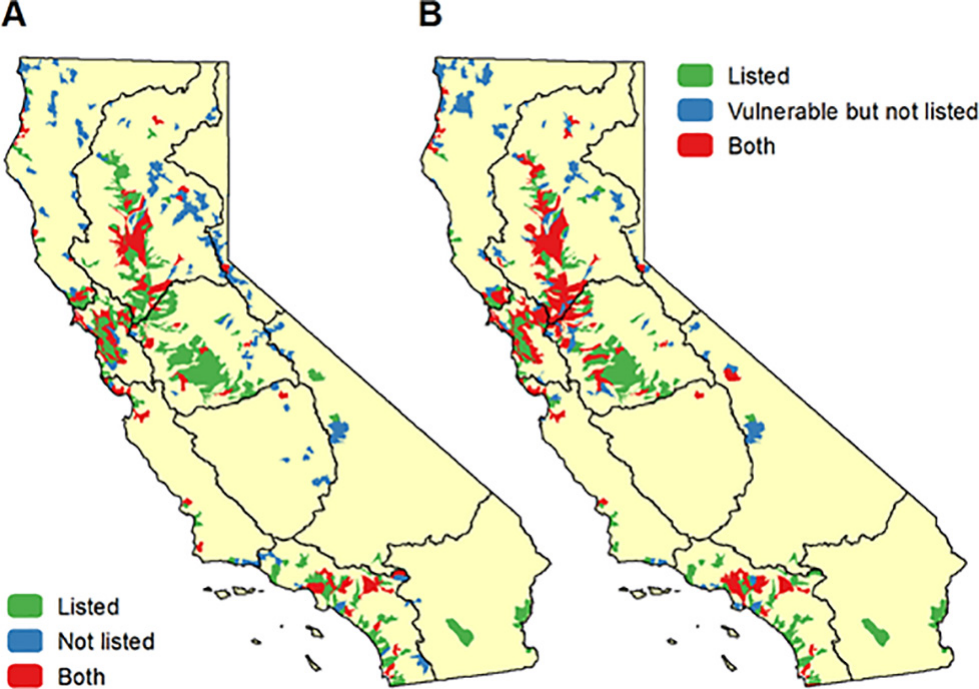
Location of hotspots. Comparison of the location of hotspot watersheds (top 5% by richness) for A) listed species with all non-listed species, and B) vulnerable but non-listed species.

We tested how the richness of various groups of species (taxonomic groups and listed species) serve as a proxy for the richness of all other freshwater species using correlation and hotspot overlap analysis. Listed species were the most correlated at the HUC12 watershed scale with the richness of all other freshwater species (0.63), followed by herpetofauna (0.51) and mollusks and plants (0.45) (Fig 8). Insects and other invertebrates had the lowest correlation to all other species (0.23). With the hotspot overlap analysis, we found again that listed species serve as the best proxy for all other species, with a 40% overlap in hotspots, followed by plants (29%), mollusks (27%) and crustaceans (25%) (Fig 9). We also compared hotspots for each group with hotspots of vulnerable freshwater species, since these are in the highest need of conservation action. Hotspots for listed species overlapped with 58% of the hotspots for vulnerable freshwater species (excluding listed species). Mapping the hotspots shows that hotspots for listed species overlap with hotspots for all other species in the Sacramento River, San Francisco Bay, and South Coast hydrologic regions (Fig 10). However, hotspots congruence is lower in the North Coast and San Joaquin hydrologic regions.

## Discussion

We compiled the most comprehensive database of freshwater species richness and distribution for the state of California to date. Using that database, we provide the first multi-taxa analysis of richness, endemism, and vulnerability for the majority of freshwater diversity in the state. Our study finds that the plight of freshwater species in California mirrors global trends [1–3]. We found that nearly half of freshwater taxa native to California are considered vulnerable to extinction, however only 6% of those taxa are currently listed under state or federal ESA. Even more disconcerting is that 90% of the freshwater taxa endemic to California—and so wholly reliant on conservation actions within the state—are vulnerable to extinction. However, only 14% of these endemic taxa are listed under state or federal ESAs (Fig 3). Therefore, legal listing does not appear to accurately reflect the state of vulnerability of freshwater taxa in the state.

We found that freshwater fishes, amphibians, reptiles, and mollusks are the most vulnerable taxonomic groups, a finding that is consistent with other studies [5, 10, 40–42]. However, this finding could be biased by the general lack of information about vulnerability of other taxonomic groups (Fig 3). These results provide evidence that some taxonomic groups are much better evaluated for conservation status than others (Table 3). For example, all fish and bird taxa have been evaluated as have most of the reptiles, amphibians, plants, and mollusks. In contrast, only 31% of the insects and other invertebrates have been evaluated for conservation status. Furthermore, we still lack spatially-explicit information for 1,448 freshwater species, including many known or suspected to be vulnerable to extinction. Evaluating the conservation status and locations of understudied freshwater species is priority for future research. Given that data acquisition is costly and time intensive, a recent study has shown that concentrating survey efforts on species with the highest uncertainty, such as rare species, provides an effective way to enhance the accuracy of conservation planning [43].

While there are some significant data gaps in our knowledge about the locations of many freshwater species, we were able to compile spatial data for 90% of the vulnerable species in the state, and all of the listed species. With this rich dataset, we were able to test how well a conservation focus on a particular subset of species would benefit other freshwater species. We found that a conservation focus on hotspots for a single taxonomic group such as fishes would provide poor overlap with hotspots for all other freshwater species. Our results are similar to a recent study on global patterns of freshwater species distribution [4]. Interestingly, we found that listed species do provide a reasonable proxy for other freshwater species, since hotspots for listed species cover 40% of the hotspots for non-listed species and 58% of the hotspots for non-listed vulnerable species (Figs 9 and 10). In our study area, focusing conservation action on the hotspots for listed species will likely benefit other freshwater species that need conservation action but have not yet been listed. If these patterns hold for other locations, this finding has implications for conservation strategies outside of our study area because there is generally more spatially explicit information about the distribution of listed species.

The publicly-available dataset [21] we have produced provides a means to place a wide range of freshwater management actions, including water rights administration and water use permitting within the larger context of freshwater-dependent species conservation. Furthermore, the dataset supports conservation planning initiatives by federal and state agencies and non-governmental organizations at the landscape scale, including efforts to delineate priority watershed networks which, if protected or restored, can most efficiently encompass freshwater biodiversity in the state for multiple species groups.

## Conclusions

Human population growth, increasing demands for freshwater resources, and climate change are projected to exacerbate strains on freshwater resources and lead to further imperilment and extinction of freshwater taxa [1, 8–10, 44–45]. Fundamental to addressing this conservation challenge is information to elucidate what taxa are at risk and where best to focus efforts to improve conservation of freshwater species diversity. This study provides a foundation for freshwater conservation planning in California and highlights key hotspots of freshwater species which serve as priorities for conservation action. Yet, major gaps remain in our understanding of freshwater species distribution and status, as well as in the conservation protections afforded that diversity. Filling these knowledge gaps–e.g., with targeted surveys for understudied taxa, especially the listed, vulnerable, and endemic forms–is essential to inform current and future water management decisions. Addressing the gaps and inadequacies in conservation protections will be critical if we are to reverse the alarming declines of freshwater diversity seen in California as around the world.

## Supporting Information

S1 File Criteria used to define freshwater species by taxonomic group. (DOCX)

S1 Table Summary of stream characteristics for regions. Values from National Hydrography Dataset Plus, version 1 (EPA and USGS).
(DOCX)

S2 Table List of sources for freshwater taxa included in our freshwater species list. (DOCX)

S3 Table List of sources that supplied spatial data for freshwater species occurrence. (DOCX)

S4 Table List of freshwater taxa included in study. (DOCX)
