# Correction: Patterns of Freshwater Species Richness, Endemism, and Vulnerability in California

**DOI:** 10.1371/journal.pone.0158927

**Published:** 2016-07-05

**Authors:** PLOS ONE Staff

Fig 7 is incorrect. Please see the correct Fig 7 here. The publisher apologizes for the error.

**Fig 7:**
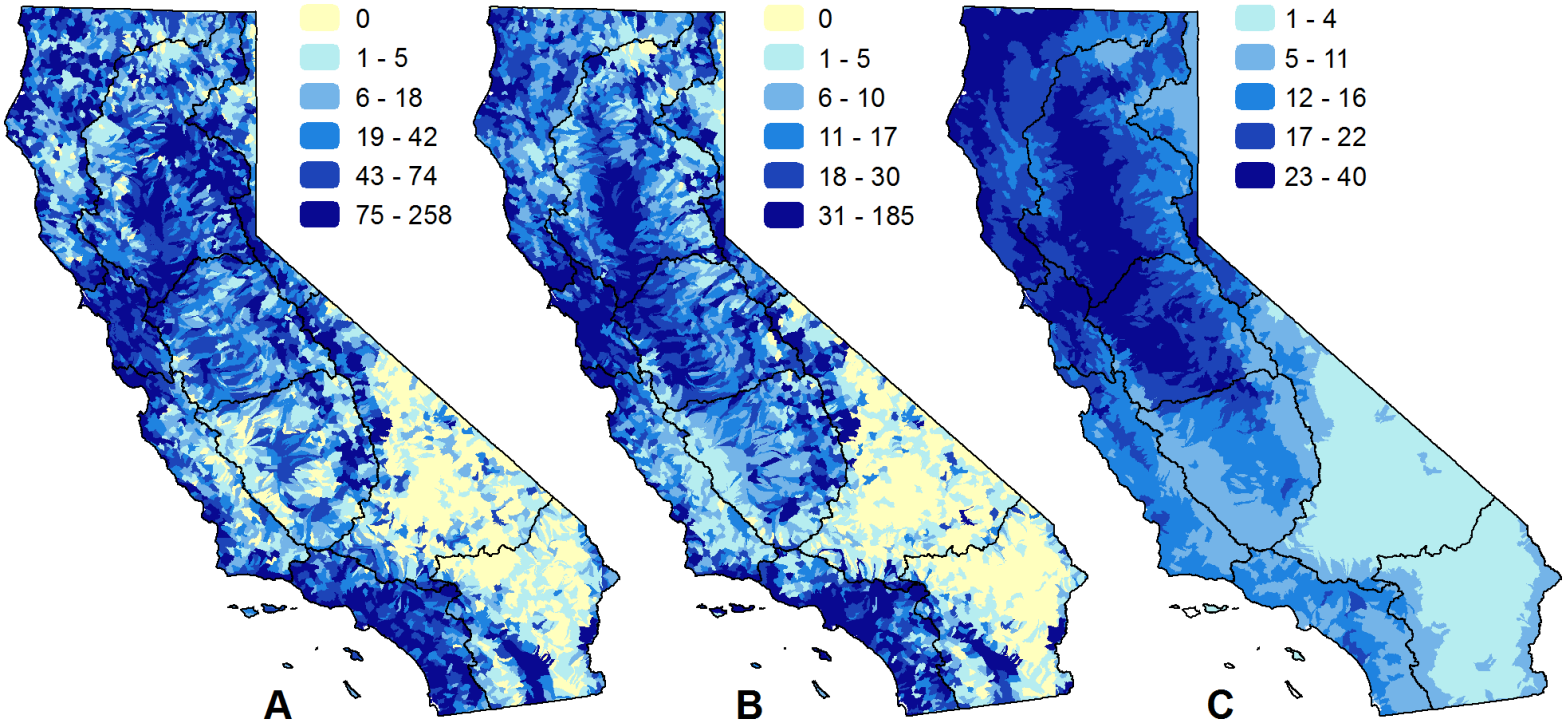
Patterns of richness by data type of California freshwater species. Maps show the number of native freshwater species when summarized by: (A) observational data recorded after 1980; (B) observational data recorded before 1980 or observations of extirpated populations; and (C) data that includes range maps, historical range maps, modeled habitat, professional judgment, critical habitat designations, and management area designations. Spatial data with an unknown observation date or unknown type are not included in any panel. The black lines on the maps represent the major hydrologic regions in the study area.
