# Supplementary material for: Patterns of Freshwater Species Richness, Endemism, and Vulnerability in California
Source: PLoS One. 2015 Jul 6;10(7):e0130710. doi: 10.1371/journal.pone.0130710 (PMC4493109; doi:10.1371/journal.pone.0130710)
Supplement: S1 File — (DOCX) [file pone.0130710.s001.docx]

**S1 File.** Criteria used to define freshwater species

1. FISH

- Freshwater fishes are defined as those that spawn in freshwater. Catadromous species wouldn't qualify, however, we do not have any catadromous species in California. This also precludes several estuarine species commonly found in brackish water such as starry flounder, striped mullet and staghorn sculpin.

1. PLANTS

- Plant species that occur exclusively in freshwater and have special adaptations for living submerged in water, or at the water's surface. Includes free-floating aquatic plans and emergent wetland plants rooted beneath the water surface (e.g., *Nuphar polysepala*).
- Plant species that occur primarily in freshwater wetland habitats but are not strictly aquatic (e.g. *Typha angustifolia*).
- Plant species requiring freshwater inundation to complete their life-cycle, such as plants occurring in long-inundated portions of vernal pools (e.g., *Orcuttia californica*).
- Plant species associated with freshwater and aquatic habitats over much of their range or life-cycle as identified by expert botanists.
- Plant species identified in the Jepson Manual of Vascular Plants of California as associated with wetland habitats such as marshes, lakes, vernal pools, fens, springs, and bogs, and dependent on wetland habitat.
- Plant species identified as Wetland Obligates in the U.S. Army Corps of Engineers list of wetland plant species.
- Plant species identified as Facultative Wetland plants in the U.S. Army Corps of Engineers list of wetland plant species, and identified by expert botanists as dependent on freshwater wetland or aquatic habitats.

1. HERPETOFAUNA

- Species that exclusively rely on freshwater or freshwater-dependent vegetation communities in California in order to complete one or more stages of a reproductive cycle.
- Species that forage within freshwater, either as obligates (e.g., *Actinemys marmorata* and *Thamnophis gigas*), non-obligates (e.g., *T. elegans* and *T. ordinoides*), or as obligates and non-obligates depending on point of ontogeny (i.e., larval and adult amphibian of a single species).
- Relict species occurring within mesic microhabitats within xeric landscapes that would not persist in such regions without freshwater springs, such as *Batrachoseps campi* (a plethodontid salamander that exhibits direct development and does not have a larval stage).
- Species that do not require freshwater for foraging or any part of their reproductive cycle, but are typically found in California occurring within the splash zone of freshwater springs and creeks, such as *Plethodon dunni* (a plethodontid salamander with direct development).

1. BIRDS
2. Criteria for Inclusion

- Species that exclusively rely on freshwater or freshwater-dependent vegetation communities in California, including taxa strongly associated with riparian vegetation.
- Species that breed widely across western North America in freshwater habitats and migrate to California where a substantial portion, but not all, of their wintering habitat consists of freshwater habitats
- Species that use coastal waters during winter and migration but rely completely on freshwater for breeding  in California (e.g, Harlequin Duck, American White Pelican, Western Grebe)
- Species that require freshwater inputs in to saline systems where reductions in freshwater inputs could result in complete habitat loss or substantial changes vegetation and habitat suitability (e.g., species that are only found at the Salton Sea , Saltmarsh Common Yellowthroat).
- Species that winter or breed in both freshwater and saline wetlands, but have large portions of their California population dependent on inland freshwater habitats, including flooded agriculture.

1. Criteria for Exclusion

- Species not dependent on the regular presence of freshwater or freshwater-dependent habitats.
- Species that no longer occur in or are not native to the region.
- Species were omitted if they are rare and do not contribute in a meaningful way to the avifauna of the region. – i.e., primarily lost “vagrants,” even if the occur every year (e.g., Swamp Sparrow, American Redstart).

1. INVERTEBRATES

- Benthic macroinvertebrates (BMIs) are those included on the Southwest Association of Freshwater Invertebrate Taxonomists (SAFIT) Standard Taxonomic Effort (STE) list collected as part of freshwater bioassessment in the southwestern United States. The list contains BMI species known to occur in streams, lakes, or wetlands, including vernal pools, but special emphasis was placed on stream taxa since freshwater bioassessment is most frequently conducted in that habitat type. The list was compiled from published literature sources and from records in the State Water Board’s bioassessment database, the latter being derived from surveys of thousands of stream sites throughout California.
- All species in the SAFIT list are benthic in one or more life stages and utilize freshwater habitats in one or more of the following critical life functions: feeding, mating, egg deposition/development, and larval development to maturity.
- The species list is more comprehensive for some taxonomic groups than others, reflecting the knowledge base and interests of the authors and other taxonomists at California’s Aquatic Bioassessment Lab, availability and regional synoptic coverage of primary taxonomic literature, and likelihood of obtaining properly preserved specimens in typical benthic samples. For example, the list is comprehensive for most aquatic insect groups such as mayflies, stoneflies, dragonflies, caddisflies, beetles, the dipteran suborder Nematocera, etc. The dipteran suborder Brachycera is a notable exception, with most taxa being listed at genus level. The species lists also include surface-dwelling groups like Gerridae (water striders, order Hemiptera) and Gyrinidae (whirligig beetles, order Coleoptera), but exclude taxa associated with riparian zones, shore-dwelling species, and plant tissue inhabitants in taxonomic groups such as Collembola, Staphylinidae, Heteroceridae, Chrysomelidae, Curculionidae, Saldidae, Isopoda and Amphipoda.
- The list is comprehensive for benthic crustaceans except Ostracoda. The list does not include planktonic microcrustacea (Copepoda and Cladocera). No attempt has been made to provide comprehensive species lists for freshwater Annelida (segmented worms) as preservation is typically poor in benthic samples, but generic lists are provided for leeches and polychaetes. Similarly, generic listings are included for Acari (water mites). An extensive taxonomic literature is available for these groups and could support compilation of species lists by appropriate experts in future versions. The list also excludes freshwater parasites such as Branchiura and mermithid Nematoda, the Branchiobdella, which are commensals on crayfish, and the Nematomorpha which are parasitic on terrestrial insects but are found in freshwater for part of their life cycle.
- Phylum Mollusca is variably treated: species lists are generally comprehensive for taxa that occur in larger streams and rivers, despite improper preservation that prevents species-level identifications in typical benthic samples that are collected for bioassessment purposes. Pebblesnails (Families Hydrobiidae and Lithoglyphidae) are a diverse group in springs of the southwestern US, but a species list has not been included.
