# Supplementary material for: Patterns of Freshwater Species Richness, Endemism, and Vulnerability in California
Source: PLoS One. 2015 Jul 6;10(7):e0130710. doi: 10.1371/journal.pone.0130710 (PMC4493109; doi:10.1371/journal.pone.0130710)
Supplement: S1 Table — Values from National Hydrography Dataset Plus, version 1 (EPA and USGS). (DOCX) [file pone.0130710.s002.docx]

**S1 Table.** Summary of stream characteristics for regions. Values from National Hydrography Dataset Plus, version 1 (EPA and USGS).

| Region | Area (km²) | Streams (km) | Ratio of perennial to intermittent stream km | Canals & pipelines (km) | Ave. stream slope (%) | Ave. mean annual flow (m³/sec.) | Ave. annual temp. (°C) | Ave. annual total ppt (cm) | Hydrological connections outside CA | Major features |
| --- | --- | --- | --- | --- | --- | --- | --- | --- | --- | --- |
| Central Coast | 29,313 | 27,830 | 0.15 | 228 | 0.07 | 0.13 | 14.0 | 52 | - | Salinas River |
| Colorado River | 51,431 | 31,668 | 0.04 | 3,859 | 0.04 | 18.20 | 18.8 | 21 | Colorado basin (WY, CO, UT, AZ, NM, NV) | Colorado River, Salton Sea |
| North Coast | 50,662 | 34,915 | 2.14 | 796 | 0.18 | 8.42 | 11.5 | 145 | Klamath basin (OR) | Klamath, Trinity, Mad, Russian rivers |
| North Lahontan | 15,863 | 8,917 | 0.75 | 391 | 0.07 | 0.85 | 6.4 | 74 | Drains to closed basins in NV | Lake Tahoe, terminal basins |
| Sacramento River | 70,684 | 49,773 | 0.72 | 11,306 | 0.06 | 6.23 | 12.2 | 98 | - | Sacramento and Pit rivers, springs |
| San Francisco Bay | 11,718 | 7,984 | 0.58 | 1,531 | 0.04 | 1.61 | 14.7 | 66 | - | San Francisco Bay, vernal pools |
| San Joaquin River | 39,686 | 29,145 | 0.57 | 9,559 | 0.06 | 4.03 | 12.5 | 77 | - | San Joaquin River |
| South Coast | 28,295 | 22,400 | 0.10 | 1,694 | 0.07 | 0.08 | 14.9 | 51 | - | Santa Clara River |
| South Lahontan | 69,063 | 43,867 | 0.07 | 1,179 | 0.06 | 0.41 | 14.1 | 27 | Drains to closed basins in NV | Owens River, isolated springs |
| Tulare Lake | 43,592 | 25,412 | 0.30 | 9,591 | 0.09 | 1.41 | 12.2 | 50 | - | Kern River |
|  |  |  |  |  |  |  |  |  |  |  |
|  | | | | | | | | | | |
