# Supplementary material for: Patterns of Freshwater Species Richness, Endemism, and Vulnerability in California
Source: PLoS One. 2015 Jul 6;10(7):e0130710. doi: 10.1371/journal.pone.0130710 (PMC4493109; doi:10.1371/journal.pone.0130710)
Supplement: S2 Table — (DOCX) [file pone.0130710.s003.docx]

**S2 Table.** List of sources used to compile list of freshwater taxa.
