# Supplementary material for: Patterns of Freshwater Species Richness, Endemism, and Vulnerability in California
Source: PLoS One. 2015 Jul 6;10(7):e0130710. doi: 10.1371/journal.pone.0130710 (PMC4493109; doi:10.1371/journal.pone.0130710)
Supplement: S3 Table — (DOCX) [file pone.0130710.s004.docx]

**S3 Table.** Sources used to compile spatial data occurrences.

| ***Citation*** | ***Weblink*** |
| --- | --- |
| Katz, J, P Moyle, R Peek, N Santos, A Bell, R Quiñones, and J Viers. PISCES database. University of California, Davis. Accessed at http://pisces.ucdavis.edu/ on January 8, 2014. | http://pisces.ucdavis.edu/node |
| Nevada Department of Wildlife. 2012. Nevada Wildlife Action Plan. Reno, NV. Accessed at http://www.ndow.org/uploadedFiles/ndoworg/Content/Nevada_Wildlife/Conservation/2013-NV-WAP-Cover-Page-TOC.pdf in 2014. | http://www.ndow.org/uploadedFiles/ndoworg/Content/Nevada_Wildlife/Conservation/2013-NV-WAP-Cover-Page-TOC.pdf |
| Calflora. 2008. The Calflora Database. Berkeley, CA. Accessed at http://www.calflora.org/ on July 18, 2012. | http://www.calflora.org |
| Eriksen, C. and D. Belk. 1999. Fairy Shrimps of California's Puddles, Pools, and Playas. Mad River Press, Eureka, CA. | http://decapoda.nhm.org/pdfs/2863/2863-001.pdf |
| Western Center for Monitoring & Assessment of Freshwater Ecosystems. 2009. WMC and NAMC Joint Database. Utah State University, Logan, UT. Accessed at http://www.usu.edu/buglab/ in July, 2014. | http://www.usu.edu/buglab/ |
| Arizona Dept. of Game and Fish. 2011. Arizona Natural Heritage Program Heritage Data Management System. Phoenix, AZ. Accessed at http://www.azgfd.gov/w_c/edits/species_concern.shtml in 2014. | http://www.azgfd.gov/w_c/edits/species_concern.shtml |
| Oregon Biodiversity Information Center. 2004. GAP Wildlife Models. Portland State University, Portland, OR. Accessed at http://www.pdx.edu/pnwlamp/wildlife-models in 2012. | http://www.pdx.edu/pnwlamp/wildlife-models |
| Hovingh, P. 2012. Field surveys of Great Basin spring habitats. Direct request to Peter Hovingh, Salt Lake City, UT. | phovingh@xmission.com |
| San Francisco Estuary Institute. 2008. SFEI San Francisco Bay Benthic Data (1992 - 2008). San Francisco, CA. Accessed at http://www.sfei.org in 2012. | http://www.sfei.org/ |
| Graening, G et al. 2012. Unpublished data, database report. The Subterranean Institute (http://www.subinstitute.org/), Citrus Heights, CA. | http://www.subinstitute.org/ |
| NatureServe. 2012. Occurrences approximated from NatureServe descriptions. NatureServe, Arlington, Virginia. Accessed via NatureServe Explorer: An online encyclopedia of life [web application], Version 7.1 at http://www.natureserve.org/explorer on July 16, | http://www.natureserve.org/explorer |
| NatureServe. 2012. Occurrences approximated from NatureServe descriptions. NatureServe, Arlington, Virginia. Accessed via NatureServe Explorer: An online encyclopedia of life [web application], Version 7.1 at http://www.natureserve.org/explorer on July 16, | http://www.natureserve.org/explorer |
| US Fish and Wildlife Service. 2011. Final Critical Habitat. Fort Collins, CO. Accessed at http://crithab.fws.gov/crithab in 2012. | http://crithab.fws.gov/crithab/ |
| Boykin, K, et al. 2007. Predicted animal-habitat distributions and species richness. Chapter 3 in JS Prior-Magee, et al. eds. Southwest Regional Gap Analysis Final Report. US Geological Survey, Moscow, ID. Available at http://swregap.nmsu.edu/. | http://swregap.nmsu.edu/ |
| Bury, R, L Gangle III, and S Litrakis. 2002. Inventory for Amphibians and Reptiles in the NPS Klamath Network. US Geological Survey, Corvallis, OR. Available at http://irmafiles.nps.gov/reference/holding/472918. | http://irmafiles.nps.gov/reference/holding/472918 |
| US Forest Service. 2006. Critical Aquatic Refuges in Sierra Nevada National Forests. US Forest Service Pacific Southwest Region - Remote Sensing Lab, McClellan, CA. Accessed at http://www.fs.usda.gov/detail/r5/landmanagement/gis/?cid=fsbdev3_048320 in | http://www.fs.usda.gov/detail/r5/landmanagement/gis/?cid=fsbdev3_048320 |
| Howard, JK. 2010. Sensitive Freshwater Mussel Surveys in the Pacific Southwest Region: Assessment of Conservation Status ("Mussel Sites 2009 Final"). The Nature Conservancy, San Francisco, CA. | jeanette_howard@tnc.org |
| Howard, JK. 2010. Sensitive Freshwater Mussel Surveys in the Pacific Southwest Region: Assessment of Conservation Status ("Forest Service Mussel Sites 062810v2"). The Nature Conservancy, San Francisco, CA. | jeanette_howard@tnc.org |
| Howard, JK. 2010. Sensitive Freshwater Mussel Surveys in the Pacific Southwest Region: Assessment of Conservation Status ("Mussel Sites Final"). The Nature Conservancy, San Francisco, CA. | jeanette_howard@tnc.org |
| California Department of Fish and Game. 2009. California Wildlife Habitat Relationships System. California Interagency Wildlife Task Group, Sacramento, CA. Accessed at http://www.dfg.ca.gov/biogeodata/cwhr/downloads/GIS/cwhr_gis.xml in 2012 | http://www.dfg.ca.gov/biogeodata/cwhr/downloads/GIS/cwhr_gis.xml |
| California Department of Fish and Game. 2009. Tuolumne Aquatic Resources Relational Inventory. Sacramento, CA. Accessed via Biogeographic Information and Observation System at http://www.dfg.ca.gov/biogeodata/bios/ in 2012. | http://bios.dfg.ca.gov/dataset_index.asp |
| Groff, L. 2010. Herptofauna Surveys, Northern California. Humboldt State University, Arcata, CA. Accessed via Biogeographic Information and Observation System at http://www.dfg.ca.gov/biogeodata/bios/ in 2012. | http://bios.dfg.ca.gov/dataset_index.asp |
| Garrison, BA. 2005. Wildlife Surveys - CDFG Lands, Region 2. CA Department of Fish and Game, Sacramento, CA. Accessed via Biogeographic Information and Observation System at http://www.dfg.ca.gov/biogeodata/bios/ in 2012. | http://bios.dfg.ca.gov/dataset_index.asp |
| Krall, M, C Tennant, and ML Westover. 2010. Mussel Sites, Klamath River - 2010. Whitman College, Walla Walla, WA. Accessed via Biogeographic Information and Observation System at http://www.dfg.ca.gov/biogeodata/bios/ in 2012. | http://bios.dfg.ca.gov/dataset_index.asp |
| Krall, M, C Tennant, and ML Westover. 2007. Mussel Sites, Klamath River - 2007. Whitman College, Walla Walla, WA. Accessed via Biogeographic Information and Observation System at http://www.dfg.ca.gov/biogeodata/bios/ in 2012. | http://bios.dfg.ca.gov/dataset_index.asp |
| Garrison, BA. 2005. Herp Coverboard Sampling - Spears and Didion Ranches. CA Department of Fish and Game, Sacramento, CA. Accessed via Biogeographic Information and Observation System at http://www.dfg.ca.gov/biogeodata/bios/ in 2012. | http://bios.dfg.ca.gov/dataset_index.asp |
| California Department of Fish and Game. 2010. Western Pond Turtle Observations - Region 1. Redding, CA. Accessed via Biogeographic Information and Observation System at http://www.dfg.ca.gov/biogeodata/bios/ in 2012. | http://bios.dfg.ca.gov/dataset_index.asp |
| Spiegelberg, M. 2009. Sensitive Wildlife - Center for Natural Lands Management. Center for Natural Lands Management, Sand Diego, CA. Accessed via Biogeographic Information and Observation System at http://www.dfg.ca.gov/biogeodata/bios/ in 2012. | http://bios.dfg.ca.gov/dataset_index.asp |
| San Diego Dept. of Planning and Land Use. 2005. Species on Multiple Species Conservation Planning preserves. San Diego Department of Planning and Land Use, San Diego, CA. Accessed via Biogeographic Information and Observation System at http://www.dfg.ca | http://bios.dfg.ca.gov/dataset_index.asp |
| Spiegelberg, M. 2007. Sensitive Plants - Center for Natural Lands Management. Center for Natural Lands Management, San Diego, CA. Accessed via Biogeographic Information and Observation System at http://www.dfg.ca.gov/biogeodata/bios/ in 2012. | http://bios.dfg.ca.gov/dataset_index.asp |
| Garrison, BA. 2006. Herp Coverboard Sampling - Spears and Didion Ranches. CA Department of Fish and Game, Sacramento, CA. Accessed via Biogeographic Information and Observation System at http://www.dfg.ca.gov/biogeodata/bios/ in 2012. | http://bios.dfg.ca.gov/dataset_index.asp |
| California State Water Resources Control Board. 2014. Surface Water Ambient Monitoring Program. Sacramento, California. Accessed at the California Environmental Data Exchange Network at http://www.ceden.org on April 10, 2014. | http://www.ceden.org |
| Howard, J. 2014. Compilation of Freshwater Mussel Surveys (Unpublished data). The Nature Conservancy, San Francisco, CA. | jeanette_howard@tnc.org |
| Frest, T. J. and E. J. Johannes. 1995. Interior Columbia Basin mollusk species of special concern. Final report to the Interior Columbia Basin Ecosystem Management Project, Walla Walla, WA. Contract #43-0E00-4-9112. 274 pp. plus appendices. | http://www.icbemp.gov/science/frest_1.pdf |
| Hershler et al. 2007. Extensive diversification of pebblesnails (Lithoglyphidae: Fluminicola) in the upper Sacramento River basin, northwestern USA. Zoological Journal of the Linnean Society 149 (3), 371-422. http://onlinelibrary.wiley.com/doi/10.1111/j | http://onlinelibrary.wiley.com/doi/10.1111/j.1096-3642.2007.00243.x/abstract |
| California Department of Fish and Wildlife. 2014. California Natural Diversity Database, May 2014 Version. Sacramento, California. Accessed at http://www.dfg.ca.gov/biogeodata/cnddb/ in May, 2014. | http://www.dfg.ca.gov/biogeodata/cnddb/ |
| Howard, J. 2014. Freshwater Mussel Range Analysis (Unpublished data). The Nature Conservancy, San Francisco, CA. | jeanette_howard@tnc.org |
| California Academy of Sciences. Herpetology Collection. 2014. Species Records. Accessed via HerpNET data portal at http://www.herpnet.org on May 21, 2014. | http://www.herpnet.org/ |
| California Academy of Sciences. Amphibian Collection. 2014. Species Records. Accessed via HerpNET data portal at http://www.herpnet.org on May 21, 2014. | http://www.herpnet.org/ |
| California Academy of Sciences. Reptile Collection. 2014. Species Records. Accessed via HerpNET data portal at http://www.herpnet.org on May 21, 2014. | http://www.herpnet.org/ |
| Carnegie Museum of Natural History. Herpetology Collection. 2014. Species Records. Accessed via HerpNET data portal at http://www.herpnet.org on May 21, 2014. | http://www.herpnet.org/ |
| Cincinnati Museum Center. Herpetology Vouchers. 2014. Species Records. Accessed via HerpNET data portal at http://www.herpnet.org on May 21, 2014. | http://www.herpnet.org/ |
| Cornell University Museum of Vertebrates. Amphibian Collection. 2014. Species Records. Accessed via HerpNET data portal at http://www.herpnet.org on May 21, 2014. | http://www.herpnet.org/ |
| Cornell University Museum of Vertebrates. Reptile Collection. 2014. Species Records. Accessed via HerpNET data portal at http://www.herpnet.org on May 21, 2014. | http://www.herpnet.org/ |
| University of Kansas. Herpetology Collection. 2014. Species Records. Accessed via HerpNET data portal at http://www.herpnet.org on May 21, 2014. | http://www.herpnet.org/ |
| Natural History Museum of Los Angeles County. Herpetology Collection. 2014. Species Records. Accessed via HerpNET data portal at http://www.herpnet.org on May 21, 2014. | http://www.herpnet.org/ |
| Harvard University Museum of Comparative Zoology. Herpetology Collection. 2014. Species Records. Accessed via HerpNET data portal at http://www.herpnet.org on May 21, 2014. | http://www.herpnet.org/ |
| Museum of Southwestern Biology. Herpetology Collection. 2014. Species Records. Accessed via HerpNET data portal at http://www.herpnet.org on May 21, 2014. | http://www.herpnet.org/ |
| Michigan State University Museum. Herpetology Collection. 2014. Species Records. Accessed via HerpNET data portal at http://www.herpnet.org on May 21, 2014. | http://www.herpnet.org/ |
| University of California Berkeley Museum of Vertebrate Zoology. Herpetology Collection. 2014. Species Records. Accessed via HerpNET data portal at http://www.herpnet.org on May 21, 2014. | http://www.herpnet.org/ |
| University of California Berkeley Museum of Vertebrate Zoology. Hildebrand Collection. 2014. Species Records. Accessed via HerpNET data portal at http://www.herpnet.org on May 21, 2014. | http://www.herpnet.org/ |
| University of California Berkeley Museum of Vertebrate Zoology. Herpetology Observations. 2014. Species Records. Accessed via HerpNET data portal at http://www.herpnet.org on May 21, 2014. | http://www.herpnet.org/ |
| James R. Slater Museum of Natural History. Herpetology Collection. 2014. Species Records. Accessed via HerpNET data portal at http://www.herpnet.org on May 21, 2014. | http://www.herpnet.org/ |
| Royal Ontario Museum. Herpetology Collection. 2014. Species Records. Accessed via HerpNET data portal at http://www.herpnet.org on May 21, 2014. | http://www.herpnet.org/ |
| Santa Barbara Museum of Natural History. Herpetology Collection. 2014. Species Records. Accessed via HerpNET data portal at http://www.herpnet.org on May 21, 2014. | http://www.herpnet.org/ |
| Santa Barbara Museum of Natural History. Osteological Collection. 2014. Species Records. Accessed via HerpNET data portal at http://www.herpnet.org on May 21, 2014. | http://www.herpnet.org/ |
| San Diego Natural History Museum. Herpetology Collection. 2014. Species Records. Accessed via HerpNET data portal at http://www.herpnet.org on May 21, 2014. | http://www.herpnet.org/ |
| Staatliches Museum für Naturkunde Stuttgart. Herpetology Collection. 2014. Species Records. Accessed via HerpNET data portal at http://www.herpnet.org on May 21, 2014. | http://www.herpnet.org/ |
| University of Alberta Museum of Zoology. Herpetology Collection. 2014. Species Records. Accessed via HerpNET data portal at http://www.herpnet.org on May 21, 2014. | http://www.herpnet.org/ |
| University of British Columbia Beaty Biodiversity Museum. Cowan Tetrapod Collection - Herpetology. 2014. Species Records. Accessed via HerpNET data portal at http://www.herpnet.org on May 21, 2014. | http://www.herpnet.org/ |
| University of Colorado Museum of Natural History. Herpetology Collection. 2014. Species Records. Accessed via HerpNET data portal at http://www.herpnet.org on May 21, 2014. | http://www.herpnet.org/ |
| University of Nevada, Reno. Herpetology Collection. 2014. Species Records. Accessed via HerpNET data portal at http://www.herpnet.org on May 21, 2014. | http://www.herpnet.org/ |
| Smithsonian Institution National Museum of Natural History. Amphibian & Reptile Collection. 2014. Species Records. Accessed via HerpNET data portal at http://www.herpnet.org on May 21, 2014. | http://www.herpnet.org/ |
| University of Washington Burke Museum. Herpetology Collection. 2014. Species Records. Accessed via HerpNET data portal at http://www.herpnet.org on May 21, 2014. | http://www.herpnet.org/ |
| Yale University Peabody Museum Vertebrate Zoology Division. Herpetology Collection. 2014. Species Records. Accessed via HerpNET data portal at http://www.herpnet.org on May 21, 2014. | http://www.herpnet.org/ |
| Zoological Institute, Russian Academy of Sciences, St. Petersburg. Amphibian Specimens. 2014. Species Records. Accessed via HerpNET data portal at http://www.herpnet.org on May 21, 2014. | http://www.herpnet.org/ |
| California Academy of Sciences. Entomology Collection. 2014. Species Records. Accessed via CalBug, a collaborative specimen-based database curated by the University of California, Berkeley - Essig Museum at http://calbug.berkeley.edu on June 2, 2014. | http://calbug.berkeley.edu/ |
| University of California, Berkeley - Essig Museum. California Terrestrial Arthropod Database. 2014. Species Records. Accessed via CalBug, a collaborative specimen-based database curated by the University of California, Berkeley - Essig Museum at http:/ | http://calbug.berkeley.edu/ |
| California State Arthropod Collection. 2014. Species Records. Accessed via CalBug, a collaborative specimen-based database curated by the University of California, Berkeley - Essig Museum at http://calbug.berkeley.edu on June 2, 2014. | http://calbug.berkeley.edu/ |
| University of California, Berkeley - Essig Museum. California Terrestrial Arthropod Database. 2014. Species Records. Accessed via CalBug, a collaborative specimen-based database curated by the University of California, Berkeley - Essig Museum at http:/ | http://calbug.berkeley.edu/ |
| Los Angeles County Museum. Entomology Collection. 2014. Species Records. Accessed via CalBug, a collaborative specimen-based database curated by the University of California, Berkeley - Essig Museum at http://calbug.berkeley.edu on June 2, 2014. | http://calbug.berkeley.edu/ |
| Oakland Museum of California. 2014. Species Records. Accessed via CalBug, a collaborative specimen-based database curated by the University of California, Berkeley - Essig Museum at http://calbug.berkeley.edu on June 2, 2014. | http://calbug.berkeley.edu/ |
| Santa Barbara Museum of Natural History. Entomology Collection. 2014. Species Records. Accessed via CalBug, a collaborative specimen-based database curated by the University of California, Berkeley - Essig Museum at http://calbug.berkeley.edu on June 2, | http://calbug.berkeley.edu/ |
| San Diego Natural History Museum. 2014. Species Records. Accessed via CalBug, a collaborative specimen-based database curated by the University of California, Berkeley - Essig Museum at http://calbug.berkeley.edu on June 2, 2014. | http://calbug.berkeley.edu/ |
| University of California, Davis. Bohart Museum. 2014. Species Records. Accessed via CalBug, a collaborative specimen-based database curated by the University of California, Berkeley - Essig Museum at http://calbug.berkeley.edu on June 2, 2014. | http://calbug.berkeley.edu/ |
| University of California, Riverside. Entomology Research Museum. 2014. Species Records. Accessed via CalBug, a collaborative specimen-based database curated by the University of California, Berkeley - Essig Museum at http://calbug.berkeley.edu on June 2, | http://calbug.berkeley.edu/ |
| University of Michigan Museum of Zoology. 2014. Species Records. Accessed via CalBug, a collaborative specimen-based database curated by the University of California, Berkeley - Essig Museum at http://calbug.berkeley.edu on June 2, 2014. | http://calbug.berkeley.edu/ |
| President and Fellows of Harvard College. Herbarium of the Arnold Arboretum. Accessed via Consortium of California Herbaria at http://ucjeps.berkeley.edu/consortium/ on May 30, 2014. | http://ucjeps.berkeley.edu/consortium/ |
| President and Fellows of Harvard College. Oakes Ames Orchid Herbarium. Accessed via Consortium of California Herbaria at http://ucjeps.berkeley.edu/consortium/ on May 30, 2014. | http://ucjeps.berkeley.edu/consortium/ |
| Bureau of Land Management, Arcata Field Office. Herbarium. Accessed via Consortium of California Herbaria at http://ucjeps.berkeley.edu/consortium/ on May 30, 2014. | http://ucjeps.berkeley.edu/consortium/ |
| California Academy of Sciences. Herbarium. Accessed via Consortium of California Herbaria at http://ucjeps.berkeley.edu/consortium/ on May 30, 2014. | http://ucjeps.berkeley.edu/consortium/ |
| California Department of Food and Agriculture. Herbarium. Accessed via Consortium of California Herbaria at http://ucjeps.berkeley.edu/consortium/ on May 30, 2014. | http://ucjeps.berkeley.edu/consortium/ |
| California State University, Chico. Chico State Herbarium. Accessed via Consortium of California Herbaria at http://ucjeps.berkeley.edu/consortium/ on May 30, 2014. | http://ucjeps.berkeley.edu/consortium/ |
| Riverside Metropolitan Museum. The Clark Herbarium. Accessed via Consortium of California Herbaria at http://ucjeps.berkeley.edu/consortium/ on May 30, 2014. | http://ucjeps.berkeley.edu/consortium/ |
| California State University, San Bernardino. Herbarium. Accessed via Consortium of California Herbaria at http://ucjeps.berkeley.edu/consortium/ on May 30, 2014. | http://ucjeps.berkeley.edu/consortium/ |
| California Academy of Sciences. Herbarium. Accessed via Consortium of California Herbaria at http://ucjeps.berkeley.edu/consortium/ on May 30, 2014. | http://ucjeps.berkeley.edu/consortium/ |
| Harvard University. Economic Herbarium of Oakes Ames. Accessed via Consortium of California Herbaria at http://ucjeps.berkeley.edu/consortium/ on May 30, 2014. | http://ucjeps.berkeley.edu/consortium/ |
| Harvard University. Gray Herbarium. Accessed via Consortium of California Herbaria at http://ucjeps.berkeley.edu/consortium/ on May 30, 2014. | http://ucjeps.berkeley.edu/consortium/ |
| Humboldt State University. Herbarium. Accessed via Consortium of California Herbaria at http://ucjeps.berkeley.edu/consortium/ on May 30, 2014. | http://ucjeps.berkeley.edu/consortium/ |
| University of California, Irvine. Herbarium. Accessed via Consortium of California Herbaria at http://ucjeps.berkeley.edu/consortium/ on May 30, 2014. | http://ucjeps.berkeley.edu/consortium/ |
| University of California, Berkeley. Jepson Herbarium. Accessed via Consortium of California Herbaria at http://ucjeps.berkeley.edu/consortium/ on May 30, 2014. | http://ucjeps.berkeley.edu/consortium/ |
| Joshua Tree National Park. Herbarium. Accessed via Consortium of California Herbaria at http://ucjeps.berkeley.edu/consortium/ on May 30, 2014. | http://ucjeps.berkeley.edu/consortium/ |
| Stanford University. Jasper Ridge Biological Preserve Herbarium. Accessed via Consortium of California Herbaria at http://ucjeps.berkeley.edu/consortium/ on May 30, 2014. | http://ucjeps.berkeley.edu/consortium/ |
| University of California, Los Angeles. Herbarium. Accessed via Consortium of California Herbaria at http://ucjeps.berkeley.edu/consortium/ on May 30, 2014. | http://ucjeps.berkeley.edu/consortium/ |
| New York Botanical Garden. Herbarium. Accessed via Consortium of California Herbaria at http://ucjeps.berkeley.edu/consortium/ on May 30, 2014. | http://ucjeps.berkeley.edu/consortium/ |
| California Polytechnic State University, San Luis Obispo. Herbarium. Accessed via Consortium of California Herbaria at http://ucjeps.berkeley.edu/consortium/ on May 30, 2014. | http://ucjeps.berkeley.edu/consortium/ |
| Pacific Grove Museum of Natural History. Herbarium. Accessed via Consortium of California Herbaria at http://ucjeps.berkeley.edu/consortium/ on May 30, 2014. | http://ucjeps.berkeley.edu/consortium/ |
| Pomona College. Herbarium. Accessed via Consortium of California Herbaria at http://ucjeps.berkeley.edu/consortium/ on May 30, 2014. | http://ucjeps.berkeley.edu/consortium/ |
| Rancho Santa Ana Botanic Garden. Herbarium. Accessed via Consortium of California Herbaria at http://ucjeps.berkeley.edu/consortium/ on May 30, 2014. | http://ucjeps.berkeley.edu/consortium/ |
| California State University, Sacramento. Herbarium. Accessed via Consortium of California Herbaria at http://ucjeps.berkeley.edu/consortium/ on May 30, 2014. | http://ucjeps.berkeley.edu/consortium/ |
| Santa Barbara Botanic Garden. Herbarium. Accessed via Consortium of California Herbaria at http://ucjeps.berkeley.edu/consortium/ on May 30, 2014. | http://ucjeps.berkeley.edu/consortium/ |
| San Diego Natural History Museum. Herbarium. Accessed via Consortium of California Herbaria at http://ucjeps.berkeley.edu/consortium/ on May 30, 2014. | http://ucjeps.berkeley.edu/consortium/ |
| Southwest Environmental Information Network. Herbarium. Accessed via Consortium of California Herbaria at http://ucjeps.berkeley.edu/consortium/ on May 30, 2014. | http://ucjeps.berkeley.edu/consortium/ |
| California State University, Northridge. Herbarium. Accessed via Consortium of California Herbaria at http://ucjeps.berkeley.edu/consortium/ on May 30, 2014. | http://ucjeps.berkeley.edu/consortium/ |
| San Jose State University. Herbarium. Accessed via Consortium of California Herbaria at http://ucjeps.berkeley.edu/consortium/ on May 30, 2014. | http://ucjeps.berkeley.edu/consortium/ |
| University of California, Berkeley. University Herbarium. Accessed via Consortium of California Herbaria at http://ucjeps.berkeley.edu/consortium/ on May 30, 2014. | http://ucjeps.berkeley.edu/consortium/ |
| University of California, Davis. Herbarium. Accessed via Consortium of California Herbaria at http://ucjeps.berkeley.edu/consortium/ on May 30, 2014. | http://ucjeps.berkeley.edu/consortium/ |
| University of California, Los Angeles. Herbarium. Accessed via Consortium of California Herbaria at http://ucjeps.berkeley.edu/consortium/ on May 30, 2014. | http://ucjeps.berkeley.edu/consortium/ |
| University of California, Riverside. Herbarium. Accessed via Consortium of California Herbaria at http://ucjeps.berkeley.edu/consortium/ on May 30, 2014. | http://ucjeps.berkeley.edu/consortium/ |
| University of California, Santa Barbara. Herbarium. Accessed via Consortium of California Herbaria at http://ucjeps.berkeley.edu/consortium/ on May 30, 2014. | http://ucjeps.berkeley.edu/consortium/ |
| Victor Valley College. Herbarium. Accessed via Consortium of California Herbaria at http://ucjeps.berkeley.edu/consortium/ on May 30, 2014. | http://ucjeps.berkeley.edu/consortium/ |
| Yosemite National Park. Herbarium. Accessed via Consortium of California Herbaria at http://ucjeps.berkeley.edu/consortium/ on May 30, 2014. | http://ucjeps.berkeley.edu/consortium/ |
| The Aarhus University. Herbarium Database. Accessed via Global Biodiversity Information Facility at http://www.gbif.org/dataset/833db434-f762-11e1-a439-00145eb45e9a on May 14, 2014. | http://www.gbif.org/dataset/833db434-f762-11e1-a439-00145eb45e9a |
| Santa Barbara Museum of Natural History. California Beetle Project. Accessed via Global Biodiversity Information Facility at http://www.gbif.org/dataset/84b130ac-f762-11e1-a439-00145eb45e9a on May 14, 2014. | http://www.gbif.org/dataset/84b130ac-f762-11e1-a439-00145eb45e9a |
| Academy of Natural Sciences. Ocean Biogeographic Information System Mollusc Database. Accessed via Global Biodiversity Information Facility at http://www.gbif.org/dataset/83a09216-f762-11e1-a439-00145eb45e9a on May 14, 2014. | http://www.gbif.org/dataset/83a09216-f762-11e1-a439-00145eb45e9a |
| Academy of Natural Sciences. Malacology Philadelphia. Accessed via Global Biodiversity Information Facility at http://www.gbif.org/dataset/86b50d88-f762-11e1-a439-00145eb45e9a on May 14, 2014. | http://www.gbif.org/dataset/86b50d88-f762-11e1-a439-00145eb45e9a |
| Ohio State University Museum of Biological Diversity. Borror Laboratory of Bioacoustics Birds Collection. Accessed via Global Biodiversity Information Facility at http://www.gbif.org/dataset/85fd399c-f762-11e1-a439-00145eb45e9a on July 22, 2014. | http://www.gbif.org/dataset/85fd399c-f762-11e1-a439-00145eb45e9a |
| Ohio State University Museum of Biological Diversity. Charles A. Triplehorn Insect Collection. Accessed via Global Biodiversity Information Facility at http://www.gbif.org/dataset/84ab7b76-f762-11e1-a439-00145eb45e9a on May 14, 2014. | http://www.gbif.org/dataset/84ab7b76-f762-11e1-a439-00145eb45e9a |
| Ohio State University Museum of Biological Diversity. Borror Laboratory of Bioacoustics Recordings. Accessed via Global Biodiversity Information Facility at http://www.gbif.org/dataset/f11db245-3f9f-4fc6-a0cc-12b4124d081b on May 14, 2014. | http://www.gbif.org/dataset/f11db245-3f9f-4fc6-a0cc-12b4124d081b |
| Ohio State University Museum of Biological Diversity. Charles A. Triplehorn Insect Collection. Accessed via Global Biodiversity Information Facility at http://www.gbif.org/dataset/84ab7b76-f762-11e1-a439-00145eb45e9a on May 14, 2014. | http://www.gbif.org/dataset/84ab7b76-f762-11e1-a439-00145eb45e9a |
| Australian National Herbarium. Accessed via Global Biodiversity Information Facility at http://www.gbif.org/dataset/82cd8df8-f762-11e1-a439-00145eb45e9a on May 14, 2014. | http://www.gbif.org/dataset/82cd8df8-f762-11e1-a439-00145eb45e9a |
| Australian National Herbarium. Accessed via Global Biodiversity Information Facility at http://www.gbif.org/dataset/82cd8df8-f762-11e1-a439-00145eb45e9a on May 14, 2014. | http://www.gbif.org/dataset/82cd8df8-f762-11e1-a439-00145eb45e9a |
| Australian National Herbarium. Accessed via Global Biodiversity Information Facility at http://www.gbif.org/dataset/82cd8df8-f762-11e1-a439-00145eb45e9a on May 14, 2014. | http://www.gbif.org/dataset/82cd8df8-f762-11e1-a439-00145eb45e9a |
| Australian National Herbarium. Accessed via Global Biodiversity Information Facility at http://www.gbif.org/dataset/82cd8df8-f762-11e1-a439-00145eb45e9a on May 14, 2014. | http://www.gbif.org/dataset/82cd8df8-f762-11e1-a439-00145eb45e9a |
| Australian National Herbarium. Accessed via Global Biodiversity Information Facility at http://www.gbif.org/dataset/82cd8df8-f762-11e1-a439-00145eb45e9a on May 14, 2014. | http://www.gbif.org/dataset/82cd8df8-f762-11e1-a439-00145eb45e9a |
| Australian National Herbarium. Accessed via Global Biodiversity Information Facility at http://www.gbif.org/dataset/82cd8df8-f762-11e1-a439-00145eb45e9a on May 14, 2014. | http://www.gbif.org/dataset/82cd8df8-f762-11e1-a439-00145eb45e9a |
| Australian National Herbarium. Accessed via Global Biodiversity Information Facility at http://www.gbif.org/dataset/82cd8df8-f762-11e1-a439-00145eb45e9a on May 14, 2014. | http://www.gbif.org/dataset/82cd8df8-f762-11e1-a439-00145eb45e9a |
| Australian National Herbarium. Accessed via Global Biodiversity Information Facility at http://www.gbif.org/dataset/82cd8df8-f762-11e1-a439-00145eb45e9a on May 14, 2014. | http://www.gbif.org/dataset/82cd8df8-f762-11e1-a439-00145eb45e9a |
| Australian National Herbarium. Accessed via Global Biodiversity Information Facility at http://www.gbif.org/dataset/82cd8df8-f762-11e1-a439-00145eb45e9a on May 14, 2014. | http://www.gbif.org/dataset/82cd8df8-f762-11e1-a439-00145eb45e9a |
| Australian National Herbarium. Accessed via Global Biodiversity Information Facility at http://www.gbif.org/dataset/82cd8df8-f762-11e1-a439-00145eb45e9a on May 14, 2014. | http://www.gbif.org/dataset/82cd8df8-f762-11e1-a439-00145eb45e9a |
| Australian National Herbarium. Accessed via Global Biodiversity Information Facility at http://www.gbif.org/dataset/82cd8df8-f762-11e1-a439-00145eb45e9a on May 14, 2014. | http://www.gbif.org/dataset/82cd8df8-f762-11e1-a439-00145eb45e9a |
| California Academy of Sciences. Botany Collection. Accessed via Global Biodiversity Information Facility at http://www.gbif.org/dataset/f934f8e2-32ca-46a7-b2f8-b032a4740454 on May 14, 2014. | http://www.gbif.org/dataset/f934f8e2-32ca-46a7-b2f8-b032a4740454 |
| Consortium of California Herbaria, California Academy of Sciences. Botany Collection. Accessed via Global Biodiversity Information Facility at http://www.gbif.org/dataset/4fa894f4-b6c6-4ec0-b816-9bb03b3ca106 on May 14, 2014. | http://www.gbif.org/dataset/4fa894f4-b6c6-4ec0-b816-9bb03b3ca106 |
| California Academy of Sciences. Botany Collection. Accessed via Global Biodiversity Information Facility at http://www.gbif.org/dataset/f934f8e2-32ca-46a7-b2f8-b032a4740454 on May 14, 2014. | http://www.gbif.org/dataset/f934f8e2-32ca-46a7-b2f8-b032a4740454 |
| California Academy of Sciences. Herpetology Collection. Accessed via Global Biodiversity Information Facility at http://www.gbif.org/dataset/cece4fc2-1fec-4bb5-a335-7252548e3f0b on May 14, 2014. | http://www.gbif.org/dataset/cece4fc2-1fec-4bb5-a335-7252548e3f0b |
| California Academy of Sciences. Invertebrate Collection. Accessed via Global Biodiversity Information Facility at http://www.gbif.org/dataset/44bcde48-ac71-46f2-bf73-24fc3c008b6c on May 14, 2014. | http://www.gbif.org/dataset/44bcde48-ac71-46f2-bf73-24fc3c008b6c |
| California Academy of Sciences. Amphibian Collection. Accessed via Global Biodiversity Information Facility at http://www.gbif.org/dataset/cece4fc2-1fec-4bb5-a335-7252548e3f0b on May 14, 2014. | http://www.gbif.org/dataset/cece4fc2-1fec-4bb5-a335-7252548e3f0b |
| California Academy of Sciences. Reptile Collection. Accessed via Global Biodiversity Information Facility at http://www.gbif.org/dataset/cece4fc2-1fec-4bb5-a335-7252548e3f0b on May 14, 2014. | http://www.gbif.org/dataset/cece4fc2-1fec-4bb5-a335-7252548e3f0b |
| University of California, Berkeley - Essig Museum. California Terrestrial Arthropod Database. Accessed via Global Biodiversity Information Facility at http://www.gbif.org/dataset/5d283bb6-64dd-4626-8b3b-a4e8db5415c3 on May 14, 2014. | http://www.gbif.org/dataset/5d283bb6-64dd-4626-8b3b-a4e8db5415c3 |
| Cheadle Center for Biodiversity and Ecological Restoration. Herpetology Collection. Accessed via Global Biodiversity Information Facility at http://www.gbif.org/dataset/1050a336-b87a-44b1-b0ec-6fe5fcb3d298 on May 14, 2014. | http://www.gbif.org/dataset/1050a336-b87a-44b1-b0ec-6fe5fcb3d298 |
| Consortium of California Herbaria. California Department of Food and Agriculture. Accessed via Global Biodiversity Information Facility at http://www.gbif.org/dataset/4fa894f4-b6c6-4ec0-b816-9bb03b3ca106 on May 14, 2014. | http://www.gbif.org/dataset/4fa894f4-b6c6-4ec0-b816-9bb03b3ca106 |
| Consortium of California Herbaria. California State University, Chico. Accessed via Global Biodiversity Information Facility at http://www.gbif.org/dataset/4fa894f4-b6c6-4ec0-b816-9bb03b3ca106 on May 14, 2014. | http://www.gbif.org/dataset/4fa894f4-b6c6-4ec0-b816-9bb03b3ca106 |
| University of California, Berkeley - Essig Museum. California Terrestrial Arthropod Database. Accessed via Global Biodiversity Information Facility at http://www.gbif.org/dataset/5d283bb6-64dd-4626-8b3b-a4e8db5415c3 on May 14, 2014. | http://www.gbif.org/dataset/5d283bb6-64dd-4626-8b3b-a4e8db5415c3 |
| Consortium of California Herbaria. Riverside Metropolitan Museum Clark Herbarium. Accessed via Global Biodiversity Information Facility at http://www.gbif.org/dataset/4fa894f4-b6c6-4ec0-b816-9bb03b3ca106 on May 14, 2014. | http://www.gbif.org/dataset/4fa894f4-b6c6-4ec0-b816-9bb03b3ca106 |
| Carnegie Museums. Herpetology Collection. Accessed via Global Biodiversity Information Facility at http://www.gbif.org/dataset/76dd8f0d-2daa-4a69-9fcd-55e04230334a on May 14, 2014. | http://www.gbif.org/dataset/76dd8f0d-2daa-4a69-9fcd-55e04230334a |
| Cincinnati Museum Center. Herpetology Vouchers. Accessed via Global Biodiversity Information Facility at http://www.gbif.org/dataset/81a975b3-d86f-434e-ad9e-16bc43f68a36 on May 14, 2014. | http://www.gbif.org/dataset/81a975b3-d86f-434e-ad9e-16bc43f68a36 |
| Canadian Museum of Nature Herbarium. Vascular Plant Collection. Accessed via Global Biodiversity Information Facility at http://www.gbif.org/dataset/830da118-f762-11e1-a439-00145eb45e9a on May 14, 2014. | http://www.gbif.org/dataset/830da118-f762-11e1-a439-00145eb45e9a |
| Canadian Museum of Nature. Amphibian and Reptile Collection - Anura. Accessed via Global Biodiversity Information Facility at http://www.gbif.org/dataset/830a1f84-f762-11e1-a439-00145eb45e9a on May 14, 2014. | http://www.gbif.org/dataset/830a1f84-f762-11e1-a439-00145eb45e9a |
| Canadian Museum of Nature Mollusc Collection - Unionoida. Accessed via Global Biodiversity Information Facility at http://www.gbif.org/dataset/830c7b08-f762-11e1-a439-00145eb45e9a on May 14, 2014. | http://www.gbif.org/dataset/830c7b08-f762-11e1-a439-00145eb45e9a |
| Consortium of California Herbaria. California Academy of Sciences. Accessed via Global Biodiversity Information Facility at http://www.gbif.org/dataset/0fb2c370-a84f-11de-978d-b8a03c50a862 on May 14, 2014. | http://www.gbif.org/dataset/0fb2c370-a84f-11de-978d-b8a03c50a862 |
| Consortium of California Herbaria. California Department of Food and Agriculture. Accessed via Global Biodiversity Information Facility at http://www.gbif.org/dataset/0fb2c370-a84f-11de-978d-b8a03c50a862 on May 14, 2014. | http://www.gbif.org/dataset/0fb2c370-a84f-11de-978d-b8a03c50a862 |
| Consortium of California Herbaria. California State University, Chico. Accessed via Global Biodiversity Information Facility at http://www.gbif.org/dataset/0fb2c370-a84f-11de-978d-b8a03c50a862 on May 14, 2014. | http://www.gbif.org/dataset/0fb2c370-a84f-11de-978d-b8a03c50a862 |
| Consortium of California Herbaria. California Academy of Sciences. Accessed via Global Biodiversity Information Facility at http://www.gbif.org/dataset/0fb2c370-a84f-11de-978d-b8a03c50a862 on May 14, 2014. | http://www.gbif.org/dataset/0fb2c370-a84f-11de-978d-b8a03c50a862 |
| Consortium of California Herbaria. Humboldt State University. Accessed via Global Biodiversity Information Facility at http://www.gbif.org/dataset/0fb2c370-a84f-11de-978d-b8a03c50a862 on May 14, 2014. | http://www.gbif.org/dataset/0fb2c370-a84f-11de-978d-b8a03c50a862 |
| Consortium of California Herbaria. University of California, Irvine. Accessed via Global Biodiversity Information Facility at http://www.gbif.org/dataset/0fb2c370-a84f-11de-978d-b8a03c50a862 on May 14, 2014. | http://www.gbif.org/dataset/0fb2c370-a84f-11de-978d-b8a03c50a862 |
| Consortium of California Herbaria. University of California, Berkeley Jepson Herbarium. Accessed via Global Biodiversity Information Facility at http://www.gbif.org/dataset/0fb2c370-a84f-11de-978d-b8a03c50a862 on May 14, 2014. | http://www.gbif.org/dataset/0fb2c370-a84f-11de-978d-b8a03c50a862 |
| Consortium of California Herbaria. Pacific Grove Museum of Natural History. Accessed via Global Biodiversity Information Facility at http://www.gbif.org/dataset/0fb2c370-a84f-11de-978d-b8a03c50a862 on May 14, 2014. | http://www.gbif.org/dataset/0fb2c370-a84f-11de-978d-b8a03c50a862 |
| Consortium of California Herbaria. Pomona College Herbaria. Accessed via Global Biodiversity Information Facility at http://www.gbif.org/dataset/0fb2c370-a84f-11de-978d-b8a03c50a862 on May 14, 2014. | http://www.gbif.org/dataset/0fb2c370-a84f-11de-978d-b8a03c50a862 |
| Consortium of California Herbaria. Rancho Santa Ana Botanic Garden Herbaria. Accessed via Global Biodiversity Information Facility at http://www.gbif.org/dataset/0fb2c370-a84f-11de-978d-b8a03c50a862 on May 14, 2014. | http://www.gbif.org/dataset/0fb2c370-a84f-11de-978d-b8a03c50a862 |
| Consortium of California Herbaria. Santa Barbara Botanic Garden. Accessed via Global Biodiversity Information Facility at http://www.gbif.org/dataset/0fb2c370-a84f-11de-978d-b8a03c50a862 on May 14, 2014. | http://www.gbif.org/dataset/0fb2c370-a84f-11de-978d-b8a03c50a862 |
| Consortium of California Herbaria. San Diego Natural History Museum. Accessed via Global Biodiversity Information Facility at http://www.gbif.org/dataset/0fb2c370-a84f-11de-978d-b8a03c50a862 on May 14, 2014. | http://www.gbif.org/dataset/0fb2c370-a84f-11de-978d-b8a03c50a862 |
| Consortium of California Herbaria. San Diego State University. Accessed via Global Biodiversity Information Facility at http://www.gbif.org/dataset/0fb2c370-a84f-11de-978d-b8a03c50a862 on May 14, 2014. | http://www.gbif.org/dataset/0fb2c370-a84f-11de-978d-b8a03c50a862 |
| Consortium of California Herbaria. San Jose State University. Accessed via Global Biodiversity Information Facility at http://www.gbif.org/dataset/0fb2c370-a84f-11de-978d-b8a03c50a862 on May 14, 2014. | http://www.gbif.org/dataset/0fb2c370-a84f-11de-978d-b8a03c50a862 |
| Consortium of California Herbaria. University of California, Berkeley University Herbarium. Accessed via Global Biodiversity Information Facility at http://www.gbif.org/dataset/0fb2c370-a84f-11de-978d-b8a03c50a862 on May 14, 2014. | http://www.gbif.org/dataset/0fb2c370-a84f-11de-978d-b8a03c50a862 |
| Consortium of California Herbaria. University of California, Los Angeles. Accessed via Global Biodiversity Information Facility at http://www.gbif.org/dataset/0fb2c370-a84f-11de-978d-b8a03c50a862 on May 14, 2014. | http://www.gbif.org/dataset/0fb2c370-a84f-11de-978d-b8a03c50a862 |
| Consortium of California Herbaria. University of California, Riverside. Accessed via Global Biodiversity Information Facility at http://www.gbif.org/dataset/0fb2c370-a84f-11de-978d-b8a03c50a862 on May 14, 2014. | http://www.gbif.org/dataset/0fb2c370-a84f-11de-978d-b8a03c50a862 |
| Consortium of California Herbaria. University of California, Santa Barbara. Accessed via Global Biodiversity Information Facility at http://www.gbif.org/dataset/0fb2c370-a84f-11de-978d-b8a03c50a862 on May 14, 2014. | http://www.gbif.org/dataset/0fb2c370-a84f-11de-978d-b8a03c50a862 |
| Consortium of California Herbaria. University of California, Santa Cruz. Accessed via Global Biodiversity Information Facility at http://www.gbif.org/dataset/0fb2c370-a84f-11de-978d-b8a03c50a862 on May 14, 2014. | http://www.gbif.org/dataset/0fb2c370-a84f-11de-978d-b8a03c50a862 |
| University of California, Berkeley - Essig Museum. California Terrestrial Arthropod Database. Accessed via Global Biodiversity Information Facility at http://www.gbif.org/dataset/5d283bb6-64dd-4626-8b3b-a4e8db5415c3 on May 14, 2014. | http://www.gbif.org/dataset/5d283bb6-64dd-4626-8b3b-a4e8db5415c3 |
| Consortium of California Herbaria. California State University, San Bernardino. Accessed via Global Biodiversity Information Facility at http://www.gbif.org/dataset/4fa894f4-b6c6-4ec0-b816-9bb03b3ca106 on May 14, 2014. | http://www.gbif.org/dataset/4fa894f4-b6c6-4ec0-b816-9bb03b3ca106 |
| Cornell Lab of Ornithology. Macaulay Library Audio and Video Collection. Accessed via Global Biodiversity Information Facility at http://www.gbif.org/dataset/7f6dd0f7-9ed4-49c0-bb71-b2a9c7fed9f1 on July 22, 2014. | http://www.gbif.org/dataset/7f6dd0f7-9ed4-49c0-bb71-b2a9c7fed9f1 |
| Cornell University Museum of Vertebrates. Amphibian Collection. Accessed via Global Biodiversity Information Facility at http://www.gbif.org/dataset/a8ee9bc6-5914-427d-9fba-f8545250ac34 on May 14, 2014. | http://www.gbif.org/dataset/a8ee9bc6-5914-427d-9fba-f8545250ac34 |
| Cornell University Museum of Vertebrates. Reptile Collection. Accessed via Global Biodiversity Information Facility at http://www.gbif.org/dataset/b99095f3-d1e9-4902-9938-10ff1711ca5d on May 14, 2014. | http://www.gbif.org/dataset/b99095f3-d1e9-4902-9938-10ff1711ca5d |
| Consortium of California Herbaria. California Academy of Sciences. Accessed via Global Biodiversity Information Facility at http://www.gbif.org/dataset/4fa894f4-b6c6-4ec0-b816-9bb03b3ca106 on May 14, 2014. | http://www.gbif.org/dataset/4fa894f4-b6c6-4ec0-b816-9bb03b3ca106 |
| Royal Botanic Garden Edinburgh. Royal Botanic Garden Edinburgh Living Plant Collections. Accessed via Global Biodiversity Information Facility at http://www.gbif.org/dataset/7adf20e0-c955-11de-95c0-b8a03c50a862 on May 14, 2014. | http://www.gbif.org/dataset/7adf20e0-c955-11de-95c0-b8a03c50a862 |
| Academy of Natural Sciences. Ocean Biogeographic Information System Mollusc Database, 2000. Accessed via Global Biodiversity Information Facility at http://www.gbif.org/dataset/838bb5ee-f762-11e1-a439-00145eb45e9a on May 14, 2014. | http://www.gbif.org/dataset/838bb5ee-f762-11e1-a439-00145eb45e9a |
| Academy of Natural Sciences. Ocean Biogeographic Information System Mollusc Database, 2002. Accessed via Global Biodiversity Information Facility at http://www.gbif.org/dataset/838bb5ee-f762-11e1-a439-00145eb45e9a on May 14, 2014. | http://www.gbif.org/dataset/838bb5ee-f762-11e1-a439-00145eb45e9a |
| Academy of Natural Sciences. Ocean Biogeographic Information System Mollusc Database, 2003. Accessed via Global Biodiversity Information Facility at http://www.gbif.org/dataset/838bb5ee-f762-11e1-a439-00145eb45e9a on May 14, 2014. | http://www.gbif.org/dataset/838bb5ee-f762-11e1-a439-00145eb45e9a |
| Academy of Natural Sciences. Ocean Biogeographic Information System Mollusc Database, 1999. Accessed via Global Biodiversity Information Facility at http://www.gbif.org/dataset/838bb5ee-f762-11e1-a439-00145eb45e9a on May 14, 2014. | http://www.gbif.org/dataset/838bb5ee-f762-11e1-a439-00145eb45e9a |
| Academy of Natural Sciences. Ocean Biogeographic Information System Mollusc Database, 2000. Accessed via Global Biodiversity Information Facility at http://www.gbif.org/dataset/838bb5ee-f762-11e1-a439-00145eb45e9a on May 14, 2014. | http://www.gbif.org/dataset/838bb5ee-f762-11e1-a439-00145eb45e9a |
| Academy of Natural Sciences. Ocean Biogeographic Information System Mollusc Database, 1999. Accessed via Global Biodiversity Information Facility at http://www.gbif.org/dataset/838bb5ee-f762-11e1-a439-00145eb45e9a on May 14, 2014. | http://www.gbif.org/dataset/838bb5ee-f762-11e1-a439-00145eb45e9a |
| University of California, Berkeley - Essig Museum. California Terrestrial Arthropod Database. Accessed via Global Biodiversity Information Facility at http://www.gbif.org/dataset/5d283bb6-64dd-4626-8b3b-a4e8db5415c3 on May 14, 2014. | http://www.gbif.org/dataset/5d283bb6-64dd-4626-8b3b-a4e8db5415c3 |
| Field Museum of Natural History (Botany). Seed Plant Collection. Accessed via Global Biodiversity Information Facility at http://www.gbif.org/dataset/90c853e6-56bd-480b-8e8f-6285c3f8d42b on May 14, 2014. | http://www.gbif.org/dataset/90c853e6-56bd-480b-8e8f-6285c3f8d42b |
| Florida Museum of Natural History. Invertebrate Zoology. Accessed via Global Biodiversity Information Facility at http://www.gbif.org/dataset/85b1cfb6-f762-11e1-a439-00145eb45e9a on May 14, 2014. | http://www.gbif.org/dataset/85b1cfb6-f762-11e1-a439-00145eb45e9a |
| Senckenberg Nature Research Society. Herbarium Senckenbergianum. Accessed via Global Biodiversity Information Facility at http://www.gbif.org/dataset/966426ce-f762-11e1-a439-00145eb45e9a on May 14, 2014. | http://www.gbif.org/dataset/966426ce-f762-11e1-a439-00145eb45e9a |
| Staatliche Naturwissenschaftliche Sammlungen Bayerns. The Fungal Collection at the Senckenberg Museum fÃ¼r Naturkunde GÃ¶rlitz. Accessed via Global Biodiversity Information Facility at http://www.gbif.org/dataset/7a2660bc-f762-11e1-a439-00145eb45e9a on May | http://www.gbif.org/dataset/7a2660bc-f762-11e1-a439-00145eb45e9a |
| Karl Franzens University of Graz. Insitute for Botany - Herbarium. Accessed via Global Biodiversity Information Facility at http://www.gbif.org/dataset/807a0573-87ec-4c1e-a23a-15a327c85dd3 on May 14, 2014. | http://www.gbif.org/dataset/807a0573-87ec-4c1e-a23a-15a327c85dd3 |
| Harvard University Herbaria. Gray Herbarium. Accessed via Global Biodiversity Information Facility at http://www.gbif.org/dataset/861e6afe-f762-11e1-a439-00145eb45e9a on May 14, 2014. | http://www.gbif.org/dataset/861e6afe-f762-11e1-a439-00145eb45e9a |
| University of Arizona Herbarium. Accessed via Global Biodiversity Information Facility at http://www.gbif.org/dataset/95b97882-f762-11e1-a439-00145eb45e9a on May 14, 2014. | http://www.gbif.org/dataset/95b97882-f762-11e1-a439-00145eb45e9a |
| Consortium of California Herbaria. Humboldt State University. Accessed via Global Biodiversity Information Facility at http://www.gbif.org/dataset/4fa894f4-b6c6-4ec0-b816-9bb03b3ca106 on May 14, 2014. | http://www.gbif.org/dataset/4fa894f4-b6c6-4ec0-b816-9bb03b3ca106 |
| iNaturalist.org. Research-Grade Observations. Accessed via Global Biodiversity Information Facility at http://www.gbif.org/dataset/50c9509d-22c7-4a22-a47d-8c48425ef4a7 on May 14, 2014. | http://www.gbif.org/dataset/50c9509d-22c7-4a22-a47d-8c48425ef4a7 |
| Illinois Natural History Survey. Insect Collection. Accessed via Global Biodiversity Information Facility at http://www.gbif.org/dataset/68513375-3aa5-4f6f-9975-d97d56c21d61 on May 14, 2014. | http://www.gbif.org/dataset/68513375-3aa5-4f6f-9975-d97d56c21d61 |
| Consortium of California Herbaria. University of California, Irvine. Accessed via Global Biodiversity Information Facility at http://www.gbif.org/dataset/4fa894f4-b6c6-4ec0-b816-9bb03b3ca106 on May 14, 2014. | http://www.gbif.org/dataset/4fa894f4-b6c6-4ec0-b816-9bb03b3ca106 |
| Consortium of California Herbaria. University of California, Berkeley Jepson Herbarium. Accessed via Global Biodiversity Information Facility at http://www.gbif.org/dataset/4fa894f4-b6c6-4ec0-b816-9bb03b3ca106 on May 14, 2014. | http://www.gbif.org/dataset/4fa894f4-b6c6-4ec0-b816-9bb03b3ca106 |
| Consortium of California Herbaria. Joshua Tree National Park. Accessed via Global Biodiversity Information Facility at http://www.gbif.org/dataset/4fa894f4-b6c6-4ec0-b816-9bb03b3ca106 on May 14, 2014. | http://www.gbif.org/dataset/4fa894f4-b6c6-4ec0-b816-9bb03b3ca106 |
| Consortium of California Herbaria. Jasper Ridge Biological Preserve, Stanford University. Accessed via Global Biodiversity Information Facility at http://www.gbif.org/dataset/4fa894f4-b6c6-4ec0-b816-9bb03b3ca106 on May 14, 2014. | http://www.gbif.org/dataset/4fa894f4-b6c6-4ec0-b816-9bb03b3ca106 |
| Royal Botanic Gardens. Kew Herbarium. Accessed via Global Biodiversity Information Facility at http://www.gbif.org/dataset/84aca1ae-f762-11e1-a439-00145eb45e9a on May 14, 2014. | http://www.gbif.org/dataset/84aca1ae-f762-11e1-a439-00145eb45e9a |
| University of Kansas Biodiversity Institute. R. L. McGregor Herbarium Vascular Plants Collection. Accessed via Global Biodiversity Information Facility at http://www.gbif.org/dataset/95c938a8-f762-11e1-a439-00145eb45e9a on May 14, 2014. | http://www.gbif.org/dataset/95c938a8-f762-11e1-a439-00145eb45e9a |
| University of Kansas Biodiversity Institute. Herpetology Collection. Accessed via Global Biodiversity Information Facility at http://www.gbif.org/dataset/dce00a1f-f6b4-4e11-9771-92c62c40ad80 on May 14, 2014. | http://www.gbif.org/dataset/dce00a1f-f6b4-4e11-9771-92c62c40ad80 |
| University of Kansas Biodiversity Institute. Snow Entomological Museum Collection. Accessed via Global Biodiversity Information Facility at http://www.gbif.org/dataset/aae308f4-9f9c-4cdd-b4ef-c026f48be551 on May 14, 2014. | http://www.gbif.org/dataset/aae308f4-9f9c-4cdd-b4ef-c026f48be551 |
| Naturalis Biodiversity Center. Nationaal Herbarium Nederland. Accessed via Global Biodiversity Information Facility at http://www.gbif.org/dataset/7b33b040-f762-11e1-a439-00145eb45e9a on May 14, 2014. | http://www.gbif.org/dataset/7b33b040-f762-11e1-a439-00145eb45e9a |
| Consortium of California Herbaria. University of California, Los Angeles. Accessed via Global Biodiversity Information Facility at http://www.gbif.org/dataset/4fa894f4-b6c6-4ec0-b816-9bb03b3ca106 on May 14, 2014. | http://www.gbif.org/dataset/4fa894f4-b6c6-4ec0-b816-9bb03b3ca106 |
| Natural History Museum of Los Angeles County. Herpetology Collection. Accessed via Global Biodiversity Information Facility at http://www.gbif.org/dataset/7a25f7aa-03fb-4322-aaeb-66719e1a9527 on May 14, 2014. | http://www.gbif.org/dataset/7a25f7aa-03fb-4322-aaeb-66719e1a9527 |
| University of California, Berkeley - Essig Museum. California Terrestrial Arthropod Database. Accessed via Global Biodiversity Information Facility at http://www.gbif.org/dataset/5d283bb6-64dd-4626-8b3b-a4e8db5415c3 on May 14, 2014. | http://www.gbif.org/dataset/5d283bb6-64dd-4626-8b3b-a4e8db5415c3 |
| Lund Botanical Museum. Accessed via Global Biodiversity Information Facility at http://www.gbif.org/dataset/aab0cf80-0c64-11dd-84d1-b8a03c50a862 on May 14, 2014. | http://www.gbif.org/dataset/aab0cf80-0c64-11dd-84d1-b8a03c50a862 |
| Biologiezentrum Linz Oberoesterreich. Biologiezentrum Linz. Accessed via Global Biodiversity Information Facility at http://www.gbif.org/dataset/857bce66-f762-11e1-a439-00145eb45e9a on May 14, 2014. | http://www.gbif.org/dataset/857bce66-f762-11e1-a439-00145eb45e9a |
| Louisiana State University Herbarium. Accessed via Global Biodiversity Information Facility at http://www.gbif.org/dataset/56e9c560-bd2a-11dd-b15e-b8a03c50a862 on May 14, 2014. | http://www.gbif.org/dataset/56e9c560-bd2a-11dd-b15e-b8a03c50a862 |
| Staatliche Naturwissenschaftliche Sammlungen Bayerns. The Erysiphales Collection at the Botanische Staatssammlung MÃ¼nchen. Accessed via Global Biodiversity Information Facility at http://www.gbif.org/dataset/858d51e0-f762-11e1-a439-00145eb45e9a on May 14, | http://www.gbif.org/dataset/858d51e0-f762-11e1-a439-00145eb45e9a |
| Real Jardin Botanico de Madrid. Algae Collection. Accessed via Global Biodiversity Information Facility at http://www.gbif.org/dataset/834c9918-f762-11e1-a439-00145eb45e9a on May 14, 2014. | http://www.gbif.org/dataset/834c9918-f762-11e1-a439-00145eb45e9a |
| Harvard University Museum of Comparative Zoology. Herpetology Collection. Accessed via Global Biodiversity Information Facility at http://www.gbif.org/dataset/4bfac3ea-8763-4f4b-a71a-76a6f5f243d3 on May 14, 2014. | http://www.gbif.org/dataset/4bfac3ea-8763-4f4b-a71a-76a6f5f243d3 |
| Harvard University Museum of Comparative Zoology. Invertebrate Zoology Collection. Accessed via Global Biodiversity Information Facility at http://www.gbif.org/dataset/4bfac3ea-8763-4f4b-a71a-76a6f5f243d3 on May 14, 2014. | http://www.gbif.org/dataset/4bfac3ea-8763-4f4b-a71a-76a6f5f243d3 |
| Harvard University Museum of Comparative Zoology. Malcology Collection. Accessed via Global Biodiversity Information Facility at http://www.gbif.org/dataset/4bfac3ea-8763-4f4b-a71a-76a6f5f243d3 on May 14, 2014. | http://www.gbif.org/dataset/4bfac3ea-8763-4f4b-a71a-76a6f5f243d3 |
| Santa Barbara Museum of Natural History. California Beetle Project. Accessed via Global Biodiversity Information Facility at http://www.gbif.org/dataset/84b130ac-f762-11e1-a439-00145eb45e9a on May 14, 2014. | http://www.gbif.org/dataset/84b130ac-f762-11e1-a439-00145eb45e9a |
| Sternberg Museum of Natural History. Herpetology Collection. Accessed via Global Biodiversity Information Facility at http://www.gbif.org/dataset/84e823d2-f762-11e1-a439-00145eb45e9a on May 14, 2014. | http://www.gbif.org/dataset/84e823d2-f762-11e1-a439-00145eb45e9a |
| MusÃ©um National d'Histoire Naturelle. Vascular Plants Collection. Accessed via Global Biodiversity Information Facility at http://www.gbif.org/dataset/b5cdf794-8fa4-4a85-8b26-755d087bf531 on May 14, 2014. | http://www.gbif.org/dataset/b5cdf794-8fa4-4a85-8b26-755d087bf531 |
| Missouri Botanical Garden. Tropicos. Accessed via Global Biodiversity Information Facility at http://www.gbif.org/dataset/7bd65a7a-f762-11e1-a439-00145eb45e9a on May 14, 2014. | http://www.gbif.org/dataset/7bd65a7a-f762-11e1-a439-00145eb45e9a |
| Michigan State University Museum. Ichthyology and Herpetology Collections. Accessed via Global Biodiversity Information Facility at http://www.gbif.org/dataset/847bbbde-f762-11e1-a439-00145eb45e9a on May 14, 2014. | http://www.gbif.org/dataset/847bbbde-f762-11e1-a439-00145eb45e9a |
| Museum of Vertebrate Zoology. Herpetology Collection. Accessed via Global Biodiversity Information Facility at http://www.gbif.org/dataset/09c4287e-e6d5-4552-a07f-bff8a00833d8 on May 14, 2014. | http://www.gbif.org/dataset/09c4287e-e6d5-4552-a07f-bff8a00833d8 |
| Museum of Vertebrate Zoology. Hildebrand Collection. Accessed via Global Biodiversity Information Facility at http://www.gbif.org/dataset/423d9318-4dd4-4d31-81cb-27778c44a3bc on May 14, 2014. | http://www.gbif.org/dataset/423d9318-4dd4-4d31-81cb-27778c44a3bc |
| Museum of Vertebrate Zoology. Herpetology Collection. Accessed via Global Biodiversity Information Facility at http://www.gbif.org/dataset/09c4287e-e6d5-4552-a07f-bff8a00833d8 on May 14, 2014. | http://www.gbif.org/dataset/09c4287e-e6d5-4552-a07f-bff8a00833d8 |
| Museum of Vertebrate Zoology. Hildebrand Collection. Accessed via Global Biodiversity Information Facility at http://www.gbif.org/dataset/423d9318-4dd4-4d31-81cb-27778c44a3bc on May 14, 2014. | http://www.gbif.org/dataset/423d9318-4dd4-4d31-81cb-27778c44a3bc |
| Museum of Vertebrate Zoology. Herpetology Observations. Accessed via Global Biodiversity Information Facility at http://www.gbif.org/dataset/f3e4b261-00c5-4f3a-a5b7-d66075b7f3e1 on May 14, 2014. | http://www.gbif.org/dataset/f3e4b261-00c5-4f3a-a5b7-d66075b7f3e1 |
| Museum of Vertebrate Zoology. Herpetology Observations. Accessed via Global Biodiversity Information Facility at http://www.gbif.org/dataset/f3e4b261-00c5-4f3a-a5b7-d66075b7f3e1 on May 14, 2014. | http://www.gbif.org/dataset/f3e4b261-00c5-4f3a-a5b7-d66075b7f3e1 |
| North Carolina Museum of Natural Sciences. Invertebrates Collection. Accessed via Global Biodiversity Information Facility at http://www.gbif.org/dataset/d7ce3688-e91d-4f26-b2bb-333357c6da9f on May 14, 2014. | http://www.gbif.org/dataset/d7ce3688-e91d-4f26-b2bb-333357c6da9f |
| Centre for Genetic Resources, the Netherlands. Plant Genetic Resouces Passport Data. Accessed via Global Biodiversity Information Facility at http://www.gbif.org/dataset/85796928-f762-11e1-a439-00145eb45e9a on May 14, 2014. | http://www.gbif.org/dataset/85796928-f762-11e1-a439-00145eb45e9a |
| Natural History Museum Rotterdam. Insecta collection. Accessed via Global Biodiversity Information Facility at http://www.gbif.org/dataset/d5e61920-9863-4fc3-8e5a-80f0c7bfe640 on May 14, 2014. | http://www.gbif.org/dataset/d5e61920-9863-4fc3-8e5a-80f0c7bfe640 |
| Ocean Biogeographic Information System. San Francisco Bay Data. Accessed via Global Biodiversity Information Facility at http://www.gbif.org/dataset/8399a5be-f762-11e1-a439-00145eb45e9a on May 14, 2014. | http://www.gbif.org/dataset/8399a5be-f762-11e1-a439-00145eb45e9a |
| National Herbarium of New South Wales. Herbarium Collection. Accessed via Global Biodiversity Information Facility at http://www.gbif.org/dataset/853006c0-f762-11e1-a439-00145eb45e9a on May 14, 2014. | http://www.gbif.org/dataset/853006c0-f762-11e1-a439-00145eb45e9a |
| Consortium of California Herbarium. New York Botanical Garden. Accessed via Global Biodiversity Information Facility at http://www.gbif.org/dataset/7133ff0a-f762-11e1-a439-00145eb45e9a on May 14, 2014. | http://www.gbif.org/dataset/7133ff0a-f762-11e1-a439-00145eb45e9a |
| Consortium of California Herbaria. California Polytechnic State University, San Luis Obispo. Accessed via Global Biodiversity Information Facility at http://www.gbif.org/dataset/4fa894f4-b6c6-4ec0-b816-9bb03b3ca106 on May 14, 2014. | http://www.gbif.org/dataset/4fa894f4-b6c6-4ec0-b816-9bb03b3ca106 |
| Ohio State University Museum of Biological Diversity Tetrapod Division. Amphibian Collection. Accessed via Global Biodiversity Information Facility at http://www.gbif.org/dataset/3d84f407-8a76-473a-b8c8-54a58d5f581b on May 14, 2014. | http://www.gbif.org/dataset/3d84f407-8a76-473a-b8c8-54a58d5f581b |
| Ohio State University Museum of Biological Diversity Tetrapod Division. Reptile Collection. Accessed via Global Biodiversity Information Facility at http://www.gbif.org/dataset/51fa0155-a545-4154-ac20-b89dbb2c312b on May 14, 2014. | http://www.gbif.org/dataset/51fa0155-a545-4154-ac20-b89dbb2c312b |
| Ohio State University Museum of Biological Diversity Acarology Laboratory. Mites Collection. Accessed via Global Biodiversity Information Facility at http://www.gbif.org/dataset/96b54e8c-f762-11e1-a439-00145eb45e9a on May 14, 2014. | http://www.gbif.org/dataset/96b54e8c-f762-11e1-a439-00145eb45e9a |
| University of California, Berkeley - Essig Museum. California Terrestrial Arthropod Database. Accessed via Global Biodiversity Information Facility at http://www.gbif.org/dataset/5d283bb6-64dd-4626-8b3b-a4e8db5415c3 on May 14, 2014. | http://www.gbif.org/dataset/5d283bb6-64dd-4626-8b3b-a4e8db5415c3 |
| Sam Noble Oklahoma Museum of Natural History. Amphibian Specimens. Accessed via Global Biodiversity Information Facility at http://www.gbif.org/dataset/2e64dedd-0996-4cd6-b6cd-4f055a46c38c on May 14, 2014. | http://www.gbif.org/dataset/2e64dedd-0996-4cd6-b6cd-4f055a46c38c |
| Sam Noble Oklahoma Museum of Natural History. Reptile Specimens. Accessed via Global Biodiversity Information Facility at http://www.gbif.org/dataset/ad0d4b56-c620-45a5-9152-7a0da3bd48e8 on May 14, 2014. | http://www.gbif.org/dataset/ad0d4b56-c620-45a5-9152-7a0da3bd48e8 |
| Sam Noble Oklahoma Museum of Natural History. Recent Invertebrates Specimens. Accessed via Global Biodiversity Information Facility at http://www.gbif.org/dataset/5378e1cf-522d-4469-8776-b709579b4a3e on May 14, 2014. | http://www.gbif.org/dataset/5378e1cf-522d-4469-8776-b709579b4a3e |
| Ohio State University Museum of Biological Diversity Acarology Laboratory. Mites Collection. Accessed via Global Biodiversity Information Facility at http://www.gbif.org/dataset/96b54e8c-f762-11e1-a439-00145eb45e9a on May 14, 2014. | http://www.gbif.org/dataset/96b54e8c-f762-11e1-a439-00145eb45e9a |
| Oregon State University. Vascular Plant Collection. Accessed via Global Biodiversity Information Facility at http://www.gbif.org/dataset/84aa5ee4-f762-11e1-a439-00145eb45e9a on May 14, 2014. | http://www.gbif.org/dataset/84aa5ee4-f762-11e1-a439-00145eb45e9a |
| Oregon State University. Vascular Plant Collection. Accessed via Global Biodiversity Information Facility at http://www.gbif.org/dataset/84aa5ee4-f762-11e1-a439-00145eb45e9a on May 14, 2014. | http://www.gbif.org/dataset/84aa5ee4-f762-11e1-a439-00145eb45e9a |
| Ohio State University Museum of Biological Diversity Acarology Laboratory. Insect Collection. Accessed via Global Biodiversity Information Facility at http://www.gbif.org/dataset/84ab7b76-f762-11e1-a439-00145eb45e9a on May 14, 2014. | http://www.gbif.org/dataset/84ab7b76-f762-11e1-a439-00145eb45e9a |
| University of California, Santa Barbara Marine Science Institute. Paleobiology Database. Accessed via Global Biodiversity Information Facility at http://www.gbif.org/dataset/84806e86-f762-11e1-a439-00145eb45e9a on May 14, 2014. | http://www.gbif.org/dataset/84806e86-f762-11e1-a439-00145eb45e9a |
| University of California, Santa Barbara Marine Science Institute. Paleobiology Database. Accessed via Global Biodiversity Information Facility at http://www.gbif.org/dataset/84806e86-f762-11e1-a439-00145eb45e9a on May 14, 2014. | http://www.gbif.org/dataset/84806e86-f762-11e1-a439-00145eb45e9a |
| University of California, Santa Barbara Marine Science Institute. Paleobiology Database. Accessed via Global Biodiversity Information Facility at http://www.gbif.org/dataset/84806e86-f762-11e1-a439-00145eb45e9a on May 14, 2014. | http://www.gbif.org/dataset/84806e86-f762-11e1-a439-00145eb45e9a |
| University of California, Santa Barbara Marine Science Institute. Paleobiology Database. Accessed via Global Biodiversity Information Facility at http://www.gbif.org/dataset/84806e86-f762-11e1-a439-00145eb45e9a on May 14, 2014. | http://www.gbif.org/dataset/84806e86-f762-11e1-a439-00145eb45e9a |
| University of California, Santa Barbara Marine Science Institute. Paleobiology Database. Accessed via Global Biodiversity Information Facility at http://www.gbif.org/dataset/84806e86-f762-11e1-a439-00145eb45e9a on May 14, 2014. | http://www.gbif.org/dataset/84806e86-f762-11e1-a439-00145eb45e9a |
| University of California, Santa Barbara Marine Science Institute. Paleobiology Database. Accessed via Global Biodiversity Information Facility at http://www.gbif.org/dataset/84806e86-f762-11e1-a439-00145eb45e9a on May 14, 2014. | http://www.gbif.org/dataset/84806e86-f762-11e1-a439-00145eb45e9a |
| University of California, Santa Barbara Marine Science Institute. Paleobiology Database. Accessed via Global Biodiversity Information Facility at http://www.gbif.org/dataset/84806e86-f762-11e1-a439-00145eb45e9a on May 14, 2014. | http://www.gbif.org/dataset/84806e86-f762-11e1-a439-00145eb45e9a |
| University of California, Santa Barbara Marine Science Institute. Paleobiology Database. Accessed via Global Biodiversity Information Facility at http://www.gbif.org/dataset/84806e86-f762-11e1-a439-00145eb45e9a on May 14, 2014. | http://www.gbif.org/dataset/84806e86-f762-11e1-a439-00145eb45e9a |
| University of California, Santa Barbara Marine Science Institute. Paleobiology Database. Accessed via Global Biodiversity Information Facility at http://www.gbif.org/dataset/84806e86-f762-11e1-a439-00145eb45e9a on May 14, 2014. | http://www.gbif.org/dataset/84806e86-f762-11e1-a439-00145eb45e9a |
| Consortium of California Herbarium. Pacific Grove Museum of Natural History. Accessed via Global Biodiversity Information Facility at http://www.gbif.org/dataset/4fa894f4-b6c6-4ec0-b816-9bb03b3ca106 on May 14, 2014. | http://www.gbif.org/dataset/4fa894f4-b6c6-4ec0-b816-9bb03b3ca106 |
| Consortium of California Herbarium. Pomona College Herbarium. Accessed via Global Biodiversity Information Facility at http://www.gbif.org/dataset/4fa894f4-b6c6-4ec0-b816-9bb03b3ca106 on May 14, 2014. | http://www.gbif.org/dataset/4fa894f4-b6c6-4ec0-b816-9bb03b3ca106 |
| James R. Slater Museum of Natural History. Herpetology Collection. Accessed via Global Biodiversity Information Facility at http://www.gbif.org/dataset/8eddc200-f535-4c65-9b4d-f723eafe607e on May 14, 2014. | http://www.gbif.org/dataset/8eddc200-f535-4c65-9b4d-f723eafe607e |
| McGill University Redpath Museum. Herpetological specimens. Accessed via Global Biodiversity Information Facility at http://www.gbif.org/dataset/7132ed22-f762-11e1-a439-00145eb45e9a on May 14, 2014. | http://www.gbif.org/dataset/7132ed22-f762-11e1-a439-00145eb45e9a |
| Royal Ontario Museum. Herpetology Collection. Accessed via Global Biodiversity Information Facility at http://www.gbif.org/dataset/84bd4658-f762-11e1-a439-00145eb45e9a on May 14, 2014. | http://www.gbif.org/dataset/84bd4658-f762-11e1-a439-00145eb45e9a |
| Royal Belgian Institute of Natural Sciences. Amphibian Collection. Accessed via Global Biodiversity Information Facility at http://www.gbif.org/dataset/8138eb72-f762-11e1-a439-00145eb45e9a on May 14, 2014. | http://www.gbif.org/dataset/8138eb72-f762-11e1-a439-00145eb45e9a |
| Royal Belgian Institute of Natural Sciences. Accessed via Global Biodiversity Information Facility at http://www.gbif.org/dataset/8138eb72-f762-11e1-a439-00145eb45e9a on May 14, 2014. | http://www.gbif.org/dataset/8138eb72-f762-11e1-a439-00145eb45e9a |
| Royal Ontario Museum. Herpetology Collection. Accessed via Global Biodiversity Information Facility at http://www.gbif.org/dataset/8c201186-d997-4b65-aac9-2fcf442a93f6 on May 14, 2014. | http://www.gbif.org/dataset/8c201186-d997-4b65-aac9-2fcf442a93f6 |
| Consortium of California Herbarium. Rancho Santa Ana Botanic Garden Herbiarum. Accessed via Global Biodiversity Information Facility at http://www.gbif.org/dataset/4fa894f4-b6c6-4ec0-b816-9bb03b3ca106 on May 14, 2014. | http://www.gbif.org/dataset/4fa894f4-b6c6-4ec0-b816-9bb03b3ca106 |
| Consortium of California Herbaria. California State University, Sacramento. Accessed via Global Biodiversity Information Facility at http://www.gbif.org/dataset/4fa894f4-b6c6-4ec0-b816-9bb03b3ca106 on May 14, 2014. | http://www.gbif.org/dataset/4fa894f4-b6c6-4ec0-b816-9bb03b3ca106 |
| Consortium of California Herbaria. Santa Barbara Botanic Garden. Accessed via Global Biodiversity Information Facility at http://www.gbif.org/dataset/4fa894f4-b6c6-4ec0-b816-9bb03b3ca106 on May 14, 2014. | http://www.gbif.org/dataset/4fa894f4-b6c6-4ec0-b816-9bb03b3ca106 |
| Santa Barbara Museum of Natural History. Entomology Collection. Accessed via Global Biodiversity Information Facility at http://www.gbif.org/dataset/84b130ac-f762-11e1-a439-00145eb45e9a on May 14, 2014. | http://www.gbif.org/dataset/84b130ac-f762-11e1-a439-00145eb45e9a |
| Santa Barbara Museum of Natural History. Herpetology Collection. Accessed via Global Biodiversity Information Facility at http://www.gbif.org/dataset/75018539-6328-41de-b875-7c2e61dc1635 on May 14, 2014. | http://www.gbif.org/dataset/75018539-6328-41de-b875-7c2e61dc1635 |
| Santa Barbara Museum of Natural History. Osteological Collection. Accessed via Global Biodiversity Information Facility at http://www.gbif.org/dataset/75018539-6328-41de-b875-7c2e61dc1635 on May 14, 2014. | http://www.gbif.org/dataset/75018539-6328-41de-b875-7c2e61dc1635 |
| Santa Barbara Museum of Natural History. Entomology Collection. Accessed via Global Biodiversity Information Facility at http://www.gbif.org/dataset/84b130ac-f762-11e1-a439-00145eb45e9a on May 14, 2014. | http://www.gbif.org/dataset/84b130ac-f762-11e1-a439-00145eb45e9a |
| Santa Barbara Museum of Natural History. Arthropods Collection. Accessed via Global Biodiversity Information Facility at http://www.gbif.org/dataset/5d283bb6-64dd-4626-8b3b-a4e8db5415c3 on May 14, 2014. | http://www.gbif.org/dataset/5d283bb6-64dd-4626-8b3b-a4e8db5415c3 |
| Consortium of California Herbaria. San Diego Natural History Museum. Accessed via Global Biodiversity Information Facility at http://www.gbif.org/dataset/4fa894f4-b6c6-4ec0-b816-9bb03b3ca106 on May 14, 2014. | http://www.gbif.org/dataset/4fa894f4-b6c6-4ec0-b816-9bb03b3ca106 |
| University of California, Berkeley - Essig Museum. California Terrestrial Arthropod Database. Accessed via Global Biodiversity Information Facility at http://www.gbif.org/dataset/5d283bb6-64dd-4626-8b3b-a4e8db5415c3 on May 14, 2014. | http://www.gbif.org/dataset/5d283bb6-64dd-4626-8b3b-a4e8db5415c3 |
| San Diego Natural History Museum. Herpetological specimens. Accessed via Global Biodiversity Information Facility at http://www.gbif.org/dataset/84b4d6e4-f762-11e1-a439-00145eb45e9a on May 14, 2014. | http://www.gbif.org/dataset/84b4d6e4-f762-11e1-a439-00145eb45e9a |
| Consortium of California Herbaria. San Diego State University. Accessed via Global Biodiversity Information Facility at http://www.gbif.org/dataset/4fa894f4-b6c6-4ec0-b816-9bb03b3ca106 on May 14, 2014. | http://www.gbif.org/dataset/4fa894f4-b6c6-4ec0-b816-9bb03b3ca106 |
| Consortium of California Herbaria. California State University, Northridge. Accessed via Global Biodiversity Information Facility at http://www.gbif.org/dataset/4fa894f4-b6c6-4ec0-b816-9bb03b3ca106 on May 14, 2014. | http://www.gbif.org/dataset/4fa894f4-b6c6-4ec0-b816-9bb03b3ca106 |
| Consortium of California Herbaria. San Jose State University. Accessed via Global Biodiversity Information Facility at http://www.gbif.org/dataset/4fa894f4-b6c6-4ec0-b816-9bb03b3ca106 on May 14, 2014. | http://www.gbif.org/dataset/4fa894f4-b6c6-4ec0-b816-9bb03b3ca106 |
| Senckenberg Nature Research Society. Crustacean Collection. Accessed via Global Biodiversity Information Facility at http://www.gbif.org/dataset/9668b676-f762-11e1-a439-00145eb45e9a on May 14, 2014. | http://www.gbif.org/dataset/9668b676-f762-11e1-a439-00145eb45e9a |
| Staatliche Naturwissenschaftliche Sammlungen Bayerns. Fungus Collections. Accessed via Global Biodiversity Information Facility at http://www.gbif.org/dataset/61a9ca38-b62f-11e2-afcb-00145eb45e9a on May 14, 2014. | http://www.gbif.org/dataset/61a9ca38-b62f-11e2-afcb-00145eb45e9a |
| Staatliches Museum für Naturkunde Stuttgart. Herpetology Collection. Accessed via Global Biodiversity Information Facility at http://www.gbif.org/dataset/9cd0014c-b7b1-4ed1-bef7-0225acfa4ef2 on May 14, 2014. | http://www.gbif.org/dataset/9cd0014c-b7b1-4ed1-bef7-0225acfa4ef2 |
| Texas A&M University. Insect Collection. Accessed via Global Biodiversity Information Facility at http://www.gbif.org/dataset/96193ea2-f762-11e1-a439-00145eb45e9a on May 14, 2014. | http://www.gbif.org/dataset/96193ea2-f762-11e1-a439-00145eb45e9a |
| The University of Texas at Austin - Texas Natural History Collections. Herpetology Collection. Accessed via Global Biodiversity Information Facility at http://www.gbif.org/dataset/852628c6-f762-11e1-a439-00145eb45e9a on May 14, 2014. | http://www.gbif.org/dataset/852628c6-f762-11e1-a439-00145eb45e9a |
| University of Alberta Museums. Vascular Plant Herbarium. Accessed via Global Biodiversity Information Facility at http://www.gbif.org/dataset/7b3e4870-f762-11e1-a439-00145eb45e9a on May 14, 2014. | http://www.gbif.org/dataset/7b3e4870-f762-11e1-a439-00145eb45e9a |
| University of Alberta Museums. Amphibian and Reptile Collection. Accessed via Global Biodiversity Information Facility at http://www.gbif.org/dataset/88d7437e-f762-11e1-a439-00145eb45e9a on May 14, 2014. | http://www.gbif.org/dataset/88d7437e-f762-11e1-a439-00145eb45e9a |
| University of Alabama Biodiversity and Systematics. Herbarium. Accessed via Global Biodiversity Information Facility at http://www.gbif.org/dataset/84f9770e-f762-11e1-a439-00145eb45e9a on May 14, 2014. | http://www.gbif.org/dataset/84f9770e-f762-11e1-a439-00145eb45e9a |
| University of British Columbia. Cowan Tetrapod Collection - Herpetology. Accessed via Global Biodiversity Information Facility at http://www.gbif.org/dataset/df9c8b86-9d36-4e29-91b3-4274dff053e5 on May 14, 2014. | http://www.gbif.org/dataset/df9c8b86-9d36-4e29-91b3-4274dff053e5 |
| Consortium of California Herbaria. University of California, Berkeley - University Herbarium. Accessed via Global Biodiversity Information Facility at http://www.gbif.org/dataset/4fa894f4-b6c6-4ec0-b816-9bb03b3ca106 on May 14, 2014. | http://www.gbif.org/dataset/4fa894f4-b6c6-4ec0-b816-9bb03b3ca106 |
| University of California, Berkeley Natural History Museums. TAPIR Provider. Accessed via Global Biodiversity Information Facility at http://www.gbif.org/dataset/51b92d4e-556f-4a05-bc5c-bfe982ee1156 on May 14, 2014. | http://www.gbif.org/dataset/51b92d4e-556f-4a05-bc5c-bfe982ee1156 |
| University of California, Berkeley Natural History Museums. TAPIR Provider. Accessed via Global Biodiversity Information Facility at http://www.gbif.org/dataset/51b92d4e-556f-4a05-bc5c-bfe982ee1156 on May 14, 2014. | http://www.gbif.org/dataset/51b92d4e-556f-4a05-bc5c-bfe982ee1156 |
| University of California, Berkeley Natural History Museums. Mather Redwood Grove. Accessed via Global Biodiversity Information Facility at http://www.gbif.org/dataset/51b92d4e-556f-4a05-bc5c-bfe982ee1156 on May 14, 2014. | http://www.gbif.org/dataset/51b92d4e-556f-4a05-bc5c-bfe982ee1156 |
| University of California, Berkeley Natural History Museums. Unspecified. Accessed via Global Biodiversity Information Facility at http://www.gbif.org/dataset/51b92d4e-556f-4a05-bc5c-bfe982ee1156 on May 14, 2014. | http://www.gbif.org/dataset/51b92d4e-556f-4a05-bc5c-bfe982ee1156 |
| University of California, Berkeley - Essig Museum. California Terrestrial Arthropod Database. Accessed via Global Biodiversity Information Facility at http://www.gbif.org/dataset/5d283bb6-64dd-4626-8b3b-a4e8db5415c3 on May 14, 2014. | http://www.gbif.org/dataset/5d283bb6-64dd-4626-8b3b-a4e8db5415c3 |
| Consortium of California Herbaria. University of California, Davis. Accessed via Global Biodiversity Information Facility at http://www.gbif.org/dataset/4fa894f4-b6c6-4ec0-b816-9bb03b3ca106 on May 14, 2014. | http://www.gbif.org/dataset/4fa894f4-b6c6-4ec0-b816-9bb03b3ca106 |
| Consortium of California Herbaria. University of California, Berkeley - Jepson Herbarium. Accessed via Global Biodiversity Information Facility at http://www.gbif.org/dataset/5729fd1d-04af-46bd-9da7-0ff79977c6f8 on May 14, 2014. | http://www.gbif.org/dataset/5729fd1d-04af-46bd-9da7-0ff79977c6f8 |
| Consortium of California Herbaria. University of California, Berkeley - University Herbarium. Accessed via Global Biodiversity Information Facility at http://www.gbif.org/dataset/5729fd1d-04af-46bd-9da7-0ff79977c6f8 on May 14, 2014. | http://www.gbif.org/dataset/5729fd1d-04af-46bd-9da7-0ff79977c6f8 |
| Consortium of California Herbaria. University of California, Los Angeles. Accessed via Global Biodiversity Information Facility at http://www.gbif.org/dataset/4fa894f4-b6c6-4ec0-b816-9bb03b3ca106 on May 14, 2014. | http://www.gbif.org/dataset/4fa894f4-b6c6-4ec0-b816-9bb03b3ca106 |
| University of Colorado Museum of Natural History. Amphibian and Reptile Collection. Accessed via Global Biodiversity Information Facility at http://www.gbif.org/dataset/8935e64a-f762-11e1-a439-00145eb45e9a on May 14, 2014. | http://www.gbif.org/dataset/8935e64a-f762-11e1-a439-00145eb45e9a |
| University of Connecticut. George Safford Torrey Herbarium. Accessed via Global Biodiversity Information Facility at http://www.gbif.org/dataset/5288946d-5fcf-4b53-8fd3-74f4cc6b53fc on May 14, 2014. | http://www.gbif.org/dataset/5288946d-5fcf-4b53-8fd3-74f4cc6b53fc |
| Consortium of California Herbaria. University of California, Riverside. Accessed via Global Biodiversity Information Facility at http://www.gbif.org/dataset/4fa894f4-b6c6-4ec0-b816-9bb03b3ca106 on May 14, 2014. | http://www.gbif.org/dataset/4fa894f4-b6c6-4ec0-b816-9bb03b3ca106 |
| University of California, Berkeley - Essig Museum. California Terrestrial Arthropod Database. Accessed via Global Biodiversity Information Facility at http://www.gbif.org/dataset/5d283bb6-64dd-4626-8b3b-a4e8db5415c3 on May 14, 2014. | http://www.gbif.org/dataset/5d283bb6-64dd-4626-8b3b-a4e8db5415c3 |
| Consortium of California Herbaria. University of California, Santa Barbara. Accessed via Global Biodiversity Information Facility at http://www.gbif.org/dataset/4fa894f4-b6c6-4ec0-b816-9bb03b3ca106 on May 14, 2014. | http://www.gbif.org/dataset/4fa894f4-b6c6-4ec0-b816-9bb03b3ca106 |
| Consortium of California Herbaria. University of California, Santa Cruz. Accessed via Global Biodiversity Information Facility at http://www.gbif.org/dataset/4fa894f4-b6c6-4ec0-b816-9bb03b3ca106 on May 14, 2014. | http://www.gbif.org/dataset/4fa894f4-b6c6-4ec0-b816-9bb03b3ca106 |
| University of California, Berkeley - Essig Museum. California Terrestrial Arthropod Database. Accessed via Global Biodiversity Information Facility at http://www.gbif.org/dataset/5d283bb6-64dd-4626-8b3b-a4e8db5415c3 on May 14, 2014. | http://www.gbif.org/dataset/5d283bb6-64dd-4626-8b3b-a4e8db5415c3 |
| University of Alberta Museums. Vascular Plant Herbarium. Accessed via Global Biodiversity Information Facility at http://www.gbif.org/dataset/2429287b-ef65-4cfd-afcc-11cc3ba95cca on May 14, 2014. | http://www.gbif.org/dataset/2429287b-ef65-4cfd-afcc-11cc3ba95cca |
| University of Alberta Museums. Entomology Collection. Accessed via Global Biodiversity Information Facility at http://www.gbif.org/dataset/8971dfba-f762-11e1-a439-00145eb45e9a on May 14, 2014. | http://www.gbif.org/dataset/8971dfba-f762-11e1-a439-00145eb45e9a |
| University of British Columbia Herbarium. Accessed via Global Biodiversity Information Facility at http://www.gbif.org/dataset/07fd0d79-4883-435f-bba1-58fef110cd13 on May 14, 2014. | http://www.gbif.org/dataset/07fd0d79-4883-435f-bba1-58fef110cd13 |
| University of Nevada, Reno. Herpetology Collection. Accessed via Global Biodiversity Information Facility at http://www.gbif.org/dataset/c62f7574-d65a-4018-87a2-b96d6df5231b on May 14, 2014. | http://www.gbif.org/dataset/c62f7574-d65a-4018-87a2-b96d6df5231b |
| University of Puerto Rico. Invertebrate Collection. Accessed via Global Biodiversity Information Facility at http://www.gbif.org/dataset/1162234d-4e06-4d63-8a49-034184a38c7e on May 14, 2014. | http://www.gbif.org/dataset/1162234d-4e06-4d63-8a49-034184a38c7e |
| Smithsonian Institution National Museum of Natural History. Botany Collection. Accessed via Global Biodiversity Information Facility at http://www.gbif.org/dataset/5df38344-b821-49c2-8174-cf0f29f4df0d on May 14, 2014. | http://www.gbif.org/dataset/5df38344-b821-49c2-8174-cf0f29f4df0d |
| United States National Plant Germplasm System. USA151 Collection. Accessed via Global Biodiversity Information Facility at http://www.gbif.org/dataset/85802736-f762-11e1-a439-00145eb45e9a on May 14, 2014. | http://www.gbif.org/dataset/85802736-f762-11e1-a439-00145eb45e9a |
| United States National Plant Germplasm System. USA955 Collection. Accessed via Global Biodiversity Information Facility at http://www.gbif.org/dataset/85802736-f762-11e1-a439-00145eb45e9a on May 14, 2014. | http://www.gbif.org/dataset/85802736-f762-11e1-a439-00145eb45e9a |
| United Stated Geological Survey. Nonindigenous Aquatic Species. Accessed via Global Biodiversity Information Facility at http://www.gbif.org/dataset/d6cc311c-c5ab-4f23-9a20-10514f9eb9c4 on May 14, 2014. | http://www.gbif.org/dataset/d6cc311c-c5ab-4f23-9a20-10514f9eb9c4 |
| Smithsonian Institution National Museum of Natural History. Amphibian & Reptile Collection. Accessed via Global Biodiversity Information Facility at http://www.gbif.org/dataset/5df38344-b821-49c2-8174-cf0f29f4df0d on May 14, 2014. | http://www.gbif.org/dataset/5df38344-b821-49c2-8174-cf0f29f4df0d |
| Smithsonian Institution National Museum of Natural History. Entomology Collection. Accessed via Global Biodiversity Information Facility at http://www.gbif.org/dataset/5df38344-b821-49c2-8174-cf0f29f4df0d on May 14, 2014. | http://www.gbif.org/dataset/5df38344-b821-49c2-8174-cf0f29f4df0d |
| Smithsonian Institution National Museum of Natural History. Invertebrate Zoology Collection. Accessed via Global Biodiversity Information Facility at http://www.gbif.org/dataset/5df38344-b821-49c2-8174-cf0f29f4df0d on May 14, 2014. | http://www.gbif.org/dataset/5df38344-b821-49c2-8174-cf0f29f4df0d |
| Utah State University. Specimen Database. Accessed via Global Biodiversity Information Facility at http://www.gbif.org/dataset/85ac3c18-f762-11e1-a439-00145eb45e9a on May 14, 2014. | http://www.gbif.org/dataset/85ac3c18-f762-11e1-a439-00145eb45e9a |
| University of Texas at El Paso. Herpetology Collection. Accessed via Global Biodiversity Information Facility at http://www.gbif.org/dataset/bd2feca8-ec39-4480-9dad-e353ab6a506d on May 14, 2014. | http://www.gbif.org/dataset/bd2feca8-ec39-4480-9dad-e353ab6a506d |
| Utah Valley University. Utah Valley State College Herbarium. Accessed via Global Biodiversity Information Facility at http://www.gbif.org/dataset/854a88d8-f762-11e1-a439-00145eb45e9a on May 14, 2014. | http://www.gbif.org/dataset/854a88d8-f762-11e1-a439-00145eb45e9a |
| University of Washington Burke Museum. Herpetology Collection. Accessed via Global Biodiversity Information Facility at http://www.gbif.org/dataset/78122332-6315-41bd-914b-e9c1342d9093 on May 14, 2014. | http://www.gbif.org/dataset/78122332-6315-41bd-914b-e9c1342d9093 |
| University of Washington Burke Museum. Plant Collection. Accessed via Global Biodiversity Information Facility at http://www.gbif.org/dataset/8310f570-f762-11e1-a439-00145eb45e9a on May 14, 2014. | http://www.gbif.org/dataset/8310f570-f762-11e1-a439-00145eb45e9a |
| University of Washington Burke Museum. Plant Collection. Accessed via Global Biodiversity Information Facility at http://www.gbif.org/dataset/8310f570-f762-11e1-a439-00145eb45e9a on May 14, 2014. | http://www.gbif.org/dataset/8310f570-f762-11e1-a439-00145eb45e9a |
| Consortium of California Herbaria. Victor Valley College. Accessed via Global Biodiversity Information Facility at http://www.gbif.org/dataset/4fa894f4-b6c6-4ec0-b816-9bb03b3ca106 on May 14, 2014. | http://www.gbif.org/dataset/4fa894f4-b6c6-4ec0-b816-9bb03b3ca106 |
| Vienna Natural History Museum. Herbarium. Accessed via Global Biodiversity Information Facility at http://www.gbif.org/dataset/7f5260c2-f762-11e1-a439-00145eb45e9a on May 14, 2014. | http://www.gbif.org/dataset/7f5260c2-f762-11e1-a439-00145eb45e9a |
| Consortium of California Herbaria. Yosemite National Park Herbarium. Accessed via Global Biodiversity Information Facility at http://www.gbif.org/dataset/4fa894f4-b6c6-4ec0-b816-9bb03b3ca106 on May 14, 2014. | http://www.gbif.org/dataset/4fa894f4-b6c6-4ec0-b816-9bb03b3ca106 |
| Yale University Peabody Museum. Entomology Division. Accessed via Global Biodiversity Information Facility at http://www.gbif.org/dataset/96404cc2-f762-11e1-a439-00145eb45e9a on May 14, 2014. | http://www.gbif.org/dataset/96404cc2-f762-11e1-a439-00145eb45e9a |
| Yale University Peabody Museum. Vertebrate Zoology Division - Herpetology. Accessed via Global Biodiversity Information Facility at http://www.gbif.org/dataset/861d3d64-f762-11e1-a439-00145eb45e9a on May 14, 2014. | http://www.gbif.org/dataset/861d3d64-f762-11e1-a439-00145eb45e9a |
| Yale University Peabody Museum. Vertebrate Zoology Division - Invertebrate Zoology. Accessed via Global Biodiversity Information Facility at http://www.gbif.org/dataset/854e35e6-f762-11e1-a439-00145eb45e9a on May 14, 2014. | http://www.gbif.org/dataset/854e35e6-f762-11e1-a439-00145eb45e9a |
| Zoological Institute, Russian Academy of Sciences, St. Petersburg. Amphibian Specimens. Accessed via Global Biodiversity Information Facility at http://www.gbif.org/dataset/7e34ea34-f762-11e1-a439-00145eb45e9a on May 14, 2014. | http://www.gbif.org/dataset/7e34ea34-f762-11e1-a439-00145eb45e9a |
| Senckenberg Nature Research Society: Crustacean Collection. Accessed via Global Biodiversity Information Facility at http://www.gbif.org/dataset/7b84c0a2-f762-11e1-a439-00145eb45e9a on May 14, 2014. | http://www.gbif.org/dataset/7b84c0a2-f762-11e1-a439-00145eb45e9a |
| Sada, D. 2003. Desert Research Institute Springs Database (http://www.dri.edu/directory/4934-don-sada). Reno, NV. | http://www.dri.edu/directory/4934-don-sada |
| R. Hershler, H Liu, and C Bradford. 2013. Systematics of a widely distributed western North American springsnail Pyrgulopsis micrococcus (Caenogastropoda, Hydrobiidae), with description of three new congeners. Zookeys 330: 27-52 (http://zookeys.pensoft | http://zookeys.pensoft.net/articles.php?id=3635 |
| Museum für Naturkunde Berlin. Anymals+plants - Citizen Science Data - User 13. Accessed via Global Biodiversity Information Facility at http://www.gbif.org/dataset/e6c97f6e-e952-11e2-961f-00145eb45e9a on May 14, 2014. | http://www.gbif.org/dataset/e6c97f6e-e952-11e2-961f-00145eb45e9a |
| California Academy of Sciences. Ornithology Collection. Accessed via Global Biodiversity Information Facility at http://www.gbif.org/dataset/4f29b6ab-20c0-4479-8795-4915bedcebd1 on July 22, 2014. | http://www.gbif.org/dataset/4f29b6ab-20c0-4479-8795-4915bedcebd1 |
| Cheadle Center for Biodiversity and Ecological Restoration. Ornithology Collection. Accessed via Global Biodiversity Information Facility at http://www.gbif.org/dataset/4ada1c77-3895-47d8-8dc9-9ce44e1df802 on July 22, 2014. | http://www.gbif.org/dataset/4ada1c77-3895-47d8-8dc9-9ce44e1df802 |
| Cornell Lab of Ornithology. eBird Observation Dataset, 'EBIRD' Collection. Accessed via Global Biodiversity Information Facility at http://www.gbif.org/dataset/4fa7b334-ce0d-4e88-aaae-2e0c138d049e on July 22, 2014. | http://www.gbif.org/dataset/4fa7b334-ce0d-4e88-aaae-2e0c138d049e |
| Cornell Lab of Ornithology. eBird Observation Dataset, 'EBIRD_AK' Collection. Accessed via Global Biodiversity Information Facility at http://www.gbif.org/dataset/4fa7b334-ce0d-4e88-aaae-2e0c138d049e on July 22, 2014. | http://www.gbif.org/dataset/4fa7b334-ce0d-4e88-aaae-2e0c138d049e |
| Cornell Lab of Ornithology. eBird Observation Dataset, 'EBIRD_BCN' Collection. Accessed via Global Biodiversity Information Facility at http://www.gbif.org/dataset/4fa7b334-ce0d-4e88-aaae-2e0c138d049e on July 22, 2014. | http://www.gbif.org/dataset/4fa7b334-ce0d-4e88-aaae-2e0c138d049e |
| Cornell Lab of Ornithology. eBird Observation Dataset, 'EBIRD_CA' Collection. Accessed via Global Biodiversity Information Facility at http://www.gbif.org/dataset/4fa7b334-ce0d-4e88-aaae-2e0c138d049e on July 22, 2014. | http://www.gbif.org/dataset/4fa7b334-ce0d-4e88-aaae-2e0c138d049e |
| Cornell Lab of Ornithology. eBird Observation Dataset, 'EBIRD_CAN' Collection. Accessed via Global Biodiversity Information Facility at http://www.gbif.org/dataset/4fa7b334-ce0d-4e88-aaae-2e0c138d049e on July 22, 2014. | http://www.gbif.org/dataset/4fa7b334-ce0d-4e88-aaae-2e0c138d049e |
| Cornell Lab of Ornithology. eBird Observation Dataset, 'EBIRD_CB' Collection. Accessed via Global Biodiversity Information Facility at http://www.gbif.org/dataset/4fa7b334-ce0d-4e88-aaae-2e0c138d049e on July 22, 2014. | http://www.gbif.org/dataset/4fa7b334-ce0d-4e88-aaae-2e0c138d049e |
| Cornell Lab of Ornithology. eBird Observation Dataset, 'EBIRD_CBW' Collection. Accessed via Global Biodiversity Information Facility at http://www.gbif.org/dataset/4fa7b334-ce0d-4e88-aaae-2e0c138d049e on July 22, 2014. | http://www.gbif.org/dataset/4fa7b334-ce0d-4e88-aaae-2e0c138d049e |
| Cornell Lab of Ornithology. eBird Observation Dataset, 'EBIRD_CL' Collection. Accessed via Global Biodiversity Information Facility at http://www.gbif.org/dataset/4fa7b334-ce0d-4e88-aaae-2e0c138d049e on July 22, 2014. | http://www.gbif.org/dataset/4fa7b334-ce0d-4e88-aaae-2e0c138d049e |
| Cornell Lab of Ornithology. eBird Observation Dataset, 'EBIRD_CR' Collection. Accessed via Global Biodiversity Information Facility at http://www.gbif.org/dataset/4fa7b334-ce0d-4e88-aaae-2e0c138d049e on July 22, 2014. | http://www.gbif.org/dataset/4fa7b334-ce0d-4e88-aaae-2e0c138d049e |
| Cornell Lab of Ornithology. eBird Observation Dataset, 'EBIRD_ISS' Collection. Accessed via Global Biodiversity Information Facility at http://www.gbif.org/dataset/4fa7b334-ce0d-4e88-aaae-2e0c138d049e on July 22, 2014. | http://www.gbif.org/dataset/4fa7b334-ce0d-4e88-aaae-2e0c138d049e |
| Cornell Lab of Ornithology. eBird Observation Dataset, 'EBIRD_KLAM_SISK' Collection. Accessed via Global Biodiversity Information Facility at http://www.gbif.org/dataset/4fa7b334-ce0d-4e88-aaae-2e0c138d049e on July 22, 2014. | http://www.gbif.org/dataset/4fa7b334-ce0d-4e88-aaae-2e0c138d049e |
| Cornell Lab of Ornithology. eBird Observation Dataset, 'EBIRD_LWBA' Collection. Accessed via Global Biodiversity Information Facility at http://www.gbif.org/dataset/4fa7b334-ce0d-4e88-aaae-2e0c138d049e on July 22, 2014. | http://www.gbif.org/dataset/4fa7b334-ce0d-4e88-aaae-2e0c138d049e |
| Cornell Lab of Ornithology. eBird Observation Dataset, 'EBIRD_MA' Collection. Accessed via Global Biodiversity Information Facility at http://www.gbif.org/dataset/4fa7b334-ce0d-4e88-aaae-2e0c138d049e on July 22, 2014. | http://www.gbif.org/dataset/4fa7b334-ce0d-4e88-aaae-2e0c138d049e |
| Cornell Lab of Ornithology. eBird Observation Dataset, 'EBIRD_MEX' Collection. Accessed via Global Biodiversity Information Facility at http://www.gbif.org/dataset/4fa7b334-ce0d-4e88-aaae-2e0c138d049e on July 22, 2014. | http://www.gbif.org/dataset/4fa7b334-ce0d-4e88-aaae-2e0c138d049e |
| Cornell Lab of Ornithology. eBird Observation Dataset, 'EBIRD_NH' Collection. Accessed via Global Biodiversity Information Facility at http://www.gbif.org/dataset/4fa7b334-ce0d-4e88-aaae-2e0c138d049e on July 22, 2014. | http://www.gbif.org/dataset/4fa7b334-ce0d-4e88-aaae-2e0c138d049e |
| Cornell Lab of Ornithology. eBird Observation Dataset, 'EBIRD_NJ' Collection. Accessed via Global Biodiversity Information Facility at http://www.gbif.org/dataset/4fa7b334-ce0d-4e88-aaae-2e0c138d049e on July 22, 2014. | http://www.gbif.org/dataset/4fa7b334-ce0d-4e88-aaae-2e0c138d049e |
| Cornell Lab of Ornithology. eBird Observation Dataset, 'EBIRD_NY' Collection. Accessed via Global Biodiversity Information Facility at http://www.gbif.org/dataset/4fa7b334-ce0d-4e88-aaae-2e0c138d049e on July 22, 2014. | http://www.gbif.org/dataset/4fa7b334-ce0d-4e88-aaae-2e0c138d049e |
| Cornell Lab of Ornithology. eBird Observation Dataset, 'EBIRD_NZ' Collection. Accessed via Global Biodiversity Information Facility at http://www.gbif.org/dataset/4fa7b334-ce0d-4e88-aaae-2e0c138d049e on July 22, 2014. | http://www.gbif.org/dataset/4fa7b334-ce0d-4e88-aaae-2e0c138d049e |
| Cornell Lab of Ornithology. eBird Observation Dataset, 'EBIRD_PA' Collection. Accessed via Global Biodiversity Information Facility at http://www.gbif.org/dataset/4fa7b334-ce0d-4e88-aaae-2e0c138d049e on July 22, 2014. | http://www.gbif.org/dataset/4fa7b334-ce0d-4e88-aaae-2e0c138d049e |
| Cornell Lab of Ornithology. eBird Observation Dataset, 'EBIRD_PAN' Collection. Accessed via Global Biodiversity Information Facility at http://www.gbif.org/dataset/4fa7b334-ce0d-4e88-aaae-2e0c138d049e on July 22, 2014. | http://www.gbif.org/dataset/4fa7b334-ce0d-4e88-aaae-2e0c138d049e |
| Cornell Lab of Ornithology. eBird Observation Dataset, 'EBIRD_TX' Collection. Accessed via Global Biodiversity Information Facility at http://www.gbif.org/dataset/4fa7b334-ce0d-4e88-aaae-2e0c138d049e on July 22, 2014. | http://www.gbif.org/dataset/4fa7b334-ce0d-4e88-aaae-2e0c138d049e |
| Cornell Lab of Ornithology. eBird Observation Dataset, 'EBIRD_VA' Collection. Accessed via Global Biodiversity Information Facility at http://www.gbif.org/dataset/4fa7b334-ce0d-4e88-aaae-2e0c138d049e on July 22, 2014. | http://www.gbif.org/dataset/4fa7b334-ce0d-4e88-aaae-2e0c138d049e |
| Cornell Lab of Ornithology. eBird Observation Dataset, 'EBIRD_VINS' Collection. Accessed via Global Biodiversity Information Facility at http://www.gbif.org/dataset/4fa7b334-ce0d-4e88-aaae-2e0c138d049e on July 22, 2014. | http://www.gbif.org/dataset/4fa7b334-ce0d-4e88-aaae-2e0c138d049e |
| Cornell Lab of Ornithology. eBird Observation Dataset, 'EBIRD_WI' Collection. Accessed via Global Biodiversity Information Facility at http://www.gbif.org/dataset/4fa7b334-ce0d-4e88-aaae-2e0c138d049e on July 22, 2014. | http://www.gbif.org/dataset/4fa7b334-ce0d-4e88-aaae-2e0c138d049e |
| Cornell Lab of Ornithology. eBird Observation Dataset, 'EBIRD_YARD' Collection. Accessed via Global Biodiversity Information Facility at http://www.gbif.org/dataset/4fa7b334-ce0d-4e88-aaae-2e0c138d049e on July 22, 2014. | http://www.gbif.org/dataset/4fa7b334-ce0d-4e88-aaae-2e0c138d049e |
| Avian Knowledge Network. Great Backyard Bird Count. Accessed via Global Biodiversity Information Facility at http://www.gbif.org/dataset/82cb293c-f762-11e1-a439-00145eb45e9a on July 22, 2014. | http://www.gbif.org/dataset/82cb293c-f762-11e1-a439-00145eb45e9a |
| Canadian Museum of Nature. Bird Collection. Accessed via Global Biodiversity Information Facility at http://www.gbif.org/dataset/8309005e-f762-11e1-a439-00145eb45e9a on July 22, 2014. | http://www.gbif.org/dataset/8309005e-f762-11e1-a439-00145eb45e9a |
| Cornell University Museum of Vertebrates. Bird Collection. Accessed via Global Biodiversity Information Facility at http://www.gbif.org/dataset/f96a6f8c-b992-4159-8039-db8f30bac985 on July 22, 2014. | http://www.gbif.org/dataset/f96a6f8c-b992-4159-8039-db8f30bac985 |
| Delaware Museum of Natural History. Bird Collection. Accessed via Global Biodiversity Information Facility at http://www.gbif.org/dataset/c21cd435-718a-4069-b503-776bf0e22b96 on July 22, 2014. | http://www.gbif.org/dataset/c21cd435-718a-4069-b503-776bf0e22b96 |
| Denver Museum of Nature & Science. Bird Collection. Accessed via Global Biodiversity Information Facility at http://www.gbif.org/dataset/2f54cb88-4167-499a-81fb-0a2d02465212 on July 22, 2014. | http://www.gbif.org/dataset/2f54cb88-4167-499a-81fb-0a2d02465212 |
| Denver Museum of Nature & Science. Bird Collection. Accessed via Global Biodiversity Information Facility at http://www.gbif.org/dataset/2f54cb88-4167-499a-81fb-0a2d02465212 on July 22, 2014. | http://www.gbif.org/dataset/2f54cb88-4167-499a-81fb-0a2d02465212 |
| Universidad Nacional Autónoma de México. Museo de Zoologí­a "Alfonso L. Herrera", Facultad de Ciencias. Accessed via Global Biodiversity Information Facility at http://www.gbif.org/dataset/890c34ee-f762-11e1-a439-00145eb45e9a on May 14, 2014. | http://www.gbif.org/dataset/890c34ee-f762-11e1-a439-00145eb45e9a |
| Humboldt State University. Wildlife Birds Collection. Accessed via Global Biodiversity Information Facility at http://www.gbif.org/dataset/9c007868-b667-4c07-9a1a-96b796066f64 on July 22, 2014. | http://www.gbif.org/dataset/9c007868-b667-4c07-9a1a-96b796066f64 |
| Natural History Museum of Los Angeles County. Birds Collection. Accessed via Global Biodiversity Information Facility at http://www.gbif.org/dataset/7a25f7aa-03fb-4322-aaeb-66719e1a9527 on July 22, 2014. | http://www.gbif.org/dataset/7a25f7aa-03fb-4322-aaeb-66719e1a9527 |
| Harvard University Museum of Comparative Zoology. Bird Collection. Accessed via Global Biodiversity Information Facility at http://www.gbif.org/dataset/4bfac3ea-8763-4f4b-a71a-76a6f5f243d3 on July 22, 2014. | http://www.gbif.org/dataset/4bfac3ea-8763-4f4b-a71a-76a6f5f243d3 |
| Harvard University Museum of Comparative Zoology. Ornithology Collection. Accessed via Global Biodiversity Information Facility at http://www.gbif.org/dataset/4bfac3ea-8763-4f4b-a71a-76a6f5f243d3 on July 22, 2014. | http://www.gbif.org/dataset/4bfac3ea-8763-4f4b-a71a-76a6f5f243d3 |
| Museum of Southwestern Biology. Bird Collection. Accessed via Global Biodiversity Information Facility at http://www.gbif.org/dataset/c9076cd3-349f-4068-a5c7-bc34449c3916 on July 22, 2014. | http://www.gbif.org/dataset/c9076cd3-349f-4068-a5c7-bc34449c3916 |
| Museum of Southwestern Biology. Division of Parasitology. Accessed via Global Biodiversity Information Facility at http://www.gbif.org/dataset/b211f32f-326b-43d3-8012-2fbce0cc6dcc on May 14, 2014. | http://www.gbif.org/dataset/b211f32f-326b-43d3-8012-2fbce0cc6dcc |
| Museum of Southwestern Biology. Bird Collection. Accessed via Global Biodiversity Information Facility at http://www.gbif.org/dataset/c9076cd3-349f-4068-a5c7-bc34449c3916 on July 22, 2014. | http://www.gbif.org/dataset/c9076cd3-349f-4068-a5c7-bc34449c3916 |
| Museum of Southwestern Biology. Division of Parasitology. Accessed via Global Biodiversity Information Facility at http://www.gbif.org/dataset/b211f32f-326b-43d3-8012-2fbce0cc6dcc on May 14, 2014. | http://www.gbif.org/dataset/b211f32f-326b-43d3-8012-2fbce0cc6dcc |
| Museum of Vertebrate Zoology. Egg and Nest Collection. Accessed via Global Biodiversity Information Facility at http://www.gbif.org/dataset/9ce52ff6-01b6-44a2-b617-9bc2ee8e8cd1 on July 22, 2014. | http://www.gbif.org/dataset/9ce52ff6-01b6-44a2-b617-9bc2ee8e8cd1 |
| Museum of Vertebrate Zoology. Bird Collection. Accessed via Global Biodiversity Information Facility at http://www.gbif.org/dataset/e3b959d6-fcbe-4a28-a166-e4a807c340a0 on July 22, 2014. | http://www.gbif.org/dataset/e3b959d6-fcbe-4a28-a166-e4a807c340a0 |
| Museum of Vertebrate Zoology. Egg and Nest Collection. Accessed via Global Biodiversity Information Facility at http://www.gbif.org/dataset/9ce52ff6-01b6-44a2-b617-9bc2ee8e8cd1 on July 22, 2014. | http://www.gbif.org/dataset/9ce52ff6-01b6-44a2-b617-9bc2ee8e8cd1 |
| Museum of Vertebrate Zoology. Bird Observations. Accessed via Global Biodiversity Information Facility at http://www.gbif.org/dataset/62ad511d-d298-4fd7-80e7-f5d5bd32299e on July 22, 2014. | http://www.gbif.org/dataset/62ad511d-d298-4fd7-80e7-f5d5bd32299e |
| Museum of Vertebrate Zoology. Bird Observations. Accessed via Global Biodiversity Information Facility at http://www.gbif.org/dataset/62ad511d-d298-4fd7-80e7-f5d5bd32299e on July 22, 2014. | http://www.gbif.org/dataset/62ad511d-d298-4fd7-80e7-f5d5bd32299e |
| Naturgucker.de / enjoynature.net. Citizen Science Observations. Accessed via Global Biodiversity Information Facility at http://www.gbif.org/dataset/6ac3f774-d9fb-4796-b3e9-92bf6c81c084 on May 14, 2014. | http://www.gbif.org/dataset/6ac3f774-d9fb-4796-b3e9-92bf6c81c084 |
| New Brunswick Museum. Bird Collection. Accessed via Global Biodiversity Information Facility at http://www.gbif.org/dataset/84a80b12-f762-11e1-a439-00145eb45e9a on July 22, 2014. | http://www.gbif.org/dataset/84a80b12-f762-11e1-a439-00145eb45e9a |
| Ocean Biogeographic Information System. Spatial Ecological Analysis of Megavertabrate Populations 41. Accessed via Global Biodiversity Information Facility at http://www.gbif.org/dataset/83a1a8c2-f762-11e1-a439-00145eb45e9a on May 14, 2014. | http://www.gbif.org/dataset/83a1a8c2-f762-11e1-a439-00145eb45e9a |
| Ocean Biogeographic Information System. Spatial Ecological Analysis of Megavertabrate Populations 47. Accessed via Global Biodiversity Information Facility at http://www.gbif.org/dataset/83a1a8c2-f762-11e1-a439-00145eb45e9a on May 14, 2014. | http://www.gbif.org/dataset/83a1a8c2-f762-11e1-a439-00145eb45e9a |
| Ocean Biogeographic Information System. Spatial Ecological Analysis of Megavertabrate Populations 48. Accessed via Global Biodiversity Information Facility at http://www.gbif.org/dataset/83a1a8c2-f762-11e1-a439-00145eb45e9a on May 14, 2014. | http://www.gbif.org/dataset/83a1a8c2-f762-11e1-a439-00145eb45e9a |
| Ohio State University Museum of Biological Diversity Tetrapod Division. Bird Collection. Accessed via Global Biodiversity Information Facility at http://www.gbif.org/dataset/91aa5e23-9cad-4751-86e0-241da77d7407 on July 22, 2014. | http://www.gbif.org/dataset/91aa5e23-9cad-4751-86e0-241da77d7407 |
| Sam Noble Oklahoma Museum of Natural History. Birds Specimens. Accessed via Global Biodiversity Information Facility at http://www.gbif.org/dataset/84b018de-f762-11e1-a439-00145eb45e9a on May 14, 2014. | http://www.gbif.org/dataset/84b018de-f762-11e1-a439-00145eb45e9a |
| Sam Noble Oklahoma Museum of Natural History. Eggs Specimen. Accessed via Global Biodiversity Information Facility at http://www.gbif.org/dataset/39f021d5-240c-445d-b62f-33bfed94938d on May 14, 2014. | http://www.gbif.org/dataset/39f021d5-240c-445d-b62f-33bfed94938d |
| University of California, Santa Barbara Marine Science Institute. Paleobiology Database. Accessed via Global Biodiversity Information Facility at http://www.gbif.org/dataset/84806e86-f762-11e1-a439-00145eb45e9a on May 14, 2014. | http://www.gbif.org/dataset/84806e86-f762-11e1-a439-00145eb45e9a |
| University of California, Santa Barbara Marine Science Institute. Paleobiology Database. Accessed via Global Biodiversity Information Facility at http://www.gbif.org/dataset/84806e86-f762-11e1-a439-00145eb45e9a on May 14, 2014. | http://www.gbif.org/dataset/84806e86-f762-11e1-a439-00145eb45e9a |
| University of California, Santa Barbara Marine Science Institute. Paleobiology Database. Accessed via Global Biodiversity Information Facility at http://www.gbif.org/dataset/84806e86-f762-11e1-a439-00145eb45e9a on May 14, 2014. | http://www.gbif.org/dataset/84806e86-f762-11e1-a439-00145eb45e9a |
| University of California, Santa Barbara Marine Science Institute. Paleobiology Database. Accessed via Global Biodiversity Information Facility at http://www.gbif.org/dataset/84806e86-f762-11e1-a439-00145eb45e9a on May 14, 2014. | http://www.gbif.org/dataset/84806e86-f762-11e1-a439-00145eb45e9a |
| Canadian Biodiversity Information Facility. Provincial Museum of Alberta. Accessed via Global Biodiversity Information Facility at http://www.gbif.org/dataset/843df0c4-f762-11e1-a439-00145eb45e9a on May 14, 2014. | http://www.gbif.org/dataset/843df0c4-f762-11e1-a439-00145eb45e9a |
| Avian Knowledge Network. Point Reyes Bird Observatory - Point Counts. Accessed via Global Biodiversity Information Facility at http://www.gbif.org/dataset/864c8736-f762-11e1-a439-00145eb45e9a on July 22, 2014. | http://www.gbif.org/dataset/864c8736-f762-11e1-a439-00145eb45e9a |
| James R. Slater Museum of Natural History. Bird Collection. Accessed via Global Biodiversity Information Facility at http://www.gbif.org/dataset/8eddc200-f535-4c65-9b4d-f723eafe607e on July 22, 2014. | http://www.gbif.org/dataset/8eddc200-f535-4c65-9b4d-f723eafe607e |
| Royal Belgian Institute of Natural Sciences. Bird Collection. Accessed via Global Biodiversity Information Facility at http://www.gbif.org/dataset/8138eb72-f762-11e1-a439-00145eb45e9a on July 22, 2014. | http://www.gbif.org/dataset/8138eb72-f762-11e1-a439-00145eb45e9a |
| Royal Ontario Museum. Ornithology Collection Non Passeriformes. Accessed via Global Biodiversity Information Facility at http://www.gbif.org/dataset/c0d6b7e8-8263-4224-8dac-32748d945555 on July 22, 2014. | http://www.gbif.org/dataset/c0d6b7e8-8263-4224-8dac-32748d945555 |
| Avian Knowledge Network. Redwood Sciences Laboratory - Lamna Point Count. Accessed via Global Biodiversity Information Facility at http://www.gbif.org/dataset/864da4c2-f762-11e1-a439-00145eb45e9a on May 14, 2014. | http://www.gbif.org/dataset/864da4c2-f762-11e1-a439-00145eb45e9a |
| Santa Barbara Museum of Natural History. Bird Collection. Accessed via Global Biodiversity Information Facility at http://www.gbif.org/dataset/75018539-6328-41de-b875-7c2e61dc1635 on July 22, 2014. | http://www.gbif.org/dataset/75018539-6328-41de-b875-7c2e61dc1635 |
| San Diego Natural History Museum. Bird specimens. Accessed via Global Biodiversity Information Facility at http://www.gbif.org/dataset/84b26828-f762-11e1-a439-00145eb45e9a on July 22, 2014. | http://www.gbif.org/dataset/84b26828-f762-11e1-a439-00145eb45e9a |
| Senckenberg Nature Research Society. Bird Skin Collection. Accessed via Global Biodiversity Information Facility at http://www.gbif.org/dataset/96678e90-f762-11e1-a439-00145eb45e9a on July 22, 2014. | http://www.gbif.org/dataset/96678e90-f762-11e1-a439-00145eb45e9a |
| University of California, Los Angeles. Dickey Collection, Birds. Accessed via Global Biodiversity Information Facility at http://www.gbif.org/dataset/8631295a-f762-11e1-a439-00145eb45e9a on July 22, 2014. | http://www.gbif.org/dataset/8631295a-f762-11e1-a439-00145eb45e9a |
| University of Colorado Museum of Natural History. Bird Collection. Accessed via Global Biodiversity Information Facility at http://www.gbif.org/dataset/89337996-f762-11e1-a439-00145eb45e9a on July 22, 2014. | http://www.gbif.org/dataset/89337996-f762-11e1-a439-00145eb45e9a |
| University of Michigan Museum of Zoology. Birds Collection. Accessed via Global Biodiversity Information Facility at http://www.gbif.org/dataset/be5507b9-7abf-4b69-afe1-5ca2b7561734 on July 22, 2014. | http://www.gbif.org/dataset/be5507b9-7abf-4b69-afe1-5ca2b7561734 |
| University of Nebraska State Museum. Vertebrate Specimens. Accessed via Global Biodiversity Information Facility at http://www.gbif.org/dataset/851ab8c4-f762-11e1-a439-00145eb45e9a on May 14, 2014. | http://www.gbif.org/dataset/851ab8c4-f762-11e1-a439-00145eb45e9a |
| Smithsonian Institution National Museum of Natural History. Bird Collection. Accessed via Global Biodiversity Information Facility at http://www.gbif.org/dataset/5df38344-b821-49c2-8174-cf0f29f4df0d on July 22, 2014. | http://www.gbif.org/dataset/5df38344-b821-49c2-8174-cf0f29f4df0d |
| University of Washington Burke Museum. Ornithology Collection. Accessed via Global Biodiversity Information Facility at http://www.gbif.org/dataset/830fd460-f762-11e1-a439-00145eb45e9a on July 22, 2014. | http://www.gbif.org/dataset/830fd460-f762-11e1-a439-00145eb45e9a |
| University of Wyoming Museum of Vertebrates. Bird Collection. Accessed via Global Biodiversity Information Facility at http://www.gbif.org/dataset/abcaccad-9e01-4b2a-b493-32531cbed32a on May 14, 2014. | http://www.gbif.org/dataset/abcaccad-9e01-4b2a-b493-32531cbed32a |
| University of Wyoming Museum of Vertebrates. Bird Collection. Accessed via Global Biodiversity Information Facility at http://www.gbif.org/dataset/abcaccad-9e01-4b2a-b493-32531cbed32a on May 14, 2014. | http://www.gbif.org/dataset/abcaccad-9e01-4b2a-b493-32531cbed32a |
| Western Australian Museum. Western Australian Museum provider for OZCAM. Accessed via Global Biodiversity Information Facility at http://www.gbif.org/dataset/7c93d290-6c8b-11de-8226-b8a03c50a862 on May 14, 2014. | http://www.gbif.org/dataset/7c93d290-6c8b-11de-8226-b8a03c50a862 |
| Western Foundation of Vertebrate Zoology. Bird Collection. Accessed via Global Biodiversity Information Facility at http://www.gbif.org/dataset/8be43f9b-52e7-47d4-be3e-dbcc066d70ab on July 22, 2014. | http://www.gbif.org/dataset/8be43f9b-52e7-47d4-be3e-dbcc066d70ab |
| Western Foundation of Vertebrate Zoology. Egg Collection. Accessed via Global Biodiversity Information Facility at http://www.gbif.org/dataset/8be43f9b-52e7-47d4-be3e-dbcc066d70ab on July 22, 2014. | http://www.gbif.org/dataset/8be43f9b-52e7-47d4-be3e-dbcc066d70ab |
| Wildlife Sightings. Citizen Science Data. Accessed via Global Biodiversity Information Facility at http://www.gbif.org/dataset/b70121ef-b7ea-4316-a05b-abdf30f5ca09 on May 14, 2014. | http://www.gbif.org/dataset/b70121ef-b7ea-4316-a05b-abdf30f5ca09 |
| Yale University Peabody Museum. Vertebrate Zoology Division - Ornithology. Accessed via Global Biodiversity Information Facility at http://www.gbif.org/dataset/854cf79e-f762-11e1-a439-00145eb45e9a on July 22, 2014. | http://www.gbif.org/dataset/854cf79e-f762-11e1-a439-00145eb45e9a |
| Ballard, G., M. Herzog, M. Fitzgibbon, D. Moody, D. Jongsomjit, D. Stralberg. 2008. California Avian Datacenter, Level 3 - Bodie Hills and Long Valley Greater Sage Grouse PMU. [web application]. Petaluma, California. Accessed at http://data.prbo.org/cadc2 | http://data.prbo.org/cadc2/ |
| Ballard, G., M. Herzog, M. Fitzgibbon, D. Moody, D. Jongsomjit, D. Stralberg. 2008. California Avian Datacenter, Level 3 - Central Coast. [web application]. Petaluma, California. Accessed at http://data.prbo.org/cadc2 on July 21, 2014. | http://data.prbo.org/cadc2/ |
| Ballard, G., M. Herzog, M. Fitzgibbon, D. Moody, D. Jongsomjit, D. Stralberg. 2008. California Avian Datacenter, Level 3 - Clear Creek. [web application]. Petaluma, California. Accessed at http://data.prbo.org/cadc2 on July 21, 2014. | http://data.prbo.org/cadc2/ |
| Ballard, G., M. Herzog, M. Fitzgibbon, D. Moody, D. Jongsomjit, D. Stralberg. 2008. California Avian Datacenter, Level 3 - Coastal National Park Service Monitoring. [web application]. Petaluma, California. Accessed at http://data.prbo.org/cadc2 on July 21 | http://data.prbo.org/cadc2/ |
| Ballard, G., M. Herzog, M. Fitzgibbon, D. Moody, D. Jongsomjit, D. Stralberg. 2008. California Avian Datacenter, Level 3 - Cosumnes River. [web application]. Petaluma, California. Accessed at http://data.prbo.org/cadc2 on July 21, 2014. | http://data.prbo.org/cadc2/ |
| Ballard, G., M. Herzog, M. Fitzgibbon, D. Moody, D. Jongsomjit, D. Stralberg. 2008. California Avian Datacenter, Level 3 - Devil's Postpile National Monument. [web application]. Petaluma, California. Accessed at http://data.prbo.org/cadc2 on July 21, 2014 | http://data.prbo.org/cadc2/ |
| Ballard, G., M. Herzog, M. Fitzgibbon, D. Moody, D. Jongsomjit, D. Stralberg. 2008. California Avian Datacenter, Level 3 - East/West Walker River. [web application]. Petaluma, California. Accessed at http://data.prbo.org/cadc2 on July 21, 2014. | http://data.prbo.org/cadc2/ |
| Ballard, G., M. Herzog, M. Fitzgibbon, D. Moody, D. Jongsomjit, D. Stralberg. 2008. California Avian Datacenter, Level 3 - Inyo National Forest Aspen Enhancement Project. [web application]. Petaluma, California. Accessed at http://data.prbo.org/cadc2 on J | http://data.prbo.org/cadc2/ |
| Ballard, G., M. Herzog, M. Fitzgibbon, D. Moody, D. Jongsomjit, D. Stralberg. 2008. California Avian Datacenter, Level 3 - Klamath Bird Observatory Fuel Reduction Klamath National Forest. [web application]. Petaluma, California. Accessed at http://data.p | http://data.prbo.org/cadc2/ |
| Ballard, G., M. Herzog, M. Fitzgibbon, D. Moody, D. Jongsomjit, D. Stralberg. 2008. California Avian Datacenter, Level 3 - Klamath Bird Observatory Inventory Klamath Network Lava Beds. [web application]. Petaluma, California. Accessed at http://data.prbo | http://data.prbo.org/cadc2/ |
| Ballard, G., M. Herzog, M. Fitzgibbon, D. Moody, D. Jongsomjit, D. Stralberg. 2008. California Avian Datacenter, Level 3 - Klamath Bird Observatory Inventory Klamath Network Redwoods. [web application]. Petaluma, California. Accessed at http://data.prbo. | http://data.prbo.org/cadc2/ |
| Ballard, G., M. Herzog, M. Fitzgibbon, D. Moody, D. Jongsomjit, D. Stralberg. 2008. California Avian Datacenter, Level 3 - Klamath Bird Observatory Inventory Klamath Network Whiskeytown. [web application]. Petaluma, California. Accessed at http://data.pr | http://data.prbo.org/cadc2/ |
| Ballard, G., M. Herzog, M. Fitzgibbon, D. Moody, D. Jongsomjit, D. Stralberg. 2008. California Avian Datacenter, Level 3 - Klamath Bird Observatory Longterm Breeding Bird Survey. [web application]. Petaluma, California. Accessed at http://data.prbo.org/c | http://data.prbo.org/cadc2/ |
| Ballard, G., M. Herzog, M. Fitzgibbon, D. Moody, D. Jongsomjit, D. Stralberg. 2008. California Avian Datacenter, Level 3 - Klamath Bird Observatory Longterm Klamath Network Lassen. [web application]. Petaluma, California. Accessed at http://data.prbo.org | http://data.prbo.org/cadc2/ |
| Ballard, G., M. Herzog, M. Fitzgibbon, D. Moody, D. Jongsomjit, D. Stralberg. 2008. California Avian Datacenter, Level 3 - Klamath Bird Observatory Longterm Klamath Network Lava Beds. [web application]. Petaluma, California. Accessed at http://data.prbo. | http://data.prbo.org/cadc2/ |
| Ballard, G., M. Herzog, M. Fitzgibbon, D. Moody, D. Jongsomjit, D. Stralberg. 2008. California Avian Datacenter, Level 3 - Klamath Bird Observatory Longterm Klamath Network Redwoods. [web application]. Petaluma, California. Accessed at http://data.prbo.o | http://data.prbo.org/cadc2/ |
| Ballard, G., M. Herzog, M. Fitzgibbon, D. Moody, D. Jongsomjit, D. Stralberg. 2008. California Avian Datacenter, Level 3 - Klamath Bird Observatory Longterm Klamath Network Whiskeytown. [web application]. Petaluma, California. Accessed at http://data.prb | http://data.prbo.org/cadc2/ |
| Ballard, G., M. Herzog, M. Fitzgibbon, D. Moody, D. Jongsomjit, D. Stralberg. 2008. California Avian Datacenter, Level 3 - Klamath Bird Observatory Longterm Northern California. [web application]. Petaluma, California. Accessed at http://data.prbo.org/ca | http://data.prbo.org/cadc2/ |
| Ballard, G., M. Herzog, M. Fitzgibbon, D. Moody, D. Jongsomjit, D. Stralberg. 2008. California Avian Datacenter, Level 3 - Klamath Bird Observatory Water Management Marsh Passerines. [web application]. Petaluma, California. Accessed at http://data.prbo.o | http://data.prbo.org/cadc2/ |
| Ballard, G., M. Herzog, M. Fitzgibbon, D. Moody, D. Jongsomjit, D. Stralberg. 2008. California Avian Datacenter, Level 3 - Klamath Bird Observatory Water Management Trinity. [web application]. Petaluma, California. Accessed at http://data.prbo.org/cadc2 | http://data.prbo.org/cadc2/ |
| Ballard, G., M. Herzog, M. Fitzgibbon, D. Moody, D. Jongsomjit, D. Stralberg. 2008. California Avian Datacenter, Level 3 - Laguna de Santa Rosa. [web application]. Petaluma, California. Accessed at http://data.prbo.org/cadc2 on July 21, 2014. | http://data.prbo.org/cadc2/ |
| Ballard, G., M. Herzog, M. Fitzgibbon, D. Moody, D. Jongsomjit, D. Stralberg. 2008. California Avian Datacenter, Level 3 - Lands End. [web application]. Petaluma, California. Accessed at http://data.prbo.org/cadc2 on July 21, 2014. | http://data.prbo.org/cadc2/ |
| Ballard, G., M. Herzog, M. Fitzgibbon, D. Moody, D. Jongsomjit, D. Stralberg. 2008. California Avian Datacenter, Level 3 - Lassen Aspen. [web application]. Petaluma, California. Accessed at http://data.prbo.org/cadc2 on July 21, 2014. | http://data.prbo.org/cadc2/ |
| Ballard, G., M. Herzog, M. Fitzgibbon, D. Moody, D. Jongsomjit, D. Stralberg. 2008. California Avian Datacenter, Level 3 - Lassen Black Oak. [web application]. Petaluma, California. Accessed at http://data.prbo.org/cadc2 on July 21, 2014. | http://data.prbo.org/cadc2/ |
| Ballard, G., M. Herzog, M. Fitzgibbon, D. Moody, D. Jongsomjit, D. Stralberg. 2008. California Avian Datacenter, Level 3 - Lassen Foothills Oak. [web application]. Petaluma, California. Accessed at http://data.prbo.org/cadc2 on July 21, 2014. | http://data.prbo.org/cadc2/ |
| Ballard, G., M. Herzog, M. Fitzgibbon, D. Moody, D. Jongsomjit, D. Stralberg. 2008. California Avian Datacenter, Level 3 - Lassen Management Indicator Species. [web application]. Petaluma, California. Accessed at http://data.prbo.org/cadc2 on July 21, 201 | http://data.prbo.org/cadc2/ |
| Ballard, G., M. Herzog, M. Fitzgibbon, D. Moody, D. Jongsomjit, D. Stralberg. 2008. California Avian Datacenter, Level 3 - Lassen National Forest Fuels Treatment. [web application]. Petaluma, California. Accessed at http://data.prbo.org/cadc2 on July 21, | http://data.prbo.org/cadc2/ |
| Ballard, G., M. Herzog, M. Fitzgibbon, D. Moody, D. Jongsomjit, D. Stralberg. 2008. California Avian Datacenter, Level 3 - LNF Inventory. [web application]. Petaluma, California. Accessed at http://data.prbo.org/cadc2 on July 21, 2014. | http://data.prbo.org/cadc2/ |
| Ballard, G., M. Herzog, M. Fitzgibbon, D. Moody, D. Jongsomjit, D. Stralberg. 2008. California Avian Datacenter, Level 3 - Long Valley Road Closure Project. [web application]. Petaluma, California. Accessed at http://data.prbo.org/cadc2 on July 21, 2014. | http://data.prbo.org/cadc2/ |
| Ballard, G., M. Herzog, M. Fitzgibbon, D. Moody, D. Jongsomjit, D. Stralberg. 2008. California Avian Datacenter, Level 3 - Lower Owens River Project. [web application]. Petaluma, California. Accessed at http://data.prbo.org/cadc2 on July 21, 2014. | http://data.prbo.org/cadc2/ |
| Ballard, G., M. Herzog, M. Fitzgibbon, D. Moody, D. Jongsomjit, D. Stralberg. 2008. California Avian Datacenter, Level 3 - Lower Sacramento River. [web application]. Petaluma, California. Accessed at http://data.prbo.org/cadc2 on July 21, 2014. | http://data.prbo.org/cadc2/ |
| Ballard, G., M. Herzog, M. Fitzgibbon, D. Moody, D. Jongsomjit, D. Stralberg. 2008. California Avian Datacenter, Level 3 - Marble Creek. [web application]. Petaluma, California. Accessed at http://data.prbo.org/cadc2 on July 21, 2014. | http://data.prbo.org/cadc2/ |
| Ballard, G., M. Herzog, M. Fitzgibbon, D. Moody, D. Jongsomjit, D. Stralberg. 2008. California Avian Datacenter, Level 3 - Marin County Open Space. [web application]. Petaluma, California. Accessed at http://data.prbo.org/cadc2 on July 21, 2014. | http://data.prbo.org/cadc2/ |
| Ballard, G., M. Herzog, M. Fitzgibbon, D. Moody, D. Jongsomjit, D. Stralberg. 2008. California Avian Datacenter, Level 3 - Merced River. [web application]. Petaluma, California. Accessed at http://data.prbo.org/cadc2 on July 21, 2014. | http://data.prbo.org/cadc2/ |
| Ballard, G., M. Herzog, M. Fitzgibbon, D. Moody, D. Jongsomjit, D. Stralberg. 2008. California Avian Datacenter, Level 3 - MMWD Monitoring. [web application]. Petaluma, California. Accessed at http://data.prbo.org/cadc2 on July 21, 2014. | http://data.prbo.org/cadc2/ |
| Ballard, G., M. Herzog, M. Fitzgibbon, D. Moody, D. Jongsomjit, D. Stralberg. 2008. California Avian Datacenter, Level 3 - Mono Basin Riparian. [web application]. Petaluma, California. Accessed at http://data.prbo.org/cadc2 on July 21, 2014. | http://data.prbo.org/cadc2/ |
| Ballard, G., M. Herzog, M. Fitzgibbon, D. Moody, D. Jongsomjit, D. Stralberg. 2008. California Avian Datacenter, Level 3 - Owens Valley Alluvial Fan. [web application]. Petaluma, California. Accessed at http://data.prbo.org/cadc2 on July 21, 2014. | http://data.prbo.org/cadc2/ |
| Ballard, G., M. Herzog, M. Fitzgibbon, D. Moody, D. Jongsomjit, D. Stralberg. 2008. California Avian Datacenter, Level 3 - Palomarin. [web application]. Petaluma, California. Accessed at http://data.prbo.org/cadc2 on July 21, 2014. | http://data.prbo.org/cadc2/ |
| Ballard, G., M. Herzog, M. Fitzgibbon, D. Moody, D. Jongsomjit, D. Stralberg. 2008. California Avian Datacenter, Level 3 - Pinnacles National Monument. [web application]. Petaluma, California. Accessed at http://data.prbo.org/cadc2 on July 21, 2014. | http://data.prbo.org/cadc2/ |
| Ballard, G., M. Herzog, M. Fitzgibbon, D. Moody, D. Jongsomjit, D. Stralberg. 2008. California Avian Datacenter, Level 3 - Plumas/Lassen. [web application]. Petaluma, California. Accessed at http://data.prbo.org/cadc2 on July 21, 2014. | http://data.prbo.org/cadc2/ |
| Ballard, G., M. Herzog, M. Fitzgibbon, D. Moody, D. Jongsomjit, D. Stralberg. 2008. California Avian Datacenter, Level 3 - Presidio. [web application]. Petaluma, California. Accessed at http://data.prbo.org/cadc2 on July 21, 2014. | http://data.prbo.org/cadc2/ |
| Ballard, G., M. Herzog, M. Fitzgibbon, D. Moody, D. Jongsomjit, D. Stralberg. 2008. California Avian Datacenter, Level 3 - Rancheria Gulch. [web application]. Petaluma, California. Accessed at http://data.prbo.org/cadc2 on July 21, 2014. | http://data.prbo.org/cadc2/ |
| Ballard, G., M. Herzog, M. Fitzgibbon, D. Moody, D. Jongsomjit, D. Stralberg. 2008. California Avian Datacenter, Level 3 - United States Forest Service Redwood Sciences Library COOPMONITORINGBLM. [web application]. Petaluma, California. Accessed at http:/ | http://data.prbo.org/cadc2/ |
| Ballard, G., M. Herzog, M. Fitzgibbon, D. Moody, D. Jongsomjit, D. Stralberg. 2008. California Avian Datacenter, Level 3 - United States Forest Service Redwood Sciences Library COOPMONITORINGCCC. [web application]. Petaluma, California. Accessed at http:/ | http://data.prbo.org/cadc2/ |
| Ballard, G., M. Herzog, M. Fitzgibbon, D. Moody, D. Jongsomjit, D. Stralberg. 2008. California Avian Datacenter, Level 3 - United States Forest Service Redwood Sciences Library COOPMONITORINGFRANKLAKE. [web application]. Petaluma, California. Accessed at | http://data.prbo.org/cadc2/ |
| Ballard, G., M. Herzog, M. Fitzgibbon, D. Moody, D. Jongsomjit, D. Stralberg. 2008. California Avian Datacenter, Level 3 - United States Forest Service Redwood Sciences Library LBMETHODSLONGTERMMON. [web application]. Petaluma, California. Accessed at htt | http://data.prbo.org/cadc2/ |
| Ballard, G., M. Herzog, M. Fitzgibbon, D. Moody, D. Jongsomjit, D. Stralberg. 2008. California Avian Datacenter, Level 3 - United States Forest Service Redwood Sciences Library TRRPMAINSTEM. [web application]. Petaluma, California. Accessed at http://data | http://data.prbo.org/cadc2/ |
| Ballard, G., M. Herzog, M. Fitzgibbon, D. Moody, D. Jongsomjit, D. Stralberg. 2008. California Avian Datacenter, Level 3 - United States Forest Service Redwood Sciences Library TRRPSOUTHFORK. [web application]. Petaluma, California. Accessed at http://dat | http://data.prbo.org/cadc2/ |
| Ballard, G., M. Herzog, M. Fitzgibbon, D. Moody, D. Jongsomjit, D. Stralberg. 2008. California Avian Datacenter, Level 3 - United States Forest Service Redwood Sciences Library TRRPTRIBS. [web application]. Petaluma, California. Accessed at http://data.pr | http://data.prbo.org/cadc2/ |
| Ballard, G., M. Herzog, M. Fitzgibbon, D. Moody, D. Jongsomjit, D. Stralberg. 2008. California Avian Datacenter, Level 3 - United States Forest Service Redwood Sciences Library WILDFIREBISCUIT. [web application]. Petaluma, California. Accessed at http://d | http://data.prbo.org/cadc2/ |
| Ballard, G., M. Herzog, M. Fitzgibbon, D. Moody, D. Jongsomjit, D. Stralberg. 2008. California Avian Datacenter, Level 3 - United States Forest Service Redwood Sciences Library WILDFIRECANOE. [web application]. Petaluma, California. Accessed at http://dat | http://data.prbo.org/cadc2/ |
| Ballard, G., M. Herzog, M. Fitzgibbon, D. Moody, D. Jongsomjit, D. Stralberg. 2008. California Avian Datacenter, Level 3 - United States Forest Service Redwood Sciences Library WILDFIRELEWISTON. [web application]. Petaluma, California. Accessed at http:// | http://data.prbo.org/cadc2/ |
| Ballard, G., M. Herzog, M. Fitzgibbon, D. Moody, D. Jongsomjit, D. Stralberg. 2008. California Avian Datacenter, Level 3 - United States Forest Service Redwood Sciences Library WILDFIREMEGRAM. [web application]. Petaluma, California. Accessed at http://da | http://data.prbo.org/cadc2/ |
| Ballard, G., M. Herzog, M. Fitzgibbon, D. Moody, D. Jongsomjit, D. Stralberg. 2008. California Avian Datacenter, Level 3 - San Joaquin BOR. [web application]. Petaluma, California. Accessed at http://data.prbo.org/cadc2 on July 21, 2014. | http://data.prbo.org/cadc2/ |
| Ballard, G., M. Herzog, M. Fitzgibbon, D. Moody, D. Jongsomjit, D. Stralberg. 2008. California Avian Datacenter, Level 3 - San Joaquin Experimental Range. [web application]. Petaluma, California. Accessed at http://data.prbo.org/cadc2 on July 21, 2014. | http://data.prbo.org/cadc2/ |
| Ballard, G., M. Herzog, M. Fitzgibbon, D. Moody, D. Jongsomjit, D. Stralberg. 2008. California Avian Datacenter, Level 3 - San Joaquin River National Wildlife Refuge. [web application]. Petaluma, California. Accessed at http://data.prbo.org/cadc2 on July | http://data.prbo.org/cadc2/ |
| Ballard, G., M. Herzog, M. Fitzgibbon, D. Moody, D. Jongsomjit, D. Stralberg. 2008. California Avian Datacenter, Level 3 - Sierra Meadows. [web application]. Petaluma, California. Accessed at http://data.prbo.org/cadc2 on July 21, 2014. | http://data.prbo.org/cadc2/ |
| Ballard, G., M. Herzog, M. Fitzgibbon, D. Moody, D. Jongsomjit, D. Stralberg. 2008. California Avian Datacenter, Level 3 - Sierra Nevada Mgmt Indicator Spp. [web application]. Petaluma, California. Accessed at http://data.prbo.org/cadc2 on July 21, 2014. | http://data.prbo.org/cadc2/ |
| Ballard, G., M. Herzog, M. Fitzgibbon, D. Moody, D. Jongsomjit, D. Stralberg. 2008. California Avian Datacenter, Level 3 - Sonoma Oaks. [web application]. Petaluma, California. Accessed at http://data.prbo.org/cadc2 on July 21, 2014. | http://data.prbo.org/cadc2/ |
| Ballard, G., M. Herzog, M. Fitzgibbon, D. Moody, D. Jongsomjit, D. Stralberg. 2008. California Avian Datacenter, Level 3 - Sonoma Riparian. [web application]. Petaluma, California. Accessed at http://data.prbo.org/cadc2 on July 21, 2014. | http://data.prbo.org/cadc2/ |
| Ballard, G., M. Herzog, M. Fitzgibbon, D. Moody, D. Jongsomjit, D. Stralberg. 2008. California Avian Datacenter, Level 3 - Susanville. [web application]. Petaluma, California. Accessed at http://data.prbo.org/cadc2 on July 21, 2014. | http://data.prbo.org/cadc2/ |
| Ballard, G., M. Herzog, M. Fitzgibbon, D. Moody, D. Jongsomjit, D. Stralberg. 2008. California Avian Datacenter, Level 3 - Tidal Marsh. [web application]. Petaluma, California. Accessed at http://data.prbo.org/cadc2 on July 21, 2014. | http://data.prbo.org/cadc2/ |
| Ballard, G., M. Herzog, M. Fitzgibbon, D. Moody, D. Jongsomjit, D. Stralberg. 2008. California Avian Datacenter, Level 3 - Upper Owens River Watershed. [web application]. Petaluma, California. Accessed at http://data.prbo.org/cadc2 on July 21, 2014. | http://data.prbo.org/cadc2/ |
| Ballard, G., M. Herzog, M. Fitzgibbon, D. Moody, D. Jongsomjit, D. Stralberg. 2008. California Avian Datacenter, Level 5 - Big Sur Ornithology Lab. [web application]. Petaluma, California. Accessed at http://data.prbo.org/cadc2 on July 21, 2014. | http://data.prbo.org/cadc2/ |
| Ballard, G., M. Herzog, M. Fitzgibbon, D. Moody, D. Jongsomjit, D. Stralberg. 2008. California Avian Datacenter, Level 5 - BOR Grasslands. [web application]. Petaluma, California. Accessed at http://data.prbo.org/cadc2 on July 21, 2014. | http://data.prbo.org/cadc2/ |
| Ballard, G., M. Herzog, M. Fitzgibbon, D. Moody, D. Jongsomjit, D. Stralberg. 2008. California Avian Datacenter, Level 5 - Lassen Foothills Riparian. [web application]. Petaluma, California. Accessed at http://data.prbo.org/cadc2 on July 21, 2014. | http://data.prbo.org/cadc2/ |
| Ballard, G., M. Herzog, M. Fitzgibbon, D. Moody, D. Jongsomjit, D. Stralberg. 2008. California Avian Datacenter, Level 5 - Monterey RCD. [web application]. Petaluma, California. Accessed at http://data.prbo.org/cadc2 on July 21, 2014. | http://data.prbo.org/cadc2/ |
| Schoenig, S. 2009. Bird Species of Special Concern. Digitized range information from W.D. Shuford and T. Gardali, eds. 2008. California Bird Species of Special Concern. 2008. Studies in Western Birds No. 1. Accessed July 22, 2014. Accessed via Biogeographic Information and Oservation System at http://www.dfg.ca.gov/biogeodata/bios/ in 2012. | http://www.dfg.ca.gov/biogeodata/bios/ |
| US Geological Survey Southeast Ecological Science Center. 2011. Nonindigenous Aquatic Species Database. Gainsville, FL. Accessed at http://nas.er.usgs.gov in 2012. | http://nas.er.usgs.gov/ |
| California Academy of Sciences. Mammalogy Collection. Accessed via Global Biodiversity Information Facility at www.gbif.org/dataset/6ce7290f-47f6-4046-8356-371f5b6749df on September 18, 2014. | www.gbif.org/dataset/6ce7290f-47f6-4046-8356-371f5b6749df |
| Cornell University Museum of Vertebrates. Mammal Collection. Accessed via Global Biodiversity Information Facility at www.gbif.org/dataset/35720b3e-aded-4b83-b4f1-967f1d457d6a on September 18, 2014. | www.gbif.org/dataset/35720b3e-aded-4b83-b4f1-967f1d457d6a |
| Cornell University Museum of Vertebrates. Mammal Collection. Accessed via Global Biodiversity Information Facility at www.gbif.org/dataset/35720b3e-aded-4b83-b4f1-967f1d457d6a on September 18, 2014. | www.gbif.org/dataset/35720b3e-aded-4b83-b4f1-967f1d457d6a |
| Field Museum of Natural History (Zoology). Mammal Collection. Accessed via Global Biodiversity Information Facility at www.gbif.org/dataset/41fc5c40-5e81-496f-9733-6b5681b3b7a5 on September 18, 2014. | www.gbif.org/dataset/41fc5c40-5e81-496f-9733-6b5681b3b7a5 |
| University of Kansas Biodiversity Institute. Mammalogy Collection. Accessed via Global Biodiversity Information Facility at www.gbif.org/dataset/1d04e739-98a9-4e16-9970-8f8f3bf9e9e3 on September 18, 2014. | www.gbif.org/dataset/1d04e739-98a9-4e16-9970-8f8f3bf9e9e3 |
| Natural History Museum of Los Angeles County. Mammal Collection. Accessed via Global Biodiversity Information Facility at www.gbif.org/dataset/7a25f7aa-03fb-4322-aaeb-66719e1a9527 on September 18, 2014. | www.gbif.org/dataset/7a25f7aa-03fb-4322-aaeb-66719e1a9527 |
| Louisiana State University Herbarium. Mammals Collection. Accessed via Global Biodiversity Information Facility at www.gbif.org/dataset/847e2306-f762-11e1-a439-00145eb45e9a on September 18, 2014. | www.gbif.org/dataset/847e2306-f762-11e1-a439-00145eb45e9a |
| Museum of Southwestern Biology. Mammal Collection. Accessed via Global Biodiversity Information Facility at www.gbif.org/dataset/b15d4952-7d20-46f1-8a3e-556a512b04c5 on September 18, 2014. | www.gbif.org/dataset/b15d4952-7d20-46f1-8a3e-556a512b04c5 |
| Museum of Vertebrate Zoology. Mammal Collection. Accessed via Global Biodiversity Information Facility at www.gbif.org/dataset/0daed095-478a-4af6-abf5-18acb790fbb2 on September 18, 2014. | www.gbif.org/dataset/0daed095-478a-4af6-abf5-18acb790fbb2 |
| University of California, Santa Barbara Marine Science Institute. Paleobiology Database. Accessed via Global Biodiversity Information Facility at www.gbif.org/dataset/84806e86-f762-11e1-a439-00145eb45e9a on May 14, 2014. | www.gbif.org/dataset/84806e86-f762-11e1-a439-00145eb45e9a |
| James R. Slater Museum of Natural History. Mammal Collection. Accessed via Global Biodiversity Information Facility at www.gbif.org/dataset/8eddc200-f535-4c65-9b4d-f723eafe607e on September 18, 2014. | www.gbif.org/dataset/8eddc200-f535-4c65-9b4d-f723eafe607e |
| Royal Ontario Museum. Mammalogy Collection. Accessed via Global Biodiversity Information Facility at www.gbif.org/dataset/c5c4a23e-2035-4416-ab64-032d6df52ddb on September 18, 2014. | www.gbif.org/dataset/c5c4a23e-2035-4416-ab64-032d6df52ddb |
| Santa Barbara Museum of Natural History. Mammal Collection. Accessed via Global Biodiversity Information Facility at www.gbif.org/dataset/75018539-6328-41de-b875-7c2e61dc1635 on September 18, 2014. | www.gbif.org/dataset/75018539-6328-41de-b875-7c2e61dc1635 |
| Museum of Texas Tech University. Mammals Collection. Accessed via Global Biodiversity Information Facility at www.gbif.org/dataset/854f70cc-55e3-4af2-9417-0f47d6c7902d on September 18, 2014. | www.gbif.org/dataset/854f70cc-55e3-4af2-9417-0f47d6c7902d |
| University of California, Los Angeles. Dickey Collection, Mammals. Accessed via Global Biodiversity Information Facility at www.gbif.org/dataset/8631295a-f762-11e1-a439-00145eb45e9a on September 18, 2014. | www.gbif.org/dataset/8631295a-f762-11e1-a439-00145eb45e9a |
| University of Michigan Museum of Zoology. Mammal Collection. Accessed via Global Biodiversity Information Facility at www.gbif.org/dataset/6d2cfc0a-9903-40b8-802b-403398218e4a on September 18, 2014. | www.gbif.org/dataset/6d2cfc0a-9903-40b8-802b-403398218e4a |
