# Supplementary material for: Patterns of Freshwater Species Richness, Endemism, and Vulnerability in California
Source: PLoS One. 2015 Jul 6;10(7):e0130710. doi: 10.1371/journal.pone.0130710 (PMC4493109; doi:10.1371/journal.pone.0130710)
Supplement: S4 Table — (DOCX) [file pone.0130710.s005.docx]

**S4 Table.** List of taxa included in the database.

| **Scientific Name** | **Common Name** | **Group** |
| --- | --- | --- |
| Abedus breviceps |  | Insects & other |
| Abedus herberti |  | Insects & other |
| Abedus indentatus |  | Insects & other |
| Abedus ovatus |  | Insects & other |
| Abedus parkeri |  | Insects & other |
| Abedus vicinus |  | Insects & other |
| Ablabesmyia annulata |  | Insects & other |
| Ablabesmyia aspera |  | Insects & other |
| Ablabesmyia cinctipes |  | Insects & other |
| Ablabesmyia mallochi |  | Insects & other |
| Ablabesmyia monilis |  | Insects & other |
| Ablabesmyia peleensis |  | Insects & other |
| Acalyptonotus pacificus |  | Insects & other |
| Acanthomysis aspera |  | Crustaceans |
| Acanthomysis hwanhaiensis |  | Crustaceans |
| Acentrella insignificans | A Mayfly | Insects & other |
| Acentrella turbida | A Mayfly | Insects & other |
| Acerpenna pygmaea |  | Insects & other |
| Acilius abbreviatus |  | Insects & other |
| Acipenser medirostris ssp. 1 | Southern green sturgeon | Fishes |
| Acipenser medirostris ssp. 2 | Northern green sturgeon | Fishes |
| Acipenser transmontanus | White sturgeon | Fishes |
| Acneus beeri |  | Insects & other |
| Acneus burnelli |  | Insects & other |
| Acneus oregonensis |  | Insects & other |
| Acneus quadrimaculatus |  | Insects & other |
| Actinemys marmorata marmorata | Western Pond Turtle | Herps |
| Actinemys marmorata pallida | Southern Pacific Pond Turtle | Herps |
| Actitis macularius | Spotted Sandpiper | Birds |
| Aechmophorus clarkii | Clark's Grebe | Birds |
| Aechmophorus occidentalis | Western Grebe | Birds |
| Aedes aegypti |  | Insects & other |
| Aedes cinereus |  | Insects & other |
| Aedes vexans |  | Insects & other |
| Aeshna canadensis | Canada Darner | Insects & other |
| Aeshna interrupta interna |  | Insects & other |
| Aeshna juncea |  | Insects & other |
| Aeshna palmata | Paddle-tailed Darner | Insects & other |
| Aeshna persephone |  | Insects & other |
| Aeshna subarctica |  | Insects & other |
| Aeshna umbrosa occidentalis | Shadow Darner | Insects & other |
| Aeshna walkeri | Walker's Darner | Insects & other |
| Agabinus glabrellus |  | Insects & other |
| Agabinus sculpturellus |  | Insects & other |
| Agabus ancillus |  | Insects & other |
| Agabus anthracinus |  | Insects & other |
| Agabus apache |  | Insects & other |
| Agabus approximatus |  | Insects & other |
| Agabus austinii |  | Insects & other |
| Agabus austrodiscors |  | Insects & other |
| Agabus bjorkmanae |  | Insects & other |
| Agabus brevicollis |  | Insects & other |
| Agabus confertus |  | Insects & other |
| Agabus cordatus |  | Insects & other |
| Agabus discors |  | Insects & other |
| Agabus disintegratus |  | Insects & other |
| Agabus erichsoni |  | Insects & other |
| Agabus euryomus |  | Insects & other |
| Agabus griseipennis |  | Insects & other |
| Agabus hoppingi |  | Insects & other |
| Agabus hypomelas |  | Insects & other |
| Agabus ilybiiformis |  | Insects & other |
| Agabus jimzim |  | Insects & other |
| Agabus klamathensis |  | Insects & other |
| Agabus kootenai |  | Insects & other |
| Agabus lineelus |  | Insects & other |
| Agabus lugens |  | Insects & other |
| Agabus lutosus |  | Insects & other |
| Agabus minnesotensis |  | Insects & other |
| Agabus morosus |  | Insects & other |
| Agabus obliteratus nectris |  | Insects & other |
| Agabus obliteratus obliteratus |  | Insects & other |
| Agabus oblongulus |  | Insects & other |
| Agabus obsoletus |  | Insects & other |
| Agabus pandurus |  | Insects & other |
| Agabus perplexus |  | Insects & other |
| Agabus punctulatus |  | Insects & other |
| Agabus regularis |  | Insects & other |
| Agabus roguus |  | Insects & other |
| Agabus rumppi | Death Valley Agabus Diving Beetle | Insects & other |
| Agabus sasquatch |  | Insects & other |
| Agabus semivittatus |  | Insects & other |
| Agabus seriatus |  | Insects & other |
| Agabus smithi |  | Insects & other |
| Agabus strigulosus |  | Insects & other |
| Agabus tristis |  | Insects & other |
| Agabus vandykei |  | Insects & other |
| Agabus versimilis |  | Insects & other |
| Agabus walsinghami |  | Insects & other |
| Agapetus arcita | A Caddisfly | Insects & other |
| Agapetus bifidus |  | Insects & other |
| Agapetus boulderensis |  | Insects & other |
| Agapetus celatus | A Caddisfly | Insects & other |
| Agapetus denningi |  | Insects & other |
| Agapetus joannia | A Caddisfly | Insects & other |
| Agapetus malleatus | A Caddisfly | Insects & other |
| Agapetus marlo | A Caddisfly | Insects & other |
| Agapetus occidentis |  | Insects & other |
| Agapetus orosus | A Caddisfly | Insects & other |
| Agapetus taho | A Caddisfly | Insects & other |
| Agathon arizonica |  | Insects & other |
| Agathon aylmeri |  | Insects & other |
| Agathon comstocki |  | Insects & other |
| Agathon dismalea |  | Insects & other |
| Agathon doanei | A Net-winged Midge | Insects & other |
| Agathon elegantulus |  | Insects & other |
| Agathon markii |  | Insects & other |
| Agathon sequoiarum |  | Insects & other |
| Agelaius phoeniceus aciculatus | Kern Red-winged Blackbird | Birds |
| Agelaius tricolor | Tricolored Blackbird | Birds |
| Agraylea multipunctata |  | Insects & other |
| Agraylea saltesea | A Caddisfly | Insects & other |
| Agrostis oregonensis | Oregon Bentgrass | Plants |
| Agrypnia dextra |  | Insects & other |
| Agrypnia glacialis | A Caddisfly | Insects & other |
| Agrypnia improba |  | Insects & other |
| Agrypnia vestita |  | Insects & other |
| Aix sponsa | Wood Duck | Birds |
| Alienacanthomysis macropsis |  | Crustaceans |
| Alisma gramineum | Narrowleaf Water-plantain | Plants |
| Alisma triviale | Northern Water-plantain | Plants |
| Alisotrichia arizonica |  | Insects & other |
| Allium validum | Tall Swamp Onion | Plants |
| Allocosmoecus partitus | A Caddisfly | Insects & other |
| Allomyia acanthis |  | Insects & other |
| Allomyia cascadis |  | Insects & other |
| Allomyia cidoipes | A Caddisfly | Insects & other |
| Allomyia renoa |  | Insects & other |
| Alloperla chandleri | Mariposa Sallfly | Insects & other |
| Alloperla delicata | Delicate Sallfly | Insects & other |
| Alloperla elevata | A Stonefly | Insects & other |
| Alloperla fraterna | Cascades Sallfly | Insects & other |
| Alloperla thalia |  | Insects & other |
| Alnus rhombifolia | White Alder | Plants |
| Alnus rubra | Red Alder | Plants |
| Alnus viridis fruticosa | Siberian Alder | Plants |
| Alnus viridis sinuata | Sitka Alder | Plants |
| Alnus viridis virdis | Green Alder | Plants |
| Alopecurus aequalis aequalis | Short-awn Foxtail | Plants |
| Alopecurus aequalis sonomensis | Sonoma Shortawn Foxtail | Plants |
| Alopecurus carolinianus | Tufted Foxtail | Plants |
| Alopecurus geniculatus geniculatus | Meadow Foxtail | Plants |
| Alopecurus myosuroides | NA | Plants |
| Alopecurus pratensis | NA | Plants |
| Alopecurus saccatus | Pacific Foxtail | Plants |
| Alotanypus venustus |  | Insects & other |
| Ambrysus amargosus | Ash Meadows Naucorid | Insects & other |
| Ambrysus arizonus |  | Insects & other |
| Ambrysus californicus |  | Insects & other |
| Ambrysus circumcinctus |  | Insects & other |
| Ambrysus funebris | Nevares Spring Naucorid Bug | Insects & other |
| Ambrysus melanopterus |  | Insects & other |
| Ambrysus mormon |  | Insects & other |
| Ambrysus occidentalis |  | Insects & other |
| Ambrysus pulchellus |  | Insects & other |
| Ambrysus puncticollis |  | Insects & other |
| Ambrysus relictus |  | Insects & other |
| Ambrysus thermarum |  | Insects & other |
| Ambrysus woodburyi |  | Insects & other |
| Ambystoma californiense "Santa Barbara" | Santa Barbara Tiger Salamander | Herps |
| Ambystoma californiense "Sonoma" | Sonoma Tiger Salamander | Herps |
| Ambystoma californiense californiense | California Tiger Salamander | Herps |
| Ambystoma gracile | Northwestern Salamander | Herps |
| Ambystoma macrodactylum |  | Herps |
| Ambystoma macrodactylum croceum | Santa Cruz Long-toed Salamander | Herps |
| Ambystoma macrodactylum sigillatum | Southern Long-toed Salamander | Herps |
| Ameletus amador | A Mayfly | Insects & other |
| Ameletus andersoni | A Mayfly | Insects & other |
| Ameletus bellulus | A Mayfly | Insects & other |
| Ameletus celer | A Mayfly | Insects & other |
| Ameletus cooki | A Mayfly | Insects & other |
| Ameletus dissitus | A Mayfly | Insects & other |
| Ameletus doddsianus |  | Insects & other |
| Ameletus edmundsi | A Mayfly | Insects & other |
| Ameletus exquisitus |  | Insects & other |
| Ameletus falsus |  | Insects & other |
| Ameletus imbellis | A Mayfly | Insects & other |
| Ameletus majusculus | A Mayfly | Insects & other |
| Ameletus minimus | A Mayfly | Insects & other |
| Ameletus oregonensis |  | Insects & other |
| Ameletus pritchardi | A Mayfly | Insects & other |
| Ameletus quadratus |  | Insects & other |
| Ameletus shepherdi | A Mayfly | Insects & other |
| Ameletus similior | A Mayfly | Insects & other |
| Ameletus sparsatus | A Mayfly | Insects & other |
| Ameletus subnotatus | A Mayfly | Insects & other |
| Ameletus suffusus | A Mayfly | Insects & other |
| Ameletus tolae |  | Insects & other |
| Ameletus validus | A Mayfly | Insects & other |
| Ameletus vancouverensis | A Mayfly | Insects & other |
| Ameletus velox | A Mayfly | Insects & other |
| Ameletus vernalis | A Mayfly | Insects & other |
| Americorophium salmonis |  | Crustaceans |
| Americorophium spinicorne |  | Crustaceans |
| Americorophium stimpsoni |  | Crustaceans |
| Ametor latus |  | Insects & other |
| Ametor scabrosus |  | Insects & other |
| Ametropus ammophilus | A Mayfly | Insects & other |
| Amiocentrus aspilus | A Caddisfly | Insects & other |
| Ammannia coccinea | Scarlet Ammannia | Plants |
| Ammannia robusta | Grand Redstem | Plants |
| Amnicola limosa |  | Mollusks |
| Amphiagrion abbreviatum | Western Red Damsel | Insects & other |
| Amphicosmoecus canax | A Caddisfly | Insects & other |
| Amphinemura apache |  | Insects & other |
| Amphinemura mogollonica |  | Insects & other |
| Amphinemura venusta |  | Insects & other |
| Amphiscirpus nevadensis |  | Plants |
| Amphizoa insolens |  | Insects & other |
| Amphizoa lecontei |  | Insects & other |
| Amphizoa striata |  | Insects & other |
| Ampumixis dispar |  | Insects & other |
| Anabolia bimaculata |  | Insects & other |
| Anacaena limbata |  | Insects & other |
| Anacaena signaticollis |  | Insects & other |
| Anacroneuria wipukupa |  | Insects & other |
| Anagapetus aisha | A Caddisfly | Insects & other |
| Anagapetus bernea | A Caddisfly | Insects & other |
| Anagapetus chandleri | A Caddisfly | Insects & other |
| Anagapetus debilis |  | Insects & other |
| Anagapetus hoodi |  | Insects & other |
| Anas acuta | Northern Pintail | Birds |
| Anas americana | American Wigeon | Birds |
| Anas clypeata | Northern Shoveler | Birds |
| Anas crecca | Green-winged Teal | Birds |
| Anas cyanoptera | Cinnamon Teal | Birds |
| Anas discors | Blue-winged Teal | Birds |
| Anas platyrhynchos | Mallard | Birds |
| Anas strepera | Gadwall | Birds |
| Anax junius | Common Green Darner | Insects & other |
| Anax walsinghami | Giant Green Darner | Insects & other |
| Anaxyrus boreas boreas | Boreal Toad | Herps |
| Anaxyrus boreas halophilus | California Toad | Herps |
| Anaxyrus californicus | Arroyo Toad | Herps |
| Anaxyrus canorus | Yosemite Toad | Herps |
| Anaxyrus cognatus | Great Plains Toad | Herps |
| Anaxyrus exsul | Black Toad | Herps |
| Anaxyrus punctatus | Red-spotted Toad | Herps |
| Anaxyrus woodhousii woodhousii | Rocky Mountain Toad | Herps |
| Anchycteis velutina |  | Insects & other |
| Anemopsis californica | Yerba Mansa | Plants |
| Anodonta californiensis | California Floater | Mollusks |
| Anodonta dejecta | Woebegone Floater | Mollusks |
| Anodonta oregonensis | Oregon Floater | Mollusks |
| Anopheles franciscanus |  | Insects & other |
| Anopheles freeborni |  | Insects & other |
| Anopheles hermsi |  | Insects & other |
| Anopheles judithae |  | Insects & other |
| Anopheles occidentalis |  | Insects & other |
| Anopheles punctipennis |  | Insects & other |
| Anser albifrons | Greater White-fronted Goose | Birds |
| Anser albifrons elgasi | Tule White-fronted Goose | Birds |
| Anthopotamus verticis | Walker's Tusked Sprawler | Insects & other |
| Antocha monticola |  | Insects & other |
| Apanisagrion lais |  | Insects & other |
| Apatania arizona |  | Insects & other |
| Apatania chasica |  | Insects & other |
| Apatania sorex | A Caddisfly | Insects & other |
| Apatania tavala | Cascades Apatanian Caddisfly | Insects & other |
| Apedilum elachistum |  | Insects & other |
| Apedilum subcinctum |  | Insects & other |
| Aphodius alternatus |  | Insects & other |
| Apobaetis etowah | A Mayfly | Insects & other |
| Aponogeton distachyos | NA | Plants |
| Apsectrotanypus florens |  | Insects & other |
| Apteraliplus parvulus |  | Insects & other |
| Aquarius amplus arizonensis |  | Insects & other |
| Aquarius remigis |  | Insects & other |
| Aquilegia eximia | Van Houtte's Columbine | Plants |
| Aquilegia shockleyi | NA | Plants |
| Araeopidius monochus |  | Insects & other |
| Archilestes californica | California Spreadwing | Insects & other |
| Archilestes grandis | Great Spreadwing | Insects & other |
| Archoplites interruptus | Sacramento perch | Fishes |
| Arctitalitus sylvaticus |  | Crustaceans |
| Arctocorisa sutilis |  | Insects & other |
| Arctopsyche californica | A Caddisfly | Insects & other |
| Arctopsyche grandis | A Caddisfly | Insects & other |
| Ardea alba | Great Egret | Birds |
| Ardea herodias | Great Blue Heron | Birds |
| Arenaria paludicola | Marsh Sandwort | Plants |
| Argia agrioides | California Dancer | Insects & other |
| Argia alberta | Paiute Dancer | Insects & other |
| Argia emma | Emma's Dancer | Insects & other |
| Argia fumipennis |  | Insects & other |
| Argia hinei | Lavender Dancer | Insects & other |
| Argia immunda | Kiowa Dancer | Insects & other |
| Argia lacrimans |  | Insects & other |
| Argia lugens | Sooty Dancer | Insects & other |
| Argia moesta | Powdered Dancer | Insects & other |
| Argia munda |  | Insects & other |
| Argia nahuana | Aztec Dancer | Insects & other |
| Argia oenea |  | Insects & other |
| Argia pallens |  | Insects & other |
| Argia pima |  | Insects & other |
| Argia plana |  | Insects & other |
| Argia sabino |  | Insects & other |
| Argia sedula | Blue-ringed Dancer | Insects & other |
| Argia tarascana |  | Insects & other |
| Argia tezpi |  | Insects & other |
| Argia tonto |  | Insects & other |
| Argia translata |  | Insects & other |
| Argia vivida | Vivid Dancer | Insects & other |
| Artemia franciscana | San Francisco Brine Shrimp | Crustaceans |
| Artemia monica | Mono Lake Brine Shrimp | Crustaceans |
| Arundo donax | NA | Plants |
| Asarum lemmonii | Lemmon's Wild Ginger | Plants |
| Ascaphus truei | Coastal Tailed Frog | Herps |
| Asioplax edmundsi | A Mayfly | Insects & other |
| Assiminea californica |  | Mollusks |
| Assiminea infima | Badwater Snail | Mollusks |
| Asynarchus aldinus |  | Insects & other |
| Asynarchus cinnamoneus |  | Insects & other |
| Asynarchus montanus |  | Insects & other |
| Asynarchus pacificus |  | Insects & other |
| Atherix pachypus |  | Insects & other |
| Atopsyche sperryi |  | Insects & other |
| Atopsyche tripunctata |  | Insects & other |
| Atractelmis wawona | Wawona Riffle Beetle | Insects & other |
| Attenella attenuata |  | Insects & other |
| Attenella delantala | A Mayfly | Insects & other |
| Attenella margarita | A Mayfly | Insects & other |
| Attenella soquele | A Mayfly | Insects & other |
| Augyles mundulus |  | Insects & other |
| Axonopsis californica |  | Insects & other |
| Aythya affinis | Lesser Scaup | Birds |
| Aythya americana | Redhead | Birds |
| Aythya collaris | Ring-necked Duck | Birds |
| Aythya marila | Greater Scaup | Birds |
| Aythya valisineria | Canvasback | Birds |
| Azolla filiculoides | NA | Plants |
| Azolla microphylla | Mexican mosquito fern | Plants |
| Baccharis glutinosa | NA | Plants |
| Baccharis salicina |  | Plants |
| Bacopa eisenii | Gila River Water-hyssop | Plants |
| Bacopa monnieri | NA | Plants |
| Bacopa rotundifolia | NA | Plants |
| Baetis adonis | A Mayfly | Insects & other |
| Baetis alius | A Mayfly | Insects & other |
| Baetis bicaudatus | A Mayfly | Insects & other |
| Baetis diablus | A Mayfly | Insects & other |
| Baetis flavistriga | A Mayfly | Insects & other |
| Baetis magnus | A Mayfly | Insects & other |
| Baetis notos | A Mayfly | Insects & other |
| Baetis palisadi | A Mayfly | Insects & other |
| Baetis piscatoris | A Mayfly | Insects & other |
| Baetis tricaudatus | A Mayfly | Insects & other |
| Baetisca lacustris |  | Insects & other |
| Baetodes alleni |  | Insects & other |
| Baetodes arizonensis |  | Insects & other |
| Baetodes bibranchius |  | Insects & other |
| Baetodes edmundsi |  | Insects & other |
| Bandakia fragilis |  | Insects & other |
| Bandakia longipalpis |  | Insects & other |
| Bandakia oregonensis |  | Insects & other |
| Banksiola crotchi | A Caddisfly | Insects & other |
| Batis maritima | Saltwort | Plants |
| Batrachoseps campi | Inyo Mountains Salamander | Herps |
| Baumannella alameda | Alameda Springfly | Insects & other |
| Beckmannia syzigachne | American Sloughgrass | Plants |
| Belostoma bakeri |  | Insects & other |
| Belostoma confusum |  | Insects & other |
| Belostoma flumineum |  | Insects & other |
| Belostoma saratogae | Saratoga Springs Belostoman Bug | Insects & other |
| Belostoma subspinosum |  | Insects & other |
| Bergia texana | Texas Bergia | Plants |
| Berosus fraternus |  | Insects & other |
| Berosus hatchi |  | Insects & other |
| Berosus infuscatus |  | Insects & other |
| Berosus ingeminatus |  | Insects & other |
| Berosus maculosus |  | Insects & other |
| Berosus metalliceps |  | Insects & other |
| Berosus notapeltatus |  | Insects & other |
| Berosus oregonensis |  | Insects & other |
| Berosus punctatissimus |  | Insects & other |
| Berosus sayi |  | Insects & other |
| Berosus stylifera |  | Insects & other |
| Berula erecta | Wild Parsnip | Plants |
| Betula glandulosa | Resin Birch | Plants |
| Bibiocephala grandis |  | Insects & other |
| Bidens cernua | Nodding Beggarticks | Plants |
| Bidens laevis | Smooth Bur-marigold | Plants |
| Bidens tripartita | NA | Plants |
| Bidens vulgata | NA | Plants |
| Bilyjomyia algens |  | Insects & other |
| Biomphalaria havanensis | Ghost Rams-horn | Mollusks |
| Bisancora pastina | Antelope Sallfly | Insects & other |
| Bisancora rutriformis | Scooped Sallfly | Insects & other |
| Bistorta bistortoides |  | Plants |
| Bittacomorpha clavipes |  | Insects & other |
| Bittacomorpha occidentalis |  | Insects & other |
| Bittacomorphella ostenii |  | Insects & other |
| Bittacomorphella pacifica |  | Insects & other |
| Blennosperma bakeri | Baker's Blennosperma | Plants |
| Blepharicera jordani |  | Insects & other |
| Blepharicera kalmiopsis |  | Insects & other |
| Blepharicera micheneri | A Net-winged Midge | Insects & other |
| Blepharicera ostensackeni |  | Insects & other |
| Blepharicera zionensis |  | Insects & other |
| Boehmeria cylindrica | NA | Plants |
| Bolboschoenus fluviatilis |  | Plants |
| Bolboschoenus glaucus | NA | Plants |
| Bolboschoenus maritimus paludosus | NA | Plants |
| Bolboschoenus robustus |  | Plants |
| Bolshecapnia maculata | Spotted Snowfly | Insects & other |
| Boreoclus persimilis |  | Insects & other |
| Boreoclus sinuaticornis |  | Insects & other |
| Boreoheptagyia lurida |  | Insects & other |
| Botaurus lentiginosus | American Bittern | Birds |
| Bowmanasellus sequoiae | Sequoia cave isopod | Crustaceans |
| Brachycentrus americanus | A Caddisfly | Insects & other |
| Brachycentrus echo | A Caddisfly | Insects & other |
| Brachycentrus occidentalis |  | Insects & other |
| Brachymesia furcata | Red-tailed Pennant | Insects & other |
| Brachymesia gravida |  | Insects & other |
| Branchinecta campestris | Pocket Pouch Fairy Shrimp | Crustaceans |
| Branchinecta coloradensis | Colorado Fairy Shrimp | Crustaceans |
| Branchinecta conservatio | Conservancy Fairy Shrimp | Crustaceans |
| Branchinecta cornigera |  | Crustaceans |
| Branchinecta dissimilis | Dissimilar Fairy Shrimp | Crustaceans |
| Branchinecta gigas | Giant Fairy Shrimp | Crustaceans |
| Branchinecta hiberna | Winter Fairy Shrimp | Crustaceans |
| Branchinecta kaibabensis |  | Crustaceans |
| Branchinecta lindahli | Versatile Fairy Shrimp | Crustaceans |
| Branchinecta longiantenna | Longhorn Fairy Shrimp | Crustaceans |
| Branchinecta lynchi | Vernal Pool Fairy Shrimp | Crustaceans |
| Branchinecta mackini | Alkali Fairy Shrimp | Crustaceans |
| Branchinecta mesovallensis | Midvalley Fairy Shrimp | Crustaceans |
| Branchinecta oriena | A Fairy Shrimp | Crustaceans |
| Branchinecta packardi |  | Crustaceans |
| Branchinecta sandiegonensis | San Diego Fairy Shrimp | Crustaceans |
| Brasenia schreberi | Watershield | Plants |
| Brechmorhoga mendax | Pale-faced Clubskimmer | Insects & other |
| Brechmorhoga pertinax |  | Insects & other |
| Brillia flavifrons |  | Insects & other |
| Brillia laculata |  | Insects & other |
| Brillia parva |  | Insects & other |
| Brillia retifinis |  | Insects & other |
| Brodiaea nana |  | Plants |
| Brodiaea orcuttii | Orcutt's Brodiaea | Plants |
| Brodiaea pallida | Chinese Camp Brodiaea | Plants |
| Brundiniella eumorpha |  | Insects & other |
| Brychius hornii |  | Insects & other |
| Brychius pacificus |  | Insects & other |
| Bucephala albeola | Bufflehead | Birds |
| Bucephala clangula | Common Goldeneye | Birds |
| Buenoa arida |  | Insects & other |
| Buenoa arizonis |  | Insects & other |
| Buenoa hungerfordi |  | Insects & other |
| Buenoa margaritacea |  | Insects & other |
| Buenoa omani |  | Insects & other |
| Buenoa scimitra |  | Insects & other |
| Buenoa uhleri |  | Insects & other |
| Butorides virescens | Green Heron | Birds |
| Caecidotea sequoiae | An Isopod | Crustaceans |
| Caecidotea tomalensis | Tomales Isopod | Crustaceans |
| Caenis amica | A Mayfly | Insects & other |
| Caenis bajaensis | A Mayfly | Insects & other |
| Caenis latipennis | A Mayfly | Insects & other |
| Caenis punctata | A Mayfly | Insects & other |
| Caenis youngi | A Mayfly | Insects & other |
| Caladomyia pistra |  | Insects & other |
| Calamagrostis nutkaensis | Pacific Small-reedgrass | Plants |
| Calasellus californicus | An Isopod | Crustaceans |
| Calasellus longus | An Isopod | Crustaceans |
| Calidris alpina | Dunlin | Birds |
| Calidris mauri | Western Sandpiper | Birds |
| Calidris minutilla | Least Sandpiper | Birds |
| Calileuctra dobryi | Elsmere Needlefly | Insects & other |
| Calileuctra ephemera | Napa Needlefly | Insects & other |
| Calineuria californica | Western Stone | Insects & other |
| Callibaetis californicus | A Mayfly | Insects & other |
| Callibaetis ferrugineus | A Mayfly | Insects & other |
| Callibaetis fluctuans | A Mayfly | Insects & other |
| Callibaetis montanus |  | Insects & other |
| Callibaetis pallidus | A Mayfly | Insects & other |
| Callibaetis pictus | A Mayfly | Insects & other |
| Callicorixa audeni |  | Insects & other |
| Callicorixa scudderi |  | Insects & other |
| Callicorixa vulnerata |  | Insects & other |
| Calliperla luctuosa | Coast Stripetail | Insects & other |
| Callitriche fassettii | NA | Plants |
| Callitriche heterophylla bolanderi | Large Water-starwort | Plants |
| Callitriche heterophylla heterophylla | Northern Water-starwort | Plants |
| Callitriche longipedunculata | Longstock Water-starwort | Plants |
| Callitriche marginata | Winged Water-starwort | Plants |
| Callitriche palustris | Vernal Water-starwort | Plants |
| Callitriche trochlearis | Waste-water Water-starwort | Plants |
| Calochortus uniflorus | Shortstem Mariposa Lily | Plants |
| Calopteryx aequabilis | River Jewelwing | Insects & other |
| Caltha leptosepala | Slender-sepal Marsh-marigold | Plants |
| Caltha palustris | NA | Plants |
| Camelobaetidius kickapoo |  | Insects & other |
| Camelobaetidius maidu | Maidu Mayfly | Insects & other |
| Camelobaetidius mexicanus |  | Insects & other |
| Camelobaetidius musseri |  | Insects & other |
| Camelobaetidius warreni | A Mayfly | Insects & other |
| Campanula californica | Swamp Harebell | Plants |
| Capnia barberi | Plumas Snowfly | Insects & other |
| Capnia californica | California Snowfly | Insects & other |
| Capnia caryi |  | Insects & other |
| Capnia confusa |  | Insects & other |
| Capnia coyote | Coyote Snowfly | Insects & other |
| Capnia decepta |  | Insects & other |
| Capnia elongata | Caascades Snowfly | Insects & other |
| Capnia erecta |  | Insects & other |
| Capnia excavata | Saddleback Snowfly | Insects & other |
| Capnia fialai | Humboldt Snowfly | Insects & other |
| Capnia giulianii | Whitney Snowfly | Insects & other |
| Capnia glabra | Smooth Snowfly | Insects & other |
| Capnia gracilaria | Slender Snowfly | Insects & other |
| Capnia hitchcocki | Arroyo Snowfly | Insects & other |
| Capnia hornigi |  | Insects & other |
| Capnia inyo | Inyo Snowfly | Insects & other |
| Capnia jewetti |  | Insects & other |
| Capnia kersti |  | Insects & other |
| Capnia lacustra | Lake Snowfly | Insects & other |
| Capnia licina |  | Insects & other |
| Capnia lineata | Straight Snowfly | Insects & other |
| Capnia mariposa | Mariposa Snowfly | Insects & other |
| Capnia melia | Northwest Snowfly | Insects & other |
| Capnia mono | Mono Snowfly | Insects & other |
| Capnia nana |  | Insects & other |
| Capnia nedia |  | Insects & other |
| Capnia ophiona | Snakehead Snowfly | Insects & other |
| Capnia oregona |  | Insects & other |
| Capnia palomar | Palomar Snowfly | Insects & other |
| Capnia petila |  | Insects & other |
| Capnia pileata | Birdhead Snowfly | Insects & other |
| Capnia promota | Pacific Snowfly | Insects & other |
| Capnia quadrituberosa | Four-knobbed Snowfly | Insects & other |
| Capnia regilla | Royal Snowfly | Insects & other |
| Capnia saratoga | Saratoga Snowfly | Insects & other |
| Capnia scobina | Rasp Snowfly | Insects & other |
| Capnia sequoia | Sequoia Snowfly | Insects & other |
| Capnia sextuberculata |  | Insects & other |
| Capnia shasta |  | Insects & other |
| Capnia shepardi | Yuba Snowfly | Insects & other |
| Capnia spinulosa | San Gabriel Snowfly | Insects & other |
| Capnia teresa | Bernardino Snowfly | Insects & other |
| Capnia tumida | Swollen Snowfly | Insects & other |
| Capnia uintahi |  | Insects & other |
| Capnia umpqua | Umpqua Snowfly | Insects & other |
| Capnia utahensis | Utah Snowfly | Insects & other |
| Capnia valhalla | Viking Snowfly | Insects & other |
| Capnia ventura | Ventura Snowfly | Insects & other |
| Capnia willametta |  | Insects & other |
| Capnia yosemite | Yosemite Snowfly | Insects & other |
| Capnura anas |  | Insects & other |
| Capnura elevata |  | Insects & other |
| Capnura fibula |  | Insects & other |
| Capnura intermontana |  | Insects & other |
| Capnura venosa |  | Insects & other |
| Capnura wanica |  | Insects & other |
| Cardiocladius platypus |  | Insects & other |
| Carex alma | Sturdy Sedge | Plants |
| Carex amplifolia | Bigleaf Sedge | Plants |
| Carex aquatilis aquatilis | Water Sedge | Plants |
| Carex aquatilis dives | Sitka Sedge | Plants |
| Carex arcta | Northern Clustered Sedge | Plants |
| Carex atherodes | Awned Sedge | Plants |
| Carex aurea | Golden-fruit Sedge | Plants |
| Carex buxbaumii | Buxbaum's Sedge | Plants |
| Carex canescens canescens | Hoary Sedge | Plants |
| Carex comosa | Bristly Sedge | Plants |
| Carex cusickii | Cusick's Sedge | Plants |
| Carex densa | Dense Sedge | Plants |
| Carex diandra | Lesser Panicled Sedge | Plants |
| Carex disperma | Softleaf Sedge | Plants |
| Carex echinata echinata | Little Prickly Sedge | Plants |
| Carex echinata phyllomanica | Star Sedge | Plants |
| Carex exsiccata | Beaked Sedge | Plants |
| Carex feta | Green-sheath Sedge | Plants |
| Carex fissuricola | Cleft Sedge | Plants |
| Carex harfordii | Harford's Sedge | Plants |
| Carex hendersonii | Henderson's Sedge | Plants |
| Carex hirtissima | Fuzzy Sedge | Plants |
| Carex hystericina | Porcupine Sedge | Plants |
| Carex integra | Smooth-beak Sedge | Plants |
| Carex interior | Inland Sedge | Plants |
| Carex jonesii | Jones' Sedge | Plants |
| Carex klamathensis |  | Plants |
| Carex lasiocarpa | Slender Sedge | Plants |
| Carex lemmonii | Lemmon's Sedge | Plants |
| Carex lenticularis | Shore Sedge | Plants |
| Carex leporina |  | Plants |
| Carex leporinella | Sierra Hare Sedge | Plants |
| Carex leptalea | NA | Plants |
| Carex limosa | Mud Sedge | Plants |
| Carex livida | Livid Sedge | Plants |
| Carex longii | NA | Plants |
| Carex luzulina luzulina | Woodrush Sedge | Plants |
| Carex lyngbyei | Lyngbye's Sedge | Plants |
| Carex mertensii | Mertens' Sedge | Plants |
| Carex nebrascensis | Nebraska Sedge | Plants |
| Carex nervina | Sierra Sedge | Plants |
| Carex neurophora | Alpine-nerved Sedge | Plants |
| Carex nigricans | Black Alpine Sedge | Plants |
| Carex nudata | Torrent Sedge | Plants |
| Carex obnupta | Slough Sedge | Plants |
| Carex pellita | Woolly Sedge | Plants |
| Carex praeceptorum | Teacher's Sedge | Plants |
| Carex praticola | Northern Meadow Sedge | Plants |
| Carex saliniformis | Santa Cruz Sedge | Plants |
| Carex sartwelliana | Yosemite Sedge | Plants |
| Carex scabriuscula | Cascade Sedge | Plants |
| Carex schottii | Schott's Sedge | Plants |
| Carex scoparia scoparia | Broom Sedge | Plants |
| Carex scopulorum bracteosa | Holm's Rocky Mountain Sedge | Plants |
| Carex senta | Western Rough Sedge | Plants |
| Carex sheldonii | Sheldon's Sedge | Plants |
| Carex simulata | Copycat Sedge | Plants |
| Carex spectabilis | Northwestern Showy Sedge | Plants |
| Carex stipata stipata | Stalk-grain Sedge | Plants |
| Carex utriculata | Beaked Sedge | Plants |
| Carex vesicaria vesicaria | Inflated Sedge | Plants |
| Carex viridula viridula | Little Green Sedge | Plants |
| Carex vulpinoidea | NA | Plants |
| Cascadia nuttallii | NA | Plants |
| Cascadoperla trictura | Cascades Stripetail | Insects & other |
| Castilleja campestris succulenta | Fleshy Owl's-clover | Plants |
| Castilleja miniata elata | Siskiyou Indian-paintbrush | Plants |
| Castilleja miniata miniata | Greater Red Indian-paintbrush | Plants |
| Castilleja minor minor | Alkali Indian-paintbrush | Plants |
| Castilleja minor spiralis | Large-flower Annual Indian-paintbrush | Plants |
| Castor canadensis | American Beaver | Mammals |
| Catostomus fumeiventris | Owens sucker | Fishes |
| Catostomus latipinnis | Flannelmouth sucker | Fishes |
| Catostomus luxatus | Lost River sucker | Fishes |
| Catostomus microps | Modoc sucker | Fishes |
| Catostomus occidentalis humboldtianus | Humboldt sucker | Fishes |
| Catostomus occidentalis lacusanserinus | Goose Lake sucker | Fishes |
| Catostomus occidentalis mnioltiltus | Monterey sucker | Fishes |
| Catostomus occidentalis occidentalis | Sacramento sucker | Fishes |
| Catostomus platyrhynchus | Lahontan mountain sucker | Fishes |
| Catostomus rimiculus | Klamath smallscale sucker | Fishes |
| Catostomus santaanae | Santa Ana sucker | Fishes |
| Catostomus snyderi | Klamath largescale sucker | Fishes |
| Catostomus tahoensis | Tahoe sucker | Fishes |
| Caudatella columbiella |  | Insects & other |
| Caudatella edmundsi | A Mayfly | Insects & other |
| Caudatella heterocaudata | A Mayfly | Insects & other |
| Caudatella hystrix | A Mayfly | Insects & other |
| Caudatella jacobi | A Mayfly | Insects & other |
| Celina occidentalis |  | Insects & other |
| Cenocorixa andersoni |  | Insects & other |
| Cenocorixa blaisdelli |  | Insects & other |
| Cenocorixa kuiterti | A Water Boatman | Insects & other |
| Cenocorixa utahensis |  | Insects & other |
| Cenocorixa wileyae |  | Insects & other |
| Centroptilum album | A Mayfly | Insects & other |
| Centroptilum asperatum | A Mayfly | Insects & other |
| Centroptilum bifurcatum | A Mayfly | Insects & other |
| Centroptilum conturbatum | A Mayfly | Insects & other |
| Centroptilum elsa |  | Insects & other |
| Centroptilum oreophilum |  | Insects & other |
| Centroptilum selanderorum |  | Insects & other |
| Cephalanthus occidentalis | Common Buttonbush | Plants |
| Ceraclea annulicornis | A Caddisfly | Insects & other |
| Ceraclea latahensis | A Caddisfly | Insects & other |
| Ceraclea maculata | A Caddisfly | Insects & other |
| Ceraclea resurgens |  | Insects & other |
| Ceraclea tarsipunctata | A Caddisfly | Insects & other |
| Ceraclea vertreesi |  | Insects & other |
| Ceratophyllum demersum | Common Hornwort | Plants |
| Chaetarthria bicolor |  | Insects & other |
| Chaetarthria hespera |  | Insects & other |
| Chaetarthria leechi | Leech's Chaetarthrian Water Scavenger Beetle | Insects & other |
| Chaetarthria magna |  | Insects & other |
| Chaetarthria nigrella |  | Insects & other |
| Chaetarthria ochra |  | Insects & other |
| Chaetarthria pallida |  | Insects & other |
| Chaetarthria punctulata |  | Insects & other |
| Chaetarthria pusilla |  | Insects & other |
| Chaetarthria spinata |  | Insects & other |
| Chaetarthria truncata |  | Insects & other |
| Chaetocladius ligni |  | Insects & other |
| Chamaecyparis lawsoniana |  | Plants |
| Chasmatonotus hyalinus |  | Insects & other |
| Chasmatonotus maculipennis |  | Insects & other |
| Chasmatonotus univittatus |  | Insects & other |
| Chasmistes brevirostris | Shortnose sucker | Fishes |
| Chelomideopsis brunsoni |  | Insects & other |
| Chelomideopsis minuta |  | Insects & other |
| Chelomideopsis occidentalis |  | Insects & other |
| Chelomideopsis siskiyouensis |  | Insects & other |
| Chen caerulescens | Snow Goose | Birds |
| Chen rossii | Ross's Goose | Birds |
| Chernokrilus misnomus | Oregon Springfly | Insects & other |
| Chernovskiia orbicus |  | Insects & other |
| Cheumatopsyche analis |  | Insects & other |
| Cheumatopsyche arizonensis | A Caddisfly | Insects & other |
| Cheumatopsyche campyla | A Caddisfly | Insects & other |
| Cheumatopsyche enonis |  | Insects & other |
| Cheumatopsyche gelita |  | Insects & other |
| Cheumatopsyche lasia |  | Insects & other |
| Cheumatopsyche mickeli | A Caddisfly | Insects & other |
| Cheumatopsyche mollala | A Caddisfly | Insects & other |
| Cheumatopsyche pasella |  | Insects & other |
| Cheumatopsyche pinula |  | Insects & other |
| Cheumatopsyche wabasha |  | Insects & other |
| Chimarra adella |  | Insects & other |
| Chimarra angustipennis | A Caddisfly | Insects & other |
| Chimarra butleri | A Caddisfly | Insects & other |
| Chimarra elia | A Caddisfly | Insects & other |
| Chimarra lara |  | Insects & other |
| Chimarra primula |  | Insects & other |
| Chimarra ridleyi |  | Insects & other |
| Chimarra schiza |  | Insects & other |
| Chimarra siva |  | Insects & other |
| Chimarra texana |  | Insects & other |
| Chimarra utahensis | A Caddisfly | Insects & other |
| Chironomus anonymus |  | Insects & other |
| Chironomus anthracinus |  | Insects & other |
| Chironomus atrella |  | Insects & other |
| Chironomus calligraphus |  | Insects & other |
| Chironomus cucini |  | Insects & other |
| Chironomus decorus |  | Insects & other |
| Chironomus frommeri |  | Insects & other |
| Chironomus longipes |  | Insects & other |
| Chironomus maturus |  | Insects & other |
| Chironomus mendax |  | Insects & other |
| Chironomus plumosus |  | Insects & other |
| Chironomus riparius |  | Insects & other |
| Chironomus staegeri |  | Insects & other |
| Chironomus stigmaterus |  | Insects & other |
| Chironomus tuxis |  | Insects & other |
| Chironomus utahensis |  | Insects & other |
| Chironomus whitseli |  | Insects & other |
| Chlidonias niger | Black Tern | Birds |
| Chloropyron maritimum canescens |  | Plants |
| Chloropyron maritimum maritimum |  | Plants |
| Chloropyron maritimum palustre |  | Plants |
| Chloropyron molle hispidum |  | Plants |
| Chloropyron molle molle |  | Plants |
| Chloropyron palmatum | NA | Plants |
| Chloropyron tecopense |  | Plants |
| Choroterpes albiannulata | A Mayfly | Insects & other |
| Choroterpes inornata | A Mayfly | Insects & other |
| Choroterpes terratoma | A Mayfly | Insects & other |
| Chroicocephalus philadelphia | Bonaparte's Gull | Birds |
| Chrysosplenium glechomifolium | Pacific Golden-saxifrage | Plants |
| Chyrandra centralis | A Caddisfly | Insects & other |
| Cicendia quadrangularis | Oregon Microcala | Plants |
| Cicuta douglasii | Western Water-hemlock | Plants |
| Cicuta maculata angustifolia | Spotted Water-hemlock | Plants |
| Cicuta maculata bolanderi | Bolander's Water-hemlock | Plants |
| Cicuta maculata maculata | Spotted Water-hemlock | Plants |
| Cinclus mexicanus | American Dipper | Birds |
| Cinygma dimicki | A Mayfly | Insects & other |
| Cinygma integrum | A Mayfly | Insects & other |
| Cinygma lyriforme | A Mayfly | Insects & other |
| Cinygmula gartrelli | A Mayfly | Insects & other |
| Cinygmula mimus | A Mayfly | Insects & other |
| Cinygmula par | A Mayfly | Insects & other |
| Cinygmula ramaleyi | A Mayfly | Insects & other |
| Cinygmula reticulata | A Mayfly | Insects & other |
| Cinygmula tarda |  | Insects & other |
| Cinygmula tioga | A Mayfly | Insects & other |
| Cinygmula uniformis | A Mayfly | Insects & other |
| Cirsium crassicaule | Slough Thistle | Plants |
| Cirsium douglasii breweri |  | Plants |
| Cirsium douglasii douglasii | Douglas' Thistle | Plants |
| Cirsium fontinale campylon | Mt. Hamilton Thistle | Plants |
| Cirsium fontinale fontinale | Fountain Thistle | Plants |
| Cirsium fontinale obispoense | Chorro Creek Bog Thistle | Plants |
| Cirsium hydrophilum hydrophilum | Suisun Thistle | Plants |
| Cirsium hydrophilum vaseyi | Mt. Tamalpais Thistle | Plants |
| Cirsium scariosum loncholepis |  | Plants |
| Cirsium scariosum robustum |  | Plants |
| Cirsium scariosum scariosum | Drummond's Thistle | Plants |
| Cistothorus palustris clarkae | Clark's Marsh Wren | Birds |
| Cistothorus palustris palustris | Marsh Wren | Birds |
| Claassenia sabulosa | Shortwing Stone | Insects & other |
| Cladium californicum | California Sawgrass | Plants |
| Cladopelma amachaerum |  | Insects & other |
| Cladopelma edwardsi |  | Insects & other |
| Cladopelma forcipis |  | Insects & other |
| Cladopelma viridulum |  | Insects & other |
| Cladotanytarsus marki |  | Insects & other |
| Cladotanytarsus viridiventris |  | Insects & other |
| Cleptelmis addenda |  | Insects & other |
| Climacia californica |  | Insects & other |
| Clinopodium mimuloides | Monkey-flower Savory | Plants |
| Clinotanypus pinguis |  | Insects & other |
| Clistoronia formosa |  | Insects & other |
| Clistoronia maculata |  | Insects & other |
| Clistoronia magnifica | A Caddisfly | Insects & other |
| Cloeodes excogitatus | A Mayfly | Insects & other |
| Cloeodes macrolamellus |  | Insects & other |
| Cloeodes peninsulus |  | Insects & other |
| Clostoeca disjuncta | A Caddisfly | Insects & other |
| Clunio californiensis |  | Insects & other |
| Cnodocentron yavapai |  | Insects & other |
| Coccyzus americanus occidentalis | Western Yellow-billed Cuckoo | Birds |
| Coenagrion resolutum | Taiga Bluet | Insects & other |
| Colligyrus convexus | Canary Duskysnail | Mollusks |
| Colligyrus greggi |  | Mollusks |
| Colymbetes crotchi |  | Insects & other |
| Colymbetes densus |  | Insects & other |
| Colymbetes incognitus |  | Insects & other |
| Colymbetes strigatus |  | Insects & other |
| Comarum palustre | Marsh Cinquefoil | Plants |
| Conchapelopia mera |  | Insects & other |
| Conchapelopia pallens |  | Insects & other |
| Copelatus chevrolati |  | Insects & other |
| Copelatus glyphicus |  | Insects & other |
| Coptotomus longulus longulus |  | Insects & other |
| Coquillettidia peturbans |  | Insects & other |
| Cordulegaster diadema |  | Insects & other |
| Cordulegaster dorsalis | Pacific Spiketail | Insects & other |
| Cordulia shurtleffii | American Emerald | Insects & other |
| Corisella decolor |  | Insects & other |
| Corisella edulis |  | Insects & other |
| Corisella inscripta |  | Insects & other |
| Corisella tarsalis |  | Insects & other |
| Corydalus bidenticulatus |  | Insects & other |
| Corydalus texanus |  | Insects & other |
| Cosumnoperla hypocrena | Cosumnes Stripetail | Insects & other |
| Cosumnoperla sequoia | A Stonefly | Insects & other |
| Cottus aleuticus | Coastrange sculpin | Fishes |
| Cottus asper ssp. 1 | Prickly sculpin | Fishes |
| Cottus asper ssp. 2 | Clear Lake prickly sculpin | Fishes |
| Cottus asperrimus | Rough sculpin | Fishes |
| Cottus beldingi | Paiute sculpin | Fishes |
| Cottus gulosus | Riffle sculpin | Fishes |
| Cottus klamathensis klamathensis | Upper Klamath marbled sculpin | Fishes |
| Cottus klamathensis macrops | Bigeye marbled sculpin | Fishes |
| Cottus klamathensis polyporus | Lower Klamath marbled sculpin | Fishes |
| Cottus perplexus | Reticulate sculpin | Fishes |
| Cottus pitensis | Pit sculpin | Fishes |
| Cotula coronopifolia | NA | Plants |
| Coturnicops noveboracensis | Yellow Rail | Birds |
| Crangonyx richmondensis | Ellis Bog Crangonyctid | Crustaceans |
| Crassula aquatica | Water Pygmyweed | Plants |
| Crassula solieri | NA | Plants |
| Crenitis alticola |  | Insects & other |
| Crenitis dissimilis |  | Insects & other |
| Crenitis malkini |  | Insects & other |
| Crenitis morata |  | Insects & other |
| Crenitis palpalis |  | Insects & other |
| Crenitis paradigma |  | Insects & other |
| Crenitis rufiventris |  | Insects & other |
| Crenitis seriellus |  | Insects & other |
| Crenitis snoqualmie |  | Insects & other |
| Crenophylax sperryi |  | Insects & other |
| Cricotopus annulator |  | Insects & other |
| Cricotopus bicinctus |  | Insects & other |
| Cricotopus blinni |  | Insects & other |
| Cricotopus edurus |  | Insects & other |
| Cricotopus furtivus |  | Insects & other |
| Cricotopus fuscatus |  | Insects & other |
| Cricotopus globistylus |  | Insects & other |
| Cricotopus herrmanni |  | Insects & other |
| Cricotopus infuscatus |  | Insects & other |
| Cricotopus nostocicola |  | Insects & other |
| Cricotopus obscurifuscus |  | Insects & other |
| Cricotopus ornatus |  | Insects & other |
| Cricotopus parafuscatus |  | Insects & other |
| Cricotopus subfuscus |  | Insects & other |
| Cricotopus subletteorum |  | Insects & other |
| Cricotopus sylvestris |  | Insects & other |
| Cricotopus tremulus |  | Insects & other |
| Cricotopus trifascia |  | Insects & other |
| Crypsis vaginiflora | NA | Plants |
| Cryptochia califca | A Caddisfly | Insects & other |
| Cryptochia denningi | Denning's Cryptic Caddisfly | Insects & other |
| Cryptochia excella | Kings Canyon Cryptochian Caddisfly | Insects & other |
| Cryptochia neosa |  | Insects & other |
| Cryptochia pilosa |  | Insects & other |
| Cryptochia shasta | Confusion Caddisfly | Insects & other |
| Cryptochironomus curryi |  | Insects & other |
| Cryptochironomus digitatus |  | Insects & other |
| Cryptochironomus fulvus |  | Insects & other |
| Cryptochironomus ponderosus |  | Insects & other |
| Cryptochironomus psittacinus |  | Insects & other |
| Cryptotendipes ariel |  | Insects & other |
| Cryptotendipes darbyi |  | Insects & other |
| Culex anips |  | Insects & other |
| Culex apicalis |  | Insects & other |
| Culex arizonensis |  | Insects & other |
| Culex boharti |  | Insects & other |
| Culex coronator |  | Insects & other |
| Culex erythrothorax |  | Insects & other |
| Culex interrogator |  | Insects & other |
| Culex pipiens |  | Insects & other |
| Culex quinquefasciatus |  | Insects & other |
| Culex reevesi |  | Insects & other |
| Culex restuans |  | Insects & other |
| Culex salinarius |  | Insects & other |
| Culex stigmatosoma |  | Insects & other |
| Culex tarsalis |  | Insects & other |
| Culex territans |  | Insects & other |
| Culex thriambus |  | Insects & other |
| Culiseta impatiens |  | Insects & other |
| Culiseta incidens |  | Insects & other |
| Culiseta inornata |  | Insects & other |
| Culiseta minnesotae |  | Insects & other |
| Culiseta morsitans |  | Insects & other |
| Culiseta particeps |  | Insects & other |
| Culoptila cantha |  | Insects & other |
| Culoptila kimminsi |  | Insects & other |
| Culoptila moselyi |  | Insects & other |
| Culoptila thoracica |  | Insects & other |
| Cultus aestivalis |  | Insects & other |
| Cultus pilatus |  | Insects & other |
| Cultus tostonus | Toston Springfly | Insects & other |
| Curicta pronotata |  | Insects & other |
| Cybister ellipticus |  | Insects & other |
| Cybister explanatus |  | Insects & other |
| Cyclothyas siskiyouensis |  | Insects & other |
| Cygnus buccinator | Trumpeter Swan | Birds |
| Cygnus columbianus | Tundra Swan | Birds |
| Cylloepus abnormis |  | Insects & other |
| Cylloepus parkeri |  | Insects & other |
| Cymbiodyta arizonica |  | Insects & other |
| Cymbiodyta columbiana |  | Insects & other |
| Cymbiodyta dorsalis |  | Insects & other |
| Cymbiodyta fraterculus |  | Insects & other |
| Cymbiodyta howdeni |  | Insects & other |
| Cymbiodyta imbellis |  | Insects & other |
| Cymbiodyta leechi |  | Insects & other |
| Cymbiodyta minima |  | Insects & other |
| Cymbiodyta occidentalis |  | Insects & other |
| Cymbiodyta pacifica |  | Insects & other |
| Cymbiodyta pseudopacifica |  | Insects & other |
| Cymbiodyta puella |  | Insects & other |
| Cymbiodyta punctatostriata |  | Insects & other |
| Cymbiodyta seriata |  | Insects & other |
| Cyperus acuminatus | Short-point Flatsedge | Plants |
| Cyperus bipartitus | Shining Flatsedge | Plants |
| Cyperus erythrorhizos | Red-root Flatsedge | Plants |
| Cyperus flavescens | NA | Plants |
| Cyperus fuscus | NA | Plants |
| Cyperus involucratus | NA | Plants |
| Cyperus iria | NA | Plants |
| Cyperus squarrosus | Awned Cyperus | Plants |
| Cyphomella gibbera |  | Insects & other |
| Cyphon arcuatus |  | Insects & other |
| Cyphon brevicollis |  | Insects & other |
| Cyphon exiguus |  | Insects & other |
| Cyphon johni |  | Insects & other |
| Cyphon spinulosus |  | Insects & other |
| Cyphon variabilis |  | Insects & other |
| Cyprinodon macularius | Desert pupfish | Fishes |
| Cyprinodon nevadensis amargosae | Amargosa River pupfish | Fishes |
| Cyprinodon nevadensis calidae | Tecopa Pupfish | Fishes |
| Cyprinodon nevadensis nevadensis | Saratoga Springs pupfish | Fishes |
| Cyprinodon nevadensis shoshone | Shoshone pupfish | Fishes |
| Cyprinodon radiosus | Owens pupfish | Fishes |
| Cyprinodon salinus milleri | Cottonball Marsh pupfish | Fishes |
| Cyprinodon salinus salinus | Salt Creek pupfish | Fishes |
| Cypripedium californicum | California Lady's-slipper | Plants |
| Cypseloides niger | Black Swift | Birds |
| Cyzicus californicus | California Clam Shrimp | Crustaceans |
| Cyzicus elongatus | Elongate Clam Shrimp | Crustaceans |
| Cyzicus mexicanus | Mexican Clam Shrimp | Crustaceans |
| Cyzicus setosa | Bristletail Clam Shrimp | Crustaceans |
| Damasonium californicum |  | Plants |
| Darlingtonia californica | California Pitcherplant | Plants |
| Darmera peltata | Umbrella Plant | Plants |
| Datisca glomerata | Durango Root | Plants |
| Delphinium uliginosum | Swamp Larkspur | Plants |
| Deltamysis homquistae |  | Crustaceans |
| Demeijerea brachialis |  | Insects & other |
| Dendrocygna bicolor | Fulvous Whistling-Duck | Birds |
| Derotanypus aclines |  | Insects & other |
| Desmona bethula | Amphibious Caddisfly | Insects & other |
| Desmona mono | A Caddisfly | Insects & other |
| Desmopachria dispersa |  | Insects & other |
| Desmopachria latissima |  | Insects & other |
| Desmopachria mexicana |  | Insects & other |
| Desmopachria portmanni |  | Insects & other |
| Despaxia augusta | Smooth Needleflyl | Insects & other |
| Deuterophlebia coloradensis |  | Insects & other |
| Deuterophlebia inyoensis |  | Insects & other |
| Deuterophlebia nielsoni |  | Insects & other |
| Deuterophlebia personata |  | Insects & other |
| Deuterophlebia shasta | A Mountain Midge | Insects & other |
| Diamesa aberrata |  | Insects & other |
| Diamesa ancysta |  | Insects & other |
| Diamesa chorea |  | Insects & other |
| Diamesa davisi |  | Insects & other |
| Diamesa haydaki |  | Insects & other |
| Diamesa heteropus |  | Insects & other |
| Diamesa japonica |  | Insects & other |
| Diamesa sonorae |  | Insects & other |
| Diamesa spinacies |  | Insects & other |
| Dicamptodon ensatus | California Giant Salamander | Herps |
| Dicamptodon tenebrosus | Pacific Giant Salamander | Herps |
| Dicosmoecus atripes | A Caddisfly | Insects & other |
| Dicosmoecus gilvipes | A Caddisfly | Insects & other |
| Dicosmoecus pallicornis | A Caddisfly | Insects & other |
| Dicrotendipes adnilus |  | Insects & other |
| Dicrotendipes aethiops |  | Insects & other |
| Dicrotendipes californicus |  | Insects & other |
| Dicrotendipes crypticus |  | Insects & other |
| Dicrotendipes fumidus |  | Insects & other |
| Dicrotendipes milleri |  | Insects & other |
| Dicrotendipes modestus |  | Insects & other |
| Dicrotendipes nervosus |  | Insects & other |
| Dicrotendipes tritomus |  | Insects & other |
| Dineutus solitarius |  | Insects & other |
| Dineutus sublineatus |  | Insects & other |
| Diphetor hageni | Hagen's Small Minnow Mayfly | Insects & other |
| Diplectrona californica | California Diplectronan Caddisfly | Insects & other |
| Distichlis littoralis | NA | Plants |
| Diura knowltoni | Nearctic Springfly | Insects & other |
| Doddsia occidentalis | Western Willowfly | Insects & other |
| Doithrix barberi |  | Insects & other |
| Doithrix ensifer |  | Insects & other |
| Dolophilodes aequalis | A Caddisfly | Insects & other |
| Dolophilodes andora | A Caddisfly | Insects & other |
| Dolophilodes dorcus | A Caddisfly | Insects & other |
| Dolophilodes novusamericanus | A Caddisfly | Insects & other |
| Dolophilodes pallidipes | A Caddisfly | Insects & other |
| Doroneuria baumanni | Cascades Stone | Insects & other |
| Downingia bacigalupii | Bacigalup's Downingia | Plants |
| Downingia bella | Hoover's Downingia | Plants |
| Downingia bicornuta | NA | Plants |
| Downingia concolor | NA | Plants |
| Downingia cuspidata | Toothed Calicoflower | Plants |
| Downingia elegans | NA | Plants |
| Downingia insignis | Parti-color Downingia | Plants |
| Downingia laeta | Great Basin Downingia | Plants |
| Downingia montana | Sierra Downingia | Plants |
| Downingia ornatissima | NA | Plants |
| Downingia pulchella | Flat-face Downingia | Plants |
| Downingia pulcherrima |  | Plants |
| Downingia pusilla | Dwarf Downingia | Plants |
| Downingia willamettensis |  | Plants |
| Downingia yina | NA | Plants |
| Drosera anglica | English Sundew | Plants |
| Drosera rotundifolia | NA | Plants |
| Drunella coloradensis | A Mayfly | Insects & other |
| Drunella doddsii | A Mayfly | Insects & other |
| Drunella flavilinea | A Mayfly | Insects & other |
| Drunella grandis | A Mayfly | Insects & other |
| Drunella pelosa | A Mayfly | Insects & other |
| Drunella spinifera | A Mayfly | Insects & other |
| Drymocallis cuneifolia ewanii |  | Plants |
| Dryops arizonensis |  | Insects & other |
| Dubiraphia brunnescens | Brownish Dubiraphian Riffle Beetle | Insects & other |
| Dubiraphia giulianii | Giuliani's Dubiraphian Riffle Beetle | Insects & other |
| Dulichium arundinaceum | NA | Plants |
| Dumontia oregonensis | A Water Flea | Crustaceans |
| Dysmicohermes disjunctus |  | Insects & other |
| Dysmicohermes ingens |  | Insects & other |
| Dythemis fugax |  | Insects & other |
| Dythemis nigrescens |  | Insects & other |
| Dythemis velox |  | Insects & other |
| Dytiscus cordieri |  | Insects & other |
| Dytiscus dauricus |  | Insects & other |
| Dytiscus habilis |  | Insects & other |
| Dytiscus hatchi |  | Insects & other |
| Dytiscus hybridus |  | Insects & other |
| Dytiscus marginicollis |  | Insects & other |
| Ecclisocosmoecus scylla |  | Insects & other |
| Ecclisomyia bilera | King's Creek Ecclisomyian Caddisfly | Insects & other |
| Ecclisomyia conspersa | A Caddisfly | Insects & other |
| Ecclisomyia maculosa | A Caddisfly | Insects & other |
| Ecdyonurus criddlei | A Mayfly | Insects & other |
| Ecdyonurus simplicoides |  | Insects & other |
| Echinochloa oryzoides | NA | Plants |
| Echinodorus berteroi | Upright Burhead | Plants |
| Edmundsius agilis | A Mayfly | Insects & other |
| Egretta thula | Snowy Egret | Birds |
| Elatine brachysperma | Shortseed Waterwort | Plants |
| Elatine californica | California Waterwort | Plants |
| Elatine heterandra | Mosquito Waterwort | Plants |
| Elatine rubella | Southwestern Waterwort | Plants |
| Eleocharis acicularis acicularis | Least Spikerush | Plants |
| Eleocharis acicularis gracilescens | Least Spikerush | Plants |
| Eleocharis acicularis occidentalis |  | Plants |
| Eleocharis atropurpurea | Purple Spikerush | Plants |
| Eleocharis bella | Delicate Spikerush | Plants |
| Eleocharis bernardina |  | Plants |
| Eleocharis bolanderi | Bolander's Spikerush | Plants |
| Eleocharis coloradoensis |  | Plants |
| Eleocharis decumbens | Decumbent Spikerush | Plants |
| Eleocharis engelmannii detonsa |  | Plants |
| Eleocharis engelmannii engelmannii | Engelmann's Spikerush | Plants |
| Eleocharis flavescens flavescens | Pale Spikerush | Plants |
| Eleocharis geniculata | Capitate Spikerush | Plants |
| Eleocharis macrostachya | Creeping Spikerush | Plants |
| Eleocharis montevidensis | Sand Spikerush | Plants |
| Eleocharis obtusa | Blunt Spikerush | Plants |
| Eleocharis ovata |  | Plants |
| Eleocharis palustris | Creeping Spikerush | Plants |
| Eleocharis parishii | Parish's Spikerush | Plants |
| Eleocharis parvula | Small Spikerush | Plants |
| Eleocharis quadrangulata | NA | Plants |
| Eleocharis quinqueflora | Few-flower Spikerush | Plants |
| Eleocharis radicans | Rooted Spikerush | Plants |
| Eleocharis rostellata | Beaked Spikerush | Plants |
| Eleocharis suksdorfiana | NA | Plants |
| Eleocharis torticulmis | Twisted Spikerush | Plants |
| Elodea bifoliata | NA | Plants |
| Elodea canadensis | Broad Waterweed | Plants |
| Elodea nuttallii | Nuttall's Waterweed | Plants |
| Elodes angusta |  | Insects & other |
| Elodes apicalis |  | Insects & other |
| Elodes aquatica |  | Insects & other |
| Elodes emarginata |  | Insects & other |
| Elodes impressa |  | Insects & other |
| Elodes nunenmacheri |  | Insects & other |
| Empidonax traillii | Willow Flycatcher | Birds |
| Empidonax traillii adastus | A Willow Flycatcher | Birds |
| Empidonax traillii brewsteri | Willow Flycatcher | Birds |
| Empidonax traillii extimus | Southwestern Willow Flycatcher | Birds |
| Enallagma anna | River Bluet | Insects & other |
| Enallagma basidens | Double-striped Bluet | Insects & other |
| Enallagma boreale | Boreal Bluet | Insects & other |
| Enallagma carunculatum | Tule Bluet | Insects & other |
| Enallagma civile | Familiar Bluet | Insects & other |
| Enallagma clausum | Alkali Bluet | Insects & other |
| Enallagma cyathigerum |  | Insects & other |
| Enallagma praevarum | Arroyo Bluet | Insects & other |
| Enallagma semicirculare |  | Insects & other |
| Endochironomus nigricans |  | Insects & other |
| Endotribelos hesperium |  | Insects & other |
| Enochrus aridus |  | Insects & other |
| Enochrus californicus |  | Insects & other |
| Enochrus carinatus |  | Insects & other |
| Enochrus cristatus |  | Insects & other |
| Enochrus cuspidatus |  | Insects & other |
| Enochrus diffusus |  | Insects & other |
| Enochrus fimbriatus |  | Insects & other |
| Enochrus hamiltoni |  | Insects & other |
| Enochrus ochraceus |  | Insects & other |
| Enochrus piceus |  | Insects & other |
| Enochrus pygmaeus |  | Insects & other |
| Entosphenus folletti | Northern California brook lamprey | Fishes |
| Entosphenus similis | Klamath River lamprey | Fishes |
| Entosphenus tridentata ssp. 1 | Pacific lamprey | Fishes |
| Entosphenus tridentata ssp. 2 | Goose Lake lamprey | Fishes |
| Eobrachycentrus gelidae |  | Insects & other |
| Eocosmoecus frontalis |  | Insects & other |
| Eocyzicus digueti | Straightbacked Clam Shrimp | Crustaceans |
| Epeorus albertae | A Mayfly | Insects & other |
| Epeorus deceptivus | A Mayfly | Insects & other |
| Epeorus dulciana | A Mayfly | Insects & other |
| Epeorus grandis | A Mayfly | Insects & other |
| Epeorus hesperus | A Mayfly | Insects & other |
| Epeorus lagunitas | A Mayfly | Insects & other |
| Epeorus longimanus | A Mayfly | Insects & other |
| Epeorus margarita | A Mayfly | Insects & other |
| Epeorus permagnus |  | Insects & other |
| Ephemera simulans |  | Insects & other |
| Ephemerella alleni |  | Insects & other |
| Ephemerella aurivillii | A Mayfly | Insects & other |
| Ephemerella dorothea dorothea | A Mayfly | Insects & other |
| Ephemerella excrucians | A Mayfly | Insects & other |
| Ephemerella maculata | A Mayfly | Insects & other |
| Ephemerella tibialis | A Mayfly | Insects & other |
| Ephemerella velmae | A Mayfly | Insects & other |
| Ephemerella verruca |  | Insects & other |
| Ephoron album | A Mayfly | Insects & other |
| Epilobium campestre | NA | Plants |
| Epilobium cleistogamum | Cleistogamous Spike-primrose | Plants |
| Epilobium hallianum |  | Plants |
| Epilobium oreganum | Oregon Willowherb | Plants |
| Epilobium oregonense | Oregon Willow-herb | Plants |
| Epilobium palustre | Marsh Willowherb | Plants |
| Epipactis gigantea | Giant Helleborine | Plants |
| Epitheca canis | Beaverpond Baskettail | Insects & other |
| Epitheca spinigera | Spiny Baskettail | Insects & other |
| Equisetum palustre | NA | Plants |
| Eragrostis hypnoides | Teal Lovegrass | Plants |
| Erebaxonopsis nearctica |  | Insects & other |
| Eremopyrgus eganensis |  | Mollusks |
| Eretes sticticus |  | Insects & other |
| Eretmoptera browni |  | Insects & other |
| Erigeron coulteri | Coulter's Fleabane | Plants |
| Eriophorum crinigerum | Fringed Cotton-grass | Plants |
| Eriophorum gracile gracile | Slender Cotton-grass | Plants |
| Erpetogomphus compositus | White-belted Ringtail | Insects & other |
| Erpetogomphus crotalinus |  | Insects & other |
| Erpetogomphus designatus |  | Insects & other |
| Erpetogomphus lampropeltis lampropeltis | Serpent Ringtail | Insects & other |
| Eryngium alismifolium | Inland Coyote-thistle | Plants |
| Eryngium aristulatum aristulatum | California Eryngo | Plants |
| Eryngium aristulatum hooveri | Hoover's Coyote-thistle | Plants |
| Eryngium aristulatum parishii | San Diego Button Celery | Plants |
| Eryngium articulatum | Jointed Coyote-thistle | Plants |
| Eryngium castrense | Great Valley Eryngo | Plants |
| Eryngium constancei | Loch Lomond Button-celery | Plants |
| Eryngium jepsonii | NA | Plants |
| Eryngium mathiasiae | Mathias' Coyote-thistle | Plants |
| Eryngium pinnatisectum | Tuolumne Coyote-thistle | Plants |
| Eryngium racemosum | Delta Coyote-thistle | Plants |
| Eryngium spinosepalum | Spiny Sepaled Coyote-thistle | Plants |
| Eryngium vaseyi vallicola |  | Plants |
| Eryngium vaseyi vaseyi | Vasey's Coyote-thistle | Plants |
| Erythemis collocata | Western Pondhawk | Insects & other |
| Erythemis simplicicollis |  | Insects & other |
| Erythemis vesiculosa |  | Insects & other |
| Erythrodiplax basifusca |  | Insects & other |
| Erythrodiplax funerea |  | Insects & other |
| Eubranchipus bundyi | Knobbedlip Fairy Shrimp | Crustaceans |
| Eubranchipus oregonus | Oregon Fairy Shrimp | Crustaceans |
| Eubranchipus serratus | Ethologist Fairy Shrimp | Crustaceans |
| Eubrianax edwardsii |  | Insects & other |
| Eucapnopsis brevicauda | Shorttailed Snowfly | Insects & other |
| Eucorethra underwoodi |  | Insects & other |
| Eucyclogobius newberryi | Tidewater goby | Fishes |
| Eukiefferiella claripennis |  | Insects & other |
| Eukiefferiella coerulescens |  | Insects & other |
| Eukiefferiella cyanea |  | Insects & other |
| Eukiefferiella devonica |  | Insects & other |
| Eukiefferiella ilkleyensis |  | Insects & other |
| Eulimnadia diversa | Diversity Clam Shrimp | Crustaceans |
| Eulimnadia texana | Texan Clam Shrimp | Crustaceans |
| Eulimnichus analis |  | Insects & other |
| Eulimnichus californicus |  | Insects & other |
| Eulimnichus evanescens |  | Insects & other |
| Eulimnichus montanus |  | Insects & other |
| Eulimnichus perpolitus |  | Insects & other |
| Euphorbia hooveri | NA | Plants |
| Euryhapsis annuliventris |  | Insects & other |
| Euryhapsis illoba |  | Insects & other |
| Eurylophella lodi | A Mayfly | Insects & other |
| Eustoma exaltatum | NA | Plants |
| Euthamia occidentalis | Western Fragrant Goldenrod | Plants |
| Exopalaemon carinicauda |  | Crustaceans |
| Fallceon eatoni | A Mayfly | Insects & other |
| Fallceon quilleri | A Mayfly | Insects & other |
| Fallceon sonora | A Mayfly | Insects & other |
| Fallceon thermophilos | A Mayfly | Insects & other |
| Farula davisi | Green Springs Mountain Farulan Caddisfly | Insects & other |
| Farula geyseri | A Farulan Caddisfly | Insects & other |
| Farula honeyi | A Farulan Caddisfly | Insects & other |
| Farula jewetti |  | Insects & other |
| Farula malkini |  | Insects & other |
| Farula moweri | A Caddisfly | Insects & other |
| Farula petersoni | A Farulan Caddisfly | Insects & other |
| Farula praelonga | Long-tailed Caddisfly | Insects & other |
| Farula raineri |  | Insects & other |
| Farula reapiri |  | Insects & other |
| Farula wigginsi |  | Insects & other |
| Ferrissia fragilis | Fragile Ancylid | Mollusks |
| Ferrissia rivularis | Creeping Ancylid | Mollusks |
| Ferrissia walkeri | Cloche Ancylid | Mollusks |
| Ficopotamus enigmaticus |  | Insects & other |
| Fimbristylis autumnalis | NA | Plants |
| Fimbristylis thermalis | Hot Springs Fimbry | Plants |
| Floerkea proserpinacoides | False Mermaidweed | Plants |
| Fluminicola ahjumawi | Ahjumawi pebblesnail | Mollusks |
| Fluminicola anserinus | Goose Valley pebblesnail | Mollusks |
| Fluminicola caballensis | Horse Creek pebblesnail | Mollusks |
| Fluminicola erosus | Smokey Charley pebblesnail | Mollusks |
| Fluminicola favillaceus | Ash Valley pebblesnail | Mollusks |
| Fluminicola fremonti | Fremont pebblesnail | Mollusks |
| Fluminicola lunsfordensis | Lunsford pebblesnail | Mollusks |
| Fluminicola modoci | Modoc Pebblesnail | Mollusks |
| Fluminicola multifarius | Shasta pebblesnail | Mollusks |
| Fluminicola neritoides | Willow Creek pebblesnail | Mollusks |
| Fluminicola potemicus | Potem Creek pebblesnail | Mollusks |
| Fluminicola scopulinus | Castle Creek pebblesnail | Mollusks |
| Fluminicola seminalis | Nugget Pebblesnail | Mollusks |
| Fluminicola turbiniformis | Turban Pebblesnail | Mollusks |
| Fluminicola umbilicatus | Hat Creek pebblesnail | Mollusks |
| Fluminicola warnerensis | Warner pebblesnail | Mollusks |
| Frankenia palmeri | Palmer's Frankenia | Plants |
| Frisonia picticeps | Painted Springfly | Insects & other |
| Fulica americana | American Coot | Birds |
| Fundulus parvipinnis | California killifish | Fishes |
| Galba bulimoides | Prairie Fossaria | Mollusks |
| Galba cubensis | Carib Fossaria | Mollusks |
| Galba modicella | Rock Fossaria | Mollusks |
| Galba obrussa | Golden Fossaria | Mollusks |
| Galba perplexa | A Freshwater Snail | Mollusks |
| Galba sonomaensis | Sonoma Fossaria | Mollusks |
| Galba techella | A Freshwater Snail | Mollusks |
| Galium trifidum | Small Bedstraw | Plants |
| Gallinago delicata | Wilson's Snipe | Birds |
| Gallinula chloropus | Common Moorhen | Birds |
| Gammarus lacustris |  | Crustaceans |
| Gasterosteus aculeatus aculeatus | Coastal threespine stickleback | Fishes |
| Gasterosteus aculeatus microcephalus | Inland threespine stickleback | Fishes |
| Gasterosteus aculeatus ssp. 1 | Shay Creek stickleback | Fishes |
| Gasterosteus aculeatus williamsoni | Unarmored threespine stickleback | Fishes |
| Gelastocoris oculatus |  | Insects & other |
| Gelastocoris rotundatus |  | Insects & other |
| Gelochelidon nilotica vanrossemi | Gull-billed Tern | Birds |
| Gentiana calycosa | Explorer's Gentian | Plants |
| Gentiana sceptrum | Pacific Gentian | Plants |
| Gentiana setigera | Elegant Gentian | Plants |
| Gentianella amarella acuta | Autumn Dwarf Gentian | Plants |
| Gentianopsis holopetala | Sierra Gentian | Plants |
| Gentianopsis simplex | One-flower Gentian | Plants |
| Georissus californicus |  | Insects & other |
| Georthocladius platystylus |  | Insects & other |
| Georthocladius wirthi |  | Insects & other |
| Geothelpusa dehaani |  | Crustaceans |
| Geothlypis trichas sinuosa | Saltmarsh Common Yellowthroat | Birds |
| Geothlypis trichas trichas | Common Yellowthroat | Birds |
| Gerris comatus |  | Insects & other |
| Gerris gillettei |  | Insects & other |
| Gerris incognitis |  | Insects & other |
| Gerris incurvatus |  | Insects & other |
| Gerris insperatus |  | Insects & other |
| Gigantodax adleri |  | Insects & other |
| Gila coerulea | Blue chub | Fishes |
| Gila crassicauda | Thicktail Chub | Fishes |
| Gila elegans | Bonytail | Fishes |
| Gila orcutti | Arroyo chub | Fishes |
| Glinus radiatus | NA | Plants |
| Glossosoma alascense | A Caddisfly | Insects & other |
| Glossosoma bruna | A Caddisfly | Insects & other |
| Glossosoma califica | A Caddisfly | Insects & other |
| Glossosoma excitum |  | Insects & other |
| Glossosoma mereca | A Caddisfly | Insects & other |
| Glossosoma montanum |  | Insects & other |
| Glossosoma oregonense | A Caddisfly | Insects & other |
| Glossosoma penitum | A Caddisfly | Insects & other |
| Glossosoma pternum | A Caddisfly | Insects & other |
| Glossosoma pyroxum |  | Insects & other |
| Glossosoma schuhi |  | Insects & other |
| Glossosoma sequoia | A Caddisfly | Insects & other |
| Glossosoma traviatum |  | Insects & other |
| Glossosoma velonum |  | Insects & other |
| Glossosoma ventrale |  | Insects & other |
| Glossosoma verdonum | A Caddisfly | Insects & other |
| Glossosoma wenatchee |  | Insects & other |
| Glyceria borealis | Small Floating Mannagrass | Plants |
| Glyceria elata | Tall Mannagrass | Plants |
| Glyceria fluitans | NA | Plants |
| Glyceria grandis | American Mannagrass | Plants |
| Glyceria leptostachya | Slim-head Mannagrass | Plants |
| Glyceria striata var. stricta | Fowl Mannagrass | Plants |
| Glyphopsyche irrorata | A Caddisfly | Insects & other |
| Glyptotendipes barbipes |  | Insects & other |
| Glyptotendipes lobiferus |  | Insects & other |
| Glyptotendipes paripes |  | Insects & other |
| Gnorimosphaeroma insulare | An Isopod | Crustaceans |
| Gnorimosphaeroma noblei | An Isopod | Crustaceans |
| Goeldichironomus amazonicus |  | Insects & other |
| Goeldichironomus holoprasinus |  | Insects & other |
| Goera archaon | A Caddisfly | Insects & other |
| Goeracea genota |  | Insects & other |
| Goeracea oregona | Sagehen Creek Goeracean Caddisfly | Insects & other |
| Gomphus kurilis | Pacific Clubtail | Insects & other |
| Gomphus lynnae |  | Insects & other |
| Gonidea angulata | Western Ridged Mussel | Mollusks |
| Grammotaulius betteni |  | Insects & other |
| Graphoderus liberus |  | Insects & other |
| Graphoderus occidentalis |  | Insects & other |
| Graphoderus perplexus |  | Insects & other |
| Graptocorixa abdominalis |  | Insects & other |
| Graptocorixa californica |  | Insects & other |
| Graptocorixa gerhardi |  | Insects & other |
| Graptocorixa serrulata |  | Insects & other |
| Graptocorixa uhleri |  | Insects & other |
| Graptocorixa uhleroidea | A Water Boatman | Insects & other |
| Gratiola ebracteata | Bractless Hedge-hyssop | Plants |
| Gratiola heterosepala | Boggs Lake Hedge-hyssop | Plants |
| Gratiola neglecta | Clammy Hedge-hyssop | Plants |
| Greneria humeralis |  | Insects & other |
| Grus canadensis | Sandhill Crane | Birds |
| Grus canadensis canadensis | Lesser Sandhill Crane | Birds |
| Grus canadensis tabida | Greater Sandhill Crane | Birds |
| Gumaga griseola | A Bushtailed Caddisfly | Insects & other |
| Gumaga nigricula | A Bushtailed Caddisfly | Insects & other |
| Gymnochthebius falli |  | Insects & other |
| Gymnochthebius fossatus |  | Insects & other |
| Gymnochthebius laevipennis |  | Insects & other |
| Gyraulus circumstriatus | Disc Gyro | Mollusks |
| Gyraulus crista | Star Gyro | Mollusks |
| Gyraulus deflectus |  | Mollusks |
| Gyraulus parvus | Ash Gyro | Mollusks |
| Gyraulus vermicularis | Pacific Coast Gyraulus | Mollusks |
| Gyretes sinuatus |  | Insects & other |
| Gyretes torosus |  | Insects & other |
| Gyrinus affinis |  | Insects & other |
| Gyrinus bifarius |  | Insects & other |
| Gyrinus confinis |  | Insects & other |
| Gyrinus consobrinus |  | Insects & other |
| Gyrinus latilimbus |  | Insects & other |
| Gyrinus maculiventris |  | Insects & other |
| Gyrinus parcus |  | Insects & other |
| Gyrinus picipes |  | Insects & other |
| Gyrinus pleuralis |  | Insects & other |
| Gyrinus plicifer |  | Insects & other |
| Gyrinus rugosus |  | Insects & other |
| Halesochila taylori |  | Insects & other |
| Haliaeetus leucocephalus | Bald Eagle | Birds |
| Haliaeetus leucocephalus pop. 4 | Bald Eagle - Wintering Population | Birds |
| Haliplus concolor |  | Insects & other |
| Haliplus cylindricus |  | Insects & other |
| Haliplus distinctus |  | Insects & other |
| Haliplus dorsomaculatus |  | Insects & other |
| Haliplus eremicus |  | Insects & other |
| Haliplus gracilis |  | Insects & other |
| Haliplus leechi |  | Insects & other |
| Haliplus longulus |  | Insects & other |
| Haliplus mimeticus |  | Insects & other |
| Haliplus robertsi |  | Insects & other |
| Haliplus rugosus |  | Insects & other |
| Haliplus subguttatus |  | Insects & other |
| Haliplus tumidus |  | Insects & other |
| Halobates sericeus |  | Insects & other |
| Haploperla chilnualna | Yosemite Sallfly | Insects & other |
| Harnischia curtilamellata |  | Insects & other |
| Hastingsia alba | White Rushlily | Plants |
| Hayesomyia senata |  | Insects & other |
| Hebrus buenoi |  | Insects & other |
| Hebrus hubbardi |  | Insects & other |
| Hebrus longivillus |  | Insects & other |
| Hebrus major |  | Insects & other |
| Hebrus obscurus |  | Insects & other |
| Hebrus sobrinus |  | Insects & other |
| Helenium autumnale | Common Sneezeweed | Plants |
| Helenium bigelovii | Bigelow's Sneezeweed | Plants |
| Helenium bolanderi | Coast Sneezeweed | Plants |
| Helenium puberulum | Rosilla | Plants |
| Helichus columbianus |  | Insects & other |
| Helichus striatus |  | Insects & other |
| Helichus suturalis |  | Insects & other |
| Helichus triangularis |  | Insects & other |
| Helicopsyche borealis | A Caddisfly | Insects & other |
| Helicopsyche mexicana | A Caddisfly | Insects & other |
| Helicopsyche pietia |  | Insects & other |
| Helicopsyche sinuata | A Caddisfly | Insects & other |
| Helisoma anceps | Two-ridge Rams-horn | Mollusks |
| Helisoma minus | A Freshwater Snail | Mollusks |
| Helisoma newberryi newberryi | Great Basin Rams-horn | Mollusks |
| Helisoma subcrenatum |  | Mollusks |
| Helochares normatus |  | Insects & other |
| Helodon beardi |  | Insects & other |
| Helodon chaos |  | Insects & other |
| Helodon diadelphus |  | Insects & other |
| Helodon mccreadiei |  | Insects & other |
| Helodon newmani |  | Insects & other |
| Helodon onchyodactylus |  | Insects & other |
| Helodon protus |  | Insects & other |
| Helodon susanae |  | Insects & other |
| Helodon trochus |  | Insects & other |
| Helophorus alternatus |  | Insects & other |
| Helophorus auricollis |  | Insects & other |
| Helophorus californicus |  | Insects & other |
| Helophorus columbianus |  | Insects & other |
| Helophorus cuspifer |  | Insects & other |
| Helophorus eclectus |  | Insects & other |
| Helophorus fenderi |  | Insects & other |
| Helophorus fortis |  | Insects & other |
| Helophorus hatchi |  | Insects & other |
| Helophorus lacustris |  | Insects & other |
| Helophorus lecontei |  | Insects & other |
| Helophorus ledatus |  | Insects & other |
| Helophorus leechi |  | Insects & other |
| Helophorus linearis |  | Insects & other |
| Helophorus linearoides |  | Insects & other |
| Helophorus nitiduloides |  | Insects & other |
| Helophorus nitidulus |  | Insects & other |
| Helophorus oblongus |  | Insects & other |
| Helophorus oregonus |  | Insects & other |
| Helophorus orientalis |  | Insects & other |
| Helophorus parasplendidus |  | Insects & other |
| Helophorus robertsi |  | Insects & other |
| Helophorus schuhi |  | Insects & other |
| Helophorus tuberculatus |  | Insects & other |
| Hemiosus exilis |  | Insects & other |
| Heptagenia adaequata |  | Insects & other |
| Heptagenia elegantula | A Mayfly | Insects & other |
| Heptagenia solitaria | A Mayfly | Insects & other |
| Herthania compta |  | Insects & other |
| Herthania concinna |  | Insects & other |
| Hesperagrion heterodoxum |  | Insects & other |
| Hesperocorixa atopodonta |  | Insects & other |
| Hesperocorixa laevigata |  | Insects & other |
| Hesperocorixa vulgaris |  | Insects & other |
| Hesperoperla hoguei | Banded Stone | Insects & other |
| Hesperoperla pacifica | Golden Stone | Insects & other |
| Hesperophylax alaskensis | A Caddisfly | Insects & other |
| Hesperophylax consimilis |  | Insects & other |
| Hesperophylax designatus | A Caddisfly | Insects & other |
| Hesperophylax magnus | A Caddisfly | Insects & other |
| Hesperophylax minutus | A Caddisfly | Insects & other |
| Hesperophylax occidentalis | A Caddisfly | Insects & other |
| Hetaerina americana | American Rubyspot | Insects & other |
| Hetaerina vulnerata |  | Insects & other |
| Heteranthera limosa | NA | Plants |
| Heterelmis glabra |  | Insects & other |
| Heterelmis obesa |  | Insects & other |
| Heterelmis stephani |  | Insects & other |
| Heterlimnius corpulentus |  | Insects & other |
| Heterlimnius koebelei |  | Insects & other |
| Heterocerus brunneus |  | Insects & other |
| Heterocerus gemmatus |  | Insects & other |
| Heterocerus gnatho |  | Insects & other |
| Heterocerus mexicanus |  | Insects & other |
| Heterocerus mollinus |  | Insects & other |
| Heterocerus parrotus |  | Insects & other |
| Heterocerus sinuosus |  | Insects & other |
| Heterocerus tristis |  | Insects & other |
| Heterocerus unicus |  | Insects & other |
| Heterocloeon anoka |  | Insects & other |
| Heteroplectron californicum | A Caddisfly | Insects & other |
| Heterotrissocladius oliveri |  | Insects & other |
| Hexagenia limbata | A Mayfly | Insects & other |
| Hibiscus lasiocarpos occidentalis |  | Plants |
| Himalopsyche phryganea | A Caddisfly | Insects & other |
| Himantopus mexicanus | Black-necked Stilt | Birds |
| Hippuris vulgaris | Common Mare's-tail | Plants |
| Histrionicus histrionicus | Harlequin Duck | Birds |
| Holorusia hespera |  | Insects & other |
| Homoleptohyphes dimorphus | A Mayfly | Insects & other |
| Homoleptohyphes mirus |  | Insects & other |
| Homoleptohyphes quercus |  | Insects & other |
| Homophylax adriana |  | Insects & other |
| Homophylax andax |  | Insects & other |
| Homophylax flavipennis |  | Insects & other |
| Homophylax insulas | A Caddisfly | Insects & other |
| Homophylax nevadensis | A Caddisfly | Insects & other |
| Homophylax rentzi | A Caddisfly | Insects & other |
| Homoplectra alseae |  | Insects & other |
| Homoplectra luchia |  | Insects & other |
| Homoplectra nigripennis | A Caddisfly | Insects & other |
| Homoplectra norada | A Caddisfly | Insects & other |
| Homoplectra oaklandensis | A Caddisfly | Insects & other |
| Homoplectra schuhi | Schuh's Homoplectran Caddisfly | Insects & other |
| Homoplectra shasta | A Caddisfly | Insects & other |
| Homoplectra sierra | A Caddisfly | Insects & other |
| Homoplectra spora | A Caddisfly | Insects & other |
| Hosackia oblongifolia | NA | Plants |
| Howellia aquatilis | Water Howellia | Plants |
| Hyalella azteca | An Amphipod | Crustaceans |
| Hyalella muerta | An Amphipod | Crustaceans |
| Hyalella sandra | An Amphipod | Crustaceans |
| Hydaticus aruspex |  | Insects & other |
| Hydatophylax hesperus | A Caddisfly | Insects & other |
| Hydraena alternata |  | Insects & other |
| Hydraena arenicola |  | Insects & other |
| Hydraena arizonica |  | Insects & other |
| Hydraena bituberculata |  | Insects & other |
| Hydraena californica |  | Insects & other |
| Hydraena circulata |  | Insects & other |
| Hydraena leechi |  | Insects & other |
| Hydraena mignymixys |  | Insects & other |
| Hydraena nigra |  | Insects & other |
| Hydraena occidentalis |  | Insects & other |
| Hydraena pacifica |  | Insects & other |
| Hydraena petila |  | Insects & other |
| Hydraena sierra |  | Insects & other |
| Hydraena tuolumne |  | Insects & other |
| Hydraena vandykei |  | Insects & other |
| Hydraena yosemitensis |  | Insects & other |
| Hydrobaenus pilipes |  | Insects & other |
| Hydrobaenus saetheri |  | Insects & other |
| Hydrobius fuscipes |  | Insects & other |
| Hydrochara lineata |  | Insects & other |
| Hydrochara rickseckeri | Ricksecker's Water Scavenger Beetle | Insects & other |
| Hydrochus pseudosquamifer |  | Insects & other |
| Hydrochus squamifer |  | Insects & other |
| Hydrochus vagus |  | Insects & other |
| Hydrochus variolatus |  | Insects & other |
| Hydrocotyle ranunculoides | Floating Marsh-pennywort | Plants |
| Hydrocotyle umbellata | Many-flower Marsh-pennywort | Plants |
| Hydrocotyle verticillata verticillata | Whorled Marsh-pennywort | Plants |
| Hydrometra aemula |  | Insects & other |
| Hydrometra australis |  | Insects & other |
| Hydrometra lillianis |  | Insects & other |
| Hydrometra martini |  | Insects & other |
| Hydrophilus insularis |  | Insects & other |
| Hydrophilus triangularis |  | Insects & other |
| Hydroporus axillaris |  | Insects & other |
| Hydroporus carri |  | Insects & other |
| Hydroporus despectus |  | Insects & other |
| Hydroporus fortis |  | Insects & other |
| Hydroporus klamathensis |  | Insects & other |
| Hydroporus leechi | Leech's Skyline Diving Beetle | Insects & other |
| Hydroporus longiusculus |  | Insects & other |
| Hydroporus mannerheimi |  | Insects & other |
| Hydroporus notabilis |  | Insects & other |
| Hydroporus occidentalis |  | Insects & other |
| Hydroporus pervicinus | Wooly Hydroporus Diving Beetle | Insects & other |
| Hydroporus simplex | Simple Hydroporus Diving Beetle | Insects & other |
| Hydroporus sinuatipes |  | Insects & other |
| Hydroporus subpubescens |  | Insects & other |
| Hydroporus tademus |  | Insects & other |
| Hydroporus tenebrosus |  | Insects & other |
| Hydroporus transpunctatus |  | Insects & other |
| Hydroporus tristis |  | Insects & other |
| Hydroporus zackii |  | Insects & other |
| Hydropsyche alternans |  | Insects & other |
| Hydropsyche amblis | A Caddisfly | Insects & other |
| Hydropsyche andersoni |  | Insects & other |
| Hydropsyche auricolor |  | Insects & other |
| Hydropsyche californica | A Caddisfly | Insects & other |
| Hydropsyche centra |  | Insects & other |
| Hydropsyche cockerelli | A Caddisfly | Insects & other |
| Hydropsyche cora | A Caddisfly | Insects & other |
| Hydropsyche dorata |  | Insects & other |
| Hydropsyche intrica | A Caddisfly | Insects & other |
| Hydropsyche occidentalis | A Caddisfly | Insects & other |
| Hydropsyche oslari | A Caddisfly | Insects & other |
| Hydropsyche philo | A Caddisfly | Insects & other |
| Hydropsyche protis |  | Insects & other |
| Hydropsyche tana | A Caddisfly | Insects & other |
| Hydropsyche venada |  | Insects & other |
| Hydropsyche winema |  | Insects & other |
| Hydroptila ajax | A Caddisfly | Insects & other |
| Hydroptila arctia | A Caddisfly | Insects & other |
| Hydroptila argosa | A Caddisfly | Insects & other |
| Hydroptila consimilis |  | Insects & other |
| Hydroptila hamata | A Caddisfly | Insects & other |
| Hydroptila icona | A Caddisfly | Insects & other |
| Hydroptila lenora |  | Insects & other |
| Hydroptila modica |  | Insects & other |
| Hydroptila pecos |  | Insects & other |
| Hydroptila rono | A Caddisfly | Insects & other |
| Hydroptila xera | A Caddisfly | Insects & other |
| Hydroscapha natans |  | Insects & other |
| Hydrotrupes palpalis |  | Insects & other |
| Hydrovatus brevipes |  | Insects & other |
| Hydrovatus davidis |  | Insects & other |
| Hygrotus acaroides |  | Insects & other |
| Hygrotus artus | Mono Lake Hygrotus Diving Beetle | Insects & other |
| Hygrotus bruesi |  | Insects & other |
| Hygrotus collatus |  | Insects & other |
| Hygrotus curvipes | Curved-foot Hygrotus Diving Beetle | Insects & other |
| Hygrotus dissimilis |  | Insects & other |
| Hygrotus femoratus |  | Insects & other |
| Hygrotus fontinalis | Travertine Band-thigh Diving Beetle | Insects & other |
| Hygrotus fraternus |  | Insects & other |
| Hygrotus hydropicus |  | Insects & other |
| Hygrotus impressopunctatus |  | Insects & other |
| Hygrotus infuscatus |  | Insects & other |
| Hygrotus intermedius |  | Insects & other |
| Hygrotus lutescens |  | Insects & other |
| Hygrotus marklini |  | Insects & other |
| Hygrotus masculinus |  | Insects & other |
| Hygrotus nigrescens |  | Insects & other |
| Hygrotus nubilis |  | Insects & other |
| Hygrotus obscureplagiatus |  | Insects & other |
| Hygrotus patruelis |  | Insects & other |
| Hygrotus pedalis |  | Insects & other |
| Hygrotus sayi |  | Insects & other |
| Hygrotus semivittatus |  | Insects & other |
| Hygrotus sharpi |  | Insects & other |
| Hygrotus thermarum |  | Insects & other |
| Hygrotus tumidiventris |  | Insects & other |
| Hygrotus turbidus |  | Insects & other |
| Hygrotus unguicularis |  | Insects & other |
| Hygrotus wardii |  | Insects & other |
| Hyperacanthomysis longirostris |  | Crustaceans |
| Hypericum anagalloides | Tinker's-penny | Plants |
| Hypomesus pacificus | Delta smelt | Fishes |
| Hysterocarpus traskii lagunae | Clear Lake tule perch | Fishes |
| Hysterocarpus traskii pomo | Russian River tule perch | Fishes |
| Hysterocarpus traskii traskii | Sacramento tule perch | Fishes |
| Icteria virens | Yellow-breasted Chat | Birds |
| Iliamna rivularis |  | Plants |
| Ilybius angustior |  | Insects & other |
| Ilybius fraterculus |  | Insects & other |
| Ilybius quadrimaculatus |  | Insects & other |
| Incilius alvarius | Colorado River Toad | Herps |
| Ioscytus cobbeni |  | Insects & other |
| Ioscytus franciscanus |  | Insects & other |
| Ioscytus nasti |  | Insects & other |
| Ioscytus politus |  | Insects & other |
| Ioscytus tepidarius |  | Insects & other |
| Ipnobius robustus | Robust Tryonia | Mollusks |
| Iris missouriensis | Western Blue Iris | Plants |
| Ironodes arcticus |  | Insects & other |
| Ironodes californicus | A Mayfly | Insects & other |
| Ironodes lepidus | A Mayfly | Insects & other |
| Ironodes nitidus | A Mayfly | Insects & other |
| Ischnura barberi | Desert Forktail | Insects & other |
| Ischnura cervula | Pacific Forktail | Insects & other |
| Ischnura damula |  | Insects & other |
| Ischnura demorsa |  | Insects & other |
| Ischnura denticollis | Black-fronted Forktail | Insects & other |
| Ischnura erratica | Swift Forktail | Insects & other |
| Ischnura gemina | San Francisco Forktail | Insects & other |
| Ischnura hastata | Citrine Forktail | Insects & other |
| Ischnura perparva | Western Forktail | Insects & other |
| Ischnura ramburii |  | Insects & other |
| Isocapnia abbreviata | Shortlimb Snowfly | Insects & other |
| Isocapnia agassizi |  | Insects & other |
| Isocapnia eichlini | A Stonefly | Insects & other |
| Isocapnia grandis | Giant Snowfly | Insects & other |
| Isocapnia mogila | Irregular Snowfly | Insects & other |
| Isocapnia palousa |  | Insects & other |
| Isocapnia rickeri |  | Insects & other |
| Isocapnia spenceri | Chilliwack Snowfly | Insects & other |
| Isocapnia vedderensis |  | Insects & other |
| Isoetes bolanderi | NA | Plants |
| Isoetes echinospora | NA | Plants |
| Isoetes howellii | NA | Plants |
| Isoetes nuttallii | NA | Plants |
| Isoetes occidentalis | NA | Plants |
| Isoetes orcuttii | NA | Plants |
| Isogenoides colubrinus | Blackfoot Springfly | Insects & other |
| Isogenoides elongatus |  | Insects & other |
| Isogenoides zionensis |  | Insects & other |
| Isolepis cernua | Low Bulrush | Plants |
| Isolepis setacea | NA | Plants |
| Isonychia intermedia |  | Insects & other |
| Isonychia velma | A Mayfly | Insects & other |
| Isoperla acula | Fresno Stipetail | Insects & other |
| Isoperla adunca | Arroyo Stripetail | Insects & other |
| Isoperla baumanni | California Stripetail | Insects & other |
| Isoperla bifurcata | Forked Stripetail | Insects & other |
| Isoperla denningi | Angeles Stripetail | Insects & other |
| Isoperla fulva | Western Stripetail | Insects & other |
| Isoperla gravitans |  | Insects & other |
| Isoperla karuk | Klamath Stripetail | Insects & other |
| Isoperla laucki | Humboldt Stripetail | Insects & other |
| Isoperla marmorata | Red Stripetail | Insects & other |
| Isoperla miwok | Miwok Stripetail | Insects & other |
| Isoperla mormona | Mormon Stripetail | Insects & other |
| Isoperla muir |  | Insects & other |
| Isoperla phalerata |  | Insects & other |
| Isoperla pinta | Checkered Stripetail | Insects & other |
| Isoperla quinquepunctata | Fivespot Stripetail | Insects & other |
| Isoperla raineri |  | Insects & other |
| Isoperla roguensis | Rogue Stripetail | Insects & other |
| Isoperla sobria | Colorado Stripetail | Insects & other |
| Isoperla sordida | Notched Stripetail | Insects & other |
| Isoperla tilasqua |  | Insects & other |
| Ithytrichia clavata | A Caddisfly | Insects & other |
| Ithytrichia mexicana |  | Insects & other |
| Ixobrychus exilis hesperis | Western Least Bittern | Birds |
| Jaumea carnosa | Fleshy Jaumea | Plants |
| Juga acutifilosa | Topaz Juga | Mollusks |
| Juga chacei | Chace Juga | Mollusks |
| Juga nigrina | Black Juga | Mollusks |
| Juga occata | Scalloped Juga | Mollusks |
| Juga orickensis | Redwood Juga | Mollusks |
| Juncus acuminatus | Sharp-fruit Rush | Plants |
| Juncus acutus leopoldii | Spiny Rush | Plants |
| Juncus anthelatus | NA | Plants |
| Juncus articulatus articulatus |  | Plants |
| Juncus bolanderi | Bolander's Rush | Plants |
| Juncus bryoides | Moss Rush | Plants |
| Juncus chlorocephalus | Green-head Rush | Plants |
| Juncus diffusissimus | NA | Plants |
| Juncus digitatus | Finger Rush | Plants |
| Juncus dubius | Mariposa Rush | Plants |
| Juncus duranii | Duran's Rush | Plants |
| Juncus effusus austrocalifornicus |  | Plants |
| Juncus effusus effusus | NA | Plants |
| Juncus effusus pacificus |  | Plants |
| Juncus exiguus |  | Plants |
| Juncus falcatus falcatus | Sickle-leaf Rush | Plants |
| Juncus falcatus sitchensis |  | Plants |
| Juncus hemiendytus abjectus | Dwarf Rush | Plants |
| Juncus hemiendytus hemiendytus | Dwarf Rush | Plants |
| Juncus hesperius |  | Plants |
| Juncus leiospermus | NA | Plants |
| Juncus lescurii |  | Plants |
| Juncus luciensis | Santa Lucia Dwarf Rush | Plants |
| Juncus macrandrus | Long-anther Rush | Plants |
| Juncus macrophyllus | Longleaf Rush | Plants |
| Juncus marginatus | NA | Plants |
| Juncus mertensianus | Mertens' Rush | Plants |
| Juncus nevadensis inventus | Sierra Rush | Plants |
| Juncus nodosus | NA | Plants |
| Juncus phaeocephalus paniculatus | Brownhead Rush | Plants |
| Juncus phaeocephalus phaeocephalus | Brown-head Rush | Plants |
| Juncus planifolius | NA | Plants |
| Juncus regelii | Regel's Rush | Plants |
| Juncus rugulosus | Wrinkled Rush | Plants |
| Juncus saximontanus | Rocky Mountain Rush | Plants |
| Juncus supiniformis | Hairyleaf Rush | Plants |
| Juncus textilis | Basket Rush | Plants |
| Juncus uncialis | Inch-high Rush | Plants |
| Juncus usitatus | NA | Plants |
| Juncus xiphioides | Iris-leaf Rush | Plants |
| Kathroperla perdita | Longhead Sallfly | Insects & other |
| Kathroperla takhoma | Slenderhead Sallfly | Insects & other |
| Kiefferulus dux |  | Insects & other |
| Kiefferulus modocensis |  | Insects & other |
| Kinosternon sonoriense | Sonoran Mud Turtle | Herps |
| Kobresia myosuroides | Pacific Kobresia | Plants |
| Kogotus nonus | Smooth Springfly | Insects & other |
| Konikea expansipalpis |  | Insects & other |
| Krenopelopia narda |  | Insects & other |
| Kyhosia bolanderi |  | Plants |
| Labrundinia maculata |  | Insects & other |
| Labrundinia pilosella |  | Insects & other |
| Laccobius acutipenis |  | Insects & other |
| Laccobius agilis |  | Insects & other |
| Laccobius borealis |  | Insects & other |
| Laccobius bruesi |  | Insects & other |
| Laccobius californicus |  | Insects & other |
| Laccobius carri |  | Insects & other |
| Laccobius ellipticus |  | Insects & other |
| Laccobius hardyi |  | Insects & other |
| Laccobius insolitus |  | Insects & other |
| Laccobius leechi |  | Insects & other |
| Laccobius mexicanus |  | Insects & other |
| Laccobius nevadensis |  | Insects & other |
| Laccobius occidentalis |  | Insects & other |
| Laccobius oregonensis |  | Insects & other |
| Laccobius pacificus |  | Insects & other |
| Laccobius piceus |  | Insects & other |
| Laccobius tridentipenis |  | Insects & other |
| Laccobius truncatipenis |  | Insects & other |
| Laccophilus biguttatus |  | Insects & other |
| Laccophilus fasciatus terminalis |  | Insects & other |
| Laccophilus horni |  | Insects & other |
| Laccophilus maculosus |  | Insects & other |
| Laccophilus maculosus decipiens |  | Insects & other |
| Laccophilus maculosus shermani |  | Insects & other |
| Laccophilus mexicanus atristernalis |  | Insects & other |
| Laccophilus mexicanus mexicanus |  | Insects & other |
| Laccophilus oscillator |  | Insects & other |
| Laccophilus pictus |  | Insects & other |
| Laccophilus quadrilineatus quadrilineatus | | Insects & other |
| Laccophilus salvini |  | Insects & other |
| Laccophilus sonorensis |  | Insects & other |
| Laccophilus vacaensis |  | Insects & other |
| Laccornis pacificus |  | Insects & other |
| Lachlania saskatchewanensis |  | Insects & other |
| Ladona julia | Chalk-fronted Corporal | Insects & other |
| Lampetra ayersi | River lamprey | Fishes |
| Lampetra hubbsi | Kern brook lamprey | Fishes |
| Lampetra lethophaga | Pit-Klamath brook lamprey | Fishes |
| Lampetra richardsoni | Western brook lamprey | Fishes |
| Landoltia punctata | NA | Plants |
| Lanx alta | Highcap Lanx | Mollusks |
| Lanx hannai |  | Mollusks |
| Lanx klamathensis | Scale Lanx | Mollusks |
| Lanx patelloides | Kneecap Lanx | Mollusks |
| Lanx subrotundatus |  | Mollusks |
| Lara avara |  | Insects & other |
| Lara gehringi |  | Insects & other |
| Larsia decolorata |  | Insects & other |
| Larsia lyra |  | Insects & other |
| Larsia marginella |  | Insects & other |
| Larsia planensis |  | Insects & other |
| Larsia sequoiaensis |  | Insects & other |
| Larus livens | Yellow-footed Gull | Birds |
| Lasthenia burkei | Burke's Goldfields | Plants |
| Lasthenia conjugens | Contra Costa Goldfields | Plants |
| Lasthenia ferrisiae | Ferris' Goldfields | Plants |
| Lasthenia fremontii | Fremont's Goldfields | Plants |
| Lasthenia glabrata coulteri | Coulter's Goldfields | Plants |
| Laterallus jamaicensis coturniculus | California Black Rail | Birds |
| Lathyrus jepsonii | NA | Plants |
| Lathyrus palustris | Vetchling Peavine | Plants |
| Lauterborniella agrayloides |  | Insects & other |
| Lavinia exilicauda chi | Clear Lake hitch | Fishes |
| Lavinia exilicauda exilicauda | Sacramento hitch | Fishes |
| Lavinia exilicauda harengeus | Monterey hitch | Fishes |
| Lavinia mitrulus | Northern (Pit) roach | Fishes |
| Lavinia parvipinnus | Gualala roach | Fishes |
| Lavinia symmetricus navarroensis | Navarro roach | Fishes |
| Lavinia symmetricus ssp. 1 | Russian River roach | Fishes |
| Lavinia symmetricus ssp. 2 | Red Hills roach | Fishes |
| Lavinia symmetricus ssp. 3 | Clear Lake roach | Fishes |
| Lavinia symmetricus ssp. 4 | Tomales roach | Fishes |
| Lavinia symmetricus subditus | Monterey roach | Fishes |
| Lavinia symmetricus symmetricus | Central California roach | Fishes |
| Lednia sierra | A Stonefly | Insects & other |
| Leersia oryzoides | Rice Cutgrass | Plants |
| Legenere limosa | False Venus'-looking-glass | Plants |
| Lemna aequinoctialis | Lesser Duckweed | Plants |
| Lemna gibba | Inflated Duckweed | Plants |
| Lemna minor | Lesser Duckweed | Plants |
| Lemna minuta | Least Duckweed | Plants |
| Lemna trisulca | Star Duckweed | Plants |
| Lemna turionifera | Turion Duckweed | Plants |
| Lemna valdiviana | Pale Duckweed | Plants |
| Lenarchus brevipennis |  | Insects & other |
| Lenarchus gravidus | A Caddisfly | Insects & other |
| Lenarchus rho |  | Insects & other |
| Lenarchus rillus | A Caddisfly | Insects & other |
| Lenarchus vastus | A Caddisfly | Insects & other |
| Lepania cascada |  | Insects & other |
| Lepidium jaredii jaredii | Jared's Pepper-grass | Plants |
| Lepidium oxycarpum | Sharp-pod Pepper-grass | Plants |
| Lepidostoma acarolum |  | Insects & other |
| Lepidostoma apache |  | Insects & other |
| Lepidostoma apornum |  | Insects & other |
| Lepidostoma astaneum | A Caddisfly | Insects & other |
| Lepidostoma bakeri |  | Insects & other |
| Lepidostoma baxea | A Caddisfly | Insects & other |
| Lepidostoma canthum | A Caddisfly | Insects & other |
| Lepidostoma cascadense | A Caddisfly | Insects & other |
| Lepidostoma castalianum | A Caddisfly | Insects & other |
| Lepidostoma cinereum | A Caddisfly | Insects & other |
| Lepidostoma ermanae | Cold Spring Caddisfly | Insects & other |
| Lepidostoma errigenum | A Caddisfly | Insects & other |
| Lepidostoma hoodi |  | Insects & other |
| Lepidostoma jewetti | A Caddisfly | Insects & other |
| Lepidostoma knulli |  | Insects & other |
| Lepidostoma lacinatum |  | Insects & other |
| Lepidostoma licolum | A Caddisfly | Insects & other |
| Lepidostoma lotor | A Caddisfly | Insects & other |
| Lepidostoma mexicanum |  | Insects & other |
| Lepidostoma ojanum | A Caddisfly | Insects & other |
| Lepidostoma ormeum |  | Insects & other |
| Lepidostoma pluviale | A Caddisfly | Insects & other |
| Lepidostoma podagrum | A Caddisfly | Insects & other |
| Lepidostoma quericinum |  | Insects & other |
| Lepidostoma rayneri | A Caddisfly | Insects & other |
| Lepidostoma recinum | A Caddisfly | Insects & other |
| Lepidostoma roafi | A Caddisfly | Insects & other |
| Lepidostoma stigma |  | Insects & other |
| Lepidostoma unicolor | A Caddisfly | Insects & other |
| Lepidostoma verodum | A Caddisfly | Insects & other |
| Lepidurus bilobatus |  | Crustaceans |
| Lepidurus cryptus | Cryptic Tadpole Shrimp | Crustaceans |
| Lepidurus lemmoni | Lynch Tadpole Shrimp | Crustaceans |
| Lepidurus packardi | Vernal Pool Tadpole Shrimp | Crustaceans |
| Leptestheria compleximanus | Spineynose Clam Shrimp | Crustaceans |
| Leptohyphes apache |  | Insects & other |
| Leptohyphes ferruginus |  | Insects & other |
| Leptohyphes lestes |  | Insects & other |
| Leptohyphes zalope |  | Insects & other |
| Leptophlebia cupida | A Mayfly | Insects & other |
| Leptophlebia pacifica | A Mayfly | Insects & other |
| Lestes alacer |  | Insects & other |
| Lestes congener | Spotted Spreadwing | Insects & other |
| Lestes disjunctus | Northern Spreadwing | Insects & other |
| Lestes dryas | Emerald Spreadwing | Insects & other |
| Lestes stultus | Black Spreadwing | Insects & other |
| Lestes unguiculatus | Lyre-tipped Spreadwing | Insects & other |
| Lethocerus americanus |  | Insects & other |
| Lethocerus angustipes |  | Insects & other |
| Lethocerus medius |  | Insects & other |
| Leucorrhinia glacialis | Crimson-ringed Whiteface | Insects & other |
| Leucorrhinia hudsonica | Hudsonian Whiteface | Insects & other |
| Leucorrhinia intacta | Dot-tailed Whiteface | Insects & other |
| Leucorrhinia proxima | Belted Whiteface | Insects & other |
| Leucothoe davisiae | Western Doghobble | Plants |
| Leucotrichia limpia |  | Insects & other |
| Leucotrichia pictipes | A Micro Caddisfly | Insects & other |
| Leucotrichia sarita |  | Insects & other |
| Leucrocuta jewetti |  | Insects & other |
| Lewisia cantelovii | Cantelow's Lewisia | Plants |
| Libellula comanche | Comanche Skimmer | Insects & other |
| Libellula composita | Bleached Skimmer | Insects & other |
| Libellula croceipennis | Neon Skimmer | Insects & other |
| Libellula forensis | Eight-spotted Skimmer | Insects & other |
| Libellula luctuosa | Widow Skimmer | Insects & other |
| Libellula nodisticta | Hoary Skimmer | Insects & other |
| Libellula pulchella | Twelve-spotted Skimmer | Insects & other |
| Libellula quadrimaculata | Four-spotted Skimmer | Insects & other |
| Libellula saturata | Flame Skimmer | Insects & other |
| Lichminus tenuicornis |  | Insects & other |
| Ligidium kofoidi | A Cave Obligate Isopod | Crustaceans |
| Lilaeopsis masonii | Mason's Lilaeopsis | Plants |
| Lilaeopsis occidentalis | Western Lilaeopsis | Plants |
| Lilium kelleyanum | Kelley's Lily | Plants |
| Lilium pardalinum pardalinum | Leopard Lily | Plants |
| Lilium pardalinum pitkinense | Pitkin Marsh Lily | Plants |
| Lilium pardalinum shastense | Leopard Lily | Plants |
| Lilium pardalinum vollmeri | Vollmer's Lily | Plants |
| Lilium pardalinum wigginsii | Wiggin's Lily | Plants |
| Lilium parryi | Lemon Lily | Plants |
| Lilium parvum | Small Tiger Lily | Plants |
| Limnanthes alba alba | White Meadowfoam | Plants |
| Limnanthes alba parishii | NA | Plants |
| Limnanthes alba versicolor | White Meadowfoam | Plants |
| Limnanthes bakeri | Baker's Meadowfoam | Plants |
| Limnanthes douglasii douglasii | Douglas' Meadowfoam | Plants |
| Limnanthes douglasii nivea | Douglas' Meadowfoam | Plants |
| Limnanthes douglasii rosea | Douglas' Meadowfoam | Plants |
| Limnanthes douglasii striata |  | Plants |
| Limnanthes douglasii sulphurea | Pt. Reyes Meadowfoam | Plants |
| Limnanthes floccosa bellingeriana | Bellinger's Meadowfoam | Plants |
| Limnanthes floccosa californica | Shippee Meadowfoam | Plants |
| Limnanthes floccosa floccosa | Woolly Meadowfoam | Plants |
| Limnanthes montana | Mountain Meadowfoam | Plants |
| Limnanthes vinculans | Sebastopol Meadowfoam | Plants |
| Limnebius alutaceous |  | Insects & other |
| Limnebius arenicolus |  | Insects & other |
| Limnebius leechi |  | Insects & other |
| Limnebius piceus |  | Insects & other |
| Limnebius sinuatus |  | Insects & other |
| Limnephilus abbreviatus |  | Insects & other |
| Limnephilus acnestus | A Caddisfly | Insects & other |
| Limnephilus acula | A Caddisfly | Insects & other |
| Limnephilus alconura | Klamath Limnephilan Caddisfly | Insects & other |
| Limnephilus apache |  | Insects & other |
| Limnephilus aretto | A Caddisfly | Insects & other |
| Limnephilus arizona |  | Insects & other |
| Limnephilus assimilis | A Caddisfly | Insects & other |
| Limnephilus atercus | Fort Dick Limnephilus Caddisfly | Insects & other |
| Limnephilus bucketti | A Caddisfly | Insects & other |
| Limnephilus canadensis |  | Insects & other |
| Limnephilus catula | A Caddisfly | Insects & other |
| Limnephilus coloradensis | A Caddisfly | Insects & other |
| Limnephilus concolor | A Caddisfly | Insects & other |
| Limnephilus diversus |  | Insects & other |
| Limnephilus ectus |  | Insects & other |
| Limnephilus elongatus |  | Insects & other |
| Limnephilus externus | A Caddisfly | Insects & other |
| Limnephilus fagus |  | Insects & other |
| Limnephilus frijole | A Caddisfly | Insects & other |
| Limnephilus granti |  | Insects & other |
| Limnephilus hyalinus |  | Insects & other |
| Limnephilus insularis |  | Insects & other |
| Limnephilus kalama |  | Insects & other |
| Limnephilus kennicotti |  | Insects & other |
| Limnephilus lithus |  | Insects & other |
| Limnephilus lopho |  | Insects & other |
| Limnephilus lunonus |  | Insects & other |
| Limnephilus moestus |  | Insects & other |
| Limnephilus morrisoni | A Caddisfly | Insects & other |
| Limnephilus neoacula |  | Insects & other |
| Limnephilus nogus | A Caddisfly | Insects & other |
| Limnephilus occidentalis | A Caddisfly | Insects & other |
| Limnephilus peltus | A Caddisfly | Insects & other |
| Limnephilus productus | A Caddisfly | Insects & other |
| Limnephilus rothi |  | Insects & other |
| Limnephilus santanus |  | Insects & other |
| Limnephilus secludens | A Caddisfly | Insects & other |
| Limnephilus sericeus |  | Insects & other |
| Limnephilus sierrata | A Caddisfly | Insects & other |
| Limnephilus silviae |  | Insects & other |
| Limnephilus sitchensis |  | Insects & other |
| Limnephilus spinatus | A Caddisfly | Insects & other |
| Limnephilus tulatus |  | Insects & other |
| Limnichites foraminosus |  | Insects & other |
| Limnichites nebulosus |  | Insects & other |
| Limnichites perforatus |  | Insects & other |
| Limnichoderus lutrochinus |  | Insects & other |
| Limnichoderus naviculatus |  | Insects & other |
| Limnobium spongia | NA | Plants |
| Limnochares anomala |  | Insects & other |
| Limnocoris moapensis |  | Insects & other |
| Limnodromus scolopaceus | Long-billed Dowitcher | Birds |
| Limnophyes asquamatus |  | Insects & other |
| Limnophyes doughmani |  | Insects & other |
| Limnophyes hamiltoni |  | Insects & other |
| Limnophyes natalensis |  | Insects & other |
| Limnophyes pilicistulus |  | Insects & other |
| Limnoporus notabilis |  | Insects & other |
| Limonium californicum | California Sea-lavender | Plants |
| Limosella acaulis | Southern Mudwort | Plants |
| Limosella aquatica | Northern Mudwort | Plants |
| Limosella australis | NA | Plants |
| Linderiella occidentalis | California Fairy Shrimp | Crustaceans |
| Linderiella santarosae | Santa Rosa Plateau Fairy Shrimp | Crustaceans |
| Lindernia dubia | Yellowseed False Pimpernel | Plants |
| Liodessus obscurellus |  | Insects & other |
| Liodessus saratogae |  | Insects & other |
| Lipocarpha micrantha | Dwarf Bulrush | Plants |
| Lithobates pipiens | Northern Leopard Frog | Herps |
| Lithobates yavapaiensis | Yavapai Leopard Frog | Herps |
| Lobelia cardinalis cardinalis | NA | Plants |
| Lobelia cardinalis pseudosplendens |  | Plants |
| Lobelia dunnii serrata | Dunn's Lobelia | Plants |
| Lontra canadensis canadensis | North American River Otter | Mammals |
| Lontra canadensis sonora | Southwestern River Otter | Mammals |
| Lophodytes cucullatus | Hooded Merganser | Birds |
| Ludwigia grandiflora | NA | Plants |
| Ludwigia hexapetala | NA | Plants |
| Ludwigia palustris | Marsh Seedbox | Plants |
| Ludwigia peploides montevidensis | NA | Plants |
| Ludwigia peploides peploides | NA | Plants |
| Ludwigia repens | Creeping Seedbox | Plants |
| Lupinus polyphyllus burkei |  | Plants |
| Lupinus polyphyllus pallidipes | Largeleaf Lupine | Plants |
| Lupinus polyphyllus polyphyllus | Bigleaf Lupine | Plants |
| Lutrochus arizonensis |  | Insects & other |
| Lycastoides alticola |  | Insects & other |
| Lycopodiella inundata | NA | Plants |
| Lycopus americanus | American Bugleweed | Plants |
| Lycopus uniflorus uniflorus | Northern Bugleweed | Plants |
| Lymnaea stagnalis | Swamp Lymnaea | Mollusks |
| Lynceus brachyurus | Holarctic Clam Shrimp | Crustaceans |
| Lynceus brevifrons |  | Crustaceans |
| Lysichiton americanus | Yellow Skunk-cabbage | Plants |
| Lysimachia thyrsiflora | Water Loosestrife | Plants |
| Lythrum californicum | California Loosestrife | Plants |
| Lythrum portula | NA | Plants |
| Maccaffertium terminatum | A Mayfly | Insects & other |
| Macrelmis moestus |  | Insects & other |
| Macrodiplax balteata | Marl Pennant | Insects & other |
| Macromia magnifica | Western River Cruiser | Insects & other |
| Macrothemis inacuta |  | Insects & other |
| Macrovelia hornii |  | Insects & other |
| Malenka bifurcata |  | Insects & other |
| Malenka biloba | Two-lobed Forestfly | Insects & other |
| Malenka californica | California Forestfly | Insects & other |
| Malenka coloradensis |  | Insects & other |
| Malenka cornuta | Horned Forestfly | Insects & other |
| Malenka depressa | Bluntlobe Forestfly | Insects & other |
| Malenka flexura |  | Insects & other |
| Malenka marionae | Sagehen Forestfly | Insects & other |
| Malenka murvoshi |  | Insects & other |
| Malenka perplexa |  | Insects & other |
| Malenka tina |  | Insects & other |
| Margaritifera falcata | Western Pearlshell | Mollusks |
| Marilia flexuosa | A Caddisfly | Insects & other |
| Marilia nobsca |  | Insects & other |
| Marsilea oligospora | NA | Plants |
| Marsilea vestita vestita | NA | Plants |
| Martarega mexicana |  | Insects & other |
| Maruina lanceolata |  | Insects & other |
| Matriella teresa | A Mayfly | Insects & other |
| Mayatrichia acuna |  | Insects & other |
| Mayatrichia ayama |  | Insects & other |
| Mayatrichia ponta |  | Insects & other |
| Megaceryle alcyon | Belted Kingfisher | Birds |
| Megaleuctra complicata |  | Insects & other |
| Megaleuctra kincaidi |  | Insects & other |
| Megaleuctra sierra | Sierra Needlefly | Insects & other |
| Megarcys signata |  | Insects & other |
| Megarcys subtruncata |  | Insects & other |
| Megarcys yosemite | Yosemite Springfly | Insects & other |
| Menetus opercularis | Button Sprite | Mollusks |
| Menyanthes trifoliata | Bog Buckbean | Plants |
| Mergus merganser | Common Merganser | Birds |
| Mergus serrator | Red-breasted Merganser | Birds |
| Meringodixa chalonensis |  | Insects & other |
| Meropelopia flavifrons |  | Insects & other |
| Merragata hebroides |  | Insects & other |
| Mesocapnia arizonensis |  | Insects & other |
| Mesocapnia autumna |  | Insects & other |
| Mesocapnia bakeri | Pomona Snowfly | Insects & other |
| Mesocapnia bulbosa | Bulbous Snowfly | Insects & other |
| Mesocapnia frisoni |  | Insects & other |
| Mesocapnia lapwae |  | Insects & other |
| Mesocapnia oenone |  | Insects & other |
| Mesocapnia porrecta | Stretched Snowfly | Insects & other |
| Mesocapnia projecta | Spined Snowfly | Insects & other |
| Mesocapnia werneri | Sabino Snowfly | Insects & other |
| Mesocapnia yoloensis | Yolo Snowfly | Insects & other |
| Mesovelia amoena |  | Insects & other |
| Mesovelia mulsanti |  | Insects & other |
| Metacnephia coloradensis |  | Insects & other |
| Metacnephia jeanae |  | Insects & other |
| Metacnephia villosa |  | Insects & other |
| Metrichia arizonensis |  | Insects & other |
| Metrichia nigritta |  | Insects & other |
| Metriocnemus edwardsi |  | Insects & other |
| Metriocnemus stevensi |  | Insects & other |
| Metriocnemus yaquina |  | Insects & other |
| Metrobates denticornis |  | Insects & other |
| Metrobates trux |  | Insects & other |
| Micracanthia fennica |  | Insects & other |
| Micracanthia humilis |  | Insects & other |
| Micracanthia quadrimaculata |  | Insects & other |
| Micracanthia schuhi |  | Insects & other |
| Micracanthia utahensis |  | Insects & other |
| Micranthes aprica |  | Plants |
| Micranthes marshallii | NA | Plants |
| Micranthes odontoloma |  | Plants |
| Micranthes oregana | NA | Plants |
| Micrasema arizonica |  | Insects & other |
| Micrasema bactro | A Caddisfly | Insects & other |
| Micrasema dimicki |  | Insects & other |
| Micrasema diteris | A Caddisfly | Insects & other |
| Micrasema onisca | A Caddisfly | Insects & other |
| Micrasema oregona |  | Insects & other |
| Microchironomus nigrovittatus |  | Insects & other |
| Microcylloepus formicoideus | Furnace Creek Riffle Beetle | Insects & other |
| Microcylloepus moapus |  | Insects & other |
| Microcylloepus similis |  | Insects & other |
| Microcylloepus thermarum |  | Insects & other |
| Micromenetus dilatatus | Bugle Sprite | Mollusks |
| Micropsectra nigripila |  | Insects & other |
| Micropsectra polita |  | Insects & other |
| Microtendipes caducus |  | Insects & other |
| Microtendipes pedellus |  | Insects & other |
| Microvelia beameri |  | Insects & other |
| Microvelia buenoi |  | Insects & other |
| Microvelia californiensis |  | Insects & other |
| Microvelia cerifera |  | Insects & other |
| Microvelia fasculifera |  | Insects & other |
| Microvelia gerhardi |  | Insects & other |
| Microvelia glabrosulcata |  | Insects & other |
| Microvelia hinei |  | Insects & other |
| Microvelia paludicola |  | Insects & other |
| Microvelia pulchella |  | Insects & other |
| Microvelia rasilis |  | Insects & other |
| Microvelia rufescens |  | Insects & other |
| Microvelia signata |  | Insects & other |
| Microvelia torquata |  | Insects & other |
| Mideopsis pumila |  | Insects & other |
| Mimulus alsinoides | Chickweed Monkeyflower | Plants |
| Mimulus angustatus | Narrowleaf Pansy Monkeyflower | Plants |
| Mimulus breviflorus | Short-flower Monkeyflower | Plants |
| Mimulus cardinalis | Scarlet Monkeyflower | Plants |
| Mimulus dentatus | Tooth-leaf Monkeyflower | Plants |
| Mimulus evanescens | Disappearing Monkeyflower | Plants |
| Mimulus glaucescens | Shield-bract Monkeyflower | Plants |
| Mimulus guttatus | Common Large Monkeyflower | Plants |
| Mimulus laciniatus | Cutleaf Monkeyflower | Plants |
| Mimulus latidens | Broad-tooth Monkeyflower | Plants |
| Mimulus lewisii | Lewis' Monkeyflower | Plants |
| Mimulus nudatus | Bare Monkeyflower | Plants |
| Mimulus parishii | Parish's Monkeyflower | Plants |
| Mimulus pilosus |  | Plants |
| Mimulus primuloides linearifolius | Primrose Monkeyflower | Plants |
| Mimulus primuloides primuloides | Primrose Monkeyflower | Plants |
| Mimulus pulchellus | Pansy Monkeyflower | Plants |
| Mimulus ringens | Square-stem Monkeyflower | Plants |
| Mimulus tilingii tilingii | Subalpine Monkeyflower | Plants |
| Mimulus tricolor | Tricolor Monkeyflower | Plants |
| Mitellastra caulescens |  | Plants |
| Momonia projecta |  | Insects & other |
| Monopelopia tenuicalcar |  | Insects & other |
| Montia chamissoi | Chamisso's Miner's-lettuce | Plants |
| Montia fontana fontana | Fountain Miner's-lettuce | Plants |
| Montia howellii | Howell's Miner's-lettuce | Plants |
| Moribaetis mimbresaurus |  | Insects & other |
| Morphocorixa lundbladi |  | Insects & other |
| Moselia infuscata | Hairy Needlefly | Insects & other |
| Moselyana comosa |  | Insects & other |
| Muhlenbergia utilis | Aparejo Grass | Plants |
| Musulium partumeium |  | Mollusks |
| Musulium secuirs |  | Mollusks |
| Mycteria americana | Wood Stork | Birds |
| Mylopharodon conocephalus | Hardhead | Fishes |
| Myosotis laxa | Small Forget-me-not | Plants |
| Myosotis scorpioides | NA | Plants |
| Myosurus apetalus | Bristly Mousetail | Plants |
| Myosurus minimus | NA | Plants |
| Myosurus sessilis | Sessile Mousetail | Plants |
| Myriophyllum aquaticum | NA | Plants |
| Myriophyllum hippuroides | Western Water-milfoil | Plants |
| Myriophyllum quitense | Andean Water-milfoil | Plants |
| Myriophyllum sibiricum | Common Water-milfoil | Plants |
| Myriophyllum verticillatum | Whorled Water-milfoil | Plants |
| Mysis diluviana |  | Crustaceans |
| Mystacides alafimbriatus | A Caddisfly | Insects & other |
| Mystacides interjecta |  | Insects & other |
| Mystacides sepulchralis | A Caddisfly | Insects & other |
| Najas flexilis | Slender Naiad | Plants |
| Najas gracillima | NA | Plants |
| Najas guadalupensis guadalupensis | Southern Naiad | Plants |
| Namamyia plutonis | A Caddisfly | Insects & other |
| Namanereis hawaiiensis |  | Insects & other |
| Nanocladius anderseni |  | Insects & other |
| Nanonemoura wahkeena |  | Insects & other |
| Narpus angustus |  | Insects & other |
| Narpus arizonicus |  | Insects & other |
| Narpus concolor |  | Insects & other |
| Narthecium californicum | California Bog Asphodel | Plants |
| Nasturtium gambelii | NA | Plants |
| Natarsia miripes |  | Insects & other |
| Navarretia cotulifolia | Cotula Navarretia | Plants |
| Navarretia fossalis | Spreading Navarretia | Plants |
| Navarretia heterandra | Tehama Navarretia | Plants |
| Navarretia intertexta | Needleleaf Navarretia | Plants |
| Navarretia leucocephala bakeri | Baker's Navarretia | Plants |
| Navarretia leucocephala leucocephala | White-flower Navarretia | Plants |
| Navarretia leucocephala minima | Least Navarretia | Plants |
| Navarretia leucocephala pauciflora | Few-flower Navarretia | Plants |
| Navarretia leucocephala plieantha | Many-flower Navarretia | Plants |
| Navarretia myersii deminuta | Small Pincushion Navarretia | Plants |
| Navarretia myersii myersii | Pincushion Navarretia | Plants |
| Navarretia prostrata | Prostrate Navarretia | Plants |
| Neanthes limnicola |  | Insects & other |
| Nectopsyche dorsalis | A Caddisfly | Insects & other |
| Nectopsyche gracilis | A Caddisfly | Insects & other |
| Nectopsyche lahontanensis | A Caddisfly | Insects & other |
| Nectopsyche minuta | A Caddisfly | Insects & other |
| Nectopsyche stigmatica |  | Insects & other |
| Nehalennia irene | Sedge Sprite | Insects & other |
| Nemotaulius hostilis |  | Insects & other |
| Nemoura spiniloba | Spiny Forestfly | Insects & other |
| Neochoroterpes kossi |  | Insects & other |
| Neochthebius vandykei |  | Insects & other |
| Neoclypeodytes amybethae |  | Insects & other |
| Neoclypeodytes cinctellus |  | Insects & other |
| Neoclypeodytes fryii |  | Insects & other |
| Neoclypeodytes haroldi |  | Insects & other |
| Neoclypeodytes leachi |  | Insects & other |
| Neoclypeodytes ornatellus |  | Insects & other |
| Neoclypeodytes pictodes |  | Insects & other |
| Neoclypeodytes plicipennis |  | Insects & other |
| Neoclypeodytes quadripustulatus |  | Insects & other |
| Neoclypeodytes roughleyi |  | Insects & other |
| Neocorixa snowi |  | Insects & other |
| Neohermes californicus |  | Insects & other |
| Neohermes filicornis |  | Insects & other |
| Neomideopsis siuslawensis |  | Insects & other |
| Neomysis kadiakensis | A Mysid Shrimp | Crustaceans |
| Neomysis mercedis |  | Crustaceans |
| Neophylax occidentis | A Caddisfly | Insects & other |
| Neophylax rickeri | A Caddisfly | Insects & other |
| Neophylax splendens | A Caddisfly | Insects & other |
| Neoplea striola |  | Insects & other |
| Neoporus arizonicus |  | Insects & other |
| Neoporus dimidiatus |  | Insects & other |
| Neoporus undulatus |  | Insects & other |
| Neostapfia colusana | Colusa Grass | Plants |
| Neothremma alicia | A Caddisfly | Insects & other |
| Neothremma andersoni |  | Insects & other |
| Neothremma didactyla |  | Insects & other |
| Neothremma genella | Golden-horned Caddisfly | Insects & other |
| Neothremma macronata | A Caddisfly | Insects & other |
| Neothremma siskiyou | Siskiyou Caddisfly | Insects & other |
| Neotrichia blinni |  | Insects & other |
| Neotrichia halia | A Caddisfly | Insects & other |
| Neotrichia okopa | A Caddisfly | Insects & other |
| Neotrichia olorino |  | Insects & other |
| Neotrichia osmena |  | Insects & other |
| Neotrichia sandyae |  | Insects & other |
| Neotrichia sonoroa |  | Insects & other |
| Neovison vison | American Mink | Mammals |
| Nereis succinea |  | Insects & other |
| Nerophilus californicus | A Caddisfly | Insects & other |
| Nerthra manni |  | Insects & other |
| Nerthra martini |  | Insects & other |
| Nerthra mexicana |  | Insects & other |
| Nilotanypus fimbriatus |  | Insects & other |
| Nilothauma babiyi |  | Insects & other |
| Nilothauma mirabile |  | Insects & other |
| Nitrophila mohavensis | Amargosa Niterwort | Plants |
| Nixe kennedyi | A Mayfly | Insects & other |
| Nothotrichia shasta |  | Insects & other |
| Notonecta hoffmani |  | Insects & other |
| Notonecta indica |  | Insects & other |
| Notonecta irrorata |  | Insects & other |
| Notonecta kirbyi |  | Insects & other |
| Notonecta lobata |  | Insects & other |
| Notonecta repanda |  | Insects & other |
| Notonecta shooteri |  | Insects & other |
| Notonecta spinosa |  | Insects & other |
| Notonecta undulata |  | Insects & other |
| Notonecta unifasciata |  | Insects & other |
| Numenius americanus | Long-billed Curlew | Birds |
| Numenius phaeopus | Whimbrel | Birds |
| Nuphar polysepala |  | Plants |
| Nycticorax nycticorax | Black-crowned Night-Heron | Birds |
| Nyctiophylax moestus |  | Insects & other |
| Nymphaea mexicana | NA | Plants |
| Ochlerotatus aboriginis |  | Insects & other |
| Ochlerotatus aloponotum |  | Insects & other |
| Ochlerotatus bicristatus |  | Insects & other |
| Ochlerotatus burgeri |  | Insects & other |
| Ochlerotatus campestris |  | Insects & other |
| Ochlerotatus cataphylla |  | Insects & other |
| Ochlerotatus clivis |  | Insects & other |
| Ochlerotatus communis |  | Insects & other |
| Ochlerotatus deserticola |  | Insects & other |
| Ochlerotatus dorsalis |  | Insects & other |
| Ochlerotatus epactius |  | Insects & other |
| Ochlerotatus excrucians |  | Insects & other |
| Ochlerotatus fitchii |  | Insects & other |
| Ochlerotatus flavescens |  | Insects & other |
| Ochlerotatus hendersoni |  | Insects & other |
| Ochlerotatus hexodontus |  | Insects & other |
| Ochlerotatus impiger |  | Insects & other |
| Ochlerotatus implicatus |  | Insects & other |
| Ochlerotatus increpitus |  | Insects & other |
| Ochlerotatus intrudens |  | Insects & other |
| Ochlerotatus melanimon |  | Insects & other |
| Ochlerotatus monticola |  | Insects & other |
| Ochlerotatus muelleri |  | Insects & other |
| Ochlerotatus nevadensis |  | Insects & other |
| Ochlerotatus nigromaculatus |  | Insects & other |
| Ochlerotatus niphadopsis |  | Insects & other |
| Ochlerotatus papago |  | Insects & other |
| Ochlerotatus provocans |  | Insects & other |
| Ochlerotatus pullatus |  | Insects & other |
| Ochlerotatus purpureipes |  | Insects & other |
| Ochlerotatus schizopinax |  | Insects & other |
| Ochlerotatus sierrensis |  | Insects & other |
| Ochlerotatus sollicitans |  | Insects & other |
| Ochlerotatus squamiger |  | Insects & other |
| Ochlerotatus sticticus |  | Insects & other |
| Ochlerotatus taeniorhynchus |  | Insects & other |
| Ochlerotatus tahoensis |  | Insects & other |
| Ochlerotatus thelcter |  | Insects & other |
| Ochlerotatus trivittatus |  | Insects & other |
| Ochlerotatus varipalpus |  | Insects & other |
| Ochlerotatus ventrovittus |  | Insects & other |
| Ochlerotatus washinoi |  | Insects & other |
| Ochrotrichia alexanderi | A Caddisfly | Insects & other |
| Ochrotrichia alsea | Alsea Ochrotrichian Micro Caddisfly | Insects & other |
| Ochrotrichia argentea |  | Insects & other |
| Ochrotrichia arizonica | A Caddisfly | Insects & other |
| Ochrotrichia buccata | A Caddisfly | Insects & other |
| Ochrotrichia burdicki | A Caddisfly | Insects & other |
| Ochrotrichia dactylophora |  | Insects & other |
| Ochrotrichia hadria | A Caddisfly | Insects & other |
| Ochrotrichia honeyi | A Caddisfly | Insects & other |
| Ochrotrichia ildria |  | Insects & other |
| Ochrotrichia logana | A Caddisfly | Insects & other |
| Ochrotrichia lometa | A Caddisfly | Insects & other |
| Ochrotrichia lucia | A Caddisfly | Insects & other |
| Ochrotrichia mono | A Caddisfly | Insects & other |
| Ochrotrichia nacora | A Caddisfly | Insects & other |
| Ochrotrichia okanoganensis |  | Insects & other |
| Ochrotrichia oregona |  | Insects & other |
| Ochrotrichia phenosa | Deschutes Ochrotrichian Micro Caddisfly | Insects & other |
| Ochrotrichia quadrispina | A Caddisfly | Insects & other |
| Ochrotrichia rothi |  | Insects & other |
| Ochrotrichia salaris | A Caddisfly | Insects & other |
| Ochrotrichia spinulata |  | Insects & other |
| Ochrotrichia stylata | A Caddisfly | Insects & other |
| Ochrotrichia tarsalis |  | Insects & other |
| Ochrotrichia tenuata | A Caddisfly | Insects & other |
| Ochrotrichia trapoiza | A Caddisfly | Insects & other |
| Ochrotrichia vertreesi | Vertrees's Ochrotrichian Micro Caddisfly | Insects & other |
| Ochterus barberi |  | Insects & other |
| Ochterus perbosci |  | Insects & other |
| Ochterus rotundus |  | Insects & other |
| Ochthebius apache |  | Insects & other |
| Ochthebius arenicolus |  | Insects & other |
| Ochthebius arizonicus |  | Insects & other |
| Ochthebius aztecus |  | Insects & other |
| Ochthebius biinicisus |  | Insects & other |
| Ochthebius bisinuatus |  | Insects & other |
| Ochthebius borealis |  | Insects & other |
| Ochthebius brevipennis |  | Insects & other |
| Ochthebius californicus |  | Insects & other |
| Ochthebius costipennis |  | Insects & other |
| Ochthebius crassalus | Wing-shoulder Minute Moss Beetle | Insects & other |
| Ochthebius crenatus |  | Insects & other |
| Ochthebius cribricollis |  | Insects & other |
| Ochthebius discretus |  | Insects & other |
| Ochthebius gruwelli |  | Insects & other |
| Ochthebius interruptus |  | Insects & other |
| Ochthebius lecontei |  | Insects & other |
| Ochthebius leechi |  | Insects & other |
| Ochthebius lineatus |  | Insects & other |
| Ochthebius madrensis |  | Insects & other |
| Ochthebius marinus |  | Insects & other |
| Ochthebius martini |  | Insects & other |
| Ochthebius mimicus |  | Insects & other |
| Ochthebius orbus |  | Insects & other |
| Ochthebius pacificus |  | Insects & other |
| Ochthebius puncticollis |  | Insects & other |
| Ochthebius recticulus | Wilbur Springs Minute Moss Beetle | Insects & other |
| Ochthebius rectus |  | Insects & other |
| Ochthebius rectusalus |  | Insects & other |
| Ochthebius richmondi |  | Insects & other |
| Ochthebius sculptoides |  | Insects & other |
| Ochthebius sculptus |  | Insects & other |
| Ochthebius sierrensis |  | Insects & other |
| Ochthebius similis |  | Insects & other |
| Ochthebius tubus |  | Insects & other |
| Ochthebius uniformis |  | Insects & other |
| Octogomphus specularis | Grappletail | Insects & other |
| Oecetis arizonica |  | Insects & other |
| Oecetis avara | A Caddisfly | Insects & other |
| Oecetis disjuncta | A Caddisfly | Insects & other |
| Oecetis inconspicua | A Caddisfly | Insects & other |
| Oecetis metlacensis |  | Insects & other |
| Oecetis ochracea | A Caddisfly | Insects & other |
| Oemopteryx leei | A Stonefly | Insects & other |
| Oemopteryx vanduzeea | Alpine Willowfly | Insects & other |
| Oenanthe sarmentosa | Water-parsley | Plants |
| Oenothera longissima | Long-stem Evening-primrose | Plants |
| Oligophlebodes minutus |  | Insects & other |
| Oligophlebodes mostbento |  | Insects & other |
| Oligophlebodes ruthae |  | Insects & other |
| Oligophlebodes sierra | A Caddisfly | Insects & other |
| Oligophlebodes sigma |  | Insects & other |
| Onconeura semifimbriata |  | Insects & other |
| Oncorhynchus clarki clarki | Coastal cutthroat trout | Fishes |
| Oncorhynchus clarki henshawi | Lahontan cutthroat trout | Fishes |
| Oncorhynchus clarki seleneris | Paiute cutthroat trout | Fishes |
| Oncorhynchus gorbuscha | Pink salmon | Fishes |
| Oncorhynchus keta | Chum salmon | Fishes |
| Oncorhynchus kisutch - CCC | Central Coast coho salmon | Fishes |
| Oncorhynchus kisutch - SONCC | Southern Oregon Northern California coast coho salmon | Fishes |
| Oncorhynchus mykiss - CCC winter | Central California coast winter steelhead | Fishes |
| Oncorhynchus mykiss - CV | Central Valley steelhead | Fishes |
| Oncorhynchus mykiss - KMP summer | Klamath Mountains Province summer steelhead | Fishes |
| Oncorhynchus mykiss - KMP winter | Klamath Mountains Province winter steelhead | Fishes |
| Oncorhynchus mykiss - NC summer | Northern California coast summer steelhead | Fishes |
| Oncorhynchus mykiss - NC winter | Northern California coast winter steelhead | Fishes |
| Oncorhynchus mykiss - SCCC | South Central California coast steelhead | Fishes |
| Oncorhynchus mykiss - Southern CA | Southern California steelhead | Fishes |
| Oncorhynchus mykiss aguabonita | California golden trout | Fishes |
| Oncorhynchus mykiss aquilarum | Eagle Lake rainbow trout | Fishes |
| Oncorhynchus mykiss gilberti | Kern River rainbow trout | Fishes |
| Oncorhynchus mykiss irideus | Coastal rainbow trout | Fishes |
| Oncorhynchus mykiss ssp. 1 | Goose Lake redband trout | Fishes |
| Oncorhynchus mykiss stonei | McCloud River redband trout | Fishes |
| Oncorhynchus mykiss whitei | Little Kern golden trout | Fishes |
| Oncorhynchus tshawytscha - CCC fall | California Coast fall Chinook salmon | Fishes |
| Oncorhynchus tshawytscha - CV fall | Central Valley fall Chinook salmon | Fishes |
| Oncorhynchus tshawytscha - CV late fall | Central Valley late fall Chinook salmon | Fishes |
| Oncorhynchus tshawytscha - CV spring | Central Valley spring Chinook salmon | Fishes |
| Oncorhynchus tshawytscha - CV winter | Central Valley winter Chinook salmon | Fishes |
| Oncorhynchus tshawytscha - SONCC fall | Southern Oregon Northern California coast fall Chinook salmon | Fishes |
| Oncorhynchus tshawytscha - UKT fall | Upper Klamath-Trinity fall Chinook salmon | Fishes |
| Oncorhynchus tshawytscha - UKT spring | Upper Klamath-Trinity spring Chinook salmon | Fishes |
| Ondatra zibethicus | Common Muskrat | Mammals |
| Onocosmoecus sequoiae | A Caddisfly | Insects & other |
| Onocosmoecus unicolor | A Caddisfly | Insects & other |
| Ophiogomphus arizonicus |  | Insects & other |
| Ophiogomphus bison | Bison Snaketail | Insects & other |
| Ophiogomphus morrisoni | Great Basin Snaketail | Insects & other |
| Ophiogomphus occidentis | Sinuous Snaketail | Insects & other |
| Ophiogomphus severus | Pale Snaketail | Insects & other |
| Oplonaeschna armata |  | Insects & other |
| Optioservus canus | Pinnacles Optioservus Riffle Beetle | Insects & other |
| Optioservus divergens |  | Insects & other |
| Optioservus heteroclitus |  | Insects & other |
| Optioservus quadrimaculatus |  | Insects & other |
| Optioservus seriatus |  | Insects & other |
| Oravelia pege | Dry Creek Cliff Strider Bug | Insects & other |
| Orconectes neglectus neglectus |  | Crustaceans |
| Orcuttia californica | California Orcutt Grass | Plants |
| Orcuttia inaequalis | San Joaquin Valley Orcutt Grass | Plants |
| Orcuttia pilosa | Hairy Orcutt Grass | Plants |
| Orcuttia tenuis | Slender Orcutt Grass | Plants |
| Orcuttia viscida | Sacramento Orcutt Grass | Plants |
| Ordobrevia nubifera |  | Insects & other |
| Oregonasellus elliotti |  | Crustaceans |
| Oreodytes abbreviatus |  | Insects & other |
| Oreodytes angustior |  | Insects & other |
| Oreodytes congruus |  | Insects & other |
| Oreodytes crassulus |  | Insects & other |
| Oreodytes humboltensis |  | Insects & other |
| Oreodytes obesus cordillerensis |  | Insects & other |
| Oreodytes obesus obesus |  | Insects & other |
| Oreodytes picturatus |  | Insects & other |
| Oreodytes quadrimaculatus |  | Insects & other |
| Oreodytes rhyacophilus |  | Insects & other |
| Oreodytes scitulus bisulcatus |  | Insects & other |
| Oreodytes scitulus scitulus |  | Insects & other |
| Oreodytes sierrae |  | Insects & other |
| Oreodytes subrotundus |  | Insects & other |
| Oreoleptis torrenticola |  | Insects & other |
| Oreostemma alpigenum andersonii | Anderson's Tundra Aster | Plants |
| Oreostemma elatum | Plumas Mountaincrown | Plants |
| Oreostemma peirsonii | Peirson's Aster | Plants |
| Oreothlypis luciae | Lucy's Warbler | Birds |
| Orohermes crepusculus |  | Insects & other |
| Oroperla barbara | Gilltail Springfly | Insects & other |
| Orthemis discolor |  | Insects & other |
| Orthemis ferruginea | Roseate Skimmer | Insects & other |
| Orthilia secunda | One-side Wintergreen | Plants |
| Orthocladius appersoni |  | Insects & other |
| Orthocladius carlatus |  | Insects & other |
| Orthocladius dentifer |  | Insects & other |
| Orthocladius dorenus |  | Insects & other |
| Orthocladius dubitatus |  | Insects & other |
| Orthocladius frigidus |  | Insects & other |
| Orthocladius hellenthali |  | Insects & other |
| Orthocladius lignicola |  | Insects & other |
| Orthocladius luteipes |  | Insects & other |
| Orthocladius mallochi |  | Insects & other |
| Orthocladius obumbratus |  | Insects & other |
| Orthocladius oliveri |  | Insects & other |
| Orthocladius rivicola |  | Insects & other |
| Orthocladius rubicundus |  | Insects & other |
| Orthocladius subletti |  | Insects & other |
| Orthodon microlepidotus | Sacramento blackfish | Fishes |
| Orthopodomyia kummi |  | Insects & other |
| Orthopodomyia signifera |  | Insects & other |
| Osobenus yakimae | Yakima Springfly | Insects & other |
| Ostrocerca dimicki |  | Insects & other |
| Ostrocerca foersteri |  | Insects & other |
| Oxyethira aculea |  | Insects & other |
| Oxyethira aeola |  | Insects & other |
| Oxyethira arizona | A Caddisfly | Insects & other |
| Oxyethira dualis | A Caddisfly | Insects & other |
| Oxyethira pallida | A Caddisfly | Insects & other |
| Oxypolis occidentalis | Western Cowbane | Plants |
| Oxyura jamaicensis | Ruddy Duck | Birds |
| Pachydiplax longipennis | Blue Dasher | Insects & other |
| Pacifastacus connectens |  | Crustaceans |
| Pacifastacus fortis | Shasta Crayfish | Crustaceans |
| Pacifastacus gambelii | Pilose Crayfish | Crustaceans |
| Pacifastacus leniusculus klamathensis | Klamath Signal Crayfish | Crustaceans |
| Pacifastacus leniusculus leniusculus | Signal Crayfish | Crustaceans |
| Pacifastacus leniusculus trowbridgii | Columbia River Signal Crayfish | Crustaceans |
| Pacifastacus nigrescens | Sooty Crayfish | Crustaceans |
| Palaeagapetus guppyi |  | Insects & other |
| Palaeagapetus nearcticus | A Caddisfly | Insects & other |
| Palaemnema domina |  | Insects & other |
| Palaemon macrodactylus |  | Crustaceans |
| Palmacorixa buenoi |  | Insects & other |
| Paltothemis lineatipes | Red Rock Skimmer | Insects & other |
| Pandion haliaetus | Osprey | Birds |
| Panicum acuminatum acuminatum |  | Plants |
| Panicum acuminatum fasciculatum |  | Plants |
| Panicum acuminatum lindheimeri |  | Plants |
| Panicum acuminatum thermale |  | Plants |
| Panicum dichotomiflorum | NA | Plants |
| Pantala flavescens | Wandering Glider | Insects & other |
| Pantala hymenaea | Spot-winged Glider | Insects & other |
| Paracapnia baumanni | A Stonefly | Insects & other |
| Paracapnia boris | A Stonefly | Insects & other |
| Paracapnia disala | Dirty Snowfly | Insects & other |
| Paracapnia ensicala |  | Insects & other |
| Paracapnia humboldta | A Stonefly | Insects & other |
| Parachaetocladius imberbus |  | Insects & other |
| Parachironomus abortivus |  | Insects & other |
| Parachironomus chaetaolus |  | Insects & other |
| Parachironomus directus |  | Insects & other |
| Parachironomus frequens |  | Insects & other |
| Parachironomus hazelriggi |  | Insects & other |
| Parachironomus hirtalatus |  | Insects & other |
| Parachironomus tenuicaudatus |  | Insects & other |
| Paracladius conversus |  | Insects & other |
| Paracladopelma alphaeus |  | Insects & other |
| Paracloeodes minutus | A Small Minnow Mayfly | Insects & other |
| Paracoenia calida | Wilber Springs Shore Fly | Insects & other |
| Paracymus communis |  | Insects & other |
| Paracymus confusus |  | Insects & other |
| Paracymus elegans |  | Insects & other |
| Paracymus ellipsis |  | Insects & other |
| Paracymus restrictus |  | Insects & other |
| Paracymus subcupreus |  | Insects & other |
| Paracymus tarsalis |  | Insects & other |
| Parakiefferiella subaterrima |  | Insects & other |
| Paralauterborniella nigrohalteris |  | Insects & other |
| Paraleptophlebia altana | A Mayfly | Insects & other |
| Paraleptophlebia aquilina |  | Insects & other |
| Paraleptophlebia associata | A Mayfly | Insects & other |
| Paraleptophlebia bicornuta |  | Insects & other |
| Paraleptophlebia brunneipennis |  | Insects & other |
| Paraleptophlebia cachea | A Mayfly | Insects & other |
| Paraleptophlebia californica | A Mayfly | Insects & other |
| Paraleptophlebia clara | A Mayfly | Insects & other |
| Paraleptophlebia debilis | A Mayfly | Insects & other |
| Paraleptophlebia falcula |  | Insects & other |
| Paraleptophlebia gregalis | A Mayfly | Insects & other |
| Paraleptophlebia helena | A Mayfly | Insects & other |
| Paraleptophlebia heteronea | A Mayfly | Insects & other |
| Paraleptophlebia memorialis | A Mayfly | Insects & other |
| Paraleptophlebia packii | A Mayfly | Insects & other |
| Paraleptophlebia placeri | A Mayfly | Insects & other |
| Paraleptophlebia quisquilia | A Mayfly | Insects & other |
| Paraleptophlebia rufivenosa | A Mayfly | Insects & other |
| Paraleptophlebia sculleni |  | Insects & other |
| Paraleptophlebia temporalis | A Mayfly | Insects & other |
| Paraleptophlebia vaciva | A Mayfly | Insects & other |
| Paraleptophlebia zayante | A Mayfly | Insects & other |
| Paraleuctra divisa | California Needlefly | Insects & other |
| Paraleuctra forcipata | Bullshorn Needlefly | Insects & other |
| Paraleuctra occidentalis | Western Needlefly | Insects & other |
| Paraleuctra projecta |  | Insects & other |
| Paraleuctra vershina | Summit Needlefly | Insects & other |
| Paramerina fragilis |  | Insects & other |
| Paramerina smithae |  | Insects & other |
| Parametriocnemus lundbeckii |  | Insects & other |
| Paraperla frontalis | Hyporheic Sallfly | Insects & other |
| Paraperla wilsoni | Chilliwack Sallfly | Insects & other |
| Paraphaenocladius exagitans |  | Insects & other |
| Paraphaenocladius innasus |  | Insects & other |
| Parapholis strigosa | NA | Plants |
| Parapsyche almota | A Caddisfly | Insects & other |
| Parapsyche elsis | A Caddisfly | Insects & other |
| Parapsyche extensa | King's Creek Parapsyche Caddisfly | Insects & other |
| Parapsyche spinata | A Caddisfly | Insects & other |
| Parapsyche turbinata | A Caddisfly | Insects & other |
| Parasimulium crosskeyi |  | Insects & other |
| Parasimulium furcatum |  | Insects & other |
| Parasimulium species |  | Insects & other |
| Parasimulium stonei |  | Insects & other |
| Paratanytarsus grimmii |  | Insects & other |
| Paratendipes albimanus |  | Insects & other |
| Paratendipes basidens |  | Insects & other |
| Paratendipes fuscitibia |  | Insects & other |
| Paratendipes subaequalis |  | Insects & other |
| Paratendipes thermophilus |  | Insects & other |
| Paratrichocladius rufiventris |  | Insects & other |
| Parnassia cirrata cirrata | Fringed Grass-of-Parnassus | Plants |
| Parnassia cirrata intermedia |  | Plants |
| Parnassia fimbriata fimbriata | Fringed Grass-of-Parnassus | Plants |
| Parnassia palustris | Marsh Grass-of-Parnassus | Plants |
| Parnassia parviflora | Small-flower Grass-of-parnassus | Plants |
| Parochlus kiefferi |  | Insects & other |
| Parthina linea | A Caddisfly | Insects & other |
| Parthina vierra | A Caddisfly | Insects & other |
| Paspalum distichum | Joint Paspalum | Plants |
| Patapius spinosus |  | Insects & other |
| Pectiantia ovalis | NA | Plants |
| Pectiantia pentandra |  | Plants |
| Pedicularis attollens | NA | Plants |
| Pedicularis groenlandica | NA | Plants |
| Pedomoecus sierra | A Caddisfly | Insects & other |
| Pelecanus erythrorhynchos | American White Pelican | Birds |
| Pelocoris biimpressus |  | Insects & other |
| Peltodytes callosus |  | Insects & other |
| Peltodytes dispersus |  | Insects & other |
| Peltodytes mexicanus |  | Insects & other |
| Peltodytes simplex |  | Insects & other |
| Pentacora saratogae |  | Insects & other |
| Pentacora signoreti |  | Insects & other |
| Pentacora sphacelata |  | Insects & other |
| Pentaneura inconspicua |  | Insects & other |
| Pentaneura inyoensis |  | Insects & other |
| Perideridia bacigalupii | Bacigalupi's Perideridia | Plants |
| Perideridia bolanderi bolanderi | Bolander's Yampah | Plants |
| Perideridia bolanderi involucrata | Bolander's Yampah | Plants |
| Perideridia californica | California Yampah | Plants |
| Perideridia gairdneri borealis | Gairdner's Yampah | Plants |
| Perideridia gairdneri gairdneri | Gairdner's Yampah | Plants |
| Perideridia howellii | Howell's False Caraway | Plants |
| Perideridia kelloggii | Kellogg's Yampah | Plants |
| Perideridia lemmonii | Lemmon's Yampah | Plants |
| Perideridia leptocarpa | Narrow-seeded Yampah | Plants |
| Perideridia oregana | Oregon Yampah | Plants |
| Perideridia parishii latifolia | Parish's Yampah | Plants |
| Perideridia parishii parishii | Parish's Yampah | Plants |
| Perideridia pringlei | Pringle's Yampah | Plants |
| Perithemis domitia |  | Insects & other |
| Perithemis intensa | Mexican Amberwing | Insects & other |
| Perithemis tenera |  | Insects & other |
| Perlinodes aurea | Longgill Springfly | Insects & other |
| Perlomyia collaris | Black Needlefly | Insects & other |
| Perlomyia utahensis | Utah Needlefly | Insects & other |
| Persicaria amphibia |  | Plants |
| Persicaria hydropiper | NA | Plants |
| Persicaria hydropiperoides |  | Plants |
| Persicaria lapathifolia |  | Plants |
| Persicaria maculosa | NA | Plants |
| Persicaria orientalis | NA | Plants |
| Persicaria pensylvanica | NA | Plants |
| Persicaria punctata | NA | Plants |
| Persicaria wallichii | NA | Plants |
| Petrophila confusalis |  | Insects & other |
| Petrophila jaliscalis |  | Insects & other |
| Petrophila kearfottalis |  | Insects & other |
| Phacelia distans | NA | Plants |
| Phaenopsectra dyari |  | Insects & other |
| Phaenopsectra flavipes |  | Insects & other |
| Phaenopsectra mortensoni |  | Insects & other |
| Phaenopsectra pilicellata |  | Insects & other |
| Phaenopsectra profusa |  | Insects & other |
| Phalacrocorax auritus | Double-crested Cormorant | Birds |
| Phalacroseris bolanderi | NA | Plants |
| Phalaris arundinacea | Reed Canarygrass | Plants |
| Phalaropus tricolor | Wilson's Phalarope | Birds |
| Philarctus bergrothi |  | Insects & other |
| Philocasca demita |  | Insects & other |
| Philocasca oron |  | Insects & other |
| Philocasca rivularis | A Caddisfly | Insects & other |
| Philorus californica | A Net-winged Midge | Insects & other |
| Philorus jacinto | A Net-winged Midge | Insects & other |
| Philorus vanduzeei | A Net-winged Midge | Insects & other |
| Philorus yosemite | A Net-winged Midge | Insects & other |
| Phragmites australis australis | Common Reed | Plants |
| Phreatobrachypoda robusta |  | Insects & other |
| Phryganea cinerea | A Caddisfly | Insects & other |
| Phyla lanceolata | Fog-fruit | Plants |
| Phyla nodiflora | Common Frog-fruit | Plants |
| Phylloicus aeneus |  | Insects & other |
| Phylloicus mexicanus |  | Insects & other |
| Phyllospadix scouleri | Scouler's Surf-grass | Plants |
| Phyllospadix torreyi | Torrey's Surf-grass | Plants |
| Physa acuta | Pewter Physa | Mollusks |
| Physa gyrina | Tadpole Physa | Mollusks |
| Physella boucardi | Desert Physa | Mollusks |
| Physella cooperi | Olive Physa | Mollusks |
| Physella costata | Ornate Physa | Mollusks |
| Physella humerosa | Corkscrew Physa | Mollusks |
| Physella lordi | Twisted Physa | Mollusks |
| Physella osculans | Cayuse Physa | Mollusks |
| Physella propinqua | Rocky Mountain Physa | Mollusks |
| Physella traski | Sculpted Physa | Mollusks |
| Physella virgata | Protean Physa | Mollusks |
| Physella virginea | Sunset Physa | Mollusks |
| Physemus minutus |  | Insects & other |
| Pilularia americana | NA | Plants |
| Pinguicula macroceras | NA | Plants |
| Pipilo aberti | Abert's Towhee | Birds |
| Pipilo crissalis eremophilus | Inyo California Towhee | Birds |
| Piranga rubra | Summer Tanager | Birds |
| Pisidium casertanum |  | Mollusks |
| Pisidium compressum |  | Mollusks |
| Pisidium idahoense |  | Mollusks |
| Pisidium lilljeborgi |  | Mollusks |
| Pisidium nitidum |  | Mollusks |
| Pisidium subtruncatum |  | Mollusks |
| Pisidium ultramontanum | Montane Peaclam | Mollusks |
| Pisidium variabile |  | Mollusks |
| Pisidium walkeri |  | Mollusks |
| Plagiobothrys acanthocarpus | Adobe Popcorn-flower | Plants |
| Plagiobothrys austiniae | Austin's Popcorn-flower | Plants |
| Plagiobothrys chorisianus | NA | Plants |
| Plagiobothrys distantiflorus | California Popcorn-flower | Plants |
| Plagiobothrys glaber | Hairless Allocarya | Plants |
| Plagiobothrys greenei | Greene's Popcorn-flower | Plants |
| Plagiobothrys humistratus | Dwarf Popcorn-flower | Plants |
| Plagiobothrys leptocladus | Alkali Popcorn-flower | Plants |
| Plagiobothrys nitens |  | Plants |
| Plagiobothrys parishii | Parish's Popcorn-flower | Plants |
| Plagiobothrys reticulatus reticulatus |  | Plants |
| Plagiobothrys reticulatus rossianorum |  | Plants |
| Plagiobothrys tener | NA | Plants |
| Plagiobothrys undulatus | NA | Plants |
| Planorbella binneyi | Coarse Rams-horn | Mollusks |
| Planorbella occidentalis | Fine-lined Rams-horn | Mollusks |
| Planorbella subcrenata | Rough Rams-horn | Mollusks |
| Planorbella tenuis | Mexican Rams-horn | Mollusks |
| Planorbella traski | Keeled Rams-horn | Mollusks |
| Planorbella trivolvis | Marsh Rams-horn | Mollusks |
| Plantago elongata elongata | Slender Plantain | Plants |
| Platanthera dilatata leucostachys |  | Plants |
| Platanthera sparsiflora sparsiflora | Canyon Bog Orchid | Plants |
| Platanthera stricta | Slender Bog Orchid | Plants |
| Platanthera tescamnis | NA | Plants |
| Platanthera yosemitensis | Yosemite Bog-Orchid | Plants |
| Platanus racemosa | California Sycamore | Plants |
| Plathemis lydia | Common Whitetail | Insects & other |
| Plathemis subornata | Desert Whitetail | Insects & other |
| Platyhydracarus juliani |  | Insects & other |
| Platyhydracarus parvipalpis |  | Insects & other |
| Platyvelia beameri |  | Insects & other |
| Platyvelia brachialis |  | Insects & other |
| Platyvelia summersi |  | Insects & other |
| Plauditus punctiventris |  | Insects & other |
| Plegadis chihi | White-faced Ibis | Birds |
| Plethodon dunni | Dunn's Salamander | Herps |
| Pleuropogon californicus californicus |  | Plants |
| Pleuropogon californicus davyi |  | Plants |
| Pleuropogon hooverianus | North Coast False Semaphore Grass | Plants |
| Pleuropogon refractus | Nodding False Semaphore Grass | Plants |
| Pluchea odorata odorata | Scented Conyza | Plants |
| Pluchea sericea | Arrow-weed | Plants |
| Plumiperla diversa | Margined Sallfly | Insects & other |
| Plumiperla spinosa | Spiny Sallfly | Insects & other |
| Pluvialis squatarola | Black-bellied Plover | Birds |
| Podiceps nigricollis | Eared Grebe | Birds |
| Podilymbus podiceps | Pied-billed Grebe | Birds |
| Podmosta decepta |  | Insects & other |
| Podmosta delicatula | Delicate Forestfly | Insects & other |
| Podmosta obscura |  | Insects & other |
| Pogogyne abramsii | San Diego Mesamint | Plants |
| Pogogyne douglasii | NA | Plants |
| Pogogyne floribunda | Profuse-flowered Pogogyne | Plants |
| Pogogyne nudiuscula | Otay Mesamint | Plants |
| Pogogyne zizyphoroides |  | Plants |
| Pogonichthys ciscoides | Clear Lake Splittail | Fishes |
| Pogonichthys macrolepidotus | Sacramento splittail | Fishes |
| Polycentropus arizonensis |  | Insects & other |
| Polycentropus aztecus |  | Insects & other |
| Polycentropus cinereus |  | Insects & other |
| Polycentropus denningi |  | Insects & other |
| Polycentropus flavus | A Caddisfly | Insects & other |
| Polycentropus gertschi |  | Insects & other |
| Polycentropus halidus | A Caddisfly | Insects & other |
| Polycentropus variegatus | A Caddisfly | Insects & other |
| Polygonum marinense | Marin Knotweed | Plants |
| Polypedilum albicorne |  | Insects & other |
| Polypedilum albinodus |  | Insects & other |
| Polypedilum angustum |  | Insects & other |
| Polypedilum apicatum |  | Insects & other |
| Polypedilum artifer |  | Insects & other |
| Polypedilum aviceps |  | Insects & other |
| Polypedilum braseniae |  | Insects & other |
| Polypedilum californicum |  | Insects & other |
| Polypedilum cinctum |  | Insects & other |
| Polypedilum cultellatum |  | Insects & other |
| Polypedilum digitifer |  | Insects & other |
| Polypedilum halterale |  | Insects & other |
| Polypedilum illinoense |  | Insects & other |
| Polypedilum isocerus |  | Insects & other |
| Polypedilum labeculosum |  | Insects & other |
| Polypedilum laetum |  | Insects & other |
| Polypedilum obelos |  | Insects & other |
| Polypedilum ophioides |  | Insects & other |
| Polypedilum parvum |  | Insects & other |
| Polypedilum pedatum |  | Insects & other |
| Polypedilum pterospilus |  | Insects & other |
| Polypedilum scalaenum |  | Insects & other |
| Polypedilum sulaceps |  | Insects & other |
| Polypedilum trigonus |  | Insects & other |
| Polypedilum tritum |  | Insects & other |
| Polypedilum vibex |  | Insects & other |
| Polyplectropus charlesi |  | Insects & other |
| Pomacea bridgesii |  | Mollusks |
| Pomacea paludosa |  | Mollusks |
| Pomatiopsis binneyi | Robust Walker | Mollusks |
| Pomatiopsis californica | Pacific Walker | Mollusks |
| Pomatiopsis chacei | Marsh Walker | Mollusks |
| Pomoleuctra andersoni | Oregon Needlefly | Insects & other |
| Pomoleuctra purcellana |  | Insects & other |
| Populus trichocarpa | NA | Plants |
| Porterella carnosula | Western Porterella | Plants |
| Porzana carolina | Sora | Birds |
| Postelichus confluentus |  | Insects & other |
| Postelichus immsi |  | Insects & other |
| Postelichus productus |  | Insects & other |
| Potamogeton alpinus | Northern Pondweed | Plants |
| Potamogeton amplifolius | Largeleaf Pondweed | Plants |
| Potamogeton berchtoldii | NA | Plants |
| Potamogeton diversifolius | Water-thread Pondweed | Plants |
| Potamogeton epihydrus | Nuttall's Pondweed | Plants |
| Potamogeton foliosus fibrillosus | Fibrous Pondweed | Plants |
| Potamogeton foliosus foliosus | Leafy Pondweed | Plants |
| Potamogeton gramineus | Grassy Pondweed | Plants |
| Potamogeton illinoensis | Illinois Pondweed | Plants |
| Potamogeton natans | Floating Pondweed | Plants |
| Potamogeton nodosus | Longleaf Pondweed | Plants |
| Potamogeton praelongus | White-stem Pondweed | Plants |
| Potamogeton pusillus pusillus | Slender Pondweed | Plants |
| Potamogeton richardsonii | Richardson's Pondweed | Plants |
| Potamogeton robbinsii | Flatleaf Pondweed | Plants |
| Potamogeton zosteriformis | Flatstem Pondweed | Plants |
| Potentilla anserina anserina |  | Plants |
| Potentilla anserina pacifica |  | Plants |
| Potentilla multijuga | Ballona Cinquefoil | Plants |
| Potentilla newberryi | Newberry's Cinquefoil | Plants |
| Potentilla uliginosa | Cunningham Marsh cinquefoil | Plants |
| Primula jeffreyi |  | Plants |
| Primula pauciflora |  | Plants |
| Primula subalpina |  | Plants |
| Primula tetrandra | NA | Plants |
| Prionocera oregonica |  | Insects & other |
| Pristinicola hemphilli | Pristine Pyrg | Mollusks |
| Procladius barbatulus |  | Insects & other |
| Procladius bellus |  | Insects & other |
| Procladius culiciformis |  | Insects & other |
| Procladius denticulatus |  | Insects & other |
| Procladius freemani |  | Insects & other |
| Procladius sublettei |  | Insects & other |
| Procloeon pennulatum | A Mayfly | Insects & other |
| Procloeon rivulare | A Mayfly | Insects & other |
| Procloeon venosum | A Mayfly | Insects & other |
| Progomphus borealis | Gray Sanddragon | Insects & other |
| Promenetus exacuous | Sharp Sprite | Mollusks |
| Promenetus umbilicatellus | Umbilicate Sprite | Mollusks |
| Prosimulium caudatum |  | Insects & other |
| Prosimulium constrictistylum |  | Insects & other |
| Prosimulium davesi |  | Insects & other |
| Prosimulium dicentum |  | Insects & other |
| Prosimulium dicum |  | Insects & other |
| Prosimulium esselbaughi |  | Insects & other |
| Prosimulium exigens |  | Insects & other |
| Prosimulium flaviantennus |  | Insects & other |
| Prosimulium formosum |  | Insects & other |
| Prosimulium frohnei |  | Insects & other |
| Prosimulium fulvithorax |  | Insects & other |
| Prosimulium fulvum |  | Insects & other |
| Prosimulium idemai |  | Insects & other |
| Prosimulium imposter |  | Insects & other |
| Prosimulium longirostrum |  | Insects & other |
| Prosimulium minifulvum |  | Insects & other |
| Prosimulium rusticum |  | Insects & other |
| Prosimulium secretum |  | Insects & other |
| Prosimulium shewelli |  | Insects & other |
| Prosimulium travisi |  | Insects & other |
| Prosimulium uinta |  | Insects & other |
| Prosimulium unicum |  | Insects & other |
| Prosopium williamsoni | Mountain whitefish | Fishes |
| Prostoia besametsa | Bended Forestfly | Insects & other |
| Protanyderus margarita |  | Insects & other |
| Protanyderus vanduzeei |  | Insects & other |
| Protanyderus vipio |  | Insects & other |
| Protochauliodes aridus |  | Insects & other |
| Protochauliodes cascadius |  | Insects & other |
| Protochauliodes minimus |  | Insects & other |
| Protochauliodes montivagus |  | Insects & other |
| Protochauliodes simplus |  | Insects & other |
| Protochauliodes spenceri |  | Insects & other |
| Protoptila balmorhea |  | Insects & other |
| Protoptila coloma | A Caddisfly | Insects & other |
| Protoptila erotica |  | Insects & other |
| Psectrocladius barbimanus |  | Insects & other |
| Psectrocladius spinifer |  | Insects & other |
| Psectrocladius vernalis |  | Insects & other |
| Psectrotanypus dyari |  | Insects & other |
| Psephenus arizonensis |  | Insects & other |
| Psephenus falli |  | Insects & other |
| Psephenus minckleyi |  | Insects & other |
| Psephenus montanus |  | Insects & other |
| Psephenus murvoshi |  | Insects & other |
| Pseudacris cadaverina | California Treefrog | Herps |
| Pseudacris hypochondriaca | Baja California Treefrog | Herps |
| Pseudacris regilla | Northern Pacific Chorus Frog | Herps |
| Pseudacris sierra | Sierran Treefrog | Herps |
| Pseudiron centralis | White Sand-river Mayfly | Insects & other |
| Pseudochironomus richardsoni |  | Insects & other |
| Pseudocloeon apache |  | Insects & other |
| Pseudocloeon propinquum | A Mayfly | Insects & other |
| Pseudocorixa beameri |  | Insects & other |
| Pseudodiamesa branickii |  | Insects & other |
| Pseudoleon superbus |  | Insects & other |
| Pseudorthocladius dumicaudus |  | Insects & other |
| Pseudorthocladius uniserratus |  | Insects & other |
| Pseudosmittia forcipata |  | Insects & other |
| Pseudosmittia nanseni |  | Insects & other |
| Pseudostenophylax edwardsi | A Caddisfly | Insects & other |
| Psilocarphus brevissimus brevissimus | Dwarf Woolly-heads | Plants |
| Psilocarphus brevissimus multiflorus | Delta Woolly Marbles | Plants |
| Psilocarphus oregonus | Oregon Woolly-heads | Plants |
| Psilocarphus tenellus | NA | Plants |
| Psorophora columbiae |  | Insects & other |
| Psorophora discolor |  | Insects & other |
| Psorophora howardii |  | Insects & other |
| Psorophora signipennis |  | Insects & other |
| Psychoglypha alascensis |  | Insects & other |
| Psychoglypha avigo | A Caddisfly | Insects & other |
| Psychoglypha bella | A Caddisfly | Insects & other |
| Psychoglypha browni |  | Insects & other |
| Psychoglypha klamathi | A Caddisfly | Insects & other |
| Psychoglypha leechi | A Caddisfly | Insects & other |
| Psychoglypha mazamae | A Caddisfly | Insects & other |
| Psychoglypha ormiae | A Caddisfly | Insects & other |
| Psychoglypha prita |  | Insects & other |
| Psychoglypha schuhi |  | Insects & other |
| Psychoglypha subborealis | A Caddisfly | Insects & other |
| Psychomyia flavida | A Caddisfly | Insects & other |
| Psychomyia lumina | A Caddisfly | Insects & other |
| Psychomyia nomada |  | Insects & other |
| Pteronarcella badia |  | Insects & other |
| Pteronarcella regularis | Dwarf Salmonfly | Insects & other |
| Pteronarcys californica | Giant Salmonfly | Insects & other |
| Pteronarcys princeps | Ebony Salmonfly | Insects & other |
| Ptychocheilus grandis | Sacramento pikeminnow | Fishes |
| Ptychocheilus lucius | Colorado Pikeminnow | Fishes |
| Ptychoptera byersi |  | Insects & other |
| Ptychoptera lenis |  | Insects & other |
| Ptychoptera minor |  | Insects & other |
| Ptychoptera monoensis |  | Insects & other |
| Ptychoptera pendula |  | Insects & other |
| Ptychoptera sculleni |  | Insects & other |
| Ptychoptera townesi |  | Insects & other |
| Puccinellia howellii | Trinity Mountains Alkali Grass | Plants |
| Puccinellia nutkaensis | Alaska Alkaligrass | Plants |
| Puccinellia nuttalliana | Nuttall's Alkali Grass | Plants |
| Puccinellia parishii | Parish's Alkali Grass | Plants |
| Puccinellia pumila |  | Plants |
| Puccinellia simplex | Little Alkali Grass | Plants |
| Pyrgulopsis aardahli | Benton Valley Springsnail | Mollusks |
| Pyrgulopsis amargosae | Amargosa Springsnail | Mollusks |
| Pyrgulopsis archimedis | Archimedes Pyrg | Mollusks |
| Pyrgulopsis californiensis | Laguna Mountain Springsnail | Mollusks |
| Pyrgulopsis castaicensis | A Freshwater Snail | Mollusks |
| Pyrgulopsis cinerana | Ash Valley Pyrg | Mollusks |
| Pyrgulopsis diablensis | Diablo Range Pyrg | Mollusks |
| Pyrgulopsis eremica | Smoke Creek Pyrg | Mollusks |
| Pyrgulopsis falciglans | Likely Pyrg | Mollusks |
| Pyrgulopsis gibba | Surprise Valley Pyrg | Mollusks |
| Pyrgulopsis giuliani | Southern Sierra Nevada Springsnail | Mollusks |
| Pyrgulopsis greggi | Kern River Pyrg | Mollusks |
| Pyrgulopsis intermedia | Crooked Creek Springsnail | Mollusks |
| Pyrgulopsis lasseni | Willow Creek Pyrg | Mollusks |
| Pyrgulopsis licina |  | Mollusks |
| Pyrgulopsis longae | Long Valley Pyrg | Mollusks |
| Pyrgulopsis longinqua | Salton Sea Springsnail | Mollusks |
| Pyrgulopsis micrococcus | Oasis Valley Springsnail | Mollusks |
| Pyrgulopsis milleri | A Freshwater Snail | Mollusks |
| Pyrgulopsis owensensis | Owens Valley Springsnail | Mollusks |
| Pyrgulopsis perforata |  | Mollusks |
| Pyrgulopsis perturbata | Fish Slough Springsnail | Mollusks |
| Pyrgulopsis rupinicola | Sucker Springs Pyrg | Mollusks |
| Pyrgulopsis sanchezi |  | Mollusks |
| Pyrgulopsis stearnsiana | Yaqui Springsnail | Mollusks |
| Pyrgulopsis taylori | San Luis Obispo Pyrg | Mollusks |
| Pyrgulopsis turbatrix | Southeast Nevada Pyrg | Mollusks |
| Pyrgulopsis ventricosa | Clear Lake Pyrg | Mollusks |
| Pyrgulopsis wongi | Wong's Springsnail | Mollusks |
| Radotanypus submarginella |  | Insects & other |
| Rallus limicola | Virginia Rail | Birds |
| Rallus longirostris yumanensis | Yuma Clapper Rail | Birds |
| Ramellogammarus californicus |  | Crustaceans |
| Ramellogammarus campestris |  | Crustaceans |
| Ramellogammarus columbianus |  | Crustaceans |
| Ramellogammarus littoralis |  | Crustaceans |
| Ramellogammarus oregonensis |  | Crustaceans |
| Ramellogammarus ramellus |  | Crustaceans |
| Ramellogammarus similimanus |  | Crustaceans |
| Ramphocorixa rotundocephala |  | Insects & other |
| Rana aurora | Northern Red-legged Frog | Herps |
| Rana boylii | Foothill Yellow-legged Frog | Herps |
| Rana cascadae | Cascades Frog | Herps |
| Rana draytonii | California Red-legged Frog | Herps |
| Rana muscosa | Southern Mountain Yellow-legged Frog | Herps |
| Rana pretiosa | Oregon Spotted Frog | Herps |
| Rana sierrae | Sierra Nevada Yellow-legged Frog | Herps |
| Ranatra brevicollis | A Water Scorpion | Insects & other |
| Ranatra fusca |  | Insects & other |
| Ranatra montezuma |  | Insects & other |
| Ranatra quadridentata |  | Insects & other |
| Ranunculus alismifolius alismellus | Water-plantain Buttercup | Plants |
| Ranunculus alismifolius alismifolius | Water-plantain Buttercup | Plants |
| Ranunculus alismifolius hartwegii |  | Plants |
| Ranunculus alismifolius lemmonii |  | Plants |
| Ranunculus andersonii andersonii | Anderson's Buttercup | Plants |
| Ranunculus aquatilis aquatilis | White Water Buttercup | Plants |
| Ranunculus aquatilis diffusus |  | Plants |
| Ranunculus bonariensis | NA | Plants |
| Ranunculus flabellaris | Yellow Water-crowfoot | Plants |
| Ranunculus flammula flammula | Lesser Spearwort | Plants |
| Ranunculus flammula ovalis |  | Plants |
| Ranunculus hydrocharoides | NA | Plants |
| Ranunculus hystriculus |  | Plants |
| Ranunculus lobbii | Lobb's Water Buttercup | Plants |
| Ranunculus macounii | Macoun's Buttercup | Plants |
| Ranunculus populago | Mountain Buttercup | Plants |
| Ranunculus pusillus pusillus | Pursh's Buttercup | Plants |
| Ranunculus repens | NA | Plants |
| Ranunculus sardous | NA | Plants |
| Ranunculus sceleratus | NA | Plants |
| Recurvirostra americana | American Avocet | Birds |
| Remartinia luteipennis |  | Insects & other |
| Reomyia wartinbei |  | Insects & other |
| Rhagovelia becki |  | Insects & other |
| Rhagovelia choreutes |  | Insects & other |
| Rhagovelia distincta |  | Insects & other |
| Rhagovelia varipes |  | Insects & other |
| Rhamnus alnifolia | Alderleaf Buckthorn | Plants |
| Rhantus anisonychus |  | Insects & other |
| Rhantus atricolor |  | Insects & other |
| Rhantus binotatus |  | Insects & other |
| Rhantus consimilis |  | Insects & other |
| Rhantus gutticollis |  | Insects & other |
| Rhantus sericans |  | Insects & other |
| Rhantus wallisi |  | Insects & other |
| Rheotanytarsus hamatus |  | Insects & other |
| Rheumatobates hungerfordi |  | Insects & other |
| Rhinichthys osculus klamathensis | Klamath speckled dace | Fishes |
| Rhinichthys osculus nevadensis | Amargosa Canyon speckled dace | Fishes |
| Rhinichthys osculus robustus | Lahontan speckled dace | Fishes |
| Rhinichthys osculus ssp. 1 | Sacramento speckled dace | Fishes |
| Rhinichthys osculus ssp. 2 | Owens speckled dace | Fishes |
| Rhinichthys osculus ssp. 3 | Long Valley speckled dace | Fishes |
| Rhinichthys osculus ssp. 4 | Santa Ana speckled dace | Fishes |
| Rhionaeschna californica | California Darner | Insects & other |
| Rhionaeschna multicolor | Blue-eyed Darner | Insects & other |
| Rhionaeshna dugesi |  | Insects & other |
| Rhionaeshna psillus |  | Insects & other |
| Rhithrogena decora | A Mayfly | Insects & other |
| Rhithrogena flavianula | A Mayfly | Insects & other |
| Rhithrogena hageni | A Mayfly | Insects & other |
| Rhithrogena morrisoni | A Mayfly | Insects & other |
| Rhithrogena plana | A Mayfly | Insects & other |
| Rhithrogena robusta | A Mayfly | Insects & other |
| Rhithrogena undulata | A Mayfly | Insects & other |
| Rhithrogena virilis |  | Insects & other |
| Rhizelmis nigra |  | Insects & other |
| Rhododendron columbianum |  | Plants |
| Rhododendron occidentale occidentale | Western Azalea | Plants |
| Rhyacophila acuminata | A Caddisfly | Insects & other |
| Rhyacophila alberta |  | Insects & other |
| Rhyacophila amabilis | Castle Lake Rhyacophilan Caddisfly | Insects & other |
| Rhyacophila angelita | A Caddisfly | Insects & other |
| Rhyacophila arcella | A Caddisfly | Insects & other |
| Rhyacophila ardala | A Caddisfly | Insects & other |
| Rhyacophila arnaudi | A Caddisfly | Insects & other |
| Rhyacophila balosa | A Caddisfly | Insects & other |
| Rhyacophila basalis | A Caddisfly | Insects & other |
| Rhyacophila betteni | A Caddisfly | Insects & other |
| Rhyacophila bifila | A Caddisfly | Insects & other |
| Rhyacophila blarina |  | Insects & other |
| Rhyacophila californica | A Caddisfly | Insects & other |
| Rhyacophila cerita | A Caddisfly | Insects & other |
| Rhyacophila chandleri | A Caddisfly | Insects & other |
| Rhyacophila chilsia |  | Insects & other |
| Rhyacophila chordata | A Caddisfly | Insects & other |
| Rhyacophila colonus | Obrien Rhyacophilan Caddisfly | Insects & other |
| Rhyacophila coloradensis | A Caddisfly | Insects & other |
| Rhyacophila darbyi | A Caddisfly | Insects & other |
| Rhyacophila ebria |  | Insects & other |
| Rhyacophila ecosa | A Caddisfly | Insects & other |
| Rhyacophila fenderi | Fender's Rhyacophilan Caddisfly | Insects & other |
| Rhyacophila grandis | A Caddisfly | Insects & other |
| Rhyacophila haddocki |  | Insects & other |
| Rhyacophila harmstoni | A Caddisfly | Insects & other |
| Rhyacophila hyalinata | A Caddisfly | Insects & other |
| Rhyacophila inculta | A Caddisfly | Insects & other |
| Rhyacophila insularis | A Caddisfly | Insects & other |
| Rhyacophila iranda |  | Insects & other |
| Rhyacophila jenniferae | A Caddisfly | Insects & other |
| Rhyacophila jewetti | A Caddisfly | Insects & other |
| Rhyacophila karila | A Caddisfly | Insects & other |
| Rhyacophila kernada | A Caddisfly | Insects & other |
| Rhyacophila kincaidi |  | Insects & other |
| Rhyacophila leechi | A Caddisfly | Insects & other |
| Rhyacophila lineata | Castle Crags Rhyacophilan Caddisfly | Insects & other |
| Rhyacophila lurella | A Caddisfly | Insects & other |
| Rhyacophila malkini |  | Insects & other |
| Rhyacophila mosana | Bilobed Rhyacophilan Caddisfly | Insects & other |
| Rhyacophila narvae | A Caddisfly | Insects & other |
| Rhyacophila neograndis | A Caddisfly | Insects & other |
| Rhyacophila nevadensis | A Caddisfly | Insects & other |
| Rhyacophila norcuta | A Caddisfly | Insects & other |
| Rhyacophila oreta | A Caddisfly | Insects & other |
| Rhyacophila pellisa | A Caddisfly | Insects & other |
| Rhyacophila perda |  | Insects & other |
| Rhyacophila perplana |  | Insects & other |
| Rhyacophila pichaca |  | Insects & other |
| Rhyacophila rayneri | A Caddisfly | Insects & other |
| Rhyacophila reyesi | A Caddisfly | Insects & other |
| Rhyacophila rotunda | A Caddisfly | Insects & other |
| Rhyacophila sequoia | A Caddisfly | Insects & other |
| Rhyacophila sierra | A Caddisfly | Insects & other |
| Rhyacophila siskiyou | A Caddisfly | Insects & other |
| Rhyacophila spinata | Spiny Rhyacophilan Caddisfly | Insects & other |
| Rhyacophila starki | A Caddisfly | Insects & other |
| Rhyacophila tamalpaisi | A Caddisfly | Insects & other |
| Rhyacophila tehama | A Caddisfly | Insects & other |
| Rhyacophila tralala |  | Insects & other |
| Rhyacophila tucula | A Caddisfly | Insects & other |
| Rhyacophila unipunctata |  | Insects & other |
| Rhyacophila vaccua | A Caddisfly | Insects & other |
| Rhyacophila vaefes | A Caddisfly | Insects & other |
| Rhyacophila vagrita |  | Insects & other |
| Rhyacophila valuma | A Caddisfly | Insects & other |
| Rhyacophila vao | A Caddisfly | Insects & other |
| Rhyacophila vedra | A Caddisfly | Insects & other |
| Rhyacophila velora | A Caddisfly | Insects & other |
| Rhyacophila vemna |  | Insects & other |
| Rhyacophila verrula | A Caddisfly | Insects & other |
| Rhyacophila vetina |  | Insects & other |
| Rhyacophila viquaea |  | Insects & other |
| Rhyacophila visor |  | Insects & other |
| Rhyacophila vobara |  | Insects & other |
| Rhyacophila vocala | A Caddisfly | Insects & other |
| Rhyacophila vuzana | A Caddisfly | Insects & other |
| Rhyacophila willametta |  | Insects & other |
| Rhyacotriton variegatus | Southern Torrent Salamander | Herps |
| Rhynchospora alba | White Beakrush | Plants |
| Rhynchospora californica | California Beakrush | Plants |
| Rhynchospora capitellata | Brownish Beakrush | Plants |
| Rhynchospora globularis | NA | Plants |
| Richardsonius egregius | Lahontan redside | Fishes |
| Rickera sorpta | Palestripe Springfly | Insects & other |
| Riparia riparia | Bank Swallow | Birds |
| Robackia demeijeri |  | Insects & other |
| Rorippa columbiae | Columbia Yellowcress | Plants |
| Rorippa curvipes | Rocky Mountain Yellowcress | Plants |
| Rorippa curvisiliqua curvisiliqua | Curve-pod Yellowcress | Plants |
| Rorippa palustris palustris | Bog Yellowcress | Plants |
| Rorippa sphaerocarpa | Round-fruit Yellowcress | Plants |
| Rorippa subumbellata | Tahoe Yellowcress | Plants |
| Rotala ramosior | Toothcup | Plants |
| Rudbeckia klamathensis |  | Plants |
| Rumex britannica | NA | Plants |
| Rumex californicus |  | Plants |
| Rumex conglomeratus | NA | Plants |
| Rumex crassus |  | Plants |
| Rumex fueginus |  | Plants |
| Rumex kerneri | NA | Plants |
| Rumex lacustris |  | Plants |
| Rumex occidentalis |  | Plants |
| Rumex persicarioides |  | Plants |
| Rumex salicifolius salicifolius | Willow Dock | Plants |
| Rumex stenophyllus | NA | Plants |
| Rumex transitorius |  | Plants |
| Rumex triangulivalvis |  | Plants |
| Rumex utahensis |  | Plants |
| Rumex violascens | Violet Dock | Plants |
| Rupisalda dewsi |  | Insects & other |
| Rupisalda saxicola |  | Insects & other |
| Rupisalda teretis |  | Insects & other |
| Ruppia cirrhosa | Widgeon-grass | Plants |
| Ruppia maritima | Ditch-grass | Plants |
| Rynchops niger | Black Skimmer | Birds |
| Sagina saginoides | Arctic Pearlwort | Plants |
| Sagittaria cuneata | Wapatum Arrowhead | Plants |
| Sagittaria latifolia latifolia | Broadleaf Arrowhead | Plants |
| Sagittaria longiloba | Longbarb Arrowhead | Plants |
| Sagittaria montevidensis calycina |  | Plants |
| Sagittaria sanfordii | Sanford's Arrowhead | Plants |
| Salda buenoi |  | Insects & other |
| Salda littoralis |  | Insects & other |
| Salda lugubris |  | Insects & other |
| Salda obscura |  | Insects & other |
| Salda provancheri |  | Insects & other |
| Saldula andrei |  | Insects & other |
| Saldula balli |  | Insects & other |
| Saldula basingeri |  | Insects & other |
| Saldula comatula |  | Insects & other |
| Saldula dispersa |  | Insects & other |
| Saldula explanata |  | Insects & other |
| Saldula latticollis |  | Insects & other |
| Saldula lattini |  | Insects & other |
| Saldula luctuosa |  | Insects & other |
| Saldula nigrita |  | Insects & other |
| Saldula opacula |  | Insects & other |
| Saldula opiparia |  | Insects & other |
| Saldula orbiculata |  | Insects & other |
| Saldula pallipes |  | Insects & other |
| Saldula palustris |  | Insects & other |
| Saldula pexa |  | Insects & other |
| Saldula saltatoria |  | Insects & other |
| Saldula severini |  | Insects & other |
| Saldula sulcicollis |  | Insects & other |
| Saldula usingeri | Wilbur Springs Shorebug | Insects & other |
| Saldula villosa |  | Insects & other |
| Salicornia bigelovii | Dwarf Glasswort | Plants |
| Salicornia rubra | Western Glasswort | Plants |
| Salix babylonica | NA | Plants |
| Salix boothii | Booth's Willow | Plants |
| Salix breweri | Brewer's Willow | Plants |
| Salix delnortensis | Del Norte Willow | Plants |
| Salix drummondiana | Satiny Salix | Plants |
| Salix eastwoodiae | Eastwood's Willow | Plants |
| Salix exigua exigua | Narrowleaf Willow | Plants |
| Salix exigua hindsiana |  | Plants |
| Salix geyeriana | Geyer's Willow | Plants |
| Salix gooddingii | Goodding's Willow | Plants |
| Salix hookeriana | Hooker's Willow | Plants |
| Salix jepsonii | Jepson's Willow | Plants |
| Salix laevigata | Polished Willow | Plants |
| Salix lasiandra caudata |  | Plants |
| Salix lasiandra lasiandra |  | Plants |
| Salix lasiolepis lasiolepis | Arroyo Willow | Plants |
| Salix lemmonii | Lemmon's Willow | Plants |
| Salix lutea | Yellow Willow | Plants |
| Salix melanopsis | Dusky Willow | Plants |
| Salix planifolia | NA | Plants |
| Salix prolixa | Mackenzie's Willow | Plants |
| Salix purpurea | NA | Plants |
| Salix sitchensis | Sitka Willow | Plants |
| Salix tracyi |  | Plants |
| Salmasellus howarthi |  | Crustaceans |
| Salmoperla sylvanica | Bighead Springfly | Insects & other |
| Salvelinus confluentus | Bull Trout | Fishes |
| Salvinia minima | NA | Plants |
| Salvinia oblongifolia | NA | Plants |
| Samolus parviflorus | NA | Plants |
| Sanfilippodytes adelardi | A Predaceous Diving Beetle | Insects & other |
| Sanfilippodytes barbarae |  | Insects & other |
| Sanfilippodytes barbarensis |  | Insects & other |
| Sanfilippodytes belfragei |  | Insects & other |
| Sanfilippodytes bidessoides | A Predaceous Diving Beetle | Insects & other |
| Sanfilippodytes corvallis |  | Insects & other |
| Sanfilippodytes hardyi |  | Insects & other |
| Sanfilippodytes kingi |  | Insects & other |
| Sanfilippodytes latebrosus |  | Insects & other |
| Sanfilippodytes malkini |  | Insects & other |
| Sanfilippodytes palliatus |  | Insects & other |
| Sanfilippodytes rossi |  | Insects & other |
| Sanfilippodytes setifer | A Predaceous Diving Beetle | Insects & other |
| Sanfilippodytes terminalis |  | Insects & other |
| Sanfilippodytes veronicae |  | Insects & other |
| Sanfilippodytes vilis |  | Insects & other |
| Sanfilippodytes williami |  | Insects & other |
| Sarracenia purpurea | NA | Plants |
| Sasquaperla hoopa | A Stonefly | Insects & other |
| Scaphiopus couchii | Couch's Spadefoot | Herps |
| Scheuchzeria palustris | Pod Grass | Plants |
| Schoenoplectus acutus acutus | NA | Plants |
| Schoenoplectus acutus occidentalis | Hardstem Bulrush | Plants |
| Schoenoplectus americanus | Three-square Bulrush | Plants |
| Schoenoplectus californicus | California Bulrush | Plants |
| Schoenoplectus heterochaetus | Slender Bulrush | Plants |
| Schoenoplectus mucronatus | NA | Plants |
| Schoenoplectus pungens longispicatus | Three-square Bulrush | Plants |
| Schoenoplectus pungens pungens | NA | Plants |
| Schoenoplectus saximontanus | Rocky Mountain Bulrush | Plants |
| Schoenoplectus subterminalis | Water Bulrush | Plants |
| Schoenoplectus tabernaemontani | Softstem Bulrush | Plants |
| Schoenoplectus triqueter | NA | Plants |
| Schoenus nigricans | Blacksedge | Plants |
| Scirpus congdonii | Congdon's Bulrush | Plants |
| Scirpus cyperinus | NA | Plants |
| Scirpus diffusus | Umbrella Bulrush | Plants |
| Scirpus microcarpus | Small-fruit Bulrush | Plants |
| Scirpus pendulus | Pendulous Bulrush | Plants |
| Scirtes californicus |  | Insects & other |
| Scirtes orbiculatus |  | Insects & other |
| Scirtes plagiatus |  | Insects & other |
| Scutellaria galericulata | Hooded Skullcap | Plants |
| Sedella leiocarpa | Lake County Mock Stonecrop | Plants |
| Senecio hydrophiloides | Sweet Marsh Ragwort | Plants |
| Senecio hydrophilus | Great Swamp Ragwort | Plants |
| Senecio triangularis | Arrow-leaf Groundsel | Plants |
| Sequoia sempervirens |  | Plants |
| Sergentia albescens |  | Insects & other |
| Serratella levis | A Mayfly | Insects & other |
| Serratella micheneri | A Mayfly | Insects & other |
| Sesbania herbacea |  | Plants |
| Setophaga petechia | Yellow Warbler | Birds |
| Setophaga petechia brewsteri | A Yellow Warbler | Birds |
| Setophaga petechia sonorana | Sonoran Yellow Warbler | Birds |
| Setvena tibialis |  | Insects & other |
| Setvena wahkeena |  | Insects & other |
| Sialis arvalis |  | Insects & other |
| Sialis bilobata |  | Insects & other |
| Sialis californica |  | Insects & other |
| Sialis cornuta |  | Insects & other |
| Sialis hamata |  | Insects & other |
| Sialis nevadensis |  | Insects & other |
| Sialis occidens |  | Insects & other |
| Sialis rotunda |  | Insects & other |
| Sidalcea calycosa calycosa | Annual Checker-mallow | Plants |
| Sidalcea calycosa rhizomata | Point Reyes Checkerbloom | Plants |
| Sidalcea gigantea |  | Plants |
| Sidalcea hirsuta | Hairy Checker-mallow | Plants |
| Sidalcea neomexicana | Rocky Mountain Checker-mallow | Plants |
| Sidalcea oregana hydrophila | Water-loving Checker-mallow | Plants |
| Sidalcea oregana oregana | Oregon Checker-mallow | Plants |
| Sidalcea oregana valida | Kenwood Marsh Checker-mallow | Plants |
| Sidalcea pedata | Pedate Checker-mallow | Plants |
| Sidalcea ranunculacea | Marsh Checker-mallow | Plants |
| Sidalcea reptans | Creeping Checker-mallow | Plants |
| Sierraperla cora | Giant Roachfly | Insects & other |
| Sigara alternata |  | Insects & other |
| Sigara grossolineata |  | Insects & other |
| Sigara krafti |  | Insects & other |
| Sigara mckinstryi | A Water Boatman | Insects & other |
| Sigara nevadensis |  | Insects & other |
| Sigara omani |  | Insects & other |
| Sigara vallis | A Water Boatman | Insects & other |
| Sigara vandykei |  | Insects & other |
| Sigara washingtonensis |  | Insects & other |
| Simulium anduzei |  | Insects & other |
| Simulium apricarium |  | Insects & other |
| Simulium argus |  | Insects & other |
| Simulium balteatum |  | Insects & other |
| Simulium bivittatum |  | Insects & other |
| Simulium brevicercum |  | Insects & other |
| Simulium bricenoi |  | Insects & other |
| Simulium canadensis |  | Insects & other |
| Simulium canonicolum |  | Insects & other |
| Simulium carbunculum |  | Insects & other |
| Simulium chromatinum |  | Insects & other |
| Simulium chromocentrum |  | Insects & other |
| Simulium clarum |  | Insects & other |
| Simulium conicum |  | Insects & other |
| Simulium craigi |  | Insects & other |
| Simulium curiei |  | Insects & other |
| Simulium decorum |  | Insects & other |
| Simulium defoliarti |  | Insects & other |
| Simulium donovani |  | Insects & other |
| Simulium encisoi |  | Insects & other |
| Simulium exculatum |  | Insects & other |
| Simulium freemani |  | Insects & other |
| Simulium griseum |  | Insects & other |
| Simulium hechti |  | Insects & other |
| Simulium hippovorum |  | Insects & other |
| Simulium hunteri |  | Insects & other |
| Simulium infernale |  | Insects & other |
| Simulium iriartei |  | Insects & other |
| Simulium jacumbae |  | Insects & other |
| Simulium joculator |  | Insects & other |
| Simulium longithallum |  | Insects & other |
| Simulium meridionale |  | Insects & other |
| Simulium modicum |  | Insects & other |
| Simulium mysterium |  | Insects & other |
| Simulium nebulosum |  | Insects & other |
| Simulium negativum |  | Insects & other |
| Simulium notatum |  | Insects & other |
| Simulium paynei |  | Insects & other |
| Simulium petersoni |  | Insects & other |
| Simulium pilosum |  | Insects & other |
| Simulium piperi |  | Insects & other |
| Simulium pugetense |  | Insects & other |
| Simulium quadratum |  | Insects & other |
| Simulium rostratum |  | Insects & other |
| Simulium saxosum |  | Insects & other |
| Simulium silvestre |  | Insects & other |
| Simulium tescorum |  | Insects & other |
| Simulium tribulatum |  | Insects & other |
| Simulium twinni |  | Insects & other |
| Simulium vandalicum |  | Insects & other |
| Simulium venator |  | Insects & other |
| Simulium venustum |  | Insects & other |
| Simulium virgatum |  | Insects & other |
| Simulium vittatum |  | Insects & other |
| Simulium wyomingense |  | Insects & other |
| Simulium zephyrus |  | Insects & other |
| Sinapis alba | NA | Plants |
| Siphatales bicolor bicolor | Klamath tui chub | Fishes |
| Siphatales bicolor obesus | Lahontan stream tui chub | Fishes |
| Siphatales bicolor pectinifer | Lahontan lake tui chub | Fishes |
| Siphatales bicolor snyderi | Owens tui chub | Fishes |
| Siphatales bicolor ssp. 1 | Eagle Lake tui chub | Fishes |
| Siphatales bicolor ssp. 11 | High Rock Spring Tui Chub | Fishes |
| Siphatales mohavensis | Mojave tui chub | Fishes |
| Siphatales thalassinus ssp. 1 | Pit River tui chub | Fishes |
| Siphatales thalassinus thalassinus | Goose Lake tui chub | Fishes |
| Siphatales thalassinus vaccaceps | Cow Head tui chub | Fishes |
| Siphlonurus columbianus | A Mayfly | Insects & other |
| Siphlonurus occidentalis | A Mayfly | Insects & other |
| Siphlonurus spectabilis | A Mayfly | Insects & other |
| Sisko oregona |  | Insects & other |
| Sisko sisko |  | Insects & other |
| Sisyra vicaria |  | Insects & other |
| Sisyrinchium californicum | Golden Blue-eyed-grass | Plants |
| Sisyrinchium elmeri | Elmer's Blue-eyed-grass | Plants |
| Sisyrinchium longipes | Timberland Blue-eyed-grass | Plants |
| Sium suave | Hemlock Water-parsnip | Plants |
| Skwala americana | American Springfly | Insects & other |
| Skwala curvata | Curved Springfly | Insects & other |
| Smicridea arizonensis | A Caddisfly | Insects & other |
| Smicridea dispar | A Caddisfly | Insects & other |
| Smicridea fasciatella | A Caddisfly | Insects & other |
| Smicridea signata |  | Insects & other |
| Solidago elongata |  | Plants |
| Solidago guiradonis | Guirado's Goldenrod | Plants |
| Solidago lepida salebrosa |  | Plants |
| Solidago spectabilis | Nevada Goldenrod | Plants |
| Soliperla campanula |  | Insects & other |
| Soliperla quadrispinula | Four-spined Roachfly | Insects & other |
| Soliperla sierra | Sierra Roachfly | Insects & other |
| Soliperla thyra | California Roachfly | Insects & other |
| Soliperla tillamook |  | Insects & other |
| Somatochlora albicincta | Ringed Emerald | Insects & other |
| Somatochlora minor |  | Insects & other |
| Somatochlora semicircularis | Mountain Emerald | Insects & other |
| Sorex palustris | American Water Shrew | Mammals |
| Soyedina interrupta |  | Insects & other |
| Soyedina nevadensis | Nevada Forestfly | Insects & other |
| Soyedina producta | Knobbed Forestfly | Insects & other |
| Sparganium angustifolium | Narrowleaf Bur-reed | Plants |
| Sparganium emersum |  | Plants |
| Sparganium eurycarpum eurycarpum |  | Plants |
| Sparganium eurycarpum greenei |  | Plants |
| Sparganium natans | Small Bur-reed | Plants |
| Spartina densiflora | NA | Plants |
| Spartina foliosa | California Cordgrass | Plants |
| Spartina gracilis | Alkali Cordgrass | Plants |
| Spea hammondii | Western Spadefoot | Herps |
| Spea intermontana | Great Basin Spadefoot | Herps |
| Sperchon stellata |  | Insects & other |
| Sphaerium occidentale |  | Mollusks |
| Sphaerium patella | Rocky Mountain Fingernailclam | Mollusks |
| Sphaerium striatum |  | Mollusks |
| Sphenosciadium capitellatum | Swamp Whiteheads | Plants |
| Spiranthes romanzoffiana | Hooded Ladies'-tresses | Plants |
| Spirinchus thaleichthys | Longfin smelt | Fishes |
| Spirodela polyrhiza | NA | Plants |
| Stachys ajugoides | Bugle Hedge-nettle | Plants |
| Stachys albens | White-stem Hedge-nettle | Plants |
| Stachys chamissonis chamissonis | Coast Hedge-nettle | Plants |
| Stachys pycnantha | Short-spike Hedge-nettle | Plants |
| Stachys rigida quercetorum |  | Plants |
| Stachys stricta | Sonoma Hedge-nettle | Plants |
| Stactobiella brustia |  | Insects & other |
| Stactobiella delira | A Caddisfly | Insects & other |
| Stactobiella palmata |  | Insects & other |
| Stagnicola caperata | Wrinkled Marshsnail | Mollusks |
| Stagnicola elodes | Marsh Pondsnail | Mollusks |
| Stagnicola gabbi | Striate Pondsnail | Mollusks |
| Stagnicola traski | Widelip Pondsnail | Mollusks |
| Stegopterna acra |  | Insects & other |
| Stegopterna permutata |  | Insects & other |
| Stegopterna xantha |  | Insects & other |
| Stellaria littoralis | Beach Starwort | Plants |
| Stemodia durantifolia | White-woolly Stemodia | Plants |
| Stenelmis calida calida | Devil's Hole Warm Spring Riffle Beetle | Insects & other |
| Stenelmis lariversi |  | Insects & other |
| Stenelmis moapa |  | Insects & other |
| Stenelmis occidentalis |  | Insects & other |
| Stenochironomus colei |  | Insects & other |
| Stenochironomus fuscipatellus |  | Insects & other |
| Stenochironomus hilaris |  | Insects & other |
| Stenochironomus totifuscus |  | Insects & other |
| Stenocolus scutellaris |  | Insects & other |
| Stenocypris archoplites | An Ostracod | Crustaceans |
| Stictochironomus naevus |  | Insects & other |
| Stictochironomus quagga |  | Insects & other |
| Stictotarsus aequinoctialis |  | Insects & other |
| Stictotarsus coelamboides |  | Insects & other |
| Stictotarsus corvinus |  | Insects & other |
| Stictotarsus decemsignatus |  | Insects & other |
| Stictotarsus deceptus |  | Insects & other |
| Stictotarsus dolerosus |  | Insects & other |
| Stictotarsus eximius |  | Insects & other |
| Stictotarsus expositus |  | Insects & other |
| Stictotarsus funereus |  | Insects & other |
| Stictotarsus griseostriatus |  | Insects & other |
| Stictotarsus panaminti |  | Insects & other |
| Stictotarsus roffi |  | Insects & other |
| Stictotarsus spectabilis |  | Insects & other |
| Stictotarsus striatellus |  | Insects & other |
| Streptocephalus dorothae | New Mexico Fairy Shrimp | Crustaceans |
| Streptocephalus mackini |  | Crustaceans |
| Streptocephalus sealii | Spinytail Fairy Shrimp | Crustaceans |
| Streptocephalus texanus | Greater Plains Fairy Shrimp | Crustaceans |
| Streptocephalus woottoni | Riverside Fairy Shrimp | Crustaceans |
| Streptopus amplexifolius americanus |  | Plants |
| Strix nebulosa | Great Gray Owl | Birds |
| Stuckenia filiformis alpina |  | Plants |
| Stuckenia pectinata |  | Plants |
| Stuckenia striata |  | Plants |
| Stygalbiella affinis |  | Insects & other |
| Stygalbiella arizonica |  | Insects & other |
| Stygobromus cherylae | Barr's Amphipod | Crustaceans |
| Stygobromus cowani | Cowan's Amphipod | Crustaceans |
| Stygobromus gallawayae | Gallaway's Amphipod | Crustaceans |
| Stygobromus gradyi | Grady's Cave Amphipod | Crustaceans |
| Stygobromus grahami | A Cave Obligate Amphipod | Crustaceans |
| Stygobromus harai | Hara's Cave Amphipod | Crustaceans |
| Stygobromus hyporheicus | Hypoheic Amphipod | Crustaceans |
| Stygobromus imperialis | Imperial Amphipod | Crustaceans |
| Stygobromus lacicolus | Lake Tahoe Amphipod | Crustaceans |
| Stygobromus mackenziei | Mackenzie's Cave Amphipod | Crustaceans |
| Stygobromus myersae | Myers' Amphipod | Crustaceans |
| Stygobromus mysticus | A Cave Obligate Amphipod | Crustaceans |
| Stygobromus rudolphi | Rudolph's Amphipod | Crustaceans |
| Stygobromus sheldoni | Sheldon Stygobromid | Crustaceans |
| Stygobromus sierrensis | A Cave Obligate Amphipod | Crustaceans |
| Stygobromus tahoensis | Lake Tahoe Stygobromid | Crustaceans |
| Stygobromus trinus | Trinity County Amphipod | Crustaceans |
| Stygobromus wengerorum | Wenger Cave Stygobromid | Crustaceans |
| Stygonyx courtneyi |  | Crustaceans |
| Stygoporus oregonensis |  | Insects & other |
| Stylurus intricatus | Brimstone Clubtail | Insects & other |
| Stylurus olivaceus | Olive Clubtail | Insects & other |
| Stylurus plagiatus | Russet-tipped Clubtail | Insects & other |
| Suaeda calceoliformis | American Sea-blite | Plants |
| Suaeda californica | California Sea-blite | Plants |
| Suaeda esteroa | Estuary Suaeda | Plants |
| Sublettea coffmani |  | Insects & other |
| Subularia aquatica americana | Water Awlwort | Plants |
| Suphisellus bicolor |  | Insects & other |
| Susulus venustus | Beautiful Springfly | Insects & other |
| Suwallia amoenacolens |  | Insects & other |
| Suwallia autumna |  | Insects & other |
| Suwallia dubia | Pale Sallfly | Insects & other |
| Suwallia lineosa |  | Insects & other |
| Suwallia pallidula | Yellow Sallfly | Insects & other |
| Suwallia shepardi | A Stonefly | Insects & other |
| Suwallia sierra | Sierra Sallfly | Insects & other |
| Suwallia starki |  | Insects & other |
| Suwallia sublimis | A Stonefly | Insects & other |
| Sweltsa adamantea |  | Insects & other |
| Sweltsa borealis | Boreal Sallfly | Insects & other |
| Sweltsa californica | Chico Sallfly | Insects & other |
| Sweltsa coloradensis | Colorado Sallfly | Insects & other |
| Sweltsa continua | Gabriel Sallfly | Insects & other |
| Sweltsa exquisita |  | Insects & other |
| Sweltsa fidelis | Mountain Sallfly | Insects & other |
| Sweltsa lamba |  | Insects & other |
| Sweltsa occidens |  | Insects & other |
| Sweltsa oregonensis |  | Insects & other |
| Sweltsa pacifica | Pacific Sallfly | Insects & other |
| Sweltsa pisteri | Coastal Sallfly | Insects & other |
| Sweltsa resima | California Sallfly | Insects & other |
| Sweltsa revelstoka |  | Insects & other |
| Sweltsa salix | A Stonefly | Insects & other |
| Sweltsa tamalpa | Tamalpais Sallfly | Insects & other |
| Sweltsa townesi | Sierra Sallfly | Insects & other |
| Sweltsa umbonata | Shasta Sallfly | Insects & other |
| Sweltsa yurok | A Stonefly | Insects & other |
| Symbiocladius equitans |  | Insects & other |
| Sympetrum corruptum | Variegated Meadowhawk | Insects & other |
| Sympetrum costiferum | Saffron-winged Meadowhawk | Insects & other |
| Sympetrum danae | Black Meadowhawk | Insects & other |
| Sympetrum illotum | Cardinal Meadowhawk | Insects & other |
| Sympetrum internum | Cherry-faced Meadowhawk | Insects & other |
| Sympetrum madidum | Red-veined Meadowhawk | Insects & other |
| Sympetrum obtrusum | White-faced Meadowhawk | Insects & other |
| Sympetrum occidentale |  | Insects & other |
| Sympetrum pallipes | Striped Meadowhawk | Insects & other |
| Sympetrum signiferum |  | Insects & other |
| Sympetrum vicinum | Autumn Meadowhawk | Insects & other |
| Symphyotrichum bracteolatum |  | Plants |
| Symphyotrichum frondosum | Alkali Aster | Plants |
| Symphyotrichum lanceolatum hesperium | Siskiyou Aster | Plants |
| Symphyotrichum lanceolatum lanceolatum | NA | Plants |
| Symphyotrichum lentum | Suisun Marsh Aster | Plants |
| Sympotthastia diastena |  | Insects & other |
| Syncaris pacifica | California Freshwater Shrimp | Crustaceans |
| Syncaris pasadenae | Pasadena Freshwater Shrimp | Crustaceans |
| Synendotendipes luski |  | Insects & other |
| Tachycineta bicolor | Tree Swallow | Birds |
| Taenionema californicum | California Willowfly | Insects & other |
| Taenionema grinnelli | Angeles Willowfly | Insects & other |
| Taenionema jacobii |  | Insects & other |
| Taenionema jeanae | A Stonefly | Insects & other |
| Taenionema jewetti |  | Insects & other |
| Taenionema kincaidi | Pale Willowfly | Insects & other |
| Taenionema oregonense |  | Insects & other |
| Taenionema pacificum | Pacific Willowfly | Insects & other |
| Taenionema pallidum | Common Willowfly | Insects & other |
| Taenionema raynorium | Yosemite Willowfly | Insects & other |
| Taenionema uinta |  | Insects & other |
| Taenionema umatilla |  | Insects & other |
| Taeniopteryx nivalis | Boreal Willowfly | Insects & other |
| Talitroides alluaudi |  | Crustaceans |
| Talitroides topitotum |  | Crustaceans |
| Tanypteryx hageni | Black Petaltail | Insects & other |
| Tanypus carinatus |  | Insects & other |
| Tanypus grodhausi |  | Insects & other |
| Tanypus imperialis |  | Insects & other |
| Tanypus neopunctipennis |  | Insects & other |
| Tanypus nubifer |  | Insects & other |
| Tanypus parastellatus |  | Insects & other |
| Tanypus punctipennis |  | Insects & other |
| Tanypus stellatus |  | Insects & other |
| Tanytarsus angulatus |  | Insects & other |
| Tanytarsus challeti |  | Insects & other |
| Tanytarsus dendyi |  | Insects & other |
| Tanytarsus hastatus |  | Insects & other |
| Tanytarsus limneticus |  | Insects & other |
| Tanytarsus mendax |  | Insects & other |
| Tanytarsus neoflavellus |  | Insects & other |
| Tanytarsus pelsuei |  | Insects & other |
| Taricha granulosa | Rough-skinned Newt | Herps |
| Taricha rivularis | Red-bellied Newt | Herps |
| Taricha sierrae | Sierra Newt | Herps |
| Taricha torosa | Coast Range Newt | Herps |
| Taxus brevifolia |  | Plants |
| Telebasis salva | Desert Firetail | Insects & other |
| Telmatogeton alaskensis |  | Insects & other |
| Telmatogeton japonicus |  | Insects & other |
| Telmatogeton macswaini |  | Insects & other |
| Telmatogeton spinosus |  | Insects & other |
| Telmatogeton trilobatus |  | Insects & other |
| Teloleuca bifasciata |  | Insects & other |
| Teloleuca pellucens |  | Insects & other |
| Tempisquitoneura merrillorum |  | Insects & other |
| Tethymyia aptena |  | Insects & other |
| Thalassosmittia clavicornis |  | Insects & other |
| Thalassosmittia marina |  | Insects & other |
| Thalassosmittia pacifica |  | Insects & other |
| Thalassotrechus barbarae |  | Insects & other |
| Thaleichthys pacificus | Eulachon | Fishes |
| Thamnocephalus mexicanus |  | Crustaceans |
| Thamnocephalus platyurus | Beavertail Fairy Shrimp | Crustaceans |
| Thamnophis atratus atratus | Santa Cruz Gartersnake | Herps |
| Thamnophis atratus hydrophilius | Oregon Gartersnake | Herps |
| Thamnophis atratus zaxanthus | Diablo Range Gartersnake | Herps |
| Thamnophis couchii | Sierra Gartersnake | Herps |
| Thamnophis elegans elegans | Mountain Gartersnake | Herps |
| Thamnophis elegans terrestris | Coast Gartersnake | Herps |
| Thamnophis elegans vagrans | Wandering Gartersnake | Herps |
| Thamnophis gigas | Giant Gartersnake | Herps |
| Thamnophis hammondii hammondii | Two-striped Gartersnake | Herps |
| Thamnophis hammondii ssp. 1 | Santa Catalina Gartersnake | Herps |
| Thamnophis marcianus marcianus | Marcy's Checkered Gartersnake | Herps |
| Thamnophis ordinoides | Northwestern Gartersnake | Herps |
| Thamnophis sirtalis fitchi | Valley Gartersnake | Herps |
| Thamnophis sirtalis infernalis | California Red-sided Gartersnake | Herps |
| Thamnophis sirtalis sirtalis | Common Gartersnake | Herps |
| Thamnophis sirtalis ssp. 1 | South Coast Gartersnake | Herps |
| Thamnophis sirtalis tetrataenia | San Francisco Gartersnake | Herps |
| Thelypteris puberula sonorensis | NA | Plants |
| Thermonectus intermedius |  | Insects & other |
| Thermonectus marmoratus |  | Insects & other |
| Thermonectus nigrofasciatus nigrofasciatus | | Insects & other |
| Thermonectus sibleyi |  | Insects & other |
| Thienemannimyia barberi |  | Insects & other |
| Thienemannimyia fusciceps |  | Insects & other |
| Thienemannimyia norena |  | Insects & other |
| Thraulodes brunneus |  | Insects & other |
| Thraulodes gonzalesi |  | Insects & other |
| Thraulodes tenulineus |  | Insects & other |
| Throscinus crotchi |  | Insects & other |
| Timpanoga hecuba | A Mayfly | Insects & other |
| Tinodes belisus | A Caddisfly | Insects & other |
| Tinodes cascadius | A Caddisfly | Insects & other |
| Tinodes consuetus | A Caddisfly | Insects & other |
| Tinodes gabriella | A Caddisfly | Insects & other |
| Tinodes parvulus | A Caddisfly | Insects & other |
| Tinodes powelli | A Caddisfly | Insects & other |
| Tinodes provo | A Caddisfly | Insects & other |
| Tinodes schusteri | A Caddisfly | Insects & other |
| Tinodes sigodanus | A Caddisfly | Insects & other |
| Tinodes siskiyou | A Caddisfly | Insects & other |
| Tinodes twilus | A Caddisfly | Insects & other |
| Tinodes usillus | A Caddisfly | Insects & other |
| Tlalocomyia andersoni |  | Insects & other |
| Tlalocomyia osbornii |  | Insects & other |
| Tlalocomyia ramifera |  | Insects & other |
| Tlalocomyia stewarti |  | Insects & other |
| Torreyochloa pallida | NA | Plants |
| Toxicoscordion fontanum | NA | Plants |
| Toxicoscordion micranthum | NA | Plants |
| Toxicoscordion venenosum venenosum |  | Plants |
| Toxorhynchites moctezuma |  | Insects & other |
| Tramea calverti |  | Insects & other |
| Tramea lacerata | Black Saddlebags | Insects & other |
| Tramea onusta | Red Saddlebags | Insects & other |
| Traverella albertana |  | Insects & other |
| Trepobates becki |  | Insects & other |
| Trepobates pictus |  | Insects & other |
| Trepobates taylori |  | Insects & other |
| Trepobates trepidus |  | Insects & other |
| Triaenodes frontalis |  | Insects & other |
| Triaenodes injustus |  | Insects & other |
| Triaenodes reuteri |  | Insects & other |
| Triaenodes tardus | A Caddisfly | Insects & other |
| Tribelos jucundum |  | Insects & other |
| Tribelos subatrum |  | Insects & other |
| Tribelos subletteorum |  | Insects & other |
| Trichocorixa arizonensis |  | Insects & other |
| Trichocorixa calva |  | Insects & other |
| Trichocorixa reticulata |  | Insects & other |
| Trichocorixa uhleri |  | Insects & other |
| Trichocorixa verticalis |  | Insects & other |
| Tricoryhyphes condylus |  | Insects & other |
| Tricorythodes explicatus | A Mayfly | Insects & other |
| Tricorythodes fictus | A Mayfly | Insects & other |
| Triglochin maritima | Common Bog Arrow-grass | Plants |
| Triglochin palustris | Slender Bog Arrow-grass | Plants |
| Triglochin scilloides | NA | Plants |
| Triglochin striata | Three-ribbed Arrow-grass | Plants |
| Tringa melanoleuca | Greater Yellowlegs | Birds |
| Tringa semipalmata | Willet | Birds |
| Tringa solitaria | Solitary Sandpiper | Birds |
| Triops longicaudatus | Summer tadpole shrimps | Crustaceans |
| Triznaka pintada | Rough Sallfly | Insects & other |
| Triznaka sheldoni |  | Insects & other |
| Triznaka signata |  | Insects & other |
| Tropicus pusillus |  | Insects & other |
| Tropisternus californicus |  | Insects & other |
| Tropisternus columbianus |  | Insects & other |
| Tropisternus ellipticus |  | Insects & other |
| Tropisternus lateralis |  | Insects & other |
| Tropisternus orvus |  | Insects & other |
| Tropisternus salsamentus |  | Insects & other |
| Tropisternus sublaevis |  | Insects & other |
| Tryonia margae | Grapevine Springs Elongate Tryonia | Mollusks |
| Tryonia porrecta | Desert Tryonia | Mollusks |
| Tryonia rowlandsi | Grapevine Springs Squat Tryonia | Mollusks |
| Tryonia salina | Cottonball Marsh Tryonia | Mollusks |
| Tryonia variegata | Amargosa Tryonia | Mollusks |
| Tuctoria greenei | Green's Awnless Orcutt Grass | Plants |
| Tuctoria mucronata | Mucronate Orcutt Grass | Plants |
| Tvetenia vitracies |  | Insects & other |
| Twinnia hirticornis |  | Insects & other |
| Typha domingensis | Southern Cattail | Plants |
| Typha latifolia | Broadleaf Cattail | Plants |
| Uca crenulata |  | Crustaceans |
| Uranotaenia anhydor |  | Insects & other |
| Utacapnia columbiana | Columbian Snowfly | Insects & other |
| Utacapnia imbera |  | Insects & other |
| Utacapnia lemoniana |  | Insects & other |
| Utacapnia sierra | Sierra Snowfly | Insects & other |
| Utacapnia tahoensis | Tahoe Snnowflyl | Insects & other |
| Utaperla sopladora |  | Insects & other |
| Utaxatax californiensis |  | Insects & other |
| Utaxatax newelli |  | Insects & other |
| Utaxatax ovalis |  | Insects & other |
| Utricularia gibba | Humped Bladderwort | Plants |
| Utricularia intermedia | Flatleaf Bladderwort | Plants |
| Utricularia macrorhiza | Greater Bladderwort | Plants |
| Utricularia minor | Lesser Bladderwort | Plants |
| Utricularia ochroleuca | Northern Bladderwort | Plants |
| Utricularia subulata | NA | Plants |
| Uvarus amandus |  | Insects & other |
| Uvarus subtilis |  | Insects & other |
| Vaccinium macrocarpon | NA | Plants |
| Vaccinium uliginosum occidentale |  | Plants |
| Vaccupernius packeri |  | Insects & other |
| Valvata humeralis | Glossy Valvata | Mollusks |
| Valvata tricarinata |  | Mollusks |
| Valvata utahensis |  | Mollusks |
| Valvata virens | Emerald Valvata | Mollusks |
| Veratrum fimbriatum | Fringed False Hellebore | Plants |
| Verbena scabra | Sandpaper Vervain | Plants |
| Veronica americana | American Speedwell | Plants |
| Veronica anagallis-aquatica | NA | Plants |
| Veronica catenata | NA | Plants |
| Veronica peregrina | NA | Plants |
| Veronica scutellata | Marsh-speedwell | Plants |
| Vertigo ovata | Ovate Vertigo | Mollusks |
| Vespericola armiger | Santa Cruz Hesperian | Mollusks |
| Vespericola embertoni | Reeves Canyon Hesperian Snail | Mollusks |
| Vespericola eritrichius | Velvet Hesperian | Mollusks |
| Vespericola euthales | A Terrestrial Snail | Mollusks |
| Vespericola haplus | Butte Creek Hesperian | Mollusks |
| Vespericola karokorum | Karok Hesperian | Mollusks |
| Vespericola klamathicus | Klamath Hesperian | Mollusks |
| Vespericola marinensis | Marin Hesperian | Mollusks |
| Vespericola megasoma | Redwood Hesperian | Mollusks |
| Vespericola orius | El Dorado Hesperian | Mollusks |
| Vespericola pilosus | Brushfield Hesperian | Mollusks |
| Vespericola pinicola | Monterey Hesperian | Mollusks |
| Vespericola pressleyi | Big Bar Hesperian | Mollusks |
| Vespericola rhodophila | Azalea Hesperian Snail | Mollusks |
| Vespericola rothi | Ellery Creek Hesperian | Mollusks |
| Vespericola sasquatch | Sasquatch Hesperian Snail | Mollusks |
| Vespericola scotti | Benson Gulch Hesperian | Mollusks |
| Vespericola shasta | Shasta Hesperian | Mollusks |
| Vespericola sierranus | Siskiyou Hesperian | Mollusks |
| Viola langsdorffii | NA | Plants |
| Viola macloskeyi | NA | Plants |
| Vireo bellii | Bell's Vireo | Birds |
| Vireo bellii arizonae | Arizona Bell's Vireo | Birds |
| Vireo bellii pusillus | Least Bell's Vireo | Birds |
| Visoka cataractae | Cataract Forestfly | Insects & other |
| Vorticifex effusa effusa | Artemesian Rams-horn | Mollusks |
| Vorticifex solida | A Freshwater Snail | Mollusks |
| Wolffia arrhiza | NA | Plants |
| Wolffia borealis | Dotted Watermeal | Plants |
| Wolffia brasiliensis | Pointed Watermeal | Plants |
| Wolffia columbiana | Columbian Watermeal | Plants |
| Wolffia globosa | Asian Watermeal | Plants |
| Wolffiella lingulata | Tongue Bogmat | Plants |
| Wolffiella oblonga | Saber-shape Bogmat | Plants |
| Wormaldia anilla | A Caddisfly | Insects & other |
| Wormaldia arizonensis |  | Insects & other |
| Wormaldia birneyi | A Caddisfly | Insects & other |
| Wormaldia gabriella | A Caddisfly | Insects & other |
| Wormaldia gesugta | A Caddisfly | Insects & other |
| Wormaldia hamata | A Caddisfly | Insects & other |
| Wormaldia laona | A Caddisfly | Insects & other |
| Wormaldia occidea | A Caddisfly | Insects & other |
| Wormaldia pachita | A Caddisfly | Insects & other |
| Xanthocephalus xanthocephalus | Yellow-headed Blackbird | Birds |
| Xenelmis sandersoni |  | Insects & other |
| Xenochironomus xenolabis |  | Insects & other |
| Xenopelopia tincta |  | Insects & other |
| Xyrauchen texanus | Razorback sucker | Fishes |
| Yoraperla brevis | Least Roachfly | Insects & other |
| Yoraperla mariana |  | Insects & other |
| Yoraperla nigrisoma | Black Roachfly | Insects & other |
| Yoraperla siletz | Coastal Roachfly | Insects & other |
| Yphria californica | A Caddisfly | Insects & other |
| Zaitzevia parvula |  | Insects & other |
| Zaitzevia posthonia |  | Insects & other |
| Zannichellia palustris | Horned Pondweed | Plants |
| Zapada cinctipes | Common Forestfly | Insects & other |
| Zapada columbiana | Columbian Forestfly | Insects & other |
| Zapada cordillera | Cordilleran Forestfly | Insects & other |
| Zapada frigida | Frigid Forestfly | Insects & other |
| Zapada haysi | Intermountain Forestfly | Insects & other |
| Zapada oregonensis | Oregon Forestfly | Insects & other |
| Zavrelimyia sinuosa |  | Insects & other |
| Zavrelimyia thryptica |  | Insects & other |
| Zizania palustris interior | NA | Plants |
| Zizania palustris palustris | NA | Plants |
| Zoniagrion exclamationis | Exclamation Damsel | Insects & other |
| Zumatrichia notosa |  | Insects & other |
